# Fragments of the key flowering gene *GIGANTEA *are associated with helitron-type sequences in the Pooideae grass *Lolium perenne*

**DOI:** 10.1186/1471-2229-9-70

**Published:** 2009-06-07

**Authors:** Tim Langdon, Ann Thomas, Lin Huang, Kerrie Farrar, Julie King, Ian Armstead

**Affiliations:** 1 Institute of Biological, Environmental and Rural Sciences, Gogerddan Campus, Aberystwyth University, Ceredigion, SY23 3EB, UK

## Abstract

**Background:**

Helitrons are a class of transposable elements which have been identified in a number of species of plants, animals and fungi. They are unique in their proposed rolling-circle mode of replication, have a highly variable copy-number and have been implicated in the restructuring of coding sequences both by their insertion into existing genes and by their incorporation of transcriptionally competent gene fragments. Helitron discovery depends on identifying associated DNA signature sequences and comprehensive evaluation of helitron contribution to a particular genome requires detailed computational analysis of whole genome sequence. Therefore, the role which helitrons have played in modelling non-model plant genomes is largely unknown.

**Results:**

Cloning of the flowering gene *GIGANTEA *(*GI*) from a BAC library of the Pooideae grass *Lolium perenne *(perennial ryegrass) identified the target gene and several *GI *pseudogene fragments spanning the first five exons. Analysis of genomic sequence 5' and 3' of one these *GI *fragments revealed motifs consistent with helitron-type transposon insertion, specifically a putative 5'-A↓T-3' insertion site containing 5'-TC and CTAG-3' borders with a sub-terminal 16 bp hairpin. Screening of a BAC library of the closely related grass species *Festuca pratensis *(meadow fescue) indicated similar helitron-associated *GI *fragments present in this genome, as well as non-helitron associated *GI *fragments derived from the same region of *GI*. In order to investigate the possible extent of ancestral helitron-activity in *L. perenne*, a methylation-filtered GeneThresher^® ^genomic library developed from this species was screened for potential helitron 3' hairpin sequences associated with a 3'-CTRR motif. This identified 7 potential helitron hairpin-types present between at least 9 and 51 times within the *L. perenne *methylation-filtered library.

**Conclusion:**

This represents evidence for a possible ancestral role for helitrons in modelling the genomes of *Lolium *and related species.

## Background

Helitrons are a class of transposons which are unique in their proposed rolling-circle mode of replication mediated either autonomously by an internally coded putative DNA replication-initiator-helicase protein, or non-autonomously. They have been identified in a number of species of plants, animals and fungi and can have a highly variable copy-number, from an infrequent representation in many mammals to contributing up to 5% of the genome size in some *Drosophila *species (see reviews by [1,2]). They show considerable size variation (0.5 – > 15 kb for *Arabidopsis *helitrons, [3]) and, unusually, helitron transposition does not give rise to duplication of target sites. Helitrons insert within 5'-A↓T-3' target sites within the genome and can be recognised by conserved 5'-TC.. and ..CTRR-3' termini with, typically, 16–20 bp hairpin motifs 8–12 bp from the 3' termini.

A feature of helitron transposons is their ability to incorporate multiple genomic gene fragments which can still show transcriptional activity – thus creating the potential for novel truncated, alternatively spliced and chimeric mRNAs and proteins [4]. The mechanism by which helitrons incorporate gene fragments is not clear, though it is presumably associated with mutation or misidentification of recognition sites during the replication process, and models which describe the acquisition of gene fragments both at the 5' and at the 3' end have been proposed [1-4]. In rice, *Arabidopsis *and maize, the extensive genome resources have facilitated *in silico *identification of helitrons in these and related genera [3,5-7]. Helitrons identified in maize [4,8-12] and *Ipomoea tricolor *[13] have generated particular interest due to their proposed actions in creating haplotypic diversity and influencing gene function.

*Lolium perenne *(perennial ryegrass) and *Festuca pratensis *(meadow fescue) are members of the '*Lolium*/*Festuca *complex' of interfertile grasses which form the basis of many grassland agricultural and amenity systems in temperate areas of the world. They belong to the Pooideae sub-family of the Poaceae, along with the Triticeae cereal crops and *Brachypodium distachyon*, the rapidly developing model for monocot species. The haploid genome sizes of *L. perenne *and *F. pratensis *are estimated to be c. 2 Gb [14,15], less than half the size of barley and the constituent genomes of hexaploid wheat [16,17] but c. 6–7 times the size of *B. distachyon *and rice [16]. Consequently, the intermediate genome sizes of *L. perenne and F. pratensis *between *B. distachyon *and the Triticeae cereals and the close evolutionary interrelationships of these Pooideae species, makes the *Lolium/Festuca *grasses of great interest in terms of understanding the processes which influence the evolution of genome organisation and size in close relatives.

*GIGANTEA *(*GI*) was originally identified as a key gene in the perception of circadian rhythms and the photoperiodic control of flowering by mutation analysis in *Arabidopsis *[18,19]but it is only recently that detailed knowledge of the mode of action and interaction of this gene has become available [20-23]. Comparative genome analysis between dicots and monocots has indicated that orthologues of many of the key genes involved in flowering in *Arabidopsis *also exist in rice and other monocots [24-26] and experimental evidence indicates that similar control mechanisms may be involved in some cases [27-31] including for *GI *[32,33]. Consequently, the identification of the orthologues of *GI *in *L. perenne *and other monocot crop species has been a desirable goal, partly to ascertain if it is implicated in flowering control in current breeding populations through QTL/genetic mapping studies but also to identify allelic variants which may be useful in future population development.

In this study we describe how, in the process of cloning the *L. perenne *orthologue of *GI *from a BAC library, we identified *GI *pseudogene fragments associated with helitron-type sequences. Similar sequences were found to be also present in the *F. pratensis *genome. Additionally, we describe the use of a methylation-filtered *L. perenne *genomic library in an initial survey to ascertain the potential frequency of helitrons within the *L. perenne *genome.

## Results

### Identification of *GI *and *GI *pseudogene sequences from *L. perenne *and *F. pratensis *BAC libraries

A primer pair, GIG49660.6F/7R (see Table 1 for primer sequences) was designed based on conserved regions spanning the first and fourth exons in existing *GI *sequences from other monocot species. This primer pair was tested on a range of genotypes from a *L. perenne *mapping family (see Methods) and two distinct, non-segregating bands of 525 and 536 bp were amplified. Sequencing of these PCR products indicated the 536 bp was likely to be a fragment of the expected *GI *gene, whereas the 525 bp band consisted of an apparent *GI *pseudogene fragment. PCR screening of an *L. perenne *BAC library (5 genome equivalents) with a second primer pair, GIGgt2F/2R, designed directly upon derived *L. perenne *genomic sequence, estimated between 4 and 5 GIGgt2F/2R priming sites *per *genome (see Additional File 1 for derivation of this estimate). Four GIGgt2F/2R-positive BAC clones were isolated from the library; one contained *GIGANTEA (LpGI) *and 3 contained apparently non-allelic *GI *pseudogene fragments (Lp-*psGI*1–3). Primer pair GIGgt2F/2R was also screened on the 2.5 genome equivalent *F. pratensis *BAC library and, again, an estimate of 4–5 priming sites per genome was obtained (see Additional File 1). However, the PCR products amplified from the *F. pratensis *BAC library were of two distinct types, one type in the expected range and the other type smaller than expected. This latter type was subsequently confirmed by sequencing to be a truncated version of the *GI *pseudogene.

**Table 1:** PCR primer sequences, 5'....3'^1^

GIG49660.6F	GTCCCGTCTATGATGCGTGA	GIG49660.7R	CCAGTTCTCATCACTGTTCTGG
GIGgt.1F	ATTCCTGCATCTGAAACCAC	GIGgt.1R	CAGCCAGCACATACGAGTC
GIGgt.2F	GCATCAAATGGGAAGTGGAT	GIGgt.2R	TGCAACTTTGAAGATTGGCC

^1^Thermal cycling profile for all primer pairs was as follows: 1 minute at 94°C, followed by 10 cycles of 1 min at 94°C, 1 min at 60°C (with the temperature reduced by 1°C per cycle), 1 min at 72°C, followed by 30 cycles of 1 min at 94°C, 1 min at 50°C, 1 min at 72°C

Both BAC libraries were also screened with the *GI *specific primer pair GIGgt1F/1R. and the assay results estimated 1–2 copies *per *genome for the *L. perenne *library and 1 copy *per *genome for the *F. pratensis *library (see Additional File 1). All the BAC library DNA screening pools identified by primer pair GIGgt1F/1R in both libraries were also identified by primer pair GIGgt2F/2R, indicating that both *Lp/FpGI *and *Lp/Fp*-psGI sequences were amplified by the latter primer pair.

### *LpGI *sequence analysis

The region of one the BACs containing the *LpGI *gene (identified by the GIGgt1F/1R screen) was sequenced directly and the genomic region containing *LpGI *identified. The gene structure was predicted with FGENESH+, using an existing *L. perenne *GI protein sequence (ABF83898) as template and spanned 6024 bp from initiator to terminator codons. Fourteen exons coded for a protein of 1148aa which showed 99% homology with the existing *L. perenne *GI protein sequence (ABF83898) and 92%, 91% 88% and 66% with homologous GI sequences from barley (AAW66946), wheat (AAQ11738,) rice (BAF04134) and *Arabidopsis *(ABP96502), respectively (Additional File 2). *LpGI *was mapped to chromosome 3 of a *L. perenne *mapping family to a position compatible with the known syntenic relationship between *L. perenne *chromosome 3 and rice chromosome 1 (King et al., 2007; J. King, unpublished data).

### Helitron-like sequences in *Lp*-psGI.1–3

**Figure 1:**
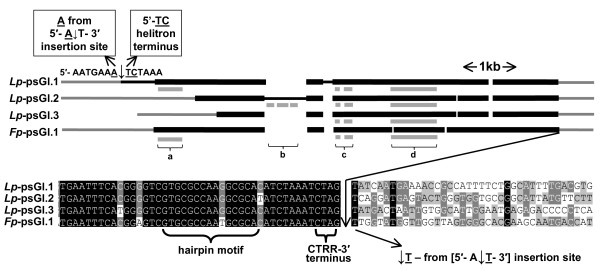
***Lolium perenne *and *Festuca pratensis *helitron sequences containing *GIGANTEA *gene fragment**. Helitron sequences conserved between *Lp-*psGI.1 and/or *Lp-*psGI.2/.3 and *Fp*-psGI.1 (thick black bar); helitron sequence unique to *Lp-*psGI.1 (thin black bar); non-helitron genomic sequence (thin grey bar); putative gene fragments (thick grey bar): a = succinate dehydrogenase, b = non-LTR retroelement, c = ribosomal protein, d = *GIGANTEA*. *Sequence*: detail of 3' helitron border illustrating hairpin motif and 3' terminus.

Between c. 8 and 11 kb of the 3 BACs containing the different *Lp*-psGI fragments (*Lp*-psGI.1–3) were sequenced directly from the BAC. Alignment of these sequences identified regions of partial homology between *Lp*-psGI.1 and *Lp*-psGI.2 of c. 6 kb and between *Lp*-psGI.1/.2 and *Lp*-psGI.3 of c. 5.6 kb. Insertions of c. 0.8 kb and 0.2 kb interrupted the homologous regions in *Lp*-psGI.1 and *Lp*-psGI.2, respectively. The 3' end of the homologous regions were terminated in all the *Lp*-psGI sequences by conserved regions containing a 14 bp motif (16 bp in *Lp*-psGI.1 and *Lp*-psGI.3) capable of forming a hairpin structure – characteristic of the 3' termini of helitron-like transposons (Fig. 1).

**Figure 2:**
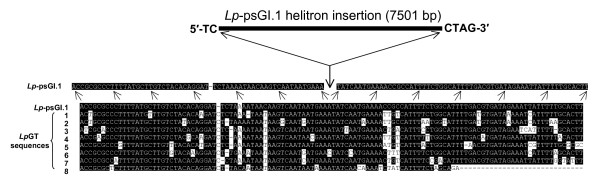
**Sequences derived from the *L. perenne *GeneThresher library (*Lp*GT) with homology to flanking regions of the complete helitron sequence *Lp*-psGI.1**. Identifiers for the *LpGT *sequences are: 1) FLPB002709C17-g0RSP_20020409, 2) FLPB002048C23-g0RSP_20011109, 3) FLPB002662H10-b0FSP_20020409, 4) FLPB001026M06-g0RSP_20010815, 5) FLPB001057C01-g1RSP_20010815, 6) FLPB001013B03-g0RSP_20010815, 7) FLPB002024D17-b0FSP_20010827, 8) FLPB001091D09-b0FSP_20011203 (see Additional File 5).

**Figure 3:**
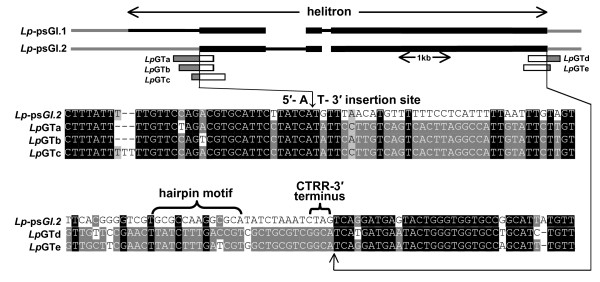
**Diagrammatic representation and sequence details of alignments between *Lp*GT sequences and *Lp*-psGI.2, indicating possible ancestral 5' (left) and 3' (right) helitron borders**.*Diagram*: sequence within helitron borders conserved (thick black bar) and not conserved (thin black bar) between *Lp*-psGI.1 and *Lp*-psGI.2; non-helitron genomic sequence (thin grey bar). *Lp*GT sequences homologous (thick grey bar) and non-homologous (thick white bar) with *Lp*-psGI.2. *Sequence details*: alignments between *Lp*-psGI.2 and *Lp*GT sequences showing potential A↓T helitron insertion sites; these indicate possible ancestral 3' and 5' borders for different helitron insertion events and also mark the borders of *Lp*-psGI.1 and *Lp*-psGI.2 homology. *Lp*GT sequences: a) FLPB002289H22-b0FSP_20020409, b) FLPB002413G09-b0FSP_20011203, c) FLPB002264M19-g0RSP_20011109, d) FLPB002078I09-b0FSP_20010827, e) FLPB002029F17-g1RSP_20010827 (see Additional File 5).

BLAST comparisons of the *Lp*-psGI sequences against the *L. perenne GeneThresher^® ^(Lp*GT) library identified 10 individual *Lp*GT sequences with homology to *Lp*-psGI.1 both at the 5' and 3' ends, with the homology interrupted by a 7501 fragment inserted into a potential helitron 5'-A↓T-3' target motif (Fig. 2). The borders of the 7501 bp insert consisted of a 5'-TC and 3' 16 bp conserved hairpin and CTAG motifs, consistent with known helitron structures (Fig. 1 and 2). No evidence of a potential DNA replication-initiator-helicase protein coding sequence was identified within the 7501 bp fragment, indicating that it was likely to represent a non-autonomous helitron. No *Lp*GT sequences could be identified which spanned potential intact helitrons in *Lp*-psGI.2 or *Lp*-psGI.3, indicating that the 5' regions of these putative helitrons may have been displaced. However, 2 different *Lp*GT sequences were identified with homology beginning immediately beyond the conserved CTAG 3' helitron terminus of *Lp*-psGI.2. In both these *Lp*GT fragments the homologous regions began at a potential 5'-A↓T-3' helitron insertion site (Fig. 3). Three further *Lp*GT sequences were identified with partial homology to the same internal region of *Lp*-psGI.2. In each of these fragments, the homology ended at potential 5'-A↓T-3' helitron insertion sites (Fig. 3). This may represent the border of a smaller ancestral helitron, which subsequently expanded in the 5' direction.

### Gene fragments within the *Lp*-psGI helitron sequences

**Figure 4:**
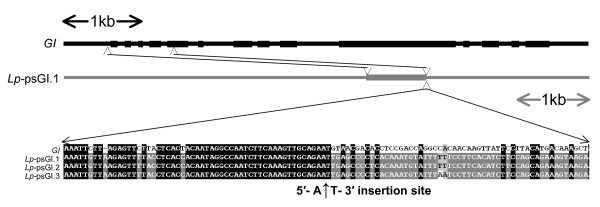
**Diagrammatic representation of region of *GIGANTEA *(*GI*) that has been ancestrally incorporated into a helitron**. Black horizontal bar = *L. perenne *genomic sequence spanning the complete *GI *coding sequences; predicted exons are indicated by the thick bar. Grey horizontal bar indicates putative complete helitron sequence from *Lp-*psGI.1; relative position of the *GI *fragment incorporated into the helitron is indicated by the thick grey bar. Sequence detail shows 3' border of conserved *GI *region with putative helitron A↓T insertion site at the border.

Within all the *Lp*-psGI sequences, the *Lp*GI-like fragment consisted of a continuous region of c. 0.9 kb from 35 bases 5' of the ATG initiation codon to 91 bases into the fifth exon (Fig. 4). Clustal alignments of the 3*Lp*-psGI sequences with *Lp*GI over the c.0.9 kb conserved region indicated different degrees of sequence conservation in exon- and intron-derived regions. Excluding base insertions and deletions, *Lp*GI showed 83–86% sequence conservation with the *Lp*-psGI sequences within the exonic regions but this dropped to 72–73% within the intronic regions. Within the 3 *Lp*-psGI sequences the ranges of sequence conservation within 'exonic' and 'intronic' regions were 94–98% and 95–97%, respectively (Table 2).

**Table 2:** Percentage sequence similarity comparing the *L. perenne *(*Lp*) and *F. pratensis *(*Fp*) pseudo-GIGANTEA (-psGI) regions and the equivalent region of *L. perenne GIGANTEA *over introns and exons.

	*Lp-*psGI.1		*Lp-*psGI.2		*Lp-*psGI.3		*Fp-*psGI.1		*Fp-*psGI.2	
	exons	introns	exons	introns	exons	introns	exons	introns	exons	introns
					
GI	83^1 ^(79)^2^	73 (67)	86 (81)	72 (65)	84 (81)	72 (61)	83 (73)	73 (68)	92 (89)	78 (70)
*Lp-*psGI.1	-	-	94 (90)	96 (90)	94 (91)	96 (86)	97 (86)	96 (94)	79 (75)	73 (64)
*Lp-*psGI.2	-	-	-	-	98 (94)	97 (89)	95 (83)	95 (90)	81 (75)	71 (61)
*Lp-*psGI.3	-	-	-	-	-	-	95 (84)	95 (86)	79 (75)	71 (57)
*Fp-*psGI.1	-	-	-	-	-	-	-	-	79 (68)	72 (64)

^1 ^Sequence similarity excluding base deletions and insertions (reflecting base substitutions).

^2 ^Sequence similarity based on alignments of the complete sequences

Additional gene fragments were identified 5' of the *GI *conserved region. A ribosomal protein S7 fragment was present approximately 1 kb upstream of *GI *in all of the *Lp*-psGI sequences while a succinate dehydrogenase (SDH) fragment was found close to the 5' end of the helitron in *Lp*-psGI.1 alone. Both of these fragments contained exon and intron sequences. A 0.8 kb insert specific to *Lp*-psGI.2 was found to contain a fragment of a non-LTR retroelement, including a partial reverse transcriptase reading frame, which most likely results from a retrotransposition event unrelated to helitron activity (e.g. TBLASTX match with AF474071.1, barley clone) (Fig. 1).

### Comparison of ps*GI *sequences from *L. perenne *and *F. pratensis*

Three different ps*GI*-type sequences (*Fp*-psGI.1–.3) were cloned from the *F. pratensis *BAC library on the basis of identification with primer pair GIGgt2F/2R. Comparison of these with the *Lp-*psGI sequences showed that one, *Fp-*psG1.1, represented a helitron remnant sequence which was highly similar to the *Lp-*psGI sequences, indicating a likely similar origin (the 6686 bp putative helitron region of *Fp*-psGI.1 showed 90% homology with *Lp*-psGI.1). *Fp*-psGI.1 contained a similar 3' terminus to the *Lp*-psGI sequences and the same SDH fragment near its 5' terminus (Fig. 1). However, *Fp*-GI.2 and .3 were noticeably different. *Fp*-psGI.2; they contained a *GI *fragment slightly longer than that found in the *Lp*-psGI sequences, extending more or less continuously from 231 bp 5' of the ATG initiation codon to 16 bp before the end of the 5^th ^exon, with subsequent partial homology up to the beginning of the 6^th ^exon (Additional File 3). The *GI *fragment in *Fp*-psGI.3 was similar to that in *Fp*-GI.2, except that it contained a 447 bp deletion covering the 3^rd ^and 4^th ^exons of the *GI *fragment. This truncated *GI *fragment corresponded to the smaller PCR product obtained in some of the DNA pools from the *F. pratensis *BAC library screened with GIGgt2f/2r. In total, *Fp*-psGI.2 and .3 shared sequence homology, interrupted by two major deletions in *Fp*-GI.3, over c. 5.1 kb region of *Fp*-GI.2 but showed no apparent homology with either *Fp*-psGI.1 or the *Lp*-psGI sequences outside of the GI region.

The conservations of exon- and intron-derived sequences within the *GI *fragment in *Fp*-GI.2 in comparison to *GI *were 92% and 78%, respectively, indicating slightly greater conservation of exon and intron sequence than was observed for the *Lp*-psGI sequences (83–86% and 72–73%); Table 1). The equivalent figures for *Fp*-psGI.2 in relation to the *Lp*-psGI sequences were 79–81% for exons and 71–73% for introns.

### Identification of additional conserved hairpin motif-like sequences in the *Lp*GT library

**Figure 5:**
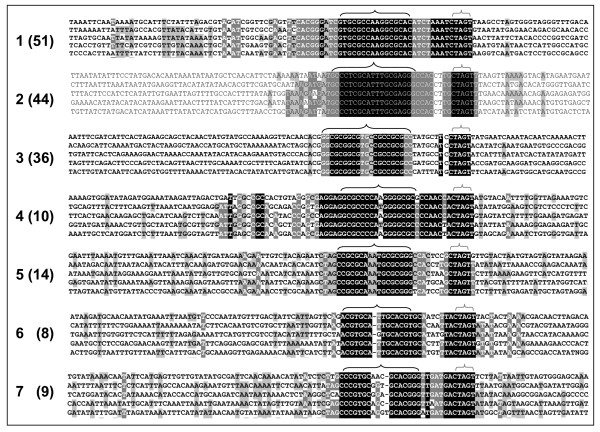
**Putative helitron hairpin and 3' border motifs identified in the *L. perenne *GeneThresher^® ^database with the SEEDTOP search**. Five examples of each of the 7 hairpin sequence types are illustrated; the total number of each type identified is given in brackets. Large horizontal brackets indicate hairpins, small horizontal brackets indicate CTRR↓T 3' helitron border. DNA base colour scheme relates to relative sequence conservation across all examples of each putative helitron hairpin and 3' border motif identified, not just the 5 examples of each type illustrated (see Additional File 3).

SEEDTOP searches of the *Lp*GT library identified 98 out of 16384 patterns with > 10 *LpGT *sequence alignments. Examination of these identified 7 possible helitron hairpin types (Fig. 5, Additional File 4). The most common type, represented 51 times in the *Lp*GT library (using the criterion of clearly non-homologous sequences, at least 40 bp of sequence present both 5' and 3' of the hairpin motif and no N scores), was the 5'-GTGCGCCAAGGCGCAC-3' 'Type 1' motif present in the *Lp*-psGI sequences. In addition to the 16 bp hairpin and the CTAG↓T terminal motifs, the 11 bases 5' of the hairpin and the 8 bases between the hairpin and the CTAG↓T were also strongly conserved. There was no apparent homology between any of the 51 sequences 3' of the CTAG↓T and only limited homology 5' of the hairpin which was probably due to the AT rich nature of this sequence. Between the different hairpin types, the length of the hairpin sequence varied from between predominantly 16 bp (types 1, 4–7) to predominantly 20 or 21 bp (types 2 and 3 respectively) with 1 to 4, but usually 2 non-complementary bases separating the 7–9 mer complementary sequence stretches. The hairpin was separated from the CTAG↓T motif by 7 to 9 bases for all hairpin types identified.

## Discussion

The discovery of the helitron families of transposons in plant species over the last few years has largely been a consequence of the availability of comprehensive genome sequence for the models rice and *Arabidopsis*, and latterly for maize. The significance of this has been demonstrated by recent analyses in maize, which have shown the potential of helitron transposition for generating haplotypic diversity and disrupting gene function [4,8,10-12]. There are still few reports of helitron-like transposons in the Pooideae grasses, a sub-family that includes the Triticeae cereals and the Poeae forage and amenity grasses, probably as a consequence of a necessary focus on transcriptome-based sequencing within these medium and large genome species. Consequently, the extent to which helitrons are present in, or may have had a role in modelling these genomes is at the moment unknown (though information for *B. distachyon *another Pooideae grass, should soon become available). Therefore, the identification of a putatively complete, non-autonomous helitron sequence as well as a number of partial helitron-like sequences in the species *L. perenne *and *F. pratensis *is important in confirming that helitron activity may have played a significant role in genome modelling within the Pooideae.

The complete non-autonomous helitron sequence, *Lp-*psGI.1, is not dissimilar to helitron-type transposons from other plant species, in that it has the expected 5'- TC and 3' hairpin and CTRR terminal motifs as well as showing apparent transposition into an AT target sequence (Figs. 1 and 2). Additionally, again as with similar helitron sequences, there is evidence that gene fragments have been captured within the helitron, in the present case fragments from a succinate dehydrogenase gene, a ribosomal protein gene and a fragment derived from the gene *GI *(Fig. 1). The partial helitron sequences *Lp-*psGI.2, *Lp-*psGI.3 and *Fp*-psGI.1 show a highly similar internal structure to *Lp-*psGI.1 towards the 3' end and so were, presumably, derived by transposition of the same ancestral helitron before the divergence of the *Lolium *and *Festuca *genomes; for *Lp*-psGI.2 and *Lp*-psGI.3, the fact that there is little homology between the 3 sequences beyond the 3' and 5' termini would indicate that they represent separate transposition events, as opposed to haplotypic variants. Whether *Lp-*psGI.2/.3 and *Fp*-psGI.1 represent partial sequences of complete helitrons or the complete sequences of helitron remnants has not yet been established.

There is no clear relationship between the helitron associated *GI *sequences (*Lp-*psGI.1/.2/.3 and *Fp*-psGI.1) and the two independent fragments (*Fp*-psGI.2 and .3). The latter are relatively more closely related to the intact *LpGI *gene, with the helitron fragments being significantly diverged both from *LpGI *and the available *Triticeae *sequences. *GI *is a single copy gene in rice and only a single *GI *copy exists in the current *Brachypodium *genome draft, but two divergent and unlinked *GI *loci have recently been described in maize [34]. The ryegrass and fescue *GI *fragments may therefore be remnants of similar ancestral duplications in the temperate grass genomes, whose intact descendants have been lost. It is surprising, however, that two apparently different *GI *lineages should both have become extinct leaving similar sized fragments preserved simultaneously in at least one genome (fescue), particularly if helitron activity was responsible for one fragmentation but not the other.

We considered whether capture by a helitron may have accelerated the divergence of the *Lp-*psGI.1/.2/.3 and *Fp*-psGI.1 lineage from a *Fp*-psGI.2/.3 fragment progenitor but this seems unlikely for at least two reasons. Firstly, comparing the divergence between the helitron *GI *sequences indicates that they have acquired a relatively large number of indels since their origin from a common ancestor, but that the number of point mutations is not remarkable (there are 7 indel differences between *Lp-*psGI.1 and *Fp*-psGI.1, for example, and only 2.7% sequence variation despite separation of the two host species by ~2.8 myr, compared with 6 indels and 13.5% sequence variation between the same region of *LpGI *and the gene from barley, whose last common ancestor was ~35 myr [35]. Secondly, divergence from *LpGI *is significantly higher in the intron sequences of the helitron *GI *fragments than in their exons, consistent with the expected selection for *GI *protein function. However, this contrasts dramatically with the large proportion of non-synonymous mutations, particularly generating frameshifts and stop codons, within the exons, indicating strong selection against this function. This suggests that the progenitor of the helitron *GI *sequences did indeed evolve gradually as an intact and functional *GI *gene, giving rise to a lineage distinct from *LpGI *and *Fp*-psGI.2/.3 but that at some stage its coding function became severely deleterious. This may have occurred before capture by the helitron or relatively soon after, as most inactivating mutations are shared by the elements described here.

The closer relationship between *Fp*-psGI.2/.3 and *LpGI *suggests that the independent *GI *fragments may derive from a more recent duplication which also suffered a subsequent extinction under selective pressure. Consistent with this, there is less divergence between *Fp*-psGI.2 and *Fp*-psGI.3 than between any two of the helitron GI fragments, while there is still a high level of non-synonymous differences from the *LpGI *and *Triticeae GI *sequences. An interesting question is whether the pre-existing helitron fragments could in some way have been responsible for the coincident fragment size of *Fp*-psGI.2 and *Fp*-psGI.3 or whether there is some inherent reason for *GI *to be disrupted in this way. In order to address this, we are currently investigating whether intact or recently fragmented GI genes related to either of the two extinct *Lolium/Festuca *lineages still exist in related species.

The observation that the common ancestral helitron from which *Lp-*psGI.1–.3 and *Fp*-psGI.1 were derived had captured *GI *and other gene fragments is of interest from two angles. Firstly, although these sequences are only fragments, replication and transposition following their capture has increased their copy number. Whether this had any direct consequence in terms of the perception and response to photoperiod is unknown, but the observation of apparently independent extinction of a subsequent *GI *duplication does suggest that the helitron capture and/or fragmentation may be beneficial to the host genome in helping to eliminate expression of unnecessary or deleterious duplicated genes, possibly in response to new selective pressures. A further question remains as to the positions of the *Lp*-psGI sequences within the *L. perenne *genome relative to each other and to *GI *itself, which maps to chromosome 3. To resolve this, attempts were made to identify allelic polymorphism across the 3' and 5' borders of the *Lp*-psGI.1–3 sequences in the mapping family, but amplified PCR products showed no sequence variation (data not shown) and, so, the *Lp*-GI sequences could not be assigned a genetic position.

The process(es) by which helitrons capture foreign sequences has yet to be clarified and either 'read-through' errors at the 3' terminus or a mechanism based upon non-homologous repair of double-stranded DNA breaks have been suggested [1,2]. Comparison of the *Lp-*psGI and *GI *sequences identified here provides some suggestion that the original capture of the *GI *fragment may have occurred by helitron expansion at the 5' end, a possibility referred to by [4]. Alignment of the *Lp-*psGI fragments with the equivalent *GI *gene sequence shows that the 3' border terminates with a potential A↓T helitron insertion site (Fig 4). It is therefore possible that helitron insertion originally occurred within this site in *GI *and upon subsequent transposition there was 'slippage' of the 5' helitron border resulting in incorporation of a fragment of *GI*. A similar mechanism is a possibility for the incorporation into *Lp-*psGI.2 of a sequence homologous to *Lp*GT fragments *a*, *b*, and *c*, as illustrated in Fig. 3.

There remains the major question as to how ubiquitous helitrons are in the *L. perenne *and other Pooideae- genomes – a question that will only be definitively answered by the accumulation of contiguous genomic sequence for these species. However, the *Lp*GT library does represent a collection of hypomethylated, presumed gene-rich [36,37] though relatively short (mean = 502 bp) genomic sequences. This size-range limitation means that they are unlikely to contain complete helitrons, but could contain recognisable helitron 3'-border motifs. Searches of the *Lp*GT library for short sequence stretches containing potential hairpins and the CTRR 3' helitron border motif identified 7 sets of sequences (Fig. 5 and Additional File 4). If these do represent true 3' helitron borders, this indicates that helitron activity in *L. perenne *may have been relatively widespread in recent evolutionary history, as evidenced by the presence of these sequences in presumed hypomethylated regions of the genome (i.e., their representation in the *LpGT *library) and by the sequence conservation across the hairpin types identified. The SEEDTOP search identified 172 non-homologous sequences containing potential 3' helitron termini. However, it should be born in mind that this is very limited survey of the *L. perenne *genome, identification relying on: a) representation within cloned, hypomethylated regions, b) the 3' helitron motifs conforming to the SEEDTOP search parameters (eg. 'perfect' complementary 7 mers) and c) > 10 copies of the same helitron type being present in the original search. Therefore, if these do represent real 3' helitron borders, the actual number of helitrons in the *L. perenne *genome may be considerable. This being the case, as comprehensive genome sequence becomes available for *L. perenne *and the various Pooideae species, it will be interesting to see the extent to which helitron activity may have been responsible for modifying and diversifying these grass and cereal genomes.

## Conclusion

An apparently complete non-autonomous helitron and a related series of incomplete helitron sequences have been identified in the Pooideae grasses *Lolium perenne *and *Festuca pratensis*. The identified helitrons had captured a number of gene fragments, including a fragment of the key flowering gene GIGANTEA. Searches of a *L. perenne *GeneThresher^® ^DNA sequence library identified a number of possible 3' helitron borders in unrelated sequences. This represents evidence for a possible ancestral role for helitrons in modelling the genomes of *Lolium *and related species.

## Methods

### Genomic libraries

The *L. perenne *(c. 5 × genome coverage) and *F. pratensis *BAC libraries (c. 2.5× genome coverage) have been described previously [38,39]. Derivation of copy number estimates from PCR screening of the BAC libraries is described in Additional File 1. The *L. perenne *GeneThresher^® ^(*Lp*GT) DNA sequence library database was obtained on license from ViaLactia Biosciences, Auckland, New Zealand and was described previously [40,41].

### Identification of *L. perenne GIGANTEA *and BAC sequencing

Primer pair GIG49660.6F (GTCCCGTCTATGATGCGTGA), GIG49660.7R (CCAGTTCTCATCACTGTTCTGG) was designed on the basis of conserved sequences in exons 2 and 4 of the rice *GI *gene (LOC_Os01g08700) and wheat and barley ESTs (GenBank: BJ245948 and BJ481891, respectively) and the identity of the PCR product confirmed by sequencing. This primer pair was then used to PCR screen the *L. perenne *BAC library to identify clones containing *GI *and *GI*-like sequences (Pseudo-*GIGANTEA*; *Lp*-psGI) which were sequenced directly from the BACs. Subsequently, both the *L. perenne *BAC library and the *F. pratensis *BAC library were screened with further primer sets based directly upon the derived *L. perenne *BAC sequences: primer pair GIGgt.2F(GCATCAAATGGGAAGTGGAT), GIGgt.2R (TGCAACTTTGAAGATTGGCC), anchored in the first and fifth exons of *GI *and which amplified c. 800 bp PCR products from both *GI *and ps*GI *containing BACS and primer pair GIGgt.1F (ATTCCTGCATCTGAAACCAC), GIGgt.1R (CAGCCAGCACATACGAGTC), which amplified c. 600 bp fragment from the 10^th ^exon of *GI *and identified just *GI *containing BACs. Thermal cycling profile for all primer pairs was as follows: 1 minute at 94°C, followed by 10 cycles of 1 min at 94°C, 1 min at 60°C (with the temperature reduced by 1°C per cycle), 1 min at 72°C, followed by 30 cycles of 1 min at 94°C, 1 min at 50°C, 1 min at 72°C

### Genetic mapping

The F2 *L. perenne *mapping population (n = 187) and framework map has been described previously [42]. *GI *was mapped as a segregating CAPS marker detected as a *Tat1 *(Fermentas, York, UK) restriction enzyme polymorphism in a PCR product amplified from the the 10^th ^exon of the *GI *gene using primer pair GIGgt1F/1R. The marker was placed on the existing genetic map using Joinmap v. 3.0 [43].

### DNA sequence alignments

*GI *and ps*GI *sequences derived from *L. perenne *and *F. pratensis *were aligned with other plant sequences in GenBank and with the local *Lp*GT library database using BLASTN. Further alignments and manual adjustments were performed using ClustalW [44] and Macaw version 2.0.5 [45]. Exon and intron sequence similarities between *GI *and the ps*GI *fragments inserted in the *Lp*/*Fp*-psGI sequences (Table 2) were calculated after ClustalW alignment and manual adjustment both directly on the complete sequence alignments and after exclusion of base insertions and deletions (*i.e*., reflecting base substitutions).

Potential helitron 3' hairpin and CTRR motifs were identified by searching the *Lp*GT library with SEEDTOP (part of the stand alone BLAST executables package [46]) for sequences of the form N^1^N^2^N^3^N^4^N^5^N^6^N^7^x(0,5)N_7_N_6_N_5_N_4_N_3_N_2_N_1_x(0,12)CT [GA] [GA]T, where N^superscript ^is a defined base and N_subscript _is its complement, x(n_1_, n_2_) is a number (n) of undefined bases between n_1 _and n_2 _(inclusive) and [GA] is either G or A. N^1^–N^7 ^consisted of all possible nucleotide 7 mers, giving 16384 search patterns. Where > 10 different *Lp*GT sequences were identified by an individual search pattern, the *Lp*GT database was additionally searched with the reverse complement of the search pattern and the sequences were examined for possible helitron 3' motifs using Macaw sequence alignments. Identical or near identical *Lp*GT sequences with different identifiers were only included once in the analysis. Possible helitron motifs were identified on the basis of sequence conservation across potential hairpin and CTRR motifs with low sequence homology 5' and 3' of these motifs. For illustration, c. 110 bp of sequence flanking the putative helitron motifs were aligned using ClustalW with manual adjustment in GenDoc (Figure 5, Additional File 4).

*LpGI *and all cited *Lp*-, *Fp*-psGI and *Lp*GT sequences cited are given in Additional File 5 along with their EMBL accession numbers

## List of Abbreviations

*Lp*GT: *L. perenne *GeneThresher^® ^genomic library; *LpGI*: *L. perenne GIGANTEA*; *FpGI*: *F. pratensis GIGANTEA*; *Lp*-psGI: *L. perenne genomic sequence containing GIGANTEA *pseudogene fragment; *Fp*-psGI: *F. pratensis genomic sequence containing GIGANTEA *pseudogene fragment

## Authors' contributions

IA and TL designed the study and analysed the data, all authors contributed to the execution of the study, IA, TL and KF contributed to the drafting of the manuscript and all authors read and approved the final version.

## Supplementary Material

Additional File 1 **PCR-screening of the *L. perenne *and *F. pratensis *BAC libraries and derived copy number estimates**. details the methods used and assumptions made in deriving sequence copy number estimates from PCR screening of the BAC libraries. References included. Click here for file

Additional File 2 **Alignments of predicted protein sequences for GIGANTEA**. Figure illustrating the alignments of GIGANTEA protein sequences from *L. perenne*, wheat, barley, rice and *Arabidopsis*. Click here for file

Additional File 3 **Alignments of partial *Lp *and *Fp*-psGI illustrating regions of sequence conservation with *LpGI *genomic and coding sequence**. Figure illustrating the regions of sequence conservation between *LpGI *genomic sequence and CDS and the *GI *fragments contained within the *Lp *and *Fp*-psGI sequences. Click here for file

Additional File 4 **Type 1 – 7 putative 3' helitron sequence motifs identified in the *L. perenne *GeneThresher^® ^library**. Figure illustrating all of the putative 3' helitron sequence motifs identified in the *L. perenne *GeneThresher^® ^library by the SEEDTOP search, including the sequences not illustrated in Figure 5 (main text). Click here for file

Additional file 5 ***L. perenne *and *F. pratensis GI*, ps*GI *and GeneThresher^® ^sequences**. FASTA formatted *L. perenne GI *and *L. perenne *and *F. pratensis *ps-*GI *and *L. perenne *GeneThresher^® ^library sequences referred to in the paper. Each sequence is accompanied by an EMBL accession numbers in brackets. GeneThresher^® ^library sequences are also described with their original library reference number. Click here for file
