# Supplementary material for: Fragments of the key flowering gene GIGANTEA are associated with helitron-type sequences in the Pooideae grass Lolium perenne
Source: BMC Plant Biol. 2009 Jun 7;9:70. doi: 10.1186/1471-2229-9-70 (PMC2702305; doi:10.1186/1471-2229-9-70)
Supplement: Additional File 1 — PCR-screening of the L. perenne and F. pratensis BAC libraries and derived copy number estimates. details the methods used and assumptions made in deriving sequence copy number estimates from PCR screening of the BAC libraries. References included. [file 1471-2229-9-70-S1.doc]

**Additional File 4**

**PCR-screening of the *L. perenne* and *F. pratensis* BAC libraries and derived copy number estimates.**

Construction of the *L. perenne* and *F. pratensis* BAC libraries and the PCR screening strategy are described in detail in Farrar et al., 2007, Farrar & Donnison, 2007 and Donnison et al., 2005.

BAC DNA preps from individual 96-well plates in the BAC libraries are systematically pooled in ‘3-dimensions’ so that a gene present as a single copy in the library can be detected in the PCR screen in 3 different BAC pools, one in each dimension. The location of the 3 different BAC pools in the 3-dimensions then identifies the original 96-well plate containing the target sequence.

In a 5 genome equivalent library (*e.g.,* the *L. perenne* library), a gene present as a single copy *per* genome will be detected in 3 x 5 = 15 individual PCR-positive BAC DNA pools. The five 96-well plates containing the target sequences can then be identified by systematic deconvolution of the 15 PCR-positive pool numbers into the 5 different ‘addresses’, each address consisting of 1 PCR-positive pool from each of the 3 dimensions. Therefore, an estimate of the copy number of a target sequence in the genome can be derived from:

(number of PCR-positive pools) /(3 x the genome equivalent coverage in the BAC library). *I.e.*, if a screen identifies 30 PCR-positive pools in a 5 genome equivalent BAC library, this gives an estimate of 30/(5x3) = 2 copies *per* genome.

However, this estimate must be treated very much as a guide, rather than a definitive number as:

1. the genome equivalent representation is itself an estimate and also an average of the gene representation in the BAC library over the whole genome.
2. the actual copy number of individual targets will be influenced by differences in cloning efficiencies and by chance variation.
3. false positive and false negative BAC DNA pools in the PCR screen will increase or decrease the copy-number estimate, respectively.
4. with higher copy number targets, there is an increasing chance that 2 (or more) target clones may be present in the same PCR-positive pool; this will lead to an underestimate of the number of target sequences in the genome.

From the above, copy number estimates of priming sites assayed on the *L. perenne* and *F. pratensis* BAC libraries can be derived as in the following table.

| Additional File 4 Table 1. Copy number estimates for priming sites in the *L. perenne* and *F. pratensis* BAC libraries. | | | | | |
| --- | --- | --- | --- | --- | --- |
| Library | Genome equivalents | Primer pair | PCR-positive BAC pools | Calculated copy- number | Copy-number estimate |
| *L. perenne* | 5 | GIGgt1F/1R | 26 | 1.7 | 1 - 2 |
| *F. pratensis* | 2.5 | GIGgt1F/1R | 3 | 0.4 | 1 |
| *L. perenne* | 5 | GIGgt2F/2R | 70 | 4.7 | 4 - 5 |
| *F. pratensis* | 2.5 | GIGgt2F/2R | 21 | 4.7 | 4 - 5 |

**Additional File 4 References**

**Donnison, I.S., O'Sullivan, D.M., Thomas, A., Canter, P., Moore, B., Armstead, I., Thomas, H., Edwards, K.J., and King, I.P.** (2005). Construction of a Festuca pratensis BAC library for map-based cloning in Festulolium substitution lines. Theoretical and Applied Genetics **110,** 846-851.

**Farrar, K., Asp, T., Lubberstedt, T., Xu, M.L., Thomas, A.M., Christiansen, C., Humphreys, M.O., and Donnison, I.S.** (2007). Construction of two Lolium perenne BAC libraries and identification of BACs containing candidate genes for disease resistance and forage quality. Molecular Breeding **19,** 15-23.

**Farrar, K. , and Donnison, I. S.** (2007) Construction and screening of BAC libraries made from Brachypodium genomic DNA. Nature Protocols **2**, 1661-1674.
