# Supplementary material for: Fragments of the key flowering gene GIGANTEA are associated with helitron-type sequences in the Pooideae grass Lolium perenne
Source: BMC Plant Biol. 2009 Jun 7;9:70. doi: 10.1186/1471-2229-9-70 (PMC2702305; doi:10.1186/1471-2229-9-70)
Supplement: Additional File 2 — Alignments of predicted protein sequences for GIGANTEA. Figure illustrating the alignments of GIGANTEA protein sequences from L. perenne, wheat, barley, rice and Arabidopsis. [file 1471-2229-9-70-S2.doc]

Additional File 1. Alignment of predicted protein sequences for GIGANTEA. LpGI, *L. perenne*, this study; Lp, *L. perenne* (ABF83898); Ta, *Triticum aestivum* (AAQ11738); Hv, *Hordeum vulgare* (AAW66946); Os, *Oryza sativa* (BAF04134 ); At, *Arabidopsis thaliana* (ABP96502).
