# Supplementary material for: Fragments of the key flowering gene GIGANTEA are associated with helitron-type sequences in the Pooideae grass Lolium perenne
Source: BMC Plant Biol. 2009 Jun 7;9:70. doi: 10.1186/1471-2229-9-70 (PMC2702305; doi:10.1186/1471-2229-9-70)
Supplement: Additional File 3 — Alignments of partial Lp and Fp-psGI illustrating regions of sequence conservation with LpGI genomic and coding sequence. Figure illustrating the regions of sequence conservation between LpGI genomic sequence and CDS and the GI fragments contained within the Lp and Fp-psGI sequences. [file 1471-2229-9-70-S3.doc]

Additional File 2 (2 pages)

Additional File 2. Alignments of partial *Lp* and *Fp*-psGI illustrating regions of sequence conservation with *LpGI* genomic and coding sequence (CDS). LpGI region illustrated (1416 bases) consists of 215 bases 5′ of the ATG initiation codon to 215 bases 3′ of the 5^th^ exon. Bases are colour-coded according to sequence conservation with *Lp*GI.
