# Supplementary material for: Fragments of the key flowering gene GIGANTEA are associated with helitron-type sequences in the Pooideae grass Lolium perenne
Source: BMC Plant Biol. 2009 Jun 7;9:70. doi: 10.1186/1471-2229-9-70 (PMC2702305; doi:10.1186/1471-2229-9-70)
Supplement: Additional File 4 — Type 1 – 7 putative 3' helitron sequence motifs identified in the L. perenne GeneThresher® library. Figure illustrating all of the putative 3' helitron sequence motifs identified in the L. perenne GeneThresher® library by the SEEDTOP search, including the sequences not illustrated in Figure 5 (main text). [file 1471-2229-9-70-S4.doc]

Additional File 3 (5 pages)

(Additional File 3 cont.)

(Additional File 3 cont.)

(Additional File 3 cont.)

(Additional File 3 cont.)

Additional File 3A-G. Putative 3′ helitron sequence motifs identified in the *L. perenne* GeneThresher® library. The conserved hairpin sequences and 3′ helitron borders associated CTRR↓T motif identified in the SEEDTOP search are indicated.
