# Supplementary material for: Fragments of the key flowering gene GIGANTEA are associated with helitron-type sequences in the Pooideae grass Lolium perenne
Source: BMC Plant Biol. 2009 Jun 7;9:70. doi: 10.1186/1471-2229-9-70 (PMC2702305; doi:10.1186/1471-2229-9-70)
Supplement: Additional file 5 — L. perenne and F. pratensis GI, psGI and GeneThresher® sequences. FASTA formatted L. perenne GI and L. perenne and F. pratensis ps-GI and L. perenne GeneThresher® library sequences referred to in the paper. Each sequence is accompanied by an EMBL accession numbers in brackets. GeneThresher® library sequences are also described with their original library reference number. [file 1471-2229-9-70-S5.doc]

**Additional File 5**

***L. perenne* and *F. pratensis* *GI*, ps*GI* and GeneThresher® sequences**

EMBL accession numbers are given in brackets after the sequence description

>*Lolium perenne* genomic sequence containing *GIGANTEA* (FN376855)

TACGATTTCGGCTATTGCCCGAGTAAGCGTGGATTCGAGCTGGATTTTCCCCACTTTTGCGGGCCATTTCGGCGTGCAGATATTCCTACCCTCGGTTCCGGAAGCAGTACGAGCAGACACGGTTATTGGAAAATCATTTGCGATGAAGATTGCTCTACGCTACCTTACAATTTCCCATTTCCGTACTTTACCTAATCAGTTTCCTTGGCCGCGTTATTCCGTTGTCCAAATCTAGTAAGCATGCGTGGCTTCTGGAAGAAATCAGTTCGTATCCTAACAGAGTATCGCGAGCAGAAACCTCAAAAGGAGCGGGTGAGCTCGTCGCATATCTGCGTTTTCCCGTTTCAGTATTTCTGATGATGTGCAGAACTAAAACTGCATCCTCCGTGCTTTGTCCTTTTTGTCGCTTTTTAGATGTTCCTTCTCTTGGTTCCGGAAGCAATAGGAGCAGAGACGGTTATTGGAAAATCATTTGTGATGAAGATTGTTCTATGCTAACTTGTAATTTCCCATTTCCGTACTTTACCTACTCAGTTTCCTTGGCCGCGCTATTTTGTTGTCCAAATCTAGTAAGCATACGTGGCTTCTGGAAGAAATCAATTCGCATCCTAACAGAATATTGCGAGCAGAAATCTCAAAAGGAGCGGGTGAACTCGTCGTATGTCTGCGTTCTGCCTTTTCAGTATTTCTGATGTGCAGAATTAGGACTCCACTCTCCCCCATGCTTTGTGTTTCGATGCTGCGGTTCAATTGCGGTAGTTACAGATTTGAAAGGGAGATTTTTTTTTTTTTGCATATGTCTAGTTGCTATAGTTAGCGTGGAGGGGCCTAACCGAATTTCCTAAACTATATGCGAGATAACGGTAATAGAAGCAAGAGAAACGCCGATATTGCTTTCTTAGGTAGCAGTCGTGTGACGAGCCTCTTTGATATATGGGGATGGTGTATCATTATGTGCTAATTTGTATGAACTTTTGAACAGCTACAGGGGAATACCAACCTTCCCTGACAAAATATGTCCTACGCTATGTTTTTTCTCTTATCTTTCTTCTGGTTTCTTCTTTTTATGGTATAACCTCAGGTTTTTATTTTGTTCTGATTTGTGGTATGCGCCGCCTATGGTGGCTGTGTTGCTTCTGTGCCGTGTCTGTTCTTCTAAGAGAAACTACATGTTTTGACTCGTCTTTTGAAAGAAGACACATTTTTTATTTTTTTATATGAGAATGGTTGAAACTTCTATATAGCATTTTTTTTTTAAATCCCCAGCTCACTGGCTCACTGGAGCTCGGGAACCAAAAATTTGCACTTCGACGAAACCCCCTTTGTGGGGGGTGATTGATGAAATCCAGTTTTTCTTTTTGGTTCCAAGACAAGTAGCTTATAAGAAGAATAGGACATGGAATAAATGGATATACTGAGGCAATTTCAGCTTGTTATTGAGTAGAGACGCTCTTATTGCAGAGCAGATTTTCCATTTTCTTACTTACTGAGAGTTTCCCTCTAGTTTCTTGAGCAGGATCTTAAACTAATAAGTATGTCTGTCTCAAATGGGAAGTGGATCGACGGGCTCCAGTTCTCTTCACTATTCTGGCCCCCGCCACACGATGCACAGCAGAAACAGGTACCATACTGATAGTTGAATTACGTTATGTATCCTGTAGCTTAGGAGACACCTGATGTTAATGAGACACTTCTTTCGTGTTCTCTGTTTGAAGGCACAAACTTTGGCCTACGTTGAGTACTTTGGTCAGTTTACATCTGACAGTGAGCAATTCCCGGAGGATGTAGCTCAGGTACTCTATCTCCTAGCACAGTTCACTACTTTTTGTGGTAATAAAATCTTTATCAAGTGTATTAACTTTTAGACCATTTTTCTTGCTTGTGTGCAGCTCATCCAAAGTTACTATCCATCGAAAGAAAAACGCTTGGTAGATGAAGTATTAGGTAATACATGAACTTCTGATAGCTATATGTTACACTACAGGCATTTGAAGAAAGAAACTAACACACCTTTTTCCATGTTACCAGCAACCTTTGTTCTCCATCACCCTGAGCATGGTCATGCAGTTGTACATCCAATTCTTTCACGCATCATAGATGGGTCCCTGAGTTATGATAGACATGGTTCCCCATTCAATTCTTTCATCTCTTTATTTACCCAAACTGCTGAGGTGAGGCCCTTACTTTCCTGCTTATCTTGAATTCTCTTGTTTGATGCTTGTTCAAGTGCAGTTGCTCATAGAGTGCATTTTCGGCTGCAGAAAGAGTATTCAGAGCAGTGGGCTTTGGCGTGTGGAGAAATTCTTAGAGTTCTTACTCACTACAATAGGCCAATCTTCAAAGTTGCAGAATGTAACGACACCTCCGACCAGGCCACAACAAGTTATTCCTTACATGACAAAGCTAATAGCTCTCCAGAAAATGAACCTGAACGAAAGCCATTGAGGCCATTATCTCCTTGGATCACAGACATTTTGTTAAATGCACCTTTGGGCATTAGAAGTGACTATTTTAGATGGTGAGTAATAATTTCCGTATTTTATTTCCTGCTGGCTAAAATTTCCTTTTACCTTTTTCTTATGGAATGTGACTATGATAGTCTTTGCTTTGTAATGTGAGCTGATTACTCTCTGCTTCGTTTCAAACACATCAGGTGTGGTGGAGTCATGGGAAAATACGCAGCTGGTGGAGAACTGAAGCCTCCAACAACTGGTTAGTAATTAAGCAGCCTATTTTTTTTTTTGATAACTAATCATATGCAGCTCCTAGTGAATAGTGCAGTGTTAGTCCTTTTGCTGTATTTACTTACTAAGGACTGGTTTGAAGTCATGCTACATTTGCATGGTCCTTTTTTCCAAGAGCACATGTTTACGTAGTTGAATTCTTAGACATGAATTGTTTGAAAATTAATTTGAATTCACATCCAGTCTGTTACTGTTATGTTGAAATTGCACATGGCTTAAAGATACGAGGGGTTTCCCAGAGTGAACACCTTTTGTTCTTGCATCTGATGCTAATTCTTATTATCAGCACAAATTTCTTATAATATTTTCCTTGAATAAGAAAATATCTGATAATATGTATTTGTGATGTAGCTTACAGCCGGGGAGCTGGTAAGCATCCACAACTTATGCCATCCACCCCTAGATGGGCTGTTGCCAATGGAGCTGGAGTCATCTTAAGTGTCTGTGACGAGGAAGTAGCTCGTTACGAGACAGCAAACTTAACCGCAGCAGCTGTTCCTGCGCTTCTGCTACCTCCACCGACAACGCCCTTGGATGAGCATTTGGTGGCAGGGTTGCCCCCTCTTGAACCATACGCTCGCTTGTTTCACAGGTATAGCTCCTATACAAAATTTATACCAAGTCTGCTTTCAAGATTATTCTAAACATGTCACTCTCCTGTCTAGATACTACGCAATTGCTACTCCAAGTGCTACACAAAGGTTGCTCTTTGGTCTTCTTGAAGCACCGCCTTCATGGGCTCCAGATGCACTTGATGCAGCAGTACAGCTTGTTGAACTCCTTCGAGCAGCCGAAGATTATGCTACTGGCATGCGGGTATAGCAACATTATGCACTCAGGCTGTTGTTTCAGTGTTATTAGGGTTATCATGATGCCCTACGACACAGAATTTCCATATCCACATGTAGGGTTGTGCAACTTTATTAACTTCAACTTCATTTTTCTGTTCAGCTTCCGAAAAATTGGCTGCATCTTCATTTCTTGCGTGCAATCGGAACTGCAATGTCTATGAGAGCTGGTATGGCTGCTGATACGGCCGCTGCCTTGCTATTTCGTATACTATCCCAACCAACGTTGCTTTTTCCTCCACTAAGACATGCCGAAGGAGTTGTGCAGCATGAACCACTAGGTGGCTATGTATCATCATACAAAAGACAGGTATGCAGTAGTTTTTGCGTCTTGCTAATTTTTATTTCAGGTGTTCCTTTTCCCTTAAGTTAATAACTGAAAGCACAGTTAGCTTTTTCTGGGGCCTACCCTGCGTTATGAAGTTCAGGACACCGTATGATACACTTATGTAGGCAGTGTTAGTTGCAAATCAAATGACTACATTACATGAGCTAAAACATTACAACAGAAAGATGATAACACATGCAGTTTAACTTCTATTTCTGAATAGAAAACTGCAACATTTGGTCATATTTGTACACTTGCTTGTATAGGATGTTTCTATCAATTCTTCATTGATTTTTCTTGTGATATACCGTGTTGGCTTGTTCTATGACTATCCTTATAGGATGATCATGAAATAAGTTAAGTATGATAAATACCTGGTAGAACAATCGCACGAAAGTTGCAAATTCCTCAATTGTCTTAATTACACCTTTACACGTGGGAATCCACATTTTAACTATAGCGTTAACTGATTTATGCCTTTTGTTTGCATGGCTAATTTACCTGCTTGCTCCTTGAATTATCCACAGCTGGAGATTCCTGCATCTGAAACCACTATTGATGCTACTGCACAAGGCATTGCTTCCTTGCTGTGCGCTCATGGTCCTGATGTTGAGTGGAGAATATGTACCATCTGGGAAGCTGCCTATGGTTTGTTACCTCTGAATTCATCAGCAGTCGATTTGCCTGAAATTGTTGTAGCTGCTCCGCTTCAGCCACCTACTTTATCATGGAGCCTATATTTGCCACTGTTGAAAGTATTTGAGTATCTACCTCGTGGAAGTCCATCTGAAGCATGCCTTATGAGAATATTTGTGGCAACTGTTGAAGCTATACTCAGGAGAACTTTCCCTTCGGAAACCGAACCATCCAAAAAACCAAGAAGTCCATCTAAGAGCCTTGCTGTTGCTGAACTCCGTACGATGATACATTCACTCTTTGTTGAATCATGTGCCTCAATGAACCTTGCTTCGCGGTTATTGTTTGTAGTATTGACTGTCTCAGTCAGTCATCAAGCTCTGCCGGGGGGCAGCAAAAGACCTACAGGCAGTGAGAACCATTCTTCTGAGGAGTCCACTGAGGACTCAAAATTAACCAATGGAAGAAACAGATGCAAGAAGAAACAAGGGCCTGTTGGTACCTTTGACTCGTATGTGCTGGCTGCTGTTTGTGCTTTATCTTGTGAGCTTCAGCTGTTCCCTATACTTTGCAAGAATGTTACGAAGACAAACATAAAAGACTCTATAAAGATTACCATGCCTGGAAAAACCAATGGGATCAGTAATGAGCTACACAATAGCGTTAACTCAGCGATTCTCCATACTCGTAGAATTCTTGGCATCCTGGAAGCTCTTTTCTCCTTGAAGCCATCATCAGTTGGTACCTCCTGGAGCTATAGTTCAAATGAGATAGTTGCAGCAGCAATGGTTGCTGCTCATGTTTCTGAGTTATTCCGTAGGTCGAGGCCATGCCTAAATGCACTATCTGCACTGAAGCGATGTAAGTGGGATGCTGAGATTTCTACCAGGGCATCATCGCTTTACCATCTGATCGACTTGCATGGTAAAACTGTGTCATCCATCGTGAACAAAGCTGAGCCTTTGGAAGCTCACCTGAACCTTACAGCAGTAAAGAAAGATGATCAACACCACATTGAGGAAAGCAATACCAGCTCATCGGATTATGGGAACTTGGAGAAGAAGAGTAAGAAAAATGGTTTTTCAAGACCACTCATGAAATGTGCAGAACAGGCTAGGAGAAATGGTAACGTTGCAAGTACATCGGGGAAAGCTACTGCAACTTTACAGGCGGAAGCATCTGATTTGGCAAACTTCCTTACCATGGACAGGAATGGGGGTTATGGAGGTTCTCAAACTCTCCTAAGAACTGTAATGTCAGAAAAGCAGGAACTATGCTTTTCTGTTGTCTCGTTGCTGTGGCATAAGCTTATTGCATCTCCCGAAACACAGATGTCTGCAGAGAGTACATCAGCTCATCAGGGTTGGAGAAAGGTATGATTACACATGCTACAGTCATAACACAGTAATCCTATGGAGATATTTTTATTTCTGGTAGCATTCTTATTAACTGTACATTTTCACATCTTTTAATGTATAGGTTGCAGATGCGCTTTGTGATGTTGTTTCAGCTTCACCGGCCAAGGCTTCAACTGCTATTGTCCTGCAGGTAAATATTTCTGCTGTATTCAAACATTTGGTCATAATAATTATTTTTTAATTTTTTTGCTTTGTTTCTGTTGTTCATTTCTCACGTTGGGAACAGACATTGGCTTCATTGCCTTCATGACATGCATCATATTTCCTGTCATATTTATGGGCTCCTGTATGCAGGCTGAGAAGGACTTGCAGCCCTGGATTGCTCGAGATGATGAGCAAGGTCAGAAGATGTGGAGAGTCAACCAGCGAATAGTGAAACTGATAGCTGAGCTTATGAGGAACCATGATAGCCCAGAAGCACTGATAATTCTTGCGAGCGCTTCAGACCTTCTGCTCCGTGCCACGGATGGGATGCTTGTTGATGGTGAAGCTTGTACCTTGCCTCAATTGGAGGTAACATCCATTCACAAACTTTCTTCAAGAGTGGAACATGTAACCTACAGTACTTGCTGTCAGAACAATATAGCTGTCATCAGAGCTACTTTTCATCCATTTTCTTGCTTTGAAAAGATGCATGTGCTGATGACATTCGTCCTATGGTTGAATATATACAGCTTCTGGAAGTAACCGCCAGAGCCATTCATCTCATCGTTGAATGGGGAGATCCAGGTGTAGCAGTTGCTGATGGCCTCTCGAATCTGCTGAAGGTATGTCGTCTCCTTGTAACTCACATCTCTTCACCAGAAACATAGCCACCTTTCAAAATGCTAACTCAATGTGATTTACAGTGCCGGCTATCACCTACCATCCGATGCCTTTCCCACCCTAGTGCACATGTACGGGCGCTCAGCATGTCCGTCCTTCGCGACATCTTGAACAGTGGACCAATAAGTTCCACCAAGATAATTCAAGGAGAGCAGCGGAACGGCATCCAAAGCCCAAGTTACCGGTGCGCGGCAGCAAGTATGACCAACTGGCAAGCGGACGTCGAGAGATGCATAGAGTGGGAAGCCCACAACCGTCAGGCCACCGGGATGACGCTTGCCTTTCTCACTGCAGCGGCTAACGAACTCGGATGCCCCCTTCCTTGCTGACACAGCCATATTTGAAGCTGACATCGGCGACACTTGACAGTTAGCGCGAGCAGTTGCTGCATGGTCAGCGAGCAGGATGGCTAATCCCTTGCTCAAGGATGACTTCCCAGTCTGCCCCCATTATGTGATTTAAAACTGATGTATATTAGTTGACCCAGTCATACGGAGCTTGCTCCCACTGTGTGATTTAACTTTTAATCTGACATTAGATGTTCAAGCATATTGAACTGCTTGTGCTGTAACTTGTATTTCTGTAGCCGAAAGATGTACACTATGGTAAATGAAGACATATCATTTTTCGTCATGTAAGATACATGCTTATCTGCAGAGCTTCCAACTAAATCTGCCTGTTAATAAGCATATGTTGCCTGGGCAGTTGTTTTTCTCGCGCCACAATGGTTAAAGGCCAGACTTTAATCTACAGTGCCAAGGCCGACCACTTCGTTGCACGATCATATCAACACAAAACAATCATGTCTAGAGTATACAGATCCAAAACCTGACAAAAGTTTCTGGTAACCCATACAAAACTTTATTGTGTATTTACAAGCTAGAGATGTTCAAACAACAAACTTTACAGAAAAAAAGAAT

>*Lp-*psGI.1 (FN376852)

ATCAAATCAACCTCCACCACTCAATTAAATTTTTCTAGTGCATAATCAACTAAAACTTGTGACAATTCAGTTGTAGAGAATATTAGCTTTTCCTTCTTGCTCTTGCGGATTGATACCCGACTTCCACATTGAGAGGTGCTACAGTGATCCCTGCTCTTGGGGGTTATCAGGCCCCTCTCTTCTTCCCCTATCTTTTCTCTTCTTATTCTTCCTCCTCCTATATCTCCCTCGCCAAATCCCAAATCGACGTTGGCCGGCTCCGGCTTCCCGCCCCAACCCCGATGTTCCCTTCCTCATCCCTCCCCCAAAGTCCAGGCCACTATCGCCCGCACTAGTAAGAAAGTTGTTATGGGCGACGAACTTGTCCAACAGCCCTGGTAGATGGTGACCGCATTTGGGGCTGGTTCTAGGGTAGTTCGCAAAACTTTTTTTTCTTGTTTTGGAGTCCCTCTACCACCACCGATTTCAAAAAAAAAAAAAAGTGGTAGATGGCCTATTTGTGCATGGTAAATTCGATTTTTTGATTAAACATGTGAAGTCAACCCCACTCAGCACCCCTAGGGTGTTTGATGATTGTATTTTTAGCATGTTTTTGCATTACAATTTTATCAAGTTATCATCAAGTAGTAGTAGGATTTTTGTATTTCACCGCGCTACCGCGCCCTTTTATGCTTGTCTACACAGGATTCTAAAATAACAAGTCAATAATGAAATCTAAAGGTATAATTGGTGCACCCCCATGGGGCTATTAGGTAAATCCAATTTTACCGGCTGTTAGATCTGTCCTCCAGTGAATCTCGGCCGTCTATTGATTTTCACCGTGTGGTATAAATTGGCCAGCCTGAGGTGAAAACCCTAACCGCCGCCTATCCCAGCGCAAGCTCCTTCCCAATCACCTCCACGAGCTCATCCTCTATCCCCTCGTGCATCCACTTCTCCCCCAATCCCCATCATTTCTTTGCTGCTGCTGTCAATCACGAGCCGCTGCTCAGCCCTCCGCTCCGCGACTTTTTCTCCTCACCGCCGCCAAGCCGTGCGAGGCGGCCAGACTCCGCCGTGCATCCTCCTTCGTCAATTTCCCCGGCGTTGGATTTGGTGGTCTTCCGTGGTGTCCTCGTCCCCTCCCCTGGCTGCGCTTGCTCCGCCTCGATGTGCCAGCGCTTGACCTCCACGGCTGCTCGTTTTCTTCAGGATTCGGTAGGAGGAGAGCTGGCCGCAGAGATGGGAGAGGTGGTCGAGCCATCGGGGGCCTTGTCTGCATCTTCTCCCTGGCCGCCGGATTCGGCCGCGAGGTACGTCATGCTGGCGCGAAGAATAGCCTCGGGGCACTTGTAGAAAGAGGGCTGAGACACTGCTCATGGTGTAATACTCACCAGTATGACGCTACTATATTGATCTGGTTTAGATGGCTTAATCACATCTGTTTCTTAGTTAATCTTCCGTATTTATTATTTTGTAAAAGTGCCAATCACATACCGGCACTTTTCCCTGCTGCATCCGAGATTTTTGGATTGGATTCGTGAAGATTGATTCTAGCTTTCCATAGTTTAATTCTACCGGTTAACTCATAATGATATTTCAGGAAACGGACAGAGAGTCATTTCCTTAATGTGGAAGATGGCATCGATCTTTCCATGCAACAAATACTCACGTCGGCCATTGGATTGGATCATCTGCTACTCAGCAACCTCAGGTCACACATGTGCTGGAGATGGTCAATACCATGGATGTGCATGCTGGTTACTTCTTCAAGTTAGTTGTTTATTCTAAATTGGATTTTAAAAGTAGTTCTTCTATCCGTTTGATTTATGCTTGCGTGATATCTGTATTGTAGGATCTCGAGTTCGTCCAGTTCCGTCCTACAGTCATATATGGTGCTGGATGCCTCATCACAGGAAGGTTCTATACATTCTACTATTTCATCATGTTGTTATATTAGGGAATCATTCTATACAACGTAAATCCCTGATTTGTGCTCCGACATTGTTCATTATAGTTGTTATCTTGAGAACAAGCCAATTATTGTGATGTTTTTTTTTGGATTGTTGGCTGTTGCTCTTCTCTTAATTGGCAAGTGTAAGCAGTGTGCATTTAGATTTTCATTATTTTCATTGTGTACGAGTGGTTGCAGCCTTTTTATTTTCTCCAGAAGATTTATGAAGGACGTACAGAAGATTTATATACAGAAATTTAACCTGATTTTTCCTCATTTTTAATTTGTAGTTTTACAACAGTTTGGCTCACAGATCAACTTCAGGTCCACATATCCTCACTGCTTTTTCCCCACTTCCACGCCTATAGCAGAGGAACCTAATAATGGAACTGAACTATGAAAAAAGTTTGAATTGTTTTTAAGAGAATAACTTTAAGTAGAAAAAAAACTGTTTTTTATGGAATGATGTTGGTAATTTATTTTCTAGATGTCATTGCAGCCAAAGAACCAAGTAGGTGATGGAGCTTGTGGAAAATGGATATAGGTACAAGAGCATGTGATAGAATATATGAATATCCTGGCCAGCTAGCTACGTTAGTTTTCTCCCTTTCCTGGCAAGTGGCAACGGACCACTTCCATAGGTATTATTCTTACATTGTTACATAGTCCTGCTATTTTAACTGTTTGGATTCGCTTCTACTATTTTTCATTTTGTTGCAATTGTAAGTATTCACATGTATATTTTCATCTTCTGGCTGCAAAAGCTGATGTACATGACGCTCTTGAAATTATTTCTGCGTGTGATATTTACTGATTTATCATGTTTCTCTTATAGCACCTTGGTTGGGTGGTGAGGTTGAGATGTCGCGCGGCAGAGTAGCCAACCAAGGCCTATTATTTTTGCAGTCCTATTGAGGTTCTTCTCTGAGTATTTCTTGTGTGAGCTTTCCTTTTTATCTGCATGTTTGGAAACTTGTAGGCCAGATTAAGAGCACTTATATTTTGTCTAAAAGTTTTGCCATCTTTTTTGTCTAAAAGTTTTGTAACAATTGATATCTTTTAGCTTGTAAAAAAATTCCCTCTAATTGTTTCTTGCTTCATGTTGATAGGTCTGCCGACAGGTTCAGACTTTCCAGTCAAGGAATTTATGCATTTTCTTCATGCAACATACATCCTGTGGTAAGTCTTTGAGCATGTAACTTCCTAATGGTGTCATCAAAGGCTTGATGGTGAAGATATGATGCTAATGAGATGAATATTGGTTTGTTGATCACGTGAATAGAAATTTTCACCTAAGAATAGCTTTCAAAAACTGAGGTCTTGGGCTTTCCGTTTTACCCTTCTCTGTTCGACTCTCGAGCGTAGAGATAACTGTGATATGCATATTTTCACATAAGAATAGCTTTGGGGCATTATGTACCAGAAAAGCATGCTCCCAGGTTAATGTATTTTTCCTACTTTCTATAAGCATTTTGGTATGTGGGAATAGAATACCGTTTAATTCTTCATTTCTTCGGGCTTTCATAACACATAATTACCATTCCCGTATCATTACCTGGCATTGTCATTTATATGCGCAAATTGGGTGTTCCATCCTACTATATGTACCTTCTATTGCATTTGGAGCTAAAAATCTTAGATTGATTTGCTATGATGGGATATAAGGAAATAGCCTTCCACGCATTTACTTATGTAGCATTTTACCTCGTTGCTTTCTACCGAAGCTTATGTTTTTCTATTTCGTAAATTGGCTAAGAAGCAATAAAAAAACGTTAGTCTGTAGGGTCTAATTTTTGTATAACACATATTGCTTTTTTCCACAACTCCTCTGTCTCGCAGGTGAGGGGTTCTCCCCCATCTCCCGTGTTTCCCCTTTGCAATTTCCACTTTGTTTTAAATAGCCGGCTATAGCTAGGCTATAGCAGGGCTATAGCCTTTCCCGCATGATGCGGCTAAATGCTATAGCCCGGCTAAAGGTACCAAAGTTCTTAAATAGCCGGCTATAGCCTGGCTATAGCCGGGCTATAGCTGTTTTGGGAGGCCGTGGCTATAGCCTATAGCTAGCTATTTAAAACCATGCCACAACTCCTCTTCCGTGTTCGGATGGGGTTAGGTGTAACACACTCATATCTTGCAAGCTTTCTTCGATCTAGAGAATGGCAACCAGGAGCTCAAGAGCGACCTAAGGACCTCTACATCAACACCGCGATGTAAGCTGATGTCTGCTACATCCTTCTAGATCTTCCGTGTCTGCCTCTTTGTTTGGCTGTTGGTGGAGCGTAATGGTGTGGTGGTGTTTTCTTTGCAGCCAGTTGGATATCGTCGGGAACAGGAAGGCCGTGGAACCACGTGCCATGTCGCCTACACCTAACATGCTAGGCTCGTCAGGGAGCTCGAGAAGAAGTACAGCGGCAAGGTGAGACACTTGCATGTCCTCTTGTGCTTTGAGATAGCACTTTCTTGTATCTTATGCTGCTGTGGCAGCGTTGAGTTATTGTGATTAGATGATGCTTGCTGGGTACAGTTTAAGAATCTATTGTTGCCATGTTAGGAAGTGAATTGAGTTTCTAGCACTTTCCACAAACCCAGAACATGATATGCCACGGTTTGGTTTCTAGCACTTTTCCCCAGGCCAGAACATTATATGTGCCATGATTAGTGTATGAAGCTGCTTGTCTATACTAACAAATAGATTTAACAGGGAATCTACACGTCTCAGATGCTATTCCTTTCCTAACATTGAAATGATAGCTAAATTGCACTATATGTTATCATTTTGGTATATATAATAAGGTAATTGCAGCTATCAATCCTACAAGTCTTGCCTTTATATGTTATGTTGTGTCCAAACTTGATTTCTGCTATTTTGTGCTTTAATCTTCTGAAAATTGTTGATTTGTAATGTAAATTCATGCGCTTTGTCCCAGATCATCCTCTGTATTAAATGCACCAGTTCATTCAATTTAACACTTGGTTGTTTTGGTCTTGAGACTTTAATTGAGCAGAAAGCTCTTGGTGGTGGCTTTGGGGGAATATTTACCATCTTCATTCATCTAAACAGTTAAGCTCTTGTGCTCTCTAAATTTTGTCAATTAGTATCTGACATATCTTTATGATGGTCATATGCAGTAGTATACGTATGTAACACCTCTATCGTTTATTGAGGAGGTTCTTAGACCACTAAATATGTCAGCATCAAGTGGGAAGTGGATCGATTGGATCCAGTTCTCATCTCTGTTCTGCCCTGGCAATGATGTGCAACAGAAACAGAACAGGTACCGTACTGATAATTGAATTACGCTAGATACGCTGCATATTTGAAATCACCTGATGTTAATGAGCCACTTCTATTTTTTTTCTGTTTGAAGGCACAAACTTTGGTCTATGTTGAGTGCTTTGGTCAGCTTACGTACGTGTGACAGCGAGCAATTTCAGAAGGATGTAGCTCAGGTATCGTATCTCTTAGCATAATTTAGTATTTTCTATGATAAAGTCTATATCAATCTGTTGACTTTCTGACCCTTCACTTTTGCTTGTGTGCAGCTCATTTAGAGTTGATATCCATCAAACGAAAAACGTTTGGTAGCTGAAGTATCAAGTAAAATATGACTTCTGGTAAGTGGTAACTACATGTTATACTATAGGCATTTCAAGAAAAGAAAAATTGACACACTTTTTTGAATAGTGTGCAACAACTTTTATTCTTCCTCACCCCAGAATGACCATGTTGTTGTACTCCAATTCATTCACGCGTCATAGATGGGATACACTGAGTTCTGGTACGTATGGTTCCCCATTCAATTCCTTCCTTTTTTTATTTACCCAAACTTCTGAGGTGAAGCCTTTAAGTACTGGTTACTTTTGGTTCTCTCGCTTGATGTTGGTTGAAGTGCTGTTGCTCATAGAGTGTCCTTTTGGCTGTAGAAGAAGTACTCACAGAAGTAAGCCTTGCCCTGTTGAGAAATTGTTAAGAGTTTTACCTCACCACAATAGGCCAATCTTCAAAGTTGCAGAATTGAGCCCCTCACAAATGTATTTTTTCCTTCACATCTTCCAGCAGAAAGTAAGAAATATGTAGTTATTTTGTATGTCTATATTTTAACGTGGTTAGAGATTAAACCGATGCTCTTCTTGCTGATGGTATGCAACTTAATATGTACGTTATATAATTGTGGTCCATTGCAGATCAAAGTTTTTGTTGGACACATGCATCGGTGCTTTATCTTGATTTGATCTCATTTTTTTCAGCGATTTTCATTTTAGTTGATATTTTTAAGATCAGTTATTTAAAAACTTGGGTTGCCTTATTGGTTTGACATTACTATTGCATCTTTCGTTATCATGACCCAGATGAATTATATTACTTCAATGACTGGGTGGTGTGGATAATAGATCTCTGTTTATTTTCTTTGAAAGTACTAGAATTTTTGTAAATATTTTACCATGTCTAGGTACAATATCAGATAGACTACTATCGCTTTTTTAATGAAAGGATTAAATCCCAAATACAAAGCTGGAACTTATTGGTTTGTAGCTTTTCAGTTTTGTGCAACTTATTCTTGCTTTTGGGTTTTGTGAAACTGATTGGTGCGTAGCTTCAGGATTAAATCCCAAATATTTTACCACTATTGGTCTGCTGATAACAATGAAAATATACTAGGAGCATTGCTTTTTGGTTTTCCTATTCTTGATTTTGGGCAACTCTTTAGTTCTTTCAGGTTTTTCAGGCCTGCTCATGTTCGGTTTGTGCCTTCCTCTTTTTTGGATTTCCTATTAGTTGTCTTCCTTTTTCCCCTCCTTTTTTGTCTTCCTGTTCTCGATTTGGAAAACCTATTTAATTCTTTTGGGGTTCTTGCTTCTGGGTTTTTTAGAGGGTTTACTCTTCGTTTTGTTACTCCTCAATTTTTCTTTATTTAAAGTTTTCCCTTCTGTTTATTATTCACCTATATGTTCCTTTTTTTGGCACTTCTTTTGGTTTGTTTCTGGCTGCTGGTTTTTTTGTTCTTTGGCCTTTTCTATGCTCCTTTGTCTTCATTTACCTTTTGGTATGTGTTTTGTGTTTGTTCGCATTGTCCTCGTCTGTGCATCCGTGAGTTATCGTGTGTGTTCGATGCCCGGTGCATATTTTTATCTCTTCTTAGTACCACACCTCCATCGAGCGGCGAGGTGGAGCTGACGCAAGTAGATACCAACATACTTTTTTTTTGTCTAAATAATAATGAAAATCCCAGCATGTATGTGTATCATAATACTATAGCAGAGCTCGCGCTTTGAAATTGTTTTGTCTAAGAAAAACACAGGCATCCCATGATCTGTGTTGTAGAAAAGTTCTTCTCTCTTTTGTCTAAACGTTATCATTGTCTCATTATTCTGAAGTACAGATGTTCTTCTCTGTTTGTCGGCGTTCAATTATATCTTATGTACCCTCTATATTGTCATTGATTTGTCGTTCAGGAGACCCGTCTTGGGCTCTGCTCCGAGATCGCCTCCGCAATGCTGAGCCACGACCGTAGGCTTGCAGTGGTGGGGTTCGTTGTGGTCGGTCTGACCCTGGCAAGGTGTCAACACTGACGATCTCGACATCTCTGTACAAGCCTGAGGTAAACGAGGCATCCCTGCTCTCAGGCTGGCCTACAACCTCACGTGATGTAGGGTCTGCTCCTCTCAAAGGTGTCATGTTATTTCATGAAATAATTTGTCTTGGGCTAATGGTTCACTTCAGTTTTGATCTCTTTCTATTTCGTAGAATATCTTAGGCTAACGCCTTGGTGGAGACTGATGTAGCCTGCTATGTGGATTCTGAAATAGATTTATACATGAATGCTAAAGCTCCATGGTGAATCATGGTTTTGTCTTTGATTACTGCTTCCAATCATGGTGTAGCTGAAGTAAGAAGTTGATTGTGCAGTAGCGCGAGTTGAGGAACCAAGTGAAAACTTTTATTATAAAACCAATAATATTAAATGAGGTATATTTGTTACAAACTAGCATCCGTATCCATCCGGTGTAACAATCAAAGAGAGGTATTTGTTGTTTTTTATTAAGTTCATACCTTCACGTGAATATACATACCACCGAATGTAGCTGCTCCATCTGATTGTATATTGTGCTATTTACCATGTCATTTATAATAGGGTGTTATTGTAATATATTTTCCTGTTTTCCTGACTGTAAGAGTTTCCTACTTTGTTCGTGATTTTGTTTACTTTAATACGGAGTACAAAATGTTAATCGATGTGAATTTCACGGGGTCGTGCGCCAAGGCGCACATCTAAATCTAGTATCAATGAAAACCGCCATTTTCTGGCATTTTGACGTGATAGAAATTATTTTTGCACTTAATAATCCGCCTGGGCGAAAGGACAATGTGGGGACAGCTTCCAGAGACTTTAACGGGCACCCGTACGGGCAGAGCCCGCCCGTACCACCCGCCAGAGGCACCACCTCGTCAAATACTTTCACCCCCCCCCCCCCCCCCCCCCGCAGACGAATCCGAGATCGGAGTTGAGACGCGACCAGAAACACCAGAACACTGCTCCAGGAGAGATCTGAGGGGAGATCAAGAAGGGGCCTCATCCACGGTGCTACCCGGATCATTGGAGGACAAGGCATCATCACCTTCATCATCATCCACAACGAATCACCATCTCCATTCTCTCCATCCCATTACATTGTAATCTCCATTACTATTGTGGGTTCATACTTGGATTCATACCGCTATCCCTTTTCCATACACAAGTTTATGATGATGTTCTTGATTGTCTCCATGTGTGAGTAGTTCATCCCGTTCTTGGGGAGATGGAGAAACCCTAGTTGTACTATTGTATGAATTAAGATGATTTATATCTTTGCACTATGATTGTATGTTAGTTTTCTCTTGGGATGTCGACCATGTAATATTTGCCTAAGGGTCACATGAGGGAACTACCATCGGAAGTTTGGTAGTGTATACGGCGGGAAGTGACATCCCGTGTTCGGTGCACTATTGGATGGAAGAGGGGGATAAGAAGGGACTCGCAAGCTTAACAAGTAACGATGGGGGAATCACTTAATTGTGATGGATCAATAGGTGGCTTGCCGAAGGTCGTTGCAGTGGTTACTCAGGGATATGTTATGAGGTACATAATCCAATTCGAGCTCTTCCCACATACAATGAGAGCTAAGATATGAGTTATCTAGTTTGTGTCTTTAGTTAATACTAGCGGTGGAACCAACAACCCCTGAGAACATTTTAGCCTTATTGCAGTTCAAACTTGACTTTTAGTTGCATTTACATTCTTCAGTTTAAGTTATTTACTCTACAAAAACACATTACACAACCTTTTCTACTTTAATCCTTCGCTTCAAATTTGAGACAACTTGCGGTAATTTTAGATAAATAGTAACAAGGGGTATTAAGATCACTACTAGTAGCTCATCGTGGTTCGATACTCTTATTTCGAAACTAGCTACAACTGATATGTGATCTTGCAGTCATCAAGCTATTTTCGGGCGCCGTTGCCGGGGAGCTCAGCGCTTTTGG

>*Lp*-psGI.2 (FN376853)

CATATCTCCTTCAGTCTCCCACTTGCACTAGAGTCAATAATCTAGTTTACATTTGTAAAGATATAACACCTTGGTCTTCTGGTGCTTTATCATGTATTGCTCACAGGAGAGGTTTTAGTCTACGGATCTGACATGCTCAGAAGCGTATGTATTTTGTAATTCATTTGCGTCTCAACGTATCACTCATTTCCAAATGAGTCGGCATTAAATATGTTTGGTCTTCTGGTGGAACCTTAATTCCGCGGTCTGAAATATGTCACTAATATTGTCACACACAATATAGCTTCAAAGTTCTGACTCTGTCGGAACTACACCAAGTTCTCAAAGAACCTCTTGACTTAACATCCTTTGTTATTGTCAAAACAATGACATACTCTGCCTTCTTTTGTAGAATCCGTCACAATATTTAGAACTTTTCTAAATCTAGCATAGACAACTTCTAGCTCATTGTGCTACCTTTTAAACAACACTTAGTCTAATTTGAGATTGAAATTATATTTTATATGTGACAAAACCAATATCGGTGTAACACCTTACAGCGATTTGTTTGTCATTTCTTCATACAAATATATATATATATATATATCCTTAGTTCTTCTAAAGTACTCAAGGATATTCTTACTGTCGTCCAATGATCATCATATGAATCATTCTGGTATATGCTCATAACACTTTATAGCACATGATATCTGATTGTGTACATATTATTCGTGATCTATAATCACTCATGTGTTTTTACTCATTGAGTGTCAGATACACTTAAGTCTTGTTAAAACTTCACATGACAAGAACATCTTCTTAATATTTCTATATTGAACTATTTCAATATCCATTCTATGTACTTTGACTTAAACTTATTTTTGTTTCAATCTATCTTCATAGATCTTGACACTAAATTTGTTTCAGTCCATATCTTTTCATTGAAGTTAATTTCTCAATGAAACCTTTTTAATCAAGTATATAATTACATTATTTATAACCAACTATATGTCACCTACATAAAGTATTATAAATATGTCTTAGCGCTCCCACTTAATTTCTTGCAAATGCAAGCCTCTTCATCGTCTCTGATGAAATCAAAAACTCTTTGACTATTTCATCTGGTGAAGATTCAAACTCCGCGATACTCACTTCATCAAATTGAAGTTTGTATACCTATCTAATATTCCACGGACCAGCAAAACTCTTGGTTGTATCTTGTATACACCTTTAATACATACTTCTGATAAGTAATGTATTTTGCCATCCTACTAGAATAATCCATATAGACTATAAGCATTTCTACGGAAGATCTAATCTTATCGTAGTCAACTCTTTGAACTTTGTCGTAAACAATTTTTCGACAAGTCGAGCTTCTTCAAGGATATTTCATCCAAGTCTATAGATCCATTTACTTTCAAAAAGTATTCATCTATTATGGATTTCATGGCGCATGGCCATTTTAACGGAGTCAGGGCCCATCATAACTTCTCTGTTTGTAGTTGGTTTATCATTGTTCAAAATCAATCCTTTGTCCACAAATCATTTATTTAATCACAAAGTAAACCATACCTACAAGGTTCAATATGTACTTCGATCTCCATGACTAAAACACTTTGTAGTAATGGGAGCCATGATCGTTGTGGCCGCTTCCGAAACCAATTCCGATGCTGCGCTACTCTGATCATTATGCTCAGGTTCATGAACCTTATCAAATTATATTGTCCTCCCACTCAAATACTTCACTAGAAACATTTTCTCGGAAATAAGAAACATTGACAAACACTTTTGTCTTTGTCTCGTGGGAAGAATTTCCAATCAAATCTCTGGGATAACCAACAAAGACATTCATCCGATTTTGGTTGTAAACTTATTTACTTATGCTATGCATACCAAAATTTAAGAAAAAGACTATCAGGTTTCTTACCTATGCCATAACTTGTATGGTGTCATTTCAACGGATCATGATGATGCTCTATTTAGTGTAAAAGCGGTAGTCACTAAAGCATAATCCACAAAAATATAATGGCGTCATAATATTTTTATCTCATCATTAATCCAACAAGGTTTGGATACATATCTTGGATACTATATCATCATTATGATACTCCAAGAAATGTGAGTTGTTGAACAATTTCATAACTCTCTTAGATGTTCGCTAAAACTCGTAATTCAAATATTTCCACCATGATCCAATCATAGATATTTGACTTTTCTATTACGATGATTTCCACTTCATGCTGAAATTTATTTGAATCCATTCAAATGTTTCAAACTTCTTCCTTATTGAATATATCCACATATATATATATTCAATTCATTGTTGAAAGTTTTCATGAAGTAGAAGAATCTCCCGCACACAACTATGCCCAGTGAACCACATACATCCTCATGTATTTTTCCACTAAGTTAGTTGCCCGTTCAACTTTTGGCCTATGAACGGTATTTATATCATTCCCTTTAGAAAAGATTTGCAAGCGCCAAACGATTCAAAAATCAAATGACTCCAAAAATCCATTTGCATGGAGTTCCTTCATGCGTTCCTTTCTAACATGACCTAAATGGCGGTTCCACAAATAAGTGGAATTCAAATCATTTGCCTTATGACATTTTAACGTCAGTGTTATGTATGTGTGTTTCACCATTAAGATTTATAATAACTTATCCATCGTACATGGAGTAATGTCATAATTTGAACAACTCATTGTTTTCATTTGACCAGAGCAAAATAACAATTATTAAGTTCTTTATTATAAATTCTAAGGGCTAGATAGAATGCCAACGATGAACATAATAATACTTTATTTTTGTTCCAGACGTGCATTCTTATCATGTTTAACATGTTTTTTCCTCATTTTTAATTTGTAGTTTTACAACAGTTTGGCTCACAGATCAACTTCAGGTCCACATATCCTCACTGCTTTTTCCCCACTTCCACGCCTATAGCAGAGGAACCTAATAATGGAACTGAACTATGAAAAAAGTTTGAATTATTGTTAAGAGAATAAATTTTAAGTAGAAAAAAACTGTTTTTTATGGAATGATGATGGTAATTTATTTTCCAGATGTCATTGCAGCCAAAGAACCAAGTAGGTGATGGAGCTTGTGGAAAATGGATATATGTACAAGAACATGTGATAGAATATATGAATATCCTGGCCAGCTAGCTACGTTAGTTTTCTCCCTTTCCTGGCAAGTGGCAACAGACCAGTTCCATAGGTGTTATTCTTACATTGTTACATAGTCCTGCTATTTTAACTGTTTGGATTTGCTTCTACTATTTTTCATTTTGTTGCAATTGTAAGTATTCACATGTATATTTTCATCTTCTGGCTGCAAAAGCTGATGTACATGACGCTCTTGAAATTATTTCAATGTTTGATATTTACTGATTTATCATGTTTCTCTTATAGCACCTTCGTTGGGTGGTGAGGTTGAGATGTCGCGCGGCAGAGTAGCCAACCAAGGCCTATTATTTTTGCAGTCCTATTGAGGTTCTTCTCTGAGTATTTCTTGCGTGAGCTTTCCTTTTTATCTGCATGTTTGGAAACTTGTAGGCCAGATTAAGAGCACTTATATTTTGTCTAAAAGTTTTGCCATCTTTTTTGTCTAAAAGTTTGGTAACAATTCATATCTTTTAGCTTGTAATTTTTTTCCCTCTAATTGTTTCTTGCTTCATGTTGATAGGTCTGGCGACAGGTTCAGACTTTCCAGTCAAGGAATTTATGCATTTCTTCATGCAACATACATCCTGTGGTATGTCTTTGATCGTGTAACTTCCTAATGGTGTCATCAAAGGCTTGATGGTGAAGATATGATGCTAATGAGATGAATATTGGTTTGTTGATCACGTGAATAGAAATTTTCACCTAAGAATAGCTTTCGAAAACTGAGGTCTTAGGCTTTCCATTTTACTGTACCCATCCTTCTCTGTTCGACTCTCGAGCGTAGAGATAACTGTGATATGCATATTTTCACATAAGAATAGCTTTGGGGCATTATGTACTAGAAAAGCATGCTCCAAGGTTAATGTATTCTTCCTACTTTCTATAAGCATTTTGGTATGTGGGAATAGAACACTGTTTAATTCTTCATTGCTTTGGGTTTCATAAACACGTAATTACCATTCACATATCATTTCCTGGCATTGTCATTTATATGCGCAAATTGACGCATGGGTCTGATGTGGTTCGCTGGAATCTAAAAGAGAGTGGTAAATTCTCTGTTGATTCTATGTATAAAGCGTTGATTCAATCCGATACGCCAGTTGTAAATAATAAAAAAATCTGGTCCATGAAGATTCCATTAAAGACGAAGGTCTTTACCTGGTATCTTCGTAGGGGGGTTATCCTTACTAAGGACAATCTTGTGAAACGTAGTTGGCAAGGTAGTAAAAGGTGTGTTTTTTGTCTTCACGATGAGACTATCAATCACCTTTTTTTTCAATGTCAATTTGCGAGATCTATATGGTCAATAACCCAAATAGGTTCTACCTTGTACCCACCTCGGAGCGTTGCGCATCTCTTTGGCAGCTGGCTCAATGGGGTGGAGCATAGGTTCAAAACTTTTATTCGGGTGGGGGCGATTGCCATTATTTGGTCGCTATGACTATGTAGAAATGACAAGATTTTCCATGATATTAACTCATCTTGTTTGCAGGTTATATACCGGTCCACCGTTTTGCTCCGTTCGTGGTCTTTGCTACAACGAGTAGAGAGTCGCGACCTCTTTATGGAGGTATCTACACGATTGGAGGATGTGGCGAAGGATATTTTTTCCCAACATGGTTGGCGGCGTAATCTACGGATAGAGCCTCCCGCGCCATAGGAGGGTGTTCTTTTTTTCTTGTTTTATTCAGTTCTTGTAACAGAGTGGTACTTGTGTGTGTGCGGCTGTGTGCATCGTGTTATGCAGAGGCCGGGTGTAATACTTTTCTAAAGTAATGAAAGCGCCCTTTATCGAAAATATGCGCAAATTGGGTGTTCCATCCTACTATATGTACCTTCTATTGCATTTGGAGCTAAAAATCTTAGATTGATTTTCTATGATGGGATATAAGGAAATAGCCTTCCACGCATTTACTTATGTATCATTTTACCTCGTCGCTTTCTACCTAAGCTTATGTTTTTCTATTTCGTAAATTGGCTAAGAAGCAATAAAAAAAAGTTAGTTTGTAGGGCATAATTTTTGTATATAACACATATTGCTTTTTTCCACAACTCATATGTCTTGCAGGTGACGGGCTCTCCCCCATTTCCCGTGTTTCCCCTCTGAAATTTCCCTATTTCCACAACTCCTCTTCCGCGTTCGGATGGGGTTAGGTGTAACACACTCATATCTTGCAAGCTTTCTTCGATCTGGAGAATGGCAACCAGGAGCTCAAGAGCGACCTAAGGACCTCTACATCAACACCGCGATGTAAGCTGATGTCTGCTACATCCCTCTAGATCTTCCGTGCCTGCCTCTTTGTTTGGCTGTTGGTGAAGCGTAATGGTGTGGTGGTGTTTTCTTTGCAGCCAGTTGGATATCGTCGGGAACAGGAAGGCCGTGGAACCACGTGCCCTGCCGCCTACATAATGCCTTCAGGAAGATCCATGCTAGGCTCGTGAGGGAGCTCGAGAAGAAGTTCGGCGGCAAGGTGAGACACTTGCGTGTCCTCTTGTGCTTTGAGATAGCACTTTCTTGTATCTTATGCTGCTGTGGCAGCGTTGAGCTATTGTGATTAGATGATGCTTGCTGGGTACAGTTTAAGAATCTATTGTTGCCATTTTAGGAAGTGAATTGAGTTTCTAGCACTTTCCACAAACCCAGAACATGATATGCCACGGTTTGATTTCTAGCACTTTTCCCCAGGCCAGAACATGATATGTGCCATGGTTAGTGTATGAAGTTGCTTGTCTATACTAATAAATAGATTTAACAGGAAATCTACATGTCTCAAATGCTATTCCTTTCCTAACATTGTAATGATAGCTAAATTGCACTATAGGTTATCATTTGGTATATATAATAAGGTAATTGCAGCTATCTATCCTACAAGTCTTGCCTTTACTATATGTTCTGTTGTGTCCAAACTTGATTTCTGCTATTTTGTGCTTTAATATTTTGAAAATTGTTGATTTGTAATGTAAATTCATGCGCTTTGTCCCAGATCATCCTCTGCATTAAATGCACCAGTTCATTCAATTTAACACTTGGTTGTTTTGGTCTTGAGACTTTAATTGAGCAGAAAGCTCTTGGTGGTGGCTTTGGGGCAATATTTATCATCTTCATTCGTCTAAACAGTCAAGCTCTTGTGCTCTCTAAATTTTGTTGATTAGTATCTGACATATCTTTACGATGGTCATATGCAGTAGTATATTTATGTAACACCTCTATCATTTATTGAGGAGGTTCTTAGAATACTAAATATGTCAGCATCAAATGGGAAGTGGATCGATGGGCTCCAGTTCTCATCTCTGTTCTGGCCCTGGCACATGATGTGCAGCAGAAACAGGTACCGTACTGATGATTGAATTACGCTAGATACGCTGCATATTTGAAATCACCTGATGTTAATGAGCCACTTCTGTTTTTTTTTCTATTTGAAGGCACAAACTTTGGTCTATGTTGAGTGCTTTGGTCAGCTTACATGTGAGAGCGAAGGATGTAGCTCAGGTATCGTATCTCTTAGGTCTTAGCATAATTTAGTATTTTCTATGATAAAGTCTATATCAATCTGTTGACCTTCTGACCCTTCACTTTTGCTTGTGTGCAGCTCATTTAGAGATGATATCCATCAAATGAAAAACGTTTGGTAGCTGAAGTATCAAGTAAAATATGACTTCTGGTAAGTGGTAACTACATATTATACTATAGGCATTTCAAGAAAAAAAATTGACACACTTTTTTGAATCAACTTTTATTCTTCCTCACCCCAGCATGATCATGCTGTTGTACTCCAATTCATTCACGCATCATAGATGGGATACACTGAGTTCTGGTACGTATGGTTCCCCATTCAATTCCTTACTTTCTTTATTTACCCAAACTTCTGAGGTGAAGCCTTTAAGTACCGGTTACTTTTCTCTCGCTTAATGTTGGTTGAAGTGCTGTTGCTTATAGAGTGTCCTTTTGGTTGTAGAAGAAGTACTCACAGAAGTAAGCCTTGCCCTGTTGAGAAATTGTTAAGAGTTTTACCTCACCACAATAGGCCAATCTTCAAAGTTGCAGAATTGAGCCCCTCACAAATGTATTTTTTCCTTCACATCTTCCAGCAGAAAGTAAGAAATATGTAGTTATTTTGTATGTCTATATTTTAACGTGGTTAGAGATTAAACCGATGCTCTTCTTGCTGATGGTATGTAACTTAATATGTACGTTATATAATTATGGTCCATTGCAGCTCAAAGTTTTTGTTGGACACATGCATCGGTGCTTTATCTTGATTTGATCTCATTTTTTCAGCGATTTTCATTTTAGTTGATATTTTTAAGATCAGTTATTTAAAAACTTGGGTTGCCTTATTGGTTTGACATTAGTATTGCATCTTTCGTTATCATGACCCAGATGAATTATATTACTTCAATGACTGGGTGGTGTGGAAAACAGATCTCTGTTTATTTTCTTTGAAAGTACTCGAATTTTTGTAAATATTTTACCATGTCTAGGTACAATATCAGATAGATCCCAAATACAAAGCTGGAACTTATTCGTTTGTAGCTTTTCAGTTTTGTGCAACCTATTCTTGCTTTAGGGTTTTGTGAAACTGATTGGTGCGTAGCTTCAGCTTCAGGATTAAATCCCAAATATTTTACCACTATTGGTCTGCTGATAACAATGAAAATATACTAGGAGCATTGCTTTTTGGTTTTCCTATTCTTGATTTTGGGCAACTCTTTAGTTCTTTCAGGTTTTTCAGGCCGGCTCATCTTCAGTTTGTGCCTTCCTCTTTTTTGGATTTCCTATTAGTTGTCTTCCTTTTTCCCCTCCTTTTTTTGTTTTTCTGTTCTCGATTTGGAAAACCTATTTAATTCTTTTGGGGTTCTTGCTTCTGGGTTTTTTAGAGGGTTTATTCTTCGTTTTGTTACTCCTCATTTTTTCTTTATTTAAAGTTTCCCCTTCTGTTTATTATTCACCTATATGTTCCTTTTTTTGGCACTTCTTTTGGTTTGTTTGTGGCTGCTGGTTTTTTTTTCTTTGGCCTTTTCTACGCTCCTTGGTCTTCATTTACCTTTTGATATGTGTTTTGTGTTTGTTCGCATTGTCCTCGTCTGTGCATCCGTGAGTTATCGTGTGTGTTCGATGCCCGGTGCATATTTTTATCTCTTTTTAGTACCACACCTCCATCGAGCGGCGAGGTGGAGCTGACGCAAGTAGATACAAACATACTTTTTTTTGGTCTAAATAATAATGAAAATCCCAGCATGTATGTGTATCATAATACTATAGCAGAGCTCGCGCTTTGAAATTGTTTTGTCTAAGAAAAACACATGCATCCCATGATCTGTGTTGTAGAAGAAAAGTTCTCTTTTGTCTAAACGTTATCATTGTCTCATTATTCTGAAGTACAGATGTTCTTCTTTGTGGTCGTTCAGTTATATCTTATGTACCCTCTATATTGTCATTGATTTGCCGTTCAGGAGACCCGTCTGGGCTCTGCTCGGAGATCGCCTCCGCAATGCTGAGCCATGACCGTAGGTTTACAGTGGTGGGGTTTGCTGTGGTTGGTCTGGTCGTGGCAAGGTGTCAACACTGACGATCTCGCCATCTCTGTGCAAGCCTGAGCTAAACGAGGCATCCCTGCTCTCAGGCTGACCTGCAACCTCACGTGATGTAGTGTCTGCTCCTCTCAAAGGTGACATGTTATTTCATGAAATAATTTGTCTTAGGCTAATGGTTCACTTCAGTTTTGATCTCTTTCTATTTCGTAGAATATCTTAGGCTAACGCCTTGGTGGAGACTGATGTAGCCTGCTATGTGGATTCTGAAATAGATTTATACATGAATGCTAAAGCTCCATGGTGAATCATGGTTTTGTCTTTGATTACTGCTTCCAATCATGGTGTAGCTGAAGTAAGATGTTGATTGTGCAGTAGCGCGAGTTGAGGAACCAAGTGAACTTTTGAACTTTTATTATAAAACCAAGAATATTAAATGAGGTATATTTGTTACAAACTAACATCCGTATCCATCCGGTGTAACAATCAAAGAGAGGTATTTGTTGTTTTTATTAAGTTCATACCTTCACGTTAGTTTACCTACCACCGAATGTAGCTGCTCCATCTGATTGTATATTGTGCTATTTACCATGTCATTTATAATAGGGTGTTATTGTAATATATTTTCCTGTTTTCCTGACGGTCTAAGAGTTTTCTACTTTGTTCGTGATTTTATTTACTTCAATACGGAGTACAAAATGTTAATCGATGTGAATTTCACGGGGTCGTGCGCCAAGGCGCATATCTAAATCTAGTCAGGATGAGTACTGGGTGGTGCCGGCATTATGTTCTTGTTTTTTAGTCTGTTTAGTTTGCCACTAAAATGTTTTCATGTTTTGTCTCAACTAGTTGAGGATTCCTGGATATTATTTAGTTTTTTGGTTGCATACTTTATTGAATCTGTCGGTACAGTTGTTGGAGGATAATTCAACCAAATTCAACTGAAAATAGCCGGGCCAGACACAACACACACTCTCTTTCTCAACATGAACGGACTGAATTTTGTTTCACCATTACAAATTATGGTTCTTTGGTTCATCACCACTTTATCCACAAATCACAAAAGAAGATATTCTGAATGTTTCGAATATCCACGGTTTGTTGATATGCTAAGTGCTTCTCTTTAGGCTTTATGCATTGCAGCCGTAAAAAAGCAAAACAGCAAGAATCTGAAACATATCAAATGCTTGGAGAGGAGGTAACGAACAGGCATCCGTGTCTGCAGAAGCAACAACGTCATGGTAACTTGGTCGAGCTCGCCCGTCCGGAGCTGAGTTTCTTAGAGATCCTCGCCCTTGACGTCATGTCGCCGATGGCTTTGCTGTCGTTTCCTCTGCGCAGATGCAACAACACCGCAAGAACAACAACCTGGGCCGGACCAGACGTAGCAACCTTGGGACTTCACTCTTCTGTCTCTCCGTGTTGTGAGACGCTCCCCTCGGTCGCCTCCTCCAGGCGACGGGGGAGCCCAACCCCAGCCCGCCGCCGCCGCCCCCTTGCCCCCTGCCCGCTCCCTCGTCGCCTCCCGAGGCCGCCGGCTAAGCCGTCCGCCGCCGATGAAGGGGGCGGCGAGGACCTATGCCTCTCGGGCTCTCGCACGGCCGTCCCGGAGGGAGGACGCCATGCCGGACCCCGGAGGCCGCCGCCGTCCTCCGCCCCTCCTGCGCCCCTCGCTGTGGCGTCGCGCCTCGCGGCGCCCCTGCTGCCTCCTGTAGTTCGCCGCCCGTCGACGAACAGCCTTGGCGCGGTCTGGCTGGGGAGGGCCCGATCTGGACCTGTTTGGGCTCGATCTGGGCGTGGAGGGCCGCGGCCTGTGCGTCGCTCCCTGGCACTCCCGGCGAACGCCCTGTCCTGCCGTCTCCCCGTGGCCTCCCGCGGTACTTTGCTGCTGCGCGCCCTGGTGGAGACGTTCCTGCCCGCGTCGCCCTAGCCTTGGTGTGGCCCGTTGTCCATGCCGCGGCGCATCTCCGACCGCTCCTCGCGTCGGGGCCCTGCCGGCGGTTTGGCGGGGCGGCTTCGGCCTTCACTGCTCTTCGCAGCTCGCCGTTCGTCCTGCTTCTCGCCCCGTCTGGCCATGGCGCGGCTTCTATCTAGCTTGTGGTGGATGCGGTTCACCTGGCGTGGCGCTGCACGGCTTCCCGGTGGCCGCCTAGTGCTTCGTTGGTGCTAGCGCGCGTGGTCTGGCCGGACGCGCTACTCAGGCGAGTGTTGAGCGTTTGGTGGACGGCAACAAGGCCCCCTGCTAGGCTGGCCACGCCTACGGCCAGGGCCTACTTGGGCCTGACGACAGCGTTGACACTCCAGGCGAAAGCTGCGCACCTTTGGTGCCGATGTTGGCGGTGCCCCTAGGTACCGTTTCCCCTGTTGAGGGCTTCATCGGGGAACTTGGTCTCCTGTTGTCACGAGTCGTTGCGTTCTCCGGGTGAAAACCTCTGCTCCTCGGAGCGAGCGGCGGCGACATACTGTGCCGTTACCTTCCTGAAGGCGCCGCTTTTGGAGTTCGCG

>Lp-psGI.3 (FN376858)

CGGCGGCGCCTCGTACCAGCGGCTCGTGCCCTGGCCAGCGTCGCCGGTGCGTGGAGGCGACGGCCGGTAGATGTCGTCGTCGCTGCTCTCCTCGAGCTTGATCACCTCCCTGGGCGCGGCGTTCCGCGAGGACGACGGTGCGGCGGCGCGGGCGGCGAGTTGTCGCGCGGCGGCCAGATCCAACATCCGCCGCTGCTGCTCCGCCTCCTGGCGCTCCCAGTCGCGCCTGGACCACTCCAGCGCGGCGTCGATGGGGAGGCTGTTGTCGGCGGGCACCAGGTCCTTCAGCGACCTGGCGATCGCCTCCGCCACCGCGGCGTCCTCCGCGCGCTTCGCCTCCTCCTCGGCGAGCTGGTTGGCGGCGGCCGCGGCGGCCTCATTCTTCGCCGTCTTCCTCTTCCGCCCGCGTTGCGGCGAGGAGGGCTGGTCGCGGATGACGAGCGCGCCGCTGCTGCGCCCTCTGGTCGGCGGTGGAGACGCCGGCTCCTTCTTCACGGTGGCCGGAGACGGCCACTCCTTCTTGACGGTGGCCGGCGTCGACCCACCGGACCTAGACGCCGATCTCGAGCCCGACGATGAAGAGGACGGCGCCATCCTCCTCGGTATCCTGGAACTACCGTGCCGGCACGACACGGTAGCCGCCACCGGCGGCGGCATCCCCAGGACAGGGGAGTTCCCGTCCTCGATGTGCGCGAGCACATTCTCGAGGGTGCGGTTGGGCGCGCTCCACCATCGGCGGTGGCCGGCGGCGTTGTTCCTTGCCGGAGGAGGAGGAGGGCCGTCGTAAGCGGCGAGTTCGCGTTCGTACCTTCGCCGGAAGAACGTGATCCACGCTTCGTGGTTGTCGGGGAAGAAGCGCGGATCCGCGCGCTGCTCGTCACTGAGAGTGACGAGCACCTCGTCGATCGCCGCTTCGAGTGCGCCGCCACCCGTTGGCGGCGGCGGGATCGGCACGCCGCCTGCGCTCAGCCTCTAGCCTCCCGGCGCGCGGAAGTCCGGCGGCGCAGGGTAGCCCGCCGCGTGCAGGAGTCGCCCCTCCCATTGGTGGAGAGAGCGGCGGCCGAAGCCGTTGTTGGCCGCACCGTCGTTCGCCATTGCTGGTTCCTTCGAGTTCGGGGATGATTTGGGGGAAGACGAGCGAGGAGGTGAATGCGGGGCAGCCGGGGTAGTTCGGCGATAAATAGCGGTAGGCGCGCATGAAACCGAGGCGACGGCATCAACTCGCCGCGTGGAAGCTACGCGTCCGGCGAATGCTGACCGGCGGCAGGCTTTTACAGCGCGCGGAAGACGATGCGATGAGGACGACGATCGGTGTCTCTCGCCGACAAGTTGGGGCCACCAGACGCGCGGGAACGTTTCACGCGCTTTCGTTTCGTCCGGAGTCCCCGAGCGCTCCCCAGGGGCCGGGGATGGCGTGGGCTCGCCGGATGGATGAAGGGCCAAATCCGGACGAAAACGAGGAACCGGGGGTGCGACTGGGCCGAATTTCGCCGTCCGGATGGGAAAAACGTCGCTCGGGGGCCTCGTCGGGGGGACGAGTGGAGATGCTCTTACATAGTCCTGCTATTTTAACTGTTTGGATTCGCTTCTACTATTTTTCATTTTGTTGCAATTGTAAGTATTCACATGTATATTTTCATCTTCTGGCTGCAAAAGCTGATGTACATGACGCTCTTGAAATTATTTCTGTGTGTGATATTTACTGATTTATCATGTTTCTCTTATAGCACCTTGGTTGGGTGGTGAGGTTGAGATGTCGCGCGGCAGAGTAGCCAATCAAGGCCTATTATTTTTGCAGTCCTATTGAGGTTCTTCTCTCAGTATTTCTTGTGTGAGCTTTCCTTCTTATCTGCATGTTTGGAAACTTGTAGGCCAGATTAAGAGCACTTATATTTTGTCTAAAAGTTTTGCCATCTTTTTTGTCTAAAAGTTTTGTAACAATTCATATCTTTTAGCTTGTAATTTTTTTCCCTCTAATTGTTTCTTGCTTCATGTTGATAGGTCTGCCGACAGGTTCAGACTTTCCAGTTAAGGAATTTATGCATTTTCTTCATGCAACATACATCCTGTGGTAAGTCTTTGATCATGTAACTTCCTAATGGTGTCATGAAAGGCTTGATGGTGAAGATATGATGCTAATGAGATGAATATTGGTTTGTTGATCACGTGAATAGAATTTTCACCTAAGAATAGCTTTCAAAAACTGAGGTCTTGGTCTTTCCATTTTACTGTACCCACCCTTCTCTGTTCGACTCTCGAGCGTAGAGATAACTGTGATATGCATATTTTCTCATAAGAATAGCTTTGGGGCATTATGTACTAGAAAAGCATGCTCCCAGGTTAATGTATTCTTCCTACTTTCTATAAGCATGTTGGTATGTGGGAATAGAACACTGTTTAATTCTTCATTGCTTTGGGTTTTCATAATACATAATTACCATTCCCGTATCATTACCTGGCATTGTCATTTATATGCGCAAATTGGGTGTTCCATCCTACTATATGTCCTTCTATTGCATTTGGAGCTAGAAATCTTAGATTGATTTGCTATGATGGGATATAAGGAAATAGCCTTCAACGCATTTACTTATGTATCATTTTACCTCGTTGCTCTCTACGTAAGCTTATGTTTTTCTATTTCGTAAATTGGCTAAGAAGCAATAAAAAAACATTAGTTTGTATGGCATAATTTTTGTATATAACACATATTGCTTTTTTCCACAACTCATCTGTCTCGCAGGTGAGGGGCTCTCCCCCATCTCCCGTGTTTCCCCTCTGCAATTTCCTTATTTCCACAACTCCTCTTCCGCGTTCGGATGGGGTTAGGTGTAACACACTCATCTTGCAAGCTTTCTTCGATCTGGAGAATGGCAACCAGGAGCTCAAGAGCGACCTAAGGACCTCTACATCAACACCGCGATGTAAGCTGATGTATGCTACATCCCTCTATATCTTCCGTGCCTGCCTCTTTGTTTGGCTGTTGGTGGACCGTAATGGTGTGGTGGTGTTTTCTTTGCAGCCAGTTGGATATCGTCGGGAACAGGAAGGCCGTGGAACCACGTGCCCTGCCGCCTACACAAGGCCTTCAGGAAGATCCATGCTAGGCTCGTGAGGGAGCTCGAGAAGAAGTTCAGCGGCAAGGTGAGACACTTGCGTGTCCTCTTGTGCTTTGAGATAGCACTTTCTTGTATCTTATGCTGCTGTGGCAGCGTTGAGCTATTGTGATTAGATGATGCTTGCTGGGTACAGTTAAGAATCTATTGTTGCCATGTTAGGAAGTGAATTGAGTTTCTAGCACTTTCCACAAACCCAGAACATGATATGCCACGGTTTGGTTTCTAGCACTTTTCCCCAGGCCAGAACATGATATGTGCCATGATAAGTGTATGAAGTTGCTTGTCTATACTAACAAATAGATTTAACAGGGAATCTACATGTCTCAAATACTATTCCTTTCCTAACATTGAAATGATAGCTAAATTGCACTATAGGTTATCATTTTGGTATATATAATAAGGTAATTGCAGCTATCTATCCTACAAGTCTTGCCTTTACTATATGTTCTGTTGTGTCCAAACTTGATTTCTACTATTTTGTGCTTTAATCTTCTGAAAATTGTTGATTTGTAATGTAAATTCATGCGCTTTGTCCCAGATCATCCTCTGCATTAAATGCACCAGTTCATTCAATTTAACACTTGGTTGTTTTGGTCTTGAGACTTCAATTGAGCAGAAAGCTCTTGGTGGTGGCTTTGGGGCAATATTTACCATCTTCATTCATCTAAACAGTCAAGCTCTTGTGCTCTCTAAATTTTGTCGATTAGTATCTGACATATCTTTACGATGGTCATATGCAGTAGTATACATATGTAACACCTCTATCGTTTATTGAGGAGGTTCTTAGACCACTAAATATGTCAGCATCAAATGGGAAGTGGATCGATGGGCTCCAGTTTTCATCTCTGTTCTGCCCTGGCACATGATGTGCAGCAGAAACAGGTACCGTACTGATGATTGAATTACGCTAGATACGCTGCATATTTGAAATCACCTGATGTTAATGAGCCACTTCTGTTTTTTTTTTCTGTTTGAAGGCACAAACTTTGGTCTATGTTGAGTGCTTTGGTCAGCTTACATGTGAGAGCGAGCAATTTCCGAAGGATTTAGCTCAGGTATCGTATCTCTTAGCATAATTTAGTATTTTCTATGATGAAGTCTATATCAATCTGTTGACCTTCTGACCCTTCACTTTTGCTTGTGTGCAGCTCATTTAGAGTTGATATCCATCAAATGAAAAACGTTTGGTAGCTGAAGTATCAAGTAAAATATGACTTCTGGTAAGTGGTAACTACATGTTATACTATAGGCATTTCATGAAAAAAAAATTGACACACTTTTTTGAATCAACTTTTATTCTTCCTCACCCCAGCATGACTATGCTGTTGTACTCCAATTCATTCACGCATCATAGATGGGATACACTGAGTTCTGGTACGTATGGTTCCCCATTCAATTCCTTTTTTTATTTACCCAAACTTCTGAGGTGAAGCCTTTAAGTACCGGTTAGAAGTGTTGTTGCTCATAGAGTGTCCTTTTGGTTGTAGAAGAAGTACTCACAGAAGTAAGCCTTGCCCTATTGAGAAATTGTTAAGAGTTTTACCTCACCACAATAGGCCAATCTTCAAAGTTGCAGAATTGAGCCCCTCACAAATGTATTTTTTCCTTCACATCTTCCAGCAGAAAGTAAGAAATATGTAGTTATTTTGTATGTCTATATTTTAACGTGGTTAGAGATTAAACCGATGCTCTTCTTTCTGATGGTATGCAACTTAATATGTACGTTATATAATTATGGTCCATTACAGCTCAAAGTTTTTGTTGGACACATGCATCGGTGCTTTATCTTGATTTGATCTCATTTTTTTCAGCGATTTTCATTTTAGTTTATATTTTTAAGATCAGTTATTTAAAAACTTGGGTTGCCTTATTGGTTTGACATTACTATTGCATCTTTCGTTATCATGACCCAGATGACGGGGTGGTGTGGATAACAGATCTCTGTTTATTTTCTTTGAAAGTACTAGAATTTTTGTAAATATTTTACCATGTCTAGGTACAATATCAGATAGATCCCAAATACAAAGCTGGAACTTATTCGTTTGTACCTTTTCAGTTTTGTGCAACCTATTCTTGCTTTTGGGTATTGTGAAACTGATTGGTGCATAGCTTCAGGATAAATCCCAAATATTTTACCAGTCTGCTGATAACAATGAAAATATACTAGGAGCATTGCTTTTTGGTTTTCCTATTCTTGATTTTGGGCAACTCTTTAGTTCTTTCAGGTTTTTCAGGCCGGCTCATCTTCGGTTTGTGCCTTCCTCTTTTTCGGATTTCCTATTAGTTGTCTTCCTTTTCCCCCTCGTTTTTTGTCTTCCTGTTCTCGATTTGGAAACCTATTTAATTCTTTTGGGGTTCTTGCTTCTGGGTTTTTTAGAGGGTTTATTCTTCGTTTTGTTACTCCTCATTTTTTCTTTATTTAAAGTTTTCCCTTCTATTTATTATTCACCTATATGTTCCTTTTTTGGCACTTCTTTTGGTTTGTTTGTGGCTGCTGTTTTTTTTTTGTTCTTTGGCCTTTTCTATGCTCCTTTGTCTTCATTTACCTTTTGGTATTTGTTTTGTGTTTGTTCGCATTGTCCTCGTCTGTGCATCCGTGAGTTATCTTGTGTGTTCGATGCCCGGTGCATATTTTTATCTCCGGTGCATATTTTTATCTCTTCTTAGTACCACACCTCCATTGAGCGGCGAGGTGGAGCTGGCGCAAGTAGATACCAACATACTTCTTTTTTTGTCTAAATAATAATGAAAATCCCAGCATGTATGTGTATCATAATACTATAGCAGAACTCGCGCTTTGAAATTGTTTTGTCTAAGAAAAACACATGCATCCCATGATTTGTGTTGTAGAAAAAAAGTTCTCTTTTGTCTAAACGTTATCATTGTCTCATTATTCTGAAGTACAGATGTTCTTCTCTGTCGTCGTTCAATTATATATCTTATGTACCCTCTATATTGTCATTGATTTGACATTCAGGAGACCCGTCTTGGGCTCTGCTCGGAGATCGCCTCCGCAATGCTGAGCCACGACCGTAGGCTTGCAGTGGTGGGGTTTGCCATGGTTGGTCTGGTCCTGGCAAGGTGTCAACACTGACGATCTCACCATCTCTGTACAAGCCTGAGCTAAACGAGGCATCCCTGCTCTCTGGCTGACCTGCAACCTCACGTGATGTAGTGTCTGCTCCTCTCAAAGGTGTCATGTTATTTCATGAAATAATTTGTCTTAGGCTAATGGTTCACTTCAGTTTTGATCTCTTTATATTTCGTAGAATATCTTAGGCTAACGTCTTGGTGGAGACTGATGTAGCCTGCTATGTTGATTCTGAAATAGATTTATACATGAATGCTAAAGCTCCATGGTAGATCATGGTTTTGTCTTTGATTACTGCTTCCAATCATGGTGTAGCTGAAGTAAGATGTTGATTGTGCAGTAGCGCGAGTTGAGGAACCAAGTGAACTTTTATTATAAAACCAAGAATATTAAATGAGGTATATTTGTTACAAACTAGCATCCGTATCCATCCGGTGTAACAATCAAAGAGAGGTATTTGTTGTTTTTATTAAGTTCATACCTTCACGTGAGTATACATACCACCGAATGTAGCTGCTCCATCTGATTGTATATTGTTCTATTTACCATGTCATTTATAATAGGGTGTTATTGTAATATATTTTACTGTTGGTTTCCTGACTGTCTAAGAGTTTTCTACTTTGTTCGTGATTTTGTTTACTTTAATACGGAGTATAAAATGTTAATCGATGTGAATTTCATGGGGTCGTGCGCCAAGGCGCACATCTAAATCTAGTATGACTAATTGTGGCATTGGAATGAGAGACCCCCTCAACTTGTCAACTTGTCTCATATGAGTAGTTCAGTTTACAACCTTATCTGTTTTTCAACTTTAAGACAAGTTTAGGAGCCTAATTTGAACTCCTCTCTACTAGAATATCTAGCCTATTGTGGCAACTAGCAAACATGTTTTCTAAAAAAAAAAAACTCAGTCGAAGTTTTAGTATGGGCTCCTATGCTATTTATGTACAAAATTAGAGGCTCGTGTCTTAACCCTGGATGATCGAGATTTTTCATGCTGAGATTGCTCAAGTTTTATGGAGTACCTTTTTTCTCTTTCATAGTCGAATTCTTTTTCTCGCTTGGGATGCTTTTCGCGTGAATTATGAACGCTATGTTAGTTGATTGATGAAAAAAAAAGATATTTCTTTGTCCAAAATAAGAACAACATACCTTTAAGGAAATCCTTGGCCAGAGAGTCAAAAAGCACACATCACTTGTAAGGAAACTGGCTAGTTCTTGTCCTAAAATAAACGTTTTGCATACGTGGTCGTGTGTGTCCATGTGCTTTCTCTATGACACGGAAGGAATGTGGACAAATCTCAGCACGTGCATTTCCGGTTTGAGCCGAGTTCTGCCCGTTCCTTAAACCACATCGTGTGCCGCAGTTTTGTTTGCATTGGTCTGGTTTAGACGGTTCGGTGCGGGCTGGTCTGACGGTCCCGCCTCTTCCTCACACCACCGTTCGCCATGAAGAGGAAACTTTTGTCGTTCGTCGCAGATAAGAAAGAGCCTCCGTTCGAGGAGAAGAAATGCCCGCCATGAGAACAAACGGATGGTCGCTGCCGACGAGAAGGCGTTGTCCTGCCAGGCCTTCACCAAGCCCTTGAAGGTAGGCTGTATCTTCGTCGCAGCCTCGGCACTGGCACATCTTGTTGATGGCTGTAAAATATACCACTTTTTTCGTGTTTAAACCTGCAGCTAAGTCAAGAAATTGATTAAGTTTACCTCTCAATTTGCTACTAATGACTAATTAATGCAAAGATGTGCAATCTCTTCTATTTTTGAATGGTGTGAAGAAAATCACGCCATAATGACAAATGAACCTGCAATTACTAAAGAAAATTGCGTCGAGTATGACAAATTTGACTAAGCTCGAAGAGGTAGTTCCAAGGACTTTAACGGGCATCGGTACGGACAAGCCCCACCCGTACCGTCCACCTGTACCATGTGCGGCAGCTTTCCCGAGGTCAAACCCTATAAGTTCTGTGAAGGACCCGACGCCAAAAGGAAAGACATTCGTCACCAAATAGAGCCGCCATCAATCAGATCTATCTGCACCACACCGAAGGATATCCACCACCATAGTTCATCCATTCCATCTCCAATCTCCTCCGTGGCCATAGTCATCACCATTGTAATAGAGATCTCCTTGAGAATTGGCGACAATTATAAGAATATTCTGATTTCAATACAAGTATTTGCCACAATGATTTCTATCTTTGTTATTGTCTTGATATTATGTGAGAGTAGTCTCACGGGCTGCAGGTTGGGGTAAATCCTCGCAGCGTTTATGTGCATCTAATCTTATTATATGTGTAATGATTGAATAGGAGTTTTACAATCTTGCATCCCAGTCTCTTTCCCTCGTTTCAATGTACCCATATCATTCAGATCGATCAAGGGAAGAGCTGGGATGAGTGGAGAACGGCATGCTACCGCGAAACCTCAGTGACAGAAAGAGAAAAGTAACGGTACATATTTAGTGATTCATTGGGGATCATGACACATTCATCTAGC

>Fp-psGI.1 (FN376856)

TCTCTACCAACACCGATGAAGAAGCTCGAGCTGAAGGACTTACCGGAATGGATTCCATGGTGGTCGGAGTCGCCGGCGACGAGGAATTAGCCGCGGTGGCTCGCTGAACTTAAGAACTGGATAAACACGGTGCGGCGGTGCTGGCTTCGGCTTTGGCTCCGGTGAAACTCCGGTGGACTCCGGCGCGGTGGAAGCTGGAGCTCGGTGGCGGCGCTCGAGTGAGAGGTGAAATGGGGGAGAATGGGGTGATTAGGAGGGTGCGGTGGGATATTTATAGGCGCGGGATGAGATTCGTGCTCCGGATCCTGTAGTCGGAACGCAAGCGTCGCGCCGTTGGATGATTGACACGTGTCGAAAACCCTAGCGGTAAAAATGGCTAAGGATAAGTTACCGCTCAAATTGTCCGAAAATGGCGCCAAGATTGGCGGAACCGTTTGAATCTTTCAAGGTTCCGGGTAAGGATTCAAAAATGACGAGCCATTATCTCTGCAGGGGAGTAACCCGGAGATCCGTTGTAAAAATGAGGTCGATGGATGAAGTCCCGCGGGGATGAAGGATTTTTTCGGCATGCAAGCTGGAGAAGGAAGAATCGTGAAGTTGGAGTTCTTTGAGTTTCCCCATGTTACCGAGAGGATTTGTCAGAAGCAAAATGATTCAAGCAAGGCGGAAACCGGAGGTGAAACCCGGAGAACTCTGGGGGCTACTGTTGTGGGTATACTTCATGGGTATATCATCGACAGTGCCTAGATCCGGCAAGCCCGGGTGGCCTATAGACGGTGATGGTGGCATATGGCCCATCGGGCGGCCCAGTTGCTGTAGATCAAGATGGATGAAGTCCAGCCCAGGAGCAAGGAGCCGGATCCACCGACCGACCCTGGAAGTCGGATCCGGCCTGGCCCATTAAGGGTAGCCGGATCCAATATGACATATAAGGAAAGGCGGATCCTTGACGTGCACGGCAATATAATATTCCGTAGATAGGCAACTTGTATTCCGGCTAGAACTCTCCTTGTAAACCCTAGATCCGTGCGCCTTTATAAGCCGGATCCCGGGCGCCCTAGAGGCACAACGACAACTCATTGTAACAACGCGAAAGCGCCCAGATAATTCCAGACAAGCAGCAGTAGGCCCTGTCATCGTGCAGGTGTTCCGAAGCTGGGTAAATCGCGTACCACCGTCCCGAGGACTCTCCGCCCGATGGCCCCTACTTCTTCTTCCCCTCATGAGGATCCCTCCTCCGAGGTACCGTCGAATAGGCAACGACAAGGGCTGAGACACTGCCCACGGTGTAATAATCACCAGTATGACGCTACTATATTGATCTCGTTTAGATGGCTTAATCACATCTGTTTATTAGTTAATCTTCCATATTTATTATTTTGTAAAAGTGCCAATCACATACCCGCACTTTTCCCTGCTGCGTCCGAGATTTTTGGATTGGATTCGTGAAGATTGATTCTAGCTTTCCATAGTTTAATTCTACCGGTTAACTCATAATGATATTTCAGGAAACGGACAGAGAGTCATTTCCTTAATGTGGAAGATGGCATCGGTCTTTCCATGCAACAAATACTCAGGTCGGCCATTGGATTGGATCATCTACTACTCAGCAACCTCAGGTCACACATGTGCTGGAGATGGTCAATGCCATGGATGTGCATGCTGGTTACTTCTTCAAGTTAGTTGTTTATTCTAAATTGGATTTTAAAAGTAGTTCCTCCATCCGTTTGATTTATGCTTGTGTGATACCTGTATTGTAGGATCTCGAGTTCGTTCAGTTCCGTCCTACAGTCATATATGGTGCTAGATGCCTCATCACAGGAAGGTTCTATACATTCTACTATTTCATCATGTTGTTATATTAGGGAATCATTCTATACAACGTAAATCCCTGATTTGTGCTCCAACATTGTTCATTATAGTTGTTATCTTGAGAACAAGCCAATTATTGTGATGTTTTTTTTTATTGTTGGATGTTGCTCTTCTCTTAATTGGCAAGTGTAAGCAGTGTGCATTTAGATTTTCATTATTTTCATTGTGTACGAGTGGTTGCAGCCTTTTTATTTTCTCCAGAAGATTTATGAAGGACGTACCATACAGAAATTTAACCTGATTTTTCCTTATTTTTAATTTGTAGTTATACAACAGCTTGGCTCACAGATCAACTTCAGGTCCACATATCATCACTGCCTTTTTCCCCACTTCCACGCCTATAGCAGAGAAACCTAATAATGGAACTGAACTATGAAAAAAGTTTGAATTGTTGTTAAGAGAACAACTTTAAGTAGAAAAACTGTTTTTTATGGAATGATGTTGGTAATTTATTTTCCAGATGTCATTGCAGCCAAAGAACCAAGTAGGTGATGGAGCTTGTGGAAAATGGATATAGGTACAAGAGCATGTGATAGAATATATGAATATCCTGGCCAGCTAGCTACGTTAGTTTTCTCCCTTTCCTGGCAAGTGGCAACATACCACTTCCATAGGTATTATTCTTACATTGTTACATAGTCCTGCTATTTTAACTGTTTGGATTCGCTTGTACTATTTTTCATTTTGTTGCAATTGTAAGTATTCACATGTATATTTTCATCTTCTGGCTGCAAAAGCTGATGTGCATGACGCTCTTGAAATTATTTCCGCGTGTGATATTTACTGATTTATCATGTTTTTCTCTTATAGCACCTTGGTTGGGTGGTGAGGTTGAGATGTTGCGCGGCAGAGTAGCCAACCAAGGCCTATTATTTTTGCAGTCCTATTGAGGTTCTTCTCTGAGTATTTCTTGTGTGAGCTTTCCTTTTTATCTGCATGTTTGGAAACTTGTAGGCCAAATTAAGAGCACTTATATTTTATCTAAAAAAATTGCCATCTTTTTTGTCTAAAAGTTTTGTAACAATTCATATCTTTTAGCTTGTAAAAAAATCCCTCTAATTATTTCTTGCTTCATGTTGATAGGTCTGCCGACAGGTTCAGACTTTCTAGTCAAGGAATTTATGCATTTTCTCCATGCAACATACATCCTGTGGTAAGTCTTTGATCATGTAACTTCCTAATGGTGTCATCAAAGGCTTGATGGTGAAGATATGATGCTAATGAGATGAATATTGGTTTGTTGATCACGTGAACAGAATTTTTCACCTAAAAATAGCTTTCAAAAACTGAGGTCTTGGGCTTTCCATTTTATTGTACCCATCCTTCTCTGTTCGACTCTCGAGCATAGAGATAACTGTGATATGCATATTTTCACGTAAGAATAGCTTTGGGGCATTATGTACTAGAAATGCATGCTCTCAGGTTAATGTATTTTTCCTACTTTCTATAAACATTTTGGTACGTGGGAATAGAATACCGTTTAATTCTTCATTGCTTTAGGTTTTCATAACACATAATTACCATTCCCGTATCATTACCTGGCATTGTCATTTATATGCGCAAATTGGGTGTTCCATCCTGCTATATGTACCTTCTATTGCATTTGGAGCTAAAAATCTTAGATTGATTTTCTATGATGGGATATAAAGAAATAGCCTTCCACGCATTTACTTATGTAGCATTTTACCTCGTTTCTTTCTACCGAAGCTTATGTTTTTCTATTTCGTAAATTGGCTAAGAAGCAATAAAAAAACATTAGTCTGTAGGGCCTAATTTTTGTATAACACATATTGCTTTTTTCCACAACTCCTCTGTCTCGCAGGTGAGGGGCTCTCCCCCATCTCCCGTGTTTCCCCTCTGCAATTTCCCTATTTCCACAACTCCTCTTCCGCGTTCGGATGGGGTTAGGTGTAACACACTCATATCTTGCAAGCTTTCTTCGATCTGGAGAATGGCAACCAGGAGCTCAAGAGCGACCTAAGGACCTCTACATCAACACCGCGATGTAAGATGATGTCTGCTACATTCCTCTAGATCTTCCGTGCCTGCCTCTTTGTTTGGCTGTTGGTGGAGCGTAATGGTGTGGTGCTATTTTCTTCGCAGCCAGTTGGATATCGTCCGGAACAGGAAGGCCGTGGAACCACGTTCCCTGCCGCCTACACAAGGCCTTCATTAAGATCCATGCTAGGCTCGTCACGGAGCTCGAGAAGAAGTTCCGTGGCAAGGTGAGACACTTGCGTGTCCTCTTGTGCTTTGAGATAGCACTTTCTTGTATCTTATGCTGCTGTGGCAGCGTTGAGCTATTGTGATTAGATGATGCTTGCTGGGTACAGTTTAAGAATCTATTGTTGCCATGTTAGGAAGTGAATTGAGTTTCTAGCACTTGCCACAAACCCAGAACATGATATGCCACGGTTTGGTTTCTAGCACTTTTCCCCAGGCCCGAACATGATATGTGCTATGATTAGTGTATGAAGCTGCTTGTCTATACTAACAAATAGATTTAACAGGGAATCTACACTTCTCAGATGCTATTCCTTTCCTAACATTGAAATGATAGCTAAATTGCACTATAGGTTATCATTTTGGTATATATAATAAGGTAATTGCAGCTATGAATCCTACAAGTCTTGCCTTTACTATATGTTCTGTTGTGTCCAAACTTGATTTCTGCTATTTTGTGCTTTAATCTTCTGAAAATTGTTGATTTGTAATGTAAATTCATGCGCTTTGTCCTAGATCATCCTCTGTATTAAATGCACCAGTTCATTCAATTTAACACTTGGTTGTTTTGGTCCTGAGACTTTAATTGAGAAGAAAGCTCTTGGTGGTGGCTTTGGGGGAATATTTACCATCGTCATTCATCTAAACAGTCAAGCTCTTGTGCTCTCTAAATTTTGTCGATTAGTATCTGACATATCTTTATGATGGTCATATGCAGTAGTATACGTGTGTAACACCTCTATCGTTTATTGAGGAGGTTCTTAGACCACTAAATATGTCAGCTTCTGGCCCTAGCACATGATGTGCAGCAGAAACATGTACCATACTGATAACTGAATTAGGATAGATACGCTGCATATTTGAAATCACCTGATGTTAATGAGCCACTTCTGTTTTTTTTCTGTTTGAAGGCACAAACTTTGGTCTATGTTGAGTGCTTTGGTCAGCTTACATGTGACAGCGAGCAATTTCCGAAGGATGTAGCTCAGGTATCGTATCTCTTGGCATAATTTAATATTTTCTATGATAAAGTCTATATCAATCTGTTGACCTTCTGACCCTTCACTTTTGCTTGTGTGCAGCTCATTTAGAGTTGATATCCATCAAACGAAAAACGTTTGGTAGCTGAAGTATCAAGTAAAATATGACTTCTGGTAAGTGGTAACTACATGTTATACTATAGGCATTTCAAGAAAAAAAATTGACACACTTTTTTGAATAGTATGCAACAACTTTTATTCTTCCTCACCCCAGCATGACCATGCTGTTGTACTCCAATTCATTCACGCATCATAGATGGGATACTGAGTTCTGGTACGTATGGTTCCCCATTCAATTCCTTACTTTTTTTATTTACCCAAACTTCTGAGGTGAAGCCTTTAAGTACTGGTTACTTTTGGTTCTCTCGTTTGATGTCGGTTGAAGTGCTGTTGCTCATAGAGTGTCCTTTTGGCTGTAGAAGAAGTACTCACAGAAGTAAGCCTTGCCCTGTTGAGAAAATGCTAAGAGTTTTACCTCACCACAATAGGCCAATCTTCAAAGTTGCAGAATTGAGCCCCTCACAAATGTATTTTTTTCCTTCACATCTTCCAGCAGAAAGTAAGAAATATGTAGTTATTTTGTATGTCTATATTTTAACGTGGTTAGAGATTAAACCGATGCTCTTCTTGCTGATGGTATGCAACTTAATATGTACGTTATTTAATTGTGGTCCATTGCATCTCAAAGTTTTTGTTGGAGATTTTCATTTTAGTTGATATTTTTAAGATCAGTTATTTAAAAACTTGGGTTGCCTTATTGGTTTGACATTACTATTGCATCTTTCGTTATCATGACCCAGATGAATTATATTACTTCAATGACTGGGTGGTGTGGATAACAGATCTCTGTTTATTTTCTTTGAAAGTACTAGAATTTTTGTAAATATTTTACCATGTCTAGGTACAATATCAGATAGACTACTATCGCTTTTTTAATGAAAGGATTAAATCCCAAATACAAAGCTGGAACTTATTGGTTTGTAGCTTTTCAGTTTTGTGCAACCTATTCTTGCTTTTGGGTTTTGTGAAACTAATTGGTGCGCAACTTCAGGATTAAAACCCAAATATTTTACCACTATTGGTCTGCTGATAACAATGAAAATATACTAGGAGCATTGCTTTTTGGTTTTCCTATTCTTGATTTTGGGCAACTCTTTAGTTCTTTCAGGTTTTTCAGGCCGGCTCATCTTCGGTTTGTGCCTTCCTCTTTTTGGATTTCCTATTAGTTGTCTTCCTTTTCCCCCTCCCTTTTTTCTTCATGTTCTCGATTTGGAAAACCTATTTAATTCTTCTGGGGTTCTTGCTTCTGGGTTTTTTAGAGAGTTTATTCTTCGTTTTGTTACTCCTCATTTTTTCTTTATTTAAAATTTTCCCTTCTGTTTATTATTCACCTATATGTTCCTTTTTTGGCACTTCTTTTGGTTTGTTTCTGGCTGCTGGTTTTTTTGTTATTTGGCCTTTTCTATGCTGCTTTGTCTTCATTTACCTTTTGGTATGTGTTTTGTGTTTGTTCGCATTGTCCTCGTCTGTGCATCCGTGTGTTATCGTGTGTGTTCGAGGCCCGGCGCATATTTTTATCTCTTCTTAGTACCACACCTCCATCGAGGGGCGAGGTGGAGCAGACGCAAGTAGATACCAACATACTCCCTCCGTTCAAATTAATTGCCTTAACATAGATGTATCTACACACTAAAACATGTCTAGATATATCAACTTTTAGGCTATTATTTTGAACCGGAGGGAGTACTTTTTTTTGTCTAAATAATAATGAAAATCCCAGCATGTATGTGTATCATAATACTATAGCAGAGCTTGCGCTTTGAAATTGTTTTGTCTAAGAAAAACACAGGCATCCCATGATCTGTGTTGTAGAAAAGTTCTTCTCTCTTTTGTCTAAATGTTATCATTGTCTCATTATTCTGAAGTACAGATGTTCTTCTCTGTTTGTCGTCGTTCAGTTATATCTTATGTACCCTCTATATTGTCATTGATTTGTCGTTCAGGAGACCGTCTTGGGCTCTGCTCCGAGATCGCCTCCGCAATGCTGAGCCACGACCGTAGGCTTGCAGTGGTGGGGTTCGTTGTGGCCGGTCTGACCCTAGCAAGGTGTCAATACTGACGATCTCGCCATCTCTGTAGTAGCCTGAGGTAAACGAGGCATCCCTGCTCTCAAGCTGACCTGCAACCTGACGTGATGTAGGGTCTGCTCCTCTCAAATGTGTCATGTTATTTCATGAAATAATTTGTATTGGGCTAATGGTTCACTTCAGTTTTGATCTCCTCTTTCTATTTCGTAGAATATCCTAGGCTAACGCCTTGGTGGAGACTGATGTAGCCTGCTATGTGGATTCTTAAATATATTTATACATGAATGCTAAAGCTCCATGGTGAATCATGGTTTTGTCTTTGATTACCGCTTCCAATCATGGTGTAGCTGAAGTAAGATGTTGATTGTGCTGAGTTGAGGAACCAAGTGAAAACTTTTATTATAAAACCAAGAATATTAAATGAGGTATATTTGTTACAAGCTAGCATCCGTATCCATCCGGTGTAACAATCAAAGAGAGGTATTTGTTGTTTTTATTAAGTTCATACCTTCCAGGAATATACATACCACTGCTCCATTTGATTGTATATTGTGTTATTTACCATGTCATTTATAATAGGGTGTTATTGTAATATATTTTCTTGTTTTCCTGACTGTCTAAGAGTTTTCTACTCTGTTCGTGATTTTGTTTAGTTTAATACGGAGTACAAAATGTTTAATCGATATGAATTTCACGGAGTCGTGCGCCAATGCGCACATCTAAATCTAGTTGGTATGGTTGGTTAGTGGGCACGAAGCAATGACCATGTAAAAACACTCACCATTTTGACAACTTAAATGTTCAGACCATATATTTGAGATCCGAGCCAAAACAAAACAAAGAACTTTATATAATGAAGTTGTACCAGTATATTAGCACTTACGGTGGTAACCCTGATCCCTGAAGGGCCGGAAGGGTCATCTACATAATTGCTTGGGTAGAACTAACGAAAAATAAATAGAATGGGAGGCTTAACGCAAAGGGAGGCTTAACGCAAAGGAGCACTCGAGACATCTCCCGTTCATAGAAGTGGAACCTGAGGTTCGACCAAGGGGTGAACAATTATCCATACGAAATAATTTACATGACGCCGCATCCAGGTGACCTGATCAGCGTTGTCAGCAGAAGTTCAGATCGCCTTCTAACTCCAGCATAGTAGCAAGCTTCCATTGTAATTAGTACAAAAGCGATATAACCTGCAAATAAATCGTTGGTATTTTGGAGAGCGTGAAAGAAAACTGGGAGAGAATAACAGAAATCGAAACTGCCTCCGGCGATGATTGGCTGCCCCCATGTTACCTAGCCTGTTGTCAGCAATGTTCTTGACATCCAATCCAAGTGCCTCTCGCCTCCGATGGTCCTAGAGAACTGTCTCTCGCATGAACTGGCGGACATCGGCAGTGCTCTAGACACGGCATCATCCACCTGGCACCTGCGGCGTGATATTCAATGTAAATTAAAATTAAGAGTAAGAGAATAAGAGATGAGAGCTTGATCTGTACCAATGCCCAGTAGTACGTACTCTGTAATATCCAATCAACTCGGTGGGGAAAAACGTCAAGCAGCTTTCGAGCCGGGCAGGAGCGCAACCTGAAAAATTGAGAAACATAGGCAGCCGGATTAGAAAAGAAAGGAGAGTAGAGGCCGGCGCCAGACATGGCGACGGGAGTGTAGGCGGGCGCGGTCGATGTAGATGGCAGCACGGGGGTAGTTATGTAAGATGGAGGGCGGGGCGGCTCATCCCTGGTTGAAACGGACTGTTTCTACTCCATGAATGCAGGATTGCATCAAGCAGTTTCATGAGCATTATGGAGGAGGCCACAGGTCGCGGAAGCGGGTGAGATGGTGCGTCGATTGCCATTTTTCTCCGGAAAACCAATTCCGCCTAGGTATAGACGTCTCGGTCTTATCACACACCTCCATTGCACTCAGCTGCATCAAGATTGGTGCTCATATTTTGTAAAAAAGTCCAGGAAGCTAATATAGTTCGCATCAAAAAGTCCGTCAAATTTTGCACATCACTCCTCTTACTTTTTTGTTCTTCCTTTTTTGAGAGAACTTTCCTTGTTCTTCCTGATCGAATACATTATTCCCAAGATCTAGGAAATCTTATATTTTAGGAAACTTATTTTTTCAATCCGGAGAACTTATTTTGACCAGAATTTATTTAGGAAACCATTTATTTCGGTCCGGAGCAATTTGTGTCGAGATTCCTTATAATTAGCAATTTTTCACCATCATAGCTATTTAACAAATTTTCACCGAATTAAGTAACAAATTTTCACAGAAGATTTCTTCCATGATTAGTATAGGATTCATACACAATGCGTACAGGATTCAAACAAAATTCATAGGGGATTCATATAGTTCCATACCCAGAAGACAATTTTAGTAGGTATCAAAGCAAATCCACCACGAAAGATGGGGCATATCAGTTTATGAAAATGAAGAAGAAAATAAACTAGATCAAAATCGAGAAACGGAACAAAAAACATGCCAGATTCAACCCTCCATGGTGGGACGTGGGATAATTAGCAATTCATTAGGACAAGAAAATCCATCCACTGCCGATTTCTCATACACAAATTCAGATTATTCAGAGAAAGAAAAGGGAAAGGGAGGAAGAAATCCGTTAATTTTTGCCCTTTCTCCGCTGTAGAATCTAAGAGAACACATAGCAGAAAAAAACCATCCTCATCTTGTTCAGATCGAGAACATAAAACAGAAAACAAAAATTTTGTCAAAGAAGATAAGAGGAGGATGAAATGGATCCAGATCCAGGAAGAGGAGTGGAACCCGTCGTCGATCTGGTGGCGACGTGATTTGGCTACAAGGACTCAGCCGCCGGCGCCGGCCGCTCCATCATCTTCCTCCCGGCAAGGTCGGGGTCGTCGAGATCTGTGGGCGAGAGAGAGGGATGGGCTCATGGTGGACTGGTGGTGTAGAGAAGGGGATGGGGATGCAAGATGGGCTGCCGGCAAGATGGGCTGTACACTTTTTCACCTACCGGGAAAAGAAACTGCACAAGTCAGGGTTGTGAGTGTAAATTGTGCAGCACGAATCTCAAATAAGTAGTACACCGTGCAGATAAGCCCGGTACCCCTATAA

>Fp-psGI.2 (FN376854)

GATAGTTGAGACCTACCTCGTTGCCAACCCTGCCGAGTACGCCTGATTCAGCCAAGATGAGAAAGGTGACCGTGATTCCTCGGAGGTTGGTGTTTGTTTCTTTGCTGGTGGAGTCTGAGAGAAGAATAAAGACGTGATCATGAGTGTATGTGAAATTTGTGTGAGTGTTTAATCCTTGTTGTATGCTTGTGTTTATTTTGCTTCAAGATATACTACAGAAGTATATGCATATGGTCTGGTTTTGTGGTGAATGTATCATATATTACATGTATGATGTGTGGTGTCAATAGACTTCAAATAATCAACGTATTTCTCTCTCTGAACATCTGACCCATGAACATTTTGTGAGTGGTCTGGTAAGTCAAGAACAAGAATTCATTAACGTGAGGAGGGCGTGCAAGTAAACCGCTGGATCTTCAAATTTAAGCGCCATGCTGCTGGTTATGTTGAAAGGTTTAAGGCCACGCTTTGCTGCCGGCATGCCAGCACATGCGGAGATCAATCTTCTTCCTTTTTATTTGTCATCGTGAAAGTTGCAATTGCCCTTCTTCCTCCTCTACCCACTTCTGTTCTTTATTCATCCATTCATTCAAGGAGATGAGATCCATTTTCTTGAATTCCTGAACCTCCTCCCCTCATGTTGATCTTAAAAGGTTTACAATAATTTAGTGTTGCGGGAGACGCCCAAAAAGCGGGAGACGCCCAAAAAATCTCCCCCCAAGGTCGCACGCGCGGGCGACCCCGGAGGTCAAAACCCTAGATCGAGCGAACCTGCCCCGTCCCGGCCCTCCTGGCCGCTGCCCCCGCCGAGGCTGGCGGTGGTGGCGGCGCCCGACCGTCAAAGGGCGGCAGACAGGTCTTGGCGAGCCCCATCGGGCCTTCCTCGCGTGGGGCGGGACGATAAGCTGGGCGGCGCGCGGGGGCGGGACGATAAGCTAGGCGGCGCGCGGGGGCGGTGTCAAGCGGCGGCGAGTTGGAGCGGAGGTGCTGGTTAGGCGTCTTGGTGGTGAGGCATGCGTGCGAAGCGGGGACCAAGGCGACGGTGCATCGAGGCGGCAGTGCGGTTCCCTGGGTGGTGGCGTGGGTCTTCCCCGGCCATGGGGTTTCGGCAGGTGGCGCAGCAGCGGTTCTACCGGTGTTCGGCTCCTAGAGGCGGCGGAGGCCCTGGAGAGGCAACGCCGGTGGGGCTAGTGGCTGGCGGAATGGGCCAGATCTGGGCCCTCCCAGAGTCCCAGGCCCGATCTTGGCCTTAGGGCTTCGGATGTAACACAGCTATCACGGGGTGACAGCTACGGTGCAGGGAATTCTCGACCGTGCAGGAGGTGGAAGAAGGGTGGGGGGCTATGGGAAGAAGGAGGGATCTCGTTCGTGCGGGTCGTGTGAGTTCCATGACATTTCAACGGCTTTGGTGAAGTGCTGCGGCGACAACGGTGTGAACAACTTGGACGTTTGTCCTTGTCCGGCGGGTAGGGTGGGAAGTATGCGGCTTTCCGGACGAAAGTCTTGCCCGCCTTGCTCGGTGCCAGCGATGACGACTCCCGCGAGTGTCGTTCCCCTCCTTGGAGGCGTCGCGTAATGTGTCGATGCATCTTGTTCTCTCTCACTGGTCTTGTAGATGTCTTTTGGAGAAAGCCTATAGGTCCTGAGTTGGATTGGCGCGATGGCGGCGTCCTCAACGCCGTCACTTTGTTGGGAGCATCGCGTTTGGAGACACGACATGGAGGTTCCTGGTTGTCCTCCTCTGGTGCTCGCCGCTATCCATGGTTGTTCTTTAGGGGCACAATGCACGCCATTGTCGGTGACTCCAAGACGACGCCTTCTCGGGCCCAACCGAGTTCCGCCATCTATCTCCTTTAGATGGTGTGTCGACAGGGAGTCCTTTTGCAATTGTTGTTCTTCGGAGGCGTCGGTCGAGACGCGGCTTTGTTGCTTAGCTTAGGATATGCTCTTTCATCTTGTGTTGCTGTTTTGTCGGTGGTGGCTTTTTTTTGGACTAGTCGGTGTGGAGTTATTGCTACGCTCGTTAATCGCAGCGAGTGTTGTGTTCTTGTACTCAAACCTCTGCCTTCTATAAAATTATGGTATGCCTCTGGCGTACTCTCAAAAAAAAATAAAGGTTTACAAATTTGATTGGGAATTTTACTTTTATTTAAATACAAACAAGAAAGAAACAATTGAATATTTCTCCCAAACCAATACATAGAATGAATTGTCCTGCAAAATTTCATTTTTCCAAACAGGTGGGCAAGAAGTCAGCCAAGCCGCGCAGAGACCAAAGGACCTGCAGTACCTAATGTGCGGTCAACAATGTAGGGTACTCGCTTGATATTATGAACTTATTGTCGTCAGTTGGAAACAATTTTCCATCTACAACACCTAGGTATGCGCTAGACACTACGTCACGCACGGCAATTTGCGGCACTTTAATGCAGGCACTTTTGTAACGTGCCGTTAACGATAGGTTTAGGCGGCATATATTCTCAATAGTGCCAGTGATGATCATTTGATTACCGGCTGTTAGAAAAAAGTGCCGCCAATGCTGGGGGAGCACAGGCAAAACTGAAAGTGCCGCTAATTTTTCGCTGATTAACGGCAGCATCTAAAAAGTGCCGCAAAAGCTAGCAGGATTAGCGGCAGTTTCAATCTGCCTTTGTTGTATGGGTGGATGGGTGGACTGGGTCAAGTCTCTCGAGAAAAGTCAACAGACCCCTTCTCTAACTCCTCTCTCATCTCACTGCACGAACGCTCGCTCGCTACCTGCCTGGCTTGATTCCCCTCCCACGCCCCTGTGCGATGGCAGCCGCCGCCGCCGTCCACGGCTCTGATCGTCGCCGGCCGCCTCCACCGCTGCGTCTCGCCGTCCCAGATTCACCCATATCTTCTCCCACGACTCCTCGCCGCCGCGATGCTGCTCTCCTCCCTTGGTCGCCCCCACGGCAAACCCTAGACGTGCGCCTCGGGTTCGCCGGCGTGGCGGCCTCCCCGCGCCTCCGCATGCTCCCTTCCGACAACTACGACTCCTGACGTCGTCTACGACCCCGCTCCAATCTCCGGTACCATCGCTAGTCCAGCCCGACGCCTGCGACCACCTCCAGGATCCCCGCGTCGACCACGTTGCTCACCTACTCCTGCTTGCCTCCCCTCCATGTCCCCGCACGGTCTGGCTTCTCCAAAATCCAGATCGCCAGTGTGGTGGTGGTGGTGGTGCTTCTGCTGCTCCACTACTACCTCGGCTACTTCCACGACGACTACTACGTCTGCCTCGCCTACGACCTCAACTCCCCCTGCAGCTCGACGGCGGTGGTTTAACCGCACAGCAACTGAAGCAGTGGTCGTTTCTCCGAACGTGTACGGTCAGGTAGCCCTTCGGTTCCCCCATGCCCTCTCTCATATACTGTCTTGATCCGGCCACTGTGTCGTGGCCAGTTCTAGTTTTATGTCTGTGGTGGTTCATGTTAGTTCTACGATGATAGAATTATAAGCTTAGGTTGTGGTGATGTTCATATTTGCTCTGTTTTTTTATACTAAAGGTGTAGTTCTATAATTTATAGCACCTTGCAGTTCTCTTTTGTATGGTTTTATGGCCATTTACCTATGAAACTAGTTGCTCGAGATCTCTCATTAAATTTCAGTTGCACCTTGAGATCAACTAATGGTGTCTCTCATGTATGTCATGTTGAAGATGACAATGGTCTTGATCTGGAGTGGTATTTGGTTCACACATGGGAAACATAGTTTCGGAATGAATTGACCTTTATAATGTCACAAGGGAAATACCCATAGCCTAACTGTTTTTAAATTCCAAATTTATATCTATCAGTACATACCAAGAACATGAATTTTAACCACTTTTTGATATCTACCAGTACAAAGTTTTTGAATGAATGTCCCTTATTATCAATATCCCTTATAATTATATCTATGAGCTCATGATAGACTTTGACCCAAGGTAAGGAAAAACTTATTCGTGTTGTTGTTTTAAATGTCCTTAATTTGTTATCCGCAGATTAGGAGACTAAAAATGTCAAATGTTGAAGTATTTGGAAAATATTGCTCTTCTCGTGCTATCCTCACCCTACCCAATCACAAACTTTTCAATTGGTTCATGTGGGATTTCTGCCTCTCAATTTGCCCTTAATTATAATGACAGTTATACTTGATGTGTGTGTTTTAGGGTTGTTAAGAGCTATTTATTATCTAGCGCAGTACCTGATCAAGGGTTGATAAAAATTACAGGGATGAAATATATATAAATTAGGACACATGTAACTTTTGAGGTAATCTTGTCTGCCTTGACTCAATTGACAAAAGTTGAGTCTCCAGATCTTTGAGTACCTTATAAGAAGAATAGAACATGTAATAGATGGATAAACTGAGGCGTTTTCAGATTGTTATCGAGTAGAGACACTCTTATTGCGGAGCGGATTTGCTATTTTCTTACTTTTCTGAGAGTTTCGCTCTAATTTCTCGAGCAGGATCTTAAACTACTAAGTATGTCTGCGTCAAATGGGAAGTGGGTTGATGGGTTCCAGTTCTCCTCACTATTTTGGCCCCCGCCACGCGATGCACAACAAAAACAGGTACCATACTGATAGTTGAATTACGTTATATATGTTGTAGCTTAGGAGACACCTGATGTTAACGAGACACTTATGTCGTGTTCTTTGGGCTACGTTGAGTAATTTGGTCAGTTTATGTCTGACAGTGAGCAATTCCCAGAGGATGTAGCTCAGGTACTGGATCTCCTAGCATAGTTCAGTGCTTTTTATGGTAAATCTTTATCAAGTGTGTTAACTATTAGATCCTTTTTGTTGCTCGTGTGCAGCTCATCCAAAGATGCTATCCATTGAAAGAAAAACATGGTAGATGAAGTAATACATGAACTTCCAATAGCTATATGTTACACTAAGAGAATCTGAAGAAAGAAATTAACACACCTTTTCTACGTTACCGGCAACCTTTGTTTTCCATCACCCCGAGCATGGTCATGCAGTTGTACATCCAATTCTTTCACGCATCATAGATGGGTCATTGAGTTATGATAGACATGGTTCTCCATTCAATTCTTTCATCTCTTTATTTACCCAAACTGCTGAGTTGAGGCCCTTACTTTCCTGGTTATTTTAAATTCTCTTGCTCGATGCTGGTTCAAATGCGGTTGCTCAAAGAATGCATTTTCGTCTGCAGAAAGAGTTTTCAGAGCAGTGGGCTTTGGCCTGTGGACAAATTCTTAGAGTTCTTCTTACTCACTACAATAGGCCAATCTTAAAAGTTGCTGAATGTAACGACCACCTCTAGCCAGGCGACAAGAAGTTATTCCTTACAGTAGAAAGCTAATAGCTCTCCAGGAAAGCTAATGCTGTCTAAAATTTCCTTTTTCCTTTTTCTTATGGAAGGTGACTATGATAGTCTTTGCTTTTTAAATTAGCTGATTACCCTCTGCTTCATTTCAAATACATCAGGTTTCCTTCCACTACTGGGTATTGTTGTTACTTCATCCCTACTAAATTTATGCTTTGCTTTGATTCAGTTTCTTCTGTATCATGTTTGTTCTAGTAATGAATCAAAGGTACTAAGGAACAAGCTAGGTGTTTGTGTGTATTGTTGTGCTATCTTGTAGCTACGAGTGATTTTTTTTATTACATCTGTTCCTACAAAATAAGCAAAACTGGCACTTCATGCAAAACTTGACATGCCACTGAATTTACAAGTGTTTCCCCGGTATACACAAAGAGTACCAGCTTGTACTTTTATTTACTATACTCAAATGGACCAACTTGTTCAAACTTGACACAACATGTACTTGGTGTATTAATTCAGTACAGTTTGAAAAGTGGAACAGACATGGATGTTATTGCAGTCCCCATCAACTTGGTACAGTAATACTGACAGTTCTTTACCAGTTCACCATTCAAATACCTCTGAATGTTCTGTTTATTTTATTGCTTGTTGTCTGCTTACGTAAAATACAATAAGATTTCAGTCAAACAATTACTGGAGTATTGCAAATTCAAATAACTGCACCAAATTCAAATAACTTCCTGATGCCAATGGTTGCAGATCACAAGCGTGGGAGTATACATGCATTAATGTGTTTTCATGTAAGCATGTCTATCTATGCAAGTGCATTCTGCCTTTCACTATGATAATACTTTCTAAGGAAAATAAAGGAGTATGAGAGTGCTTTTCAGAAGTAGTGTAATGGGGATGCACAACTGAACCTGCACATAGCTATTCGTCACAACAGAACCATGTCATAAAAAAAAACCTAGAATGTTTCAATATTCGGTATCGAAGTGTAGCCAAACACTGGTTGTGAAAACACATTATATTGGATCATGCAACATATATTTTCCCAACTTTTCAAGTAATAAGTATAATACGCTATCTTAACTCTTGATTTGCATGTCATCTGATGAACGGAACTGCGTTTTGAAACTGCAGGATGAGCAACAAGATGGAGGTTGAGGCTGCAGTGGAGGCAACCGTCCAGTTCCTGAACAAGGCAGTGAAGCTGGATCTTTCTGTCTGAGGATTGATCATTTTCCCCTCTGTAATTTATTCAGAGAGCGCTTCAATGTTACAGCTATATACAAAACATAACTAGTGAACTAACTAGCTGGATAATCTAAACCTAGTGAATTAATCTCACCATGTAATTCCAGTTCGAGAGATTGTTCTATAGGTTTCAGAGTAGAGAAAATGAATACAACTTATCAGTACTTTATAATGTGAAATTTAAGTTATAATATTGCTTAACACTGTCCCACATTCTACATAGCTTTTTGGATTTGTTGCCATTTCTAGGAGATTATTTCTCTGTATCCAGAATTATTTTGATCAAATAATTGCCATTTCTAGTTGCCATACACACACTTATTGCCATGGTACTGTCACATGATCTCTAATTTGATTTCGATTGTCATCTCCTTCCTCCCAGTCCGGCCACCATCTCCAGTTCTCAAATCCTTCAAGGTATGCTATCCTACTGTCCTACACCTCTGTTCCCCCTGTCTCGCTTCTTGTTTTCTATCACGATAAACTACTGGAACTGCTGGGGCTGGGATTGGCAGTCTGTTTTCCACCCAGGCTAGAACTCCTGCATCCCTCAACCATATTCATATCACAGGTTAGATGAATGAGTCTGCTATTCCTTTGGCTGATTCCTTCTTCCTTTTTCATCTAATCACACCATACCACCTTTTCTTCTTCTTAGGGTTCTCTTGAATCCGTTTCAATTGGTGAAATAATACTAAGTTTCCACAGGAGTGCCATTTGCTTGTTTTTAATTTCTGTATGATATAACATCTAGTGTGTTTAGAGTTGTGTAGACAGTAACATATCTAGTGTGTTCCTGTTTACAGACGGATCTTCTCAAACCGAAAGGTAGGATTGAGAAGAAAAGTCCTTATTTGCAGGAGCTACAAGACCAAGTAAGGATATGACTTTACCTGTTTTTTCTCTTGTTATTTCAAATGAAGTATATGAGATGTTCCTTCTGTTGGTTGATAGGATAGTGTAGTTTTAAGCTTACTAGTAACTCCATGAGAACAGGTTTAAAATTATTTCGGAATCCTATTTTGCGCATGTTGTTTGTTGATGCCTTCCTTGCATCTTTGCTGTTTACTCCACTTTATTCTTCTACAACATCAGTTGTTGTTTCAGGCTGATAATTCTTGGTGGCTGCGGTGGTGCTAGAGCTGGTGATGCTGTTCAGGTGATCCTGCTATGGTCTCCCACACTTGGAAGAAGGGCGACATCTGCAATGTTTACATCGTCGCTGACTCGTCATTCGCGTGGAGATAGTTCCCAGCCTCGAGCTCCAGGTAGGCATCCTGACCCGCCCGCCTCTCCCCTGCGGCTCACTTCTTCTACACTGACTAGAACTCCATTTGTATCTCTCGTGCTTGCTACTCACTTGTGTTGTTTGCATTGCTACTCACTTGTGTTGTTTGCATAATTCACACATTGTCCATAATTATCTAGACAACTTTTATAGACCGGTAGAAATTGTACAAATATTAGCACTTACCTGAGTAGAAGCACATGTTCCTTTTATGACATGATACATGACAATTTAAAATTATCCTGCTTATTAGAAAGTGAGAAGGCCTAATATCACTTCTGGGTCATAATCTATTGTTGGCAAAAACTTTTGTTTTCTGTTGTACCCATCAGTGACTAACAGTCATCAGTATGTTTGCGTTACTGTTTGTGCTAAAGCTTTTCCATAGAAGCTGCCATTGTTATCATGTTGTAGGCTCAAGATTGATAATCTCCTTACCTCGTTTCATTTGCATGCAGCGCATTTGTATTAATCATAATTCACTAATGTTTACTATAGTTTACATTTCGTAACTACAGTTTACTGTTTTAGAAATAAGTATAATGGGTTTTCATTTTTCTTGCAGATTCTACCAGCACTGTCGCTGGCCCGTTCCCGATGTTCCCAGCTGGCCACACCCCCAAACAACAGCAACACAAGTACAGCTGATGGATGTTACTATGCATCGGCCTGGCACAAATATGTCTGTTCTAACCTATGAAGGCTTCAGGTGGTAGGGTGATGTTTATTCTAGATCTGATGGTAGGGTGCTGACATTGGAGGAACAAGCAGCAAATCGGGAACAAATGAGGAAAATTGAGAACCCTATTGGTCAGACAAGTAAGATTGAAGCAAGTTAGGTGAACCCTGTTAGATGAACCTTGTTTTGTATTTTATCTAATGGTTTGAAACATGTTTCAAACCATGTTGTTCATGATATGGAGATGAACCCTGTTGGTCATGTAAGTTAGATGCCTGGATTGTGAACCCTTGAATGTTTCAAAATGTTAGTATCAGACGTAGATTGCTTGGAAATATTGGTTTTAAACATTGCTTGACTTCTTGAGTGTGATGCATGCTTGACTGTTAGATTGCTTGAATGTTTGCAGATAGTATAGCAATTTTGAAAATGCCTAAGAAAATAATCAGCGGCACTAGAATTAAAAGCCTGAAAAAATAATTGGAGGCATCTCCGGAAAAGTGCCGGCAAAAAAATTTCGCGGCATCTTCGGAAAAGTGCAGGCAAAAAGATTTCGTGGCATCTTCAAGAAGTGCCGGCAAAAGGATTCCACGGCATCTTCAAAAGTGCCAGAAATAGTTAATAGCGGCATTTCTAGAAGTGACTGAAAATACAACCGATAGCATTTTTCTGGAAGTGCCGGCAAAGAGCTATCTCGACTGAATCAAATACAACACTTTTTTTTGTGCCTCTAAAAACTATTGATGGCACTTTCAATAGAATTAGCGGCACTTTTAAGTGCCGACAATCTGGGTACCTGACGTAGTGAGACATCCATAGACAGCTAAAATTTCAATGGTGTATTGTACAGATCAACACCAAACACTCTGAGATGAGTTCTTTTGTACACTACAAGACCACGGGCAAACATAAGAACCAAATTGTCGATGAGCAACGGCAAAGATAACTAGAACTTCGACGAACTCTATGAACGAGATTTCCAAGCAGCTACGTGTTTGTGGGTGGATTGATTTGCGTGTTAGCCCCAGCTGATGCGGCATCGGTTCCGGCATCGCTGGTCACCTCTGGCTCAGGGCTGTCAGTTAAATGTCCATTCTCGGAGGCCACAGTCCCAACACTCCCGATCGACTGCTCTCTCTCGATGCTCCCGGCTGCTTTCTCTCGATGCCTCCCCACTGACTTCTTCCGATGCCGCCTGACTGCCTTCGATCAATGCTGCTGATCCCGGAACTTGACCTTGAAGGTACGGCTAACAGCTGGCTGACAACATCGAAAATGCTGGGGCACTCTTTGATCAGCACTCC

>Fp-psGI.3 (FN376857)

CTGACATAGGCATCCCCAATGGGCCTGCCGAAGAAAGTACCCGGGGTTTGTTGAAGGCCCACGACCCGAAGTTTATGAAGCCCGGAAGCCCAGTTGTGGAGCAGTTTGGAAACATAGAGTTGTATTAGAAATAATGACTTGTACCTAATAGGGGTCGAACTCAAAGAGTCTCCCGGACTTTGTAACTTGTACATCACGAAACTCTCGGCTCCGCCTCCTATATAAGGGGGAGTCGAGGAACAAAGAAAGGATCGATTCCATTGTCAACAGAACCCTAGCTTTTTAGCAGTCGAGTACTTTTCCGGCTGAAACCCTCGAGATCTACTTGCCCTCTACTTCCGCTAAAACCCTAGTCTACAATCTGTAGGCATTGACAAGTTGATACCTTGTCATGTTCATACGGAAAACATATTTAATTTGACCACGCTGGCCACACCGTGCGAGAACTAAGCCGTCTATGGGTGGACGGCAGCCTGGCTTGATTCCCCTCCCACGCCCCTGTGCAATGGCAGCCGCCGCCGGCCGCCTCCACCGCTGCGTCTCGCCGTCCCAGATTCGCCCATATCTTCTCCCTCGACTCCTCGCCGCCGCGATGCTGCTCTCCTCCCTTGGTCGCCCCCACGGCAAACCCTAGACGTGCGCCTCGGGTTCACCGGCGCGGCCCCTCCCCACGCCTCCGCATGCTCCCTTCCGACAACTACGACCCCGCTCCAATCTCCGGTACCATCGCTACTCCAGCCCGACGCCTGCGACCACCTCCAGGATCCCCGCGTCGACCACGTTGCTCACCTACTCCTGCTTGCCTCCCTCCATGTCCCCGCATGGTTTGGCTTCTCCAAAATCCAGATCGCCAGTGTGGTGGTGGCGGTGGTGCTTCTGCTGCTATTTTCTTATGCGGAGCAGATTTGCTATTTTCTTACTTTTCTGAGAGTTTCGCTCTAATTTCTCGAGCATGATCTTAAACTACTAAGTATGTCTGCGTCAAATGGGAAGTGGGTTGATGGGTTCCAGTTCTCTTCACTATTTTGGCCCCCGCCACGCGATGCACAACAAAAATAGGTACCATACTGATAGTTGAATTACGTTATATATGTTGTAGCTTAGGAGACACCTGATGTTAACGGGACACTTCTGTCGTGTTCTTTGGGCTACGTTGAGTAATTTGGTCAGTTTACGTCTGACAGTGAGCAATTCCCGGAGGAATGCATTTTCGTCTGCAGAAAGAGTTTTCAGAGCAGCGGGCTTTGGCCTGTGGACAAATTCTTAGAGTTCTTACTCACTACAATAGGCCAATCTTCAAAGTTGCTGAATGTAACGACCACCTCTAGCCAGGCGACAACAAGTTATTCCTTACAGTAGAAAGCTAATAGCTCTCCAGGAAAGCTAATGTTGTCTAAAATTTCCTTTTTTCTTCTTCTTATGGAAGGTGACTATGATAGTCTTTGCTTTTTAAATTAGCTGATTACCCTCTGCTTCATTTCAAATACATCAGGTTTCCTTCCACTACTGGGTATTGTTGTTACTTCATCCCTAACTAAATTTATGCTTTGCTTTGATTCACTTTCTTCTGTATCATGTTTGTTCTAGTAATGAATCAAAGGTATTAAGGAACAAGCTAGGTGTTTGTGTGTATTGTTGTGCTATCTTGTAGCTACGAGTGAATTTTTTTGTTACATCTGTTCCTACAACATGGATGTTATTGCAGTCCCCATCAACTGGGTACAGTAATACTGACAGTTCTTTACCAGTTCACCATTCAAATACCTCTGAATGTTCTGTTTATTTTATTGCTTGCTTTCTGCTTACTTAAAATACAATAAGATTTCAGTCAAACAAGTACTGGAGTATTGCAAATTCAAATAACTGCACCAAATTCAAATAACTTCCTGATGCCAATGGTTGCAGATCACAAGCGTGGGGAGTATACATGCATTAAATGTGTTTTCATGTAAGCATGTCTATCTATGCAAGTGCATTCTGCCGTTCACTATGATAATACTTTCTAAGGAAAATAAAGGAGTATGAGAGTGCTTTTCAGAAGTAGTGTAATGGGGATGCACAACTGAACCTGCACATAGCTGCTCGTCACAACAGAACCATGTCATAAAAAACCTAGAATGTTTCAATATTCGGTATCGAAGTGTAGCCAAACACTGGTTGCGAAAACACATTATATTGGATCATGCAACATATATTTCCCAACTTTTCAAGTAATAAGTACAATACGCTGTCTTAACTCTTGATTTGCATGTCATCTGATGAACGGAACTGCGTTTTGAAACTGCAGGATGAGCAACAAGATGGAGGTTGAGGCTGCAGTGGAGGCAAGCGTCCAGTTCCTGAACAAGGCGGTGAAGCTGGATCTTTCTGTCTGAGGATTGATCATTTTCCCCTCTGAAATTTATTCAGAGAGCGCTTCAATGTTACAGCTATATACAAAACATAACTAGTGAACTAACTAGCTGGATAATCTCACTAAACCTAGTGAATTAATCTCACCATGTAATTCCAGTTCGAGAGATTGTTCTATAGGTTTCAGAGTAGAGAAAATGAATACAACTTATCAGTACTTTATAATGTGAAATTTAAGTTATAATATTGCTTAACACTGTCCCACATTCTACATAGCTTTTTGGATTTGTTGCCATTTCTAGTAGATTATTTCTCTGTATCGAGAATTATTTTGATCAAATAATTGCCATTTCTAGTTGCCATCCACACTCTTACTGCCATGGTACTGTCACATGATCTCTAATTTGATTTCGATTGTCATCTCCTTCCTCCCAGGCCGGCCATCATCTTCAGTTCTCAAATCCTTCAAGGTATGCTATCCTACTGTCCTACACCTCCGTTCCCCGTGTCTCACTTCTTGTTTTCTATCACGATAAACTACTAGAACTGCTGGGGCTGGGATTGGCAGTCTGTTTTCCACCCAGGCTAGAACTCCTGCATCCCTCAACCATATTCACATCACAGGTTAGATGAATGAGTCTGCTATTCCTTTGGCTGATTCCTTCTTCCTTTTTCATCTAATCACACCATACCACCTTTTCTTCTTCTTAGGGTTCTCTTGAATCCGTTTCAATTGGTGAAATAATACTAAGTTTCCGCAGGAGTGCTATTTGCTTGTTTTTAATTTCTGTATGATATAACATATAGTGTGTTTAGAGTTGTGTAGACAGTAACATATCTAGTGTGTTCCTGTTTACAGATGGATCTTCTCAAACCGAAAGGTAGGATTGAGAAGAAAAGTCCTTATTTGCAGGAGCTACAAGACCAAGTAAGGATATGACTTTACCTGTTTTTTCTCTGGTTATTTCAAATGAAGTATATGAGATGTTCCTTCTGTTGGTTAATAGGATAGTGTAGTTTTCAGATCACATGAAGCTTACTAGTAACTCCATGAGAACAGGTTTTAAATTATTTCGGAATCCTATTTTGTGCATGTTGTTTGTTGATGCCTTCCTTGCATCTTTGCTGTTTACTCCACTTTATTCTTCTACAACATCAGTTGTTGTTTCAGGCTGATAATTCTTGGTGGCTGCGGTGGTGCTAGTGCTGGTGATGTTGTTCAGGTGATCCTGCTATGGTCTCCCACACTTGGAAGAAGGGCGACATCTGCAATGTTTACATCGTCGCTGACTCGTCATTCGCGTGGAGATAGTTCCCATCCTCAAGCTCTATACGGTATACTCTATCAAATCCTAAATGGCTAAGATGTGCATGAAATTCCAACTTCATCTATAAATATGCACATATTGATATGGAGTGTATCCTATGTATTGTGGTTAGTCTAACCATTGTGGACCCAATAAGTTTGGGGACCAAGATGTACTTGAATGTTGTTTTAGGTACATTGGAGATGCAATGGAGGCTTGTCTCATCTATAAGGAAGGTGTTGTCACCATATGAGAATTGGAGCCAAGCTAGGATGATCGATGAACTCTTATCTACTACATCATGCCATTGCTATCTTCGTAACAAGTATCTTATTCATGCATACTCATATAGCATCCAACCTCTTGTAGGTTGCATCTTGGCATGAAATTCTTTATTTCGAAAATATTGCGGTTCTTGAAAAATCCTTTTTAAAGAAAACTTATTATTGTACATTTGAGTAATTTGAGAAGATGCATACGATGGGAATATATGTAAATCATGTTATGATTCACCTATCCCAAATATGCCTTCTAGCAATTTATTGCATATCAATTTCTCGAAGAGCTCTTATGTGCAATATTGATGAAATTGCGAAATGAATCCCTATTTGTGATACTTGTTGCCTTTCTCAAAACATTCCAACTAGCTTTACTTCCCTTATTGTTAGTTGAGATGTTTTTGAGATTTTCTTGGTTGAAGCTCATTCATATACCATAAGTGAAAATTGGAGCCATAGGCTATATATGCTTTTAAGCAAACATCCTTTGGTATATTTTATGACTTCACCTTGGATATCATGTCTTATTTATTTTATTAACCTCTTGTGTGTGCACGTTTCTTTATGGATCATCTTGTCCACTTTAGAAACTTATATACATGAGAGCGTTAATCCATCACCTTGACATCCTTGTTCTTTGCCGACTATCTTCTTATCCTTTTTGATTTTAGTGGTGTTGGTAAAGAAGATTCATGAGTGTTTGGTTTATTTTTATCTTCATGCACTCCTATCTCGTATTGTTGGACTCAAGTTGTTCTTGATAAGCATATTCTCATTATCTTGTTTATGTTCTAAATAATATAGAGGGAGTGAGGATTCCATGTTTGTGCATATAGTATTCAAATGCAAACATTCTAAATTATGCACGAACCTTGGGGAGCTTCCTTATTTCATTTAGAGCACTATATCTCTCTTATCATGACATCTTTGTTTATCCAATTTGATCTTTGATTGCTTGCTTTATTTGATTAAAGCTTTCTTCACCATGTTATCCTTGTGCAATCTTTGATCCTCAATATTGTTTGACTTCCTTCAAGTATTCGTCATTGGATATGTGCATTTGATTCCACTCAAATTATGAGAAGTGCACACCTTGAGGAGGAACTCACATTATATTGGCTTTATAAATTTTTCACCCATTTTGGCACTTGATGCCAATGGGGGAGAAGTTTAAAGGGTTTAAGGGAATGGTTTTATACTTAAATTTGGTTGTGCTTAAATGTTTTGGCTCTCATGCATTCCATATTTATATGTCTTGCATGGTTGTATATATATAGGGGAAA

Sequences from the *L. perenne* GeneThresher® library cited in this paper.

Figure 2

>FLPB002709C17-g0RSP_20020409 (FI167409 )

GCTTATCGATACCNGTTTTTGTATTTCACTGCGCTACTGCGCCCTTTTATGTTTGTCTACACAAGATCTCTAAATAATAAGTCAATAATGAAATATCAATGAAAATTGTCATTTTCTGGCATTTTGATGTGATAAAAATCATTTTTGCACTTAATAATGCTCCTGGGTGAAAGGACAATACGAGGACAACTTCCAGTAGCTTTAAGGGGCATCGGTATGGCGTGACTTGCCCATACCATCCGCCCGTACCACCCGTCAGAAGCACCGCCTCATCAAATATTTTCACCTCCCGCAGAAGGATCTGAGATACAAGCACGTACGTGACCAGAAACACCAGGACACCGCCCCAGAAGAGATCTGAAGAGAGATCAAGAAGGGACCTCATCCACACTACTACCCGGATCATTAGAGGACAAGGCATCATCACCTTCATCATCATCCACAACGAATCACCATCTCCATTCTCTCCATACCATTAAATTGTAATCTCAATTACAATTGTGGGTTTGTACTTGGATTCATACCGTTACCCTTTTTCCATACACAAGTTTATGATGATGTTCTTGATTGTCTCCATGTGTGAGTAGTTCATCCCATTCTTGGGGGAGATAGAGAAACCATAGTAGTAGTATTATATGAATTAAGATAATTTATACCTTTGCACTATGATTGTATGTTAGTTTTCTCTTGTAGTTTTGCATGATGTCCACCATGTAATATTTACC

>FLPB002048C23-g0RSP_20011109 (FI167410 )

GTGTAGTTATAGGATTTTTTTATTTTACCGCGCTACTGCGCCCTTTTATGCTTGTCTATACAGGATCTTAAAATAATAAGGCAATGATGAAATATCAATAAAAATTGCCATTTAATGGTATTTTGATGTGATAAAAATCATTTAAGCACTTAATTATGCGTTTGGGCGAAAGGAGGATCCGAAGACGAGTTCCAGAAGGTTTAGCGAACGTCGGTACGGGGAAGGCCCGCCCGTACCGTCCGCCCGTACGGCCTGCCAGGACGACCGCCTCGTCAAATACTCTCGCCTCCTGCAGACAGATCTGATATCCAAGCACGTACGCGACCAAAAACACCAGAACACCATCCCAGAAGAGATCTGAAGGAAGATCAAGAAGGAGCCTAATCCAAGTTGCTACCCGGATCATTGGAGGACAAGGCATCATCCACATTCATCTTCATCCACAACGAAATCATCATCTCCATCCCATCCATCTCACTAGATTGTAATCTACATTGTCATTGTGGGTTTATACTTGAATTCACACCGTTATTCCTATTCCATTCGCAAGATTATGATGATGTTCTTGATTGTCTCTATGTGTGAGTAGTCCACTCTATT

>FLPB002662H10-b0FSP_20020409 (FI167411 )

ATTGAATTAGAGGTATGAATTAGTATGAGTATAATTTTACTTGCATCATGATAATGATTTTATGGTCCTTCACACCAAAAAGCTTTGATAGGATATATGAAAATTATTTACAAAATATTTAATGATCATTCGTTCCTTCGCGTGAAAAAGCAAACCATAGAAAGAAAGAAAAGGAAAAATGAGAAATAAAGAAGGGAAGATAAGTGCAAAAATAAAAAGAGTTACTAGTGAGATTCATGAATAAAAGCTAACTTCATGATTTTATGATATTTAGATAGATTAATCTTCTTGTCTTTTATTAAACTTTGAAAGGTGTGGAGGCATTTAACTCATTTGAATTTGAGAATAGTATGATGTCATTCATGCTCTAGTTTTACATACGATGTTATAATCCTCGTTCTTGAAAAATGCCTTATTTGTTTTGACGACTCTCGTTTCATGAACAAACATGACTCTGTCCATTAAACTCCTATCTTGCTCGGGGAAGAGCGAGAGTCTAGCTTGGAGAAGTTGATAAGTGCATTTTTAGCATGTTTTTGCATTATTATTTTATCAAGATATCATCAAGTAGTGGTAGGATTTTTGTATTTCACCGCGCTATCGCACCCTTTTATGCTTGTCTACACAGGATCTCAAAATAATAAGTCAATAATGAAATATTAATGAAAACTGACATTTCCTGGCATTTTGACGTGATAGAATCATTTTTGCACTTAATAATGCACCTGGGCGANAGGACAATGCGAGGACAGCTTCCAGGAGCTTTAACAAGCATTGGTACGGGCAAGCCCCGCTCGTACCATCCGCCCGTA

>FLPB001026M06-g0RSP_20010815 (FI167412 )

CTCTATCCCTTCAAGCTTCTATCTTGCTCGGGGACCAGCAAGAGTCTAGCTTGGGGAAGTTGATGAGTGCATTTTTAGCATGTTTTGCATCGATATTTTTATCAAGATATCATCGAGTAGTGGTAGGACTTTTGTATTTCACCATGCTACCGAGCCCTTTTATGCTTGTCTACGCAAGATCTCAAAATAATAAGTCAATAACGAAATATCAATGAAAATTGTCATTTTTTGGCATTTTGACATGATAGAAATTATTTTTACACTTAATAATGCACCTGGGCGAAAGGACAGAC

>FLPB001057C01-g1RSP_20010815 (FI167413 )

TATCAAGTAGTGGTAGGATTTTTGTATTTCACCACGCTACCGCGCCCGTTTATGCTTGTTTACACATGATCTCAAAATAATAAGTCAATAATGAAATATCAATGAAAAATGTCATTTACTGGCATTTAACGTGATAGAAATCGTTTTTGCGCGCCTGGGTGAAAGGACAACGCGATGACAGCAGCTTCCAGGAGCTTTAACGGGCATCGGTACGGGCAAGGCCCGCCCGTACCACCCTCGAAGGGCAGAC

>FLPB001013B03-g0RSP_20010815 (FI167414 )

GGAATTGCGTGAAACACATCACGTCCGGTCTCCGCTACCCCGATTTTCCCGCTCCCTCTCTCTACCCGCTTATCGATTCCTTTACTTGTTGCATTGCATAGCATCATACTAGGATCATCATAGTTGCTAAAGTTTCCACCTTTACTTCCTCATTGCCTAAAATTGAAAAAGAACTAAAATTGGAGTAGGGACCATTCACCCCCTCTAGTCGCGCCTTGATCCATTCAACAGCGCTACCGCGCCCTTTTATGCTTGTGTACAAAGGATCTTGAAATAATAAGTCAATGATGAACTATCCATGAAAACTTTCATTTTCTGGCATTTTGACGTGATAGAAATCATTTTAGCACTTAATTATGCACTTGGGCGAAAGGACAATGCGAGGATAGCTTCAAGTAACTTTAACGTGCATCGATATGGGCAAGACCCGCCCGTACCGTCCGCCCGCACGGCCCGCCAGAAGCACCGCCTCGTCAAATACTTTCACCAGGCGCAGACGGCGTAGAGATCCGAGACACACAGCTCCGCAAAACAGAGAAACACCATCCCA

>FLPB002024D17-b0FSP_20010827 (FI167415 )

CCCTTCTCCTCAACTGTTGCACTGGTGGGGAATCACGTGGATTATTATCACCTTCACCATCACCATCATCATCATCATCATCATCATCATATGGATGTGCATGTCTTGTACTTGTACTGCCTTGGTCAGTTGCATCATCACTATGTTCAGCATCATCATTGCCCCATGATTATCATGCACTATGGTAGGGTTAACTGGATCCAAGACAACCTGTGGGGTGGTGGCCTGATGACTGCAGTTTTAGCATGTTTTTGCATTACTATTACATCAAGTTATCATCAAGTAGTGGTAGAATTTTTGTATTTCAACACGCTACCGCGCCATTTTATGCTTGTCTACACAGGATCCTAAAATAATAAGTCAATCATGAAATATCAATGAAAACTGTCATTTTCCGACATTTTGACGTGATAGAAATTATTTTGGTATTTAATAATGCGCATGGGCGAAAGGACAATGCAAGAACAAATTCCAGGGAGTTTAACGGGCATCGGTACGGGTAGAGACCGCCCGTACCGTCTGTCCGTACCACCCTCCAAGAGCACCGCCTCGTCAAATACTTTGACCTCC

>FLPB001091D09-b0FSP_20011203 (FI167416 )

CATTAAGTTTTCACTAAATAGTGGTAGGGATTTTGTATTTCACCGCACTACCGCGCTCTTTTATGCTTGTCTACACAGGATCTTACAATAATAAGTCAATAATAAAATATCAACAAAATCTATCATTTTCTAGCAGA

Figure 3

>FLPB002078I09-b0FSP_20010827 (FI167417 )

GTCCTCCCACCGCCGCATGCTTGCCAAATAGCGGCGAGCTATAATCAATTTCTAACGGTGTTTCACTGTCGTCCTGCTCGAGGCCGGCGTCCAAGAAGTTAATCTACATTGACCTGGGCCTGATGCGCGCGTCGGCGGAGGCGCTGGGCAAGTGCTTGGTTAGGATGTTGTACATGTCAAGATGTGGAGCAGCGTCCGAGTGCCAGAGAGCCGGAGTTCCGCCGCAATCGTCGCCTTGCAGCATGAGCTGGTGTCGCTCCCCCGTGTCTCCTACTACTACTCCGGCAAGGACCAGAAGCTCCTCGCTGCACGGCGGTAGTTGCAGTTGGCATCATTAATTCTAGCACTGTATATCATGTAGTAGTAGTTGTTCCGAACTTATCTTTGACCGTCGCTGCGTCGGCATCATGATGAATACTGGGTGGTGCCTGCATCTGTTTTTACTACTCTGTTTGCCTTGACACTAAAATGTGTTCATGTTTCGTCTCAACTAGTTGATGATTTCCTAG

>FLPB002029F17-g1RSP_20010827 (FI167418 )

GAAGTTGATCTACATTGACATGGACCTGATGTGCGCGTTGTCGGAGGTGGGGGGCGAGTGCTCCTTTAGGATGGTGTACAAGGAAGGATGGGGAGCAGCGTCCGAGTGCCCGTGAGCCAGAGTTCCGCCGCAATCGTCGCCTTGCAGCGAGCTGCGGTTGCTCCCCCGCGCCTCCTACTACTACTCCGGCAAGGGCGAGAAGCTCCTCGCTGCACGGAGTTAGTTGCAGTTGGCATCATTAATTCTAGTACTGTATATTGTGTAGTTGCTTCGAACTTATCTTTGATCGTGGCTGCGTCGGCATCAGGATGAATACTGGGTGGTGCCAGCATTTGTTATGTTCTTGTTTCTTACTCTGTTCTTGTTTGCCACTAAAATGTGTTCATGTTTCGTCTCAACTAGATGAGGATTCCTGGATATTATTTAGGTTCTTTGGTTGCATACTTGTGTTATCGAATCTGTCGGTACAGTTCTTGGAGGACAATTCAACCAAATTCAACTGAAAATTGCCAGGCCAGACACCATACACACTCTCTTTCTCAACATGAACGGACTGAATTTTGTTTCACCATTACAAATTATGGTTCTTTGGTTCATCGCCACT

>FLPB002289H22-b0FSP_20020409 (FI167419 )

TATATCCACATATATACTCAATTCATTGTTGAAAGTTTTCATGAAGTAGAAGAATCTCCCGCACACAACTATGCTCAGTGAACCACATACATCATTATGTATTTTTCCACTAAGTTTGTTGCCCGTTCAACTCTTGGCCTATGAACGGTATTTTAATCATTCTCCTTAGAAAAGATTTGCAAGCGCCAAATGATTCAAAAATCAAATGACTCCAAAAATCCATTTGCATGGAGTTCCTTCATGCGTTCCTTTCTAACATGACCTAAATGGCGGTTCCACAAATAAGTGGAATTCAAATCATTTGCCTTATGGCATTTTAGCGTCAGTGTTATGTATGTGTGTTTTCACAATTAAGATTTATAATAACTTATCCATCATACATGGAGTAATGTCATAATTTGAACAACTCATTGTTTTCATTTGACCAGAGCAAAATAACAATTATTAAGTTCTTTATTATAAATTCTAAGGGCTAGATAGAATGCCAACGACGAACATAATAACACTTTATTTTGTTCTAGACGTGCATTCCTATCATATTCCTTGTCAGTCACTTAGGCCATTGTATTCTTGTATTGCGTTGTTTTGTATGACACTTCATACCAACAAATATAGTACTAATACCCAAGAATTTCATAGTGTGACTTAACTAGGAATACAACCATAACATGTATATCATTTATATACACCTGAGCTAGACTTTCTAGTCTTTTCTTTTCTTTTTGCCAAAATATCTTTTGCAGTTTCTCTTTTAGCTTTCCTCATTATTCAGAAAA

>FLPB002413G09-b0FSP_20011203 (FI167420 )

GTCCTAAGTTTGTTGCCCGTTCAACTCTTGGCCTATGAACGGTATTTTAATCATTCTCTTTAGAAAAGATTTGCAAGCGCCAAATGATTCAAAAAATCAAATGACTCCAAAAATCCATTTGCATGGAGTTCCTTCATGCGTTCCTTTCTAACATGACCTAAATGGCGGTTCCACAAATAAGTGGAATTCAAATCATTTGCCTTATGGCATTTTAGCGTCAGTGTTATGTATGTGTGTTTCACCATTAAGATTTATAATAACGTATCCATCATACATGGAGTAATGTCATAATTTGAACAACTCATTGTTTTCATTTGACCAGAGCAAAATAACAATTATTAAGTTCTTTATTATAAATTATAAGGGCTAGATAGAATGCTAACGACGAACATAATAACACTTTATTTTGTTCCAGTCGTGCATTCCTATCATATTCATTGTCAGTCACTTAGGCCATTGTATTCTTGTATTGCGTTGTTTGTATGCCACTTCATACCAACCAATATAGTACTAATACCCAAGAATTTCATAGCGTGACTTAACTAGGAATACGACCATAACATGTATATTATTTATATACACCTGAGCTAGACTTTCTAGTATTTTCTTTTCTTTTTGCCAAAATATATTTGCAGTTTCTCTTTTAGCTTTCCTCATTATTCAGAAAAACACTTTAACATTATTAACTTCTA

>FLPB002264M19-g0RSP_20011109 (FI167421 )

TGTTTCACCATTAAAATTTATAATAACTTATCCATCGTTCATGGAGTAATGTCATAATTTGAACAACTCATTGTTTTTATTTGACAAGAGCAAAATAACAATTATTAAGTTCTTTATTATAAATTCTAAGGGCTAGATAGAATGCCAACGATGAACATAATAACACTTTATTTTTTTGTTCCAGACGTGCATTCCTATCATATTCCTTGTCAGTCACTTAGGCCATTGTATTCTTGTATTGCGTTGTTTTGTATGACACTTCATACCAACCAATATGGTACAAATACCCAAGAATTTCATTGTGTGACCAAACGAGGAATACATCCATAACATGTATATCATTGATATACACCTAAGCTAGACTTTCTAGTCTTTTCTTTTCTTTCTGCCAGAATATCTTTTGCAGTTTCTCTTTTAGCTTTCCTCATTATTCAGAAAAACACTTCAACATCAATAACTTCTAGGTTTGTTGGTCAAATACCAATAACCTTGAGGTTCTTACTTTGAAGTTGATCATCATATGACAAGTGTTCTAGATTTCACTTATTAGTAACTATGATGAACAATTTCACTCATAATTTTATCCATCAATTCATGACAACTTTTTGAGACCATGTCTGTACATGC

Hairpin types

Hairpin to 3’border regions identified by the SEEDTOP search are highlighted in yellow

Type 1

>FLPB002680K09-g0RSP_20020409 (FI167422 )

CGATCTGCTGTTAGAATTCATTCATTGATTCTGATTGATTGGATTAGACTGATCCATGTATAATTTTCAGTTGATTCAAGTAACTATATTTACAACTTTACCAAGCTACTGAAACAAGTTCTGGCTAAACCACCTATTATAATGATCTGTTTTGGTGCAATTAAGCCAGTGTTGCTAAGCATATGGTGACGTAACATGTTCTGCATCTACTGTCTTATGCATATTGCGACCACACCATTGATAGGGTTGACCTCCAGTGGATCTACCTTCTTCGAGATGGCCGACCCATATGGCACCGGCAGAAAGCCCAAGCAGCATCTGAAGCTGGCGCTTGTGATGGAGGCTCTGATCCCATCCACATCGAGCTTGCCTAGCACCCAGTTAGTGCCGAGGAACTTGAAAAATCAGCAAAGATGAAATTTCTGATTCTAGAAGGTTTTAAGTGTCAAAGGACAATTGTATTAATCATCTGGCACTTTTTCGAATCAGTGGAAGTAGTATATCTAAAACTATTTTCATGATTGTATATAAGTATTGTTGTTCCGTTTGTTTTTGTGTGGTTGGGTCTAATTTCACCTTATTAACTTTGTTTTCTAAATTCAATAAAATGCATTTCTATTTAGACGTAGATCGGTTCGAGTTTCACGGGATCGTGCGCCAAGGCGCACATCTAAATCTAGTTAAGCCTAGTGGGTAGGGTTTGACAAGGGTTTTTCTAATTGGGCC

>FLPB001057F14-g0RSP_20010815 (FI167423 )

TGACTATTATCCTCAATTTGTATAAATGCTTGATGCATTTTGGATGACGGGTCTTGAGAAAACCAGTGGTAACATATTCCTCTATTTATATGGCTGCATATAAGCGTTTGCTACTATGTTCGTGATTTTGTGTATTTTGTTTATCCTAATTTCATCCTATTGACTTCATTTTAAAAATTATTTAGCCACGTTATACATTGTGTAAATTGTCGCCAAATCCACGGGATCGTGCGCCAAGGCGCACATCTAAATCTAGTGTAATATGAGAACAGACGCAACACCAACACAAACGCAAGCGACAACCTACTAATACTACGAGAAAAAATAAAACAAATCTTATACCTTACAGCTCCAATCAAAGACAGGTCGATCTCTCCGACGACGGTTGCTTCTTCGTGCCCTGCTGCTGCCAGGACTTCCCCAAACTGTGACAAAAATATTAACAGTTGTAAGCTTCGAAGTTAGAAAAACTGATCCAAATCAGTGGCGGAGCTAGCTGGCCACAGGAGAGCCTGGGCAAGACCAGTGTGTCTGCCCGATTGATGTCTTGCCCAGTAAAATAAGCTTTTCCACATGCAAGTCTGCACTGTTTCCAGTGGATGCTAAGC

>FLPB001004G18-g0RSP_20010815 (FI167424 )

GACTATCTGTGCCAGTTTTGTACATATTAGAAAAAAAACTACAATCAAGACAAATTTAAACACATGTTATCTGATTCTTTATGAATATTTGAAATAGGGTTTTATCATCGATTTGTACAAATGCTAACACGGAGGTTTGTTAATTCTGGAGCTTTTGTAATTCTCAAAATATATTGCTTTAGTGAAATTTTTAGTGCAATTATATAAAAGTTATATAAAATGTATATTGGCAAGAGCTTCACGGGATCGTGCGCCAAGGCGCACATCTAAATCTAGTAACTTATTCTACACCCCGGTCGATCGGCCCAGCCAGCCTGATGTGCTTCGGTGCGGCTCGATTGGCCCAGCCAACCATCCGTGCTCACAGTAAGAAAAATACACCGCGATGTAATATTTTTGGGCGAGTAGTTCTTTTTTTATCGTGGGAGATCCAATTGTTTACTCCCCACTCAACTCAGTCCAGAAACCGCCCCCGCACGCCTCTCCTAGCTCGACCGGCGACCTCTGGTTCCCGCCGCCGCCGCGGAGATCCGGGCGACTCTGCACCTTTTCCTCATACTGTACGGCAATCCGAGCTCCCCTAGTCCACTTCCTTCATCTTCCTCGCTGGCTGTCTTGAATTCCAGTCAGCCTGCGCGGCATGCCCCGTG

>FLPB002684A23-g0RSP_20020409 (FI167425 )

AGCTTATCGATTCGTCTTGATGATGTTAGATGCCAACACTTTGTTGAAGCCTATGACTCTCAAGCCCCCATAAGTGACCTTCAACCTATCTACGCAGCAATCAAGAACTATTGTATGACATCGATCTACATCTTTTCCATGGATTTCTTGGCCACATTGATATGTTACTTACTGGTTCATTATTATTATTATTATTATTATTATTATTATTATTATTATTATTATTATTATTATTATGCATCCCAACTTACTATGGATCATATATATATCTATCTTCGGATGTTTGTCTTGTGATCCTGAGCTGTTAGTGTAATTTTAGAGCTCTGGGCTACCATAGAATTTCGACTATCAGATTTGGTCCGCTAAGGTACCGAGCCAATAACATATTATCATTTTCTCACCTGTTTTTCATCGTCGTTGTACAAACTGCAAATTGAAGTGAACTTCACGGGATCGTGCGCCAAGGCGCACATCTAAATCTAGTGAATGTAATAACTCATTGGCCATGCAACTGCCAATGGTGATCAATCAGATCTTTGGTGAGCTGGTGATGAGTCTCGGCATTTTTAATGCCTCGATGAGCTGCAAGAAAAGCTTCAATCCGATCAGGGTCATCCTCCAACATTGTCA

>FLPB001050B12-b0FSP_20010827 (FI167426 )

TGTATAAAACCTGAAAAACACATTTATAGTTTCCCACTTAATTTTTATCTTTTTTACAAATTCTAAATCAATATGAATTTCACGGGATCGTGCGCCAAGGCGCACATCTAAATCTAGTAAGGTCAATGACTCCTGCCGCAGCCGAGATGGTGGCAGATGAGACATGCATGTACAACTATTTGATCATTAATATTACAAGATCCAAAGTATTTTGGATCATTTGGTTTGCTAATTTTGGTCTAATTGTTTAGTTTTACCAAATAGATAGCCGAAACTAAACAATGTTGGATTTTAATTCATGCTGCAGGATAAGAAGTCTGTGATGTTACTTTGGCTCCATGAAAATTATTTTCTATGTTTTGTCCGTCTTAAACAGGCAACAACGAGAATGGCTTCAAAAGAAGCTAGCTGAATCAAGCACAACTTATTGATATCATAAACAGTGTGCCACGACCACAGCAGAACCCAGGTTGATTACATTTGCTCAGTTGATTAGTATTACTGCAGCAGTT

>FLPB002229C16-b0FSP_20011109 (FI167427 )

GTAGTAGTATAAGGTATGTATATAACAATACCTTATACCCAACAATCGATGGAGTAAGAAAGCTATCATCAAGAGCTTCATATGATTCTGGATCAATCAATTTGAACGCACCCACAAATCAAACAAATTCAAGAATTTGGATTCACGATGGAGTAATTGAAATGTAATGTTTGTCATTTCATGTGTTTTATCCTAATTTTATCCTATTGATTTGGTTTTGTAAAACCGGAAAACATATTACCATTTACCCACTCGATTTCATCGATATTATACAAATTATAAATTAGTGCGAATTTCACGGGATCGTGCGCCAAGGCGCACATCTAAATCTAGTAATCCATTAACTCCACAACTGTTGGCGTGGATGATGAAGATTGTTGCCATTGTGCAGCTAGCCGATCTGTCTCAAGTCTCTCAGGTCAGGACTGAATCAAAACACCTGCAGATCATCTTCAGGGTGCACTCAATCTGCATATCATGAAGCAGCAAATCA

>FLPB002403H20-g0RSP_20011203 (FI167428 )

AATATAAGAAATATGTTGCTTTCTCAATTAGAAGAGTTGTGCCAGAAGAATACATGCATCTACTAGCCATATTTCTCGAGACTTTAGTATATGTAAGTAACCATTATGTATGTATATGGCAGTGTGCTTTGCTCTACCTCAAGACTATCACTGATATGTATTTCCTTGTGCTCTCCAATATGTAACTTTTATGTATGCAAGAGCAATGTGCTTTTCTCTTACTATATATAAGACTACCATCGATATGTATTTCCCTGTGTTCCCTAGAGATGCATGTTTTTGATATGACATGTGAATAAATCTCAAAGATTCACATCTGCTTAAATAGTTGGTTGATCCCCCCGTTGATTTGCTGATTACATAGAAGGGATTTTGTATTACATGTCTTATCATGTCTGGTAGTTTCGTGTGCTTTATCCCACTTTTATTCTAATGACAATGTTTTGTAAAACCTAAGAAATGTTTTATTATAATGGTAGTTAAATTTTATTATTGTTACATAAAATGAACATAGACATAAACTACACGGGATCGTGCGCCAAGGCGCACATCTAAATCTAGTGTTGTTTAATGCAGTCACAGTCTGAAACTCCCGGGCGTGCTTCTGGATTAAGTGGCAGACTTCTCCCACTGTCCTCTCTCGCTCGCCTGCATTTACCTTGTTTCGAGCAGTCCAC

>FLPB001074G01-b0FSP_20011203 (FI167429 )

TCGACAGACTTCAGGGAGTGAGCGAGGGAGGAACAGGTGTAGGTTCGGGTAGGCCCGTGAGTATCCTAGAGCGTCCACCACAAGCGTCTGGTGTTGGAGAGGGAAAGATGGTACGGAGTATTTATAATTTTCCGTGTTTTATCCTAAGTTTATCCTATCGACTTCAATTTCTATTACCGAAGAAACATATTATCATTTTTTTCACGTAATTTTTACAATCGATATATAAAGTGTAAATCGACACGAGTTTCACGGGATCGTGCGCCAAGGCGCACATCTAAATCTAGTTTGGTAAGCAAGCTCAGGAGTAGCTCGATCGGTAGACTATCCAGACGTTTGGAGGCAGCGGGGCCAAATCGTTGGCCGTGCACTGCCACCTGTTCCTG

>FLPB002419I08-b0FSP_20011203 (FI167430 )

GGGCGAATGGGTACGGGCCCCCTCGAGGTCATGATATGTTGTGTCTTTGCTCCTCTTCATCAACCGGTAATTCTTTCCTTCATCGAGGAAATGAAAAGGGTTATTATTCACTCTTTATTTAATGCTTGCTTATTATGATGTTTACAAATGTACATACACTAAATATTAACATAAACTTCACGGGATCGTGCGCCAAGGCGCACATCTAAATCTAGTAGAAAGAAGAGGGGAGAGGTAGAAGGAAAAAAATGGGAAGAACGAAAGTAAAAGAGAGCGGATGGTGTCTGGACGAAAAGTCGAAAAGTCCAATCTATATCTTCCATCCTAGGCCACCGCCAAGCAACAAGTTACACTCTCACCTCTTTTTCACACAACACATCCAAACAGACACTCCTGTCCTTCAGCATTCCAAGTTTCCAACTCTCTTTCTCCCTCTCACTCTCCCCCCATCCGTGGACTCCTCTCCCCTCCTCTCCCCCAAGCAGCAAGAAATCCTCCAAGTTCCACCTCAATTTCGGCCATAACCCAACGAGCATCCGAATCAAGAAACCCCTTTTCAACTCCTTCTCGATCTCTAGCTAGCCACAAGTCAAGGGCAGCAGGCATCACACCAAACCACCTGCTTGTTTGCAAGTGCAAGAAGTAGAGGAGAGAGATCAATGGAGCTGTTCCCATCACAGCCAGACCTGCAGC

>FLPB002690G10-g0RSP_20020409 (FI167431 )

TTGGCTGCTGTCCTCCTATATACTTGCTAAGTGATGTCTATATATGTTATCTCATAACCATGTCTTAGTACCTACGAGTAATATATTTTCCTACTTGACACTTATAATTTGTAGTGTGTTTAATCCATGATATGTTGTGTTCTTTTGCTCCTCTTCATCAACCGGTAATTCTTTCCTTCATCGAGGAAATGAAAAGGGTTATTATTCACTCTTTATTTAATGCTTGCTTATCATTGATGTTTACAAATGTACATACAATAAATATTAACATAAACTTCACGGGATCGTGCGCCAAGGCGCACATCTAAATCTAGTATATGAATTGGAGGGAGTACTCCATATGAAAGAACTAATTCGAGCAAAATATGTACATTGCGCAACAGGTGCGATCACAGAACGCACAAGGACACTGATTTCGTGATACGATCTAGTACGTACATATGCATATGGACATGCATCCAGAGATCAGCCACAGCTGAACTAGCAAAGCTAAAGCACGATCCACCCACTGGTCAACATTTGCATCACCAATCCACCATTACGAGAGTACGTACATATGCAGATGAACATACATATGCCAGATGAACATGCATCCAGAGATCAAGCCACACACCTAAACTAGCAAAGCTAAAACACGATCCACCCACTGGTCAACATTTGCATCACCAATCCAGCAGTACGAGCTAGCTAACTGCTGAACGGGCAGCACCAAATCGTTAATCCACTACGTAATCTTT

>FLPB002111F11-b0FSP_20011109 (FI167432 )

GGTGGTAGCCACCTAACATTTAACAGGACATTAGGCAACGACTTAGGCTTTTGCATGTCTATGGTCCTCATGTATAAGAAACAATTGATATAGCATCCGATTTATATACTTAGTAGGTTTGTTAATTCTGGAGCTTCCGTGATTCTCAAAAATGTTGTTTTAGTGAATTTTTTATCGCTATTATATAAAAATGTACATTGTCAAAAACTTCACGGGATCGTGCGCCAAGGCGCACATCTAAATCTAGTAGAGAGTAATCGGTAGCTGTGAGCATGCGAATAGTGCAGCCACAACACAGTTCGTAAAATAGTAGGCAAGAAGATCATCAACAAAGAAATGAATTAA

>FLPB002715H12-g0RSP_20020409 (FI167433 )

CTTATCGATACCGTCCTATTTTGTTTTTTGAATGTGACGTGTTACCCCCTGTCTGATAGGTTACATTGATATGTTTGTAATTTCGTCTGGTTCCGGTAATTATGTTTATGTTGTTAATTGTCTTTGTTTTATCAGCTTATTATATATTGTACAAAACGTTCAATAATGCAAAAGTTAAGGGCTGCTTCGTACTGTGTATTTGATTTGCCTCACATTTAGCTGTTTTTGTAAAACCTAAAAATCGTATTGTCATTTTCTCACTTTATTTTATCATTATTATACAAAAATGTAAATTAGCGCGAATTTCACGGGATCGTGCGCCAAGGCGCACATCTAAATCTAGTAGTAATAGGTAGGGTATGGGCAAGGTAGGCGCGGCATGGTAGAGCAATACACCATACTCATACCTATATAATAGGTAGGTGAATATAAATTTCTATACATACATGTACATATATGTATTAAGTTTTACTCGTACCCACTAGTGGAGAAGAGGCCTTTGGTCCCAAGCAAATGTCCCAGTGCTGAACCAAACCGGGTCCAATGGGGGCATTGGTCCCGGTTCGTTTCTGCCCAGGACGACCCCACCCGCTGGC

>FLPB002680M13-b0FSP_20020409 (FI167434 )

ATGAATGATGTTGTTTTGGTGATGCCAAGCACGCCATAATAGAAAGATGACTCTCGATATCATAGTCTCAGACATTTCCTATCATATTCAGAAACCACTCCCTCCCTGCTATTCTGAGATCATTTTCTGGTGGCAACTCCCAGAACTTTCTTAACCCGTTGCGGAGGGCTCTCGCCAAAGTGCATCCTATCACAGCATGATGCTCATCTACTTTCTTTCCTTGGATCACTTCTTTGGATGTTTAGATACATAATCTCACGTGAGAAGGAAAAATATTAATGTTTGTGAGAGAAGAAAAGGGTTGAGTGAGAAGGATACCAAAATATATCAAATTCTATGGGATCCTGGCCAAGGGCAACCACCACCGAAGCAACGAGACCAACATCAAAACAAATAATTCAGTCTCATATTCGGGAGATCCAAAAATGTTCCAGCTTACATCATAAGAATATTTTTTTTTTGCTTTCAACAAATAAGAGTGAGATTCAAGAGTTATGCAAATTTTTATCTTAATTGCTTCCAGTAAAATTTGAAAAATGTATTATCATATTGGCAACTATTTTTTTCATTGTGAATATTGGCATGAGCTGCACGGGGTCGTGCGCCAAGGCGCACATCTAAATCTAGTAGTAATAATACAAGGAATATGACCAGAGGTTGGTCTTCCTTGTGGAGTGACTAAAGTGTCAGCTTAAGAAGTTGTGCAATGCAAAGAGGAAAATAACCAGTCTAATTTCTCCAACAATGGATTGTCCTGCAAATTACTCCAAGTGAACGTCCTTCCT

>FLPB002408J10-b0FSP_20011203 (FI167435 )

GTCTTAATGTTTCGTCTGAATTATATGTAAATAATGGAACAACTCTCTGCGAACCCAGATGAGATGCCTCCGTGTTATTTCGGAACCATGTATTAGTATATGTATGTAATATATTTTCCCTGTTTTCTCCTCTTCATCAGCTGATAATTTTTCTTTGTATCCAGAAAATGAAATTGATAACTATTCTGCTAAATGTTGTCGTCTGGTTTGCACATCTTATATAAAAAATGAGGTCTATGTGAATATTTCCCTTGTTTGAATATTGGTTTGTTACATTAATCTTGCTCCCTGCTTGTGTTTGGTGTAGTACAAAAGATATGTGCATGAAGCGAGCTTTTTCCTGCAACAACATATGTGATTGCAATGACAATACAACATCGAACAAGGGGTAGAAGCTTCCCTATGATTGTTCATACCTATATTATTAGTTATGTACCTGGAAGCAAAACCATGAACAATAGTAAGATTATGTTATGCTTATTTGGCCATCGGATTTGAATGCTATTGCAATGTTGTCTTTCGGTGTGTCATTCTTTCGTGCTCGGTTGTTTATGCACTTCTTTTTGAAACACCTTAGTCCAATGTGTACATCTATAAGAAATGAACATAAGCTTCACGGGGTCGTGCGCCAAGGCGCACATCTAAATCTAGTACTAATGTAAAGCATATGAGATGAATGCAGCGAATTCAAGGCAAATGATCAAGACAATGATTAAACAATTGAATCATATAGCAAAGACTTTTCAT

>FLPB002197G24-b0FSP_20011109 (FI167436 )

TGATTTTTGGAGAATAACTGGCAACTATTCAGCGTCTATACAGCGACTAAAAAACCATGCTTCTTCCAATAACTAAGAGTATGAGATTCAGAATTTTATGCAATTTTTATGTTAACTGCACATAGTAAAATCTTAGAAATTTATTATCATATTGGCAGCTATTTTTACCACTGCTATAGAAAATAAATATTGGCATGAGATCCACGGGGTCGTGCGCCAAGGCGCACATCTAAATCTAGTTAAGCTAATGTCTGCTAGTTGCTCGAGCCTCTCCCTGCAGAACCAAGATAACTCTCAGGGGATAGTGATACACACGTCCCATATCTCTTTTTGTTCTGTCCTGGGTTCAGTTTCAGAACACCGGCTCGTGTTCAGCTTCAGATAGCACCATTCAAAATTCATTTATCGCCTTCTGTTGCAATACATGGGTTCTTCCTTGTTGTTTTTCAAAACCACAGAGCCTCCCCACTTGTTAGACCCTGGCATGTTGTTCTGGGATACCTAGGATCGATTTTCTGTCGCAGATTTTTTAGTGTGCCCAAAATTGTAGTAGCTTCGATTGAAGCCAAAGCTAGCGACC

>FLPB002346M05-g0RSP_20020513 (FI167437 )

CGGTGAGAGCTGAACACGGGTTTATGTGCCAAAGCGCACCACTAAATCTGTCATCGCTATGACGGCTTTGTTCTGCTTCAACTTTACCCGCGCAAGGACGGCATCATTGTTTGCTTTCTCTTGAAGATAAGGGGTAGACGCTTCTACATGGCGGTTATGTCCAGCAGACGTCCCACCGCCACCGCGTTCATCGCTTTTGATGCGGTTTTTGCAACCCTCGTGTCGTCGTCACCAATGCTTTACCACTTCATCTCCGGCCGGATACGAGAGTGTGTGCAGAGGGAGTAGGACGACTGGTTTTCGCTAGATTGGAAAAAAAAGTAGGGGAAGGGGAGCATGAGGGAAGCGATGCGGTCGACAAGAGAGCGATGCGGTTGAATGTATTTTGCTACTACTCTGTTGGTGATTTTGTTTGTTTTATCCTAATATATCTTCATATTCACTTTGTTTTTTAAAAGCTAAAACACGTATTAATATTGGTGTGAAATTCACGGGGTCGTGCGCCAAGGCGCACATCTAAATCTAGTTCCAAAATAAGCTTGCTGGCCCGGCCGGCCATGTTAGCAATTTGTTAGTTTCTTCTTTCTTTCTTCCGCGGCGTCGCGGCTG

>FLPB002086H24-b0FSP_20011109 (FI167438 )

ATTTGATGTTGTCAGTGGTGTTAGCATTTAGTGGTGCACCTACGTATTAGATTTATGTTTTCTATGTGTTGTGGCCTCTAAAGGTCGAACTGCAACAAGCTTATGTAGATGTTATAACTTTTGTACCTTCTATTGATGTCAAAAGGAAACACATAATGTTAAGTTATTGTAGTATATTTAGCTATTTCCCTGATTGTGTATAAGCATTGTTGTTCTTTTGCGAATTTATGTGTTTATTTATAATTGCACTCTATTAATTTTGTTTTCTAAATCCTCTAAAACATATTTTTATGTCAATGTGGATCTGTTCGAACTTCACGGGATCGTGCGCCAAGGCGCACATCTAAATCTAGTGACTGTACCTCTAACTGTATAGCCCGCTGCTCTGAGGGCGTACGCGGATTTTGTGTATGACGTCTTATTTACTGCTGGAATATAGAACCTAGTTACTGAACATAAAACATTTATAATTTGTAGATTTTTTTTTCATTTTGTGTAGTGTTTTTGGTGCATTGTAGTACAACCATCATGGTTCATTTTGTGTGTGTGTGTTTTTTTTGTGGCTTCTTTTTCAGTGGATGTTCCTTTCGAGATTTTGTGCTGGAATGTCAGAGGTCTGAACGA

>FLPB002056C19-g1RSP_20011109 (FI167439 )

ATACGTCTTCTATTGACTTTTTTCTGAAAGCTAATAACAATATCATTTTCACCTAAATTTAGTAGTGTTACACAAAGTGTGCATCGGCATGAGTTGCACGGGATCGTGCGCCAAGGCGCACATCTAAATCTAGTTTTGTGTAGATTACAGCACCAGTAGCCAACCTGGCACTTAGTTATCACGAGTATTTTCTTTGTCAAAGTAGATCATTCCCAAATTAAGGTTAGTCACTGTCTTGAAATTTGGTAAGAGCAGAGATCTCATATATGGTTAGGTTCTGGACTGGAATGTGTAACACATGACCTGTCTTCATGTAACCTAAGCTGCTTAAAATATATCCTATTTTACTTGAAAAAACGTTGCCCCACGGGCACATACATTCATGACGATGACAACGTCTTCTCCCCCTAATTAATTTGAGGCACCAACATGTCACTATACAAAATAGAGAGGTCAAAGTCATATAACAACCTCATGCATGCAAGACGAAGGGCGCATCCAACCATGCATCAATAAGAGCAAATACCTGCAGTGTTTGATGAACCCTAGTAAAATTTTCAGTATAAGAGCTAGCATCTCTAACAATAACCGAAAAATTCGGAACTTAAAAAGCTCTATTCAGTCTCCCGTAAACATGGCTTACGATGATTTTGCACTCGCGCAGAATA

>FLPB002713K07-b0FSP_20020409 (FI167440 )

GAGCTCTCGAATTTCGACTATCAGATTTGCTCCACTAAGATACCGAGCCAATAACATATTATTATTTTCTCACATGTTTTTCATTGTTGTTGTACAAACTGCGAACTGAAGTGAACTTCACGGGATCGTGCGCCAAGGCGCACATCTAAATCTAGTTGATAAGGTTGCTTAGTACTGCCCCCGACCATTGAGAACTATCAACCCTAGCGTTGTATACCCCTCCCGCACTCATGCAATTTGAGCTCTTTTACAAATTATGAAATAACTTTACTACACTAATATTGCATCAATATGTTAGTGAACAAGTTTTAGAGCTAATTTGATATATGAAAATTGAGAAACAAAAATGAATTTTTTTTGTATGATGAGATTTTCAGATTTGGGATGTACAGTGGGGCCTGGACTGGAGTATCCTGCCTGGCCTCGCAAACCCTAACACCCAGCCTCGCGAGCCATCGCCATGGCTGCCACCCTCCCCAGCTGGATGATGCTAGAGCGCTACGTTTTCCGGAGGGACGACCCAGAGTCCTTCCCCGGCGACGAGGCGGCGCCCTTCACGGCCTCCTCCTGCACCTCCCAGGGCGACCCTTTCCGCGTCGCCTTCCTCATCGCCGCGCCGCCGGCCATCTCCCGCCTCTACGTGCAGTGACCGGGCCCGGGCGGCCTCGACCCCGCAATCGGCATCGCGTGCGACCTCGTGGCGGCCCATCGTGACCTCGTCCTCCTCAGGCTCAACTGCAATCCTGTAACGCTGGAGGATTTTCCCCGCTCCCGCATCTGCAG

>FLPB002012O18-g0RSP_20011203 (FI167441 )

TAAAGAAATGCATCATCATATTCCACCAAAATTTTAGCAGTGTTAATACAAAGTGTGCACCGACACGAATTTCACGGGATCGTGCGCCAAGGCGCACATCTAAATCTAGTTAGTATTATTAGTACTATACTAAACCTGAACGTTAAAGTTTTACCACGATTTTCGGACTTAATTTTCACCATATTTCTTGCGCTGACACGTGTTAGTCGCTGTTAAATTGAGCTGCTGTGTGTGTTTTGGGGGACGCGTTGCTATTTCCCACGATTTCTTCTCCTCTCATTTCTTGCTTTCGTGTGTTCTGTCGTGTGGCTGTAGTGTTGCTCTGTTACTGTGCTTGGTGGGGTCAGCCTGGGACGTGGAAGGGATCCGCCACCGCTTCACCGATTCTCCGCCCGGTGTCTTCGTTATTTTTTCTCCTCTCTTATATTTCTTCGGCTCTCTTTTCTGCCGCCGGGTTCTCCGCTCTCCCCCTCCTCCGGTTTGCCTGCAATGGTGAGGGGTCTGGACGCCGATCTTGTGCGCCC

>FLPB002138G23-b0FSP_20011109 (FI167442 )

GCACTCCACGTTTTCTTACATGGTTCAACTTCACGAGATAATGACTTAGAACTGGGTAAGTCAAATTTACATATATTATCATTTTTTCATCTGATTTTCCTTTTGTTATACAAGATGTGAAATCCACGGGGTCGTGCGCCAAGGCGCACATCTAAATCTAGTCATATATATATAGTGGCTGGATTGGCGTCAAACGTCGAGCAGACGTTCTGTACTCTGGCATACACCAATGTTTGCTCTCTTCATCTGTACGCATTTGGAAATGGCTTCGTTAATGCCGCGACAGCTGACACGGCCACAAATGGCGGCATGAAACAGCACTGAACAAAGAAAGTGGGAAGATCACCAAACGCACCCCGCCCTGCCCTATCCAAGATTCCAGGTCTAATCACAATCCGTTTCTGGGAATATATATTTGGCTAGGATTTTCACGGTTAGCTCAGCAGTACCCTACATTATTCAACATTATGTAACATAAGCATGTTACTGTTGCAAGTGGAC

>FLPB001031B13-b1FSP_20011029 (FI167443 )

AGTGACATAGGTTGAATTCACATGAAATAAGATCCTGAACTTTTATTTATGGCAACTAGACAATCCAAAGCACAAGCTATATAAATACATATTATAAGGCAAGAAAGCACAAATGTTCTTTATGTCTAAGGTAAGGTTGATACATATCGTTTCACCCATTGGATAGATTTTTCATTTGATTTCTGCTAAATATAGTGTTTTACAAACATTTTACCAGAGCCAAGGCGCGCACCAAAATATTCCTCTATTTTCCTGAATATATATAAGCATGTGCTACTCTGTTCGTGATTTTCTTTGTTTTAACCTAATTTCATGATATCCACTTTGTTTTGTAAAAGCTAAAAAAATGAATTAATATTTTACATTGGATTTTTATTGTCATTATACCAAATGTAAATCGGTGCGGATTTCACGGGATCGTGCGCCAAGGCGCACATCTAAATCTAGTTGCTCTAATTATCACCTCCTCTTGCGAAACTCGGAGAATGAGGGAAACCCACATGGAATTTTTTAGAATTTTTTTCTCTCTAATTCCAGAAATAATACGGCCTATTTTCATTCTTTTTTCAGAACTAGGAAATTTGCCCGTGCGTTGCAGAC

>FLPA001034D06-g0RSP_20010509 (FI167444 )

CTCCTATGTTTCCCTGTTATATATAAATGTTCCCGATTTTGTTTGGAATTTTGTTTGCCTTATCCTAAGTTCATCCTATCGACTTCGTTTTCTGAAACCTAAGAAATGTATTATTATTTTTGCACTTAATTTTTATGGGTGCTATACGAAATGTGTATTCTTATTAGATTCACGGGGTCGTGCGCCAAGGCGCACATCTAAATCTAGTACATATGAATAGCAAAAGCGCCATGCCATCGCTACCCCATACTACTCAAGGAGCGAGAGAGAATAAGGTGAACTGTGACTTTTTTGGAGCGCGACGCCATGATGTTTGATGTGCTGCAGCCATCGCAAAAGTCCAATGAAAGATTGACACGCCATGCTGTTTGATGTGCTGCAGCCATCGCGAAAGTCCAATGAAAGATTGACACACCCTGGCAAGCAGGTACGTACAAGGGATGATAGCTTGCCGCCCTGTACCGTAGCGGGTCAGCCAG

>FLPB002711L12-b0FSP_20020409 (FI167445 )

ACGACATGGAGGAGTACCCAAAACACATTATATGTTGTGTTCTTTTGCTCCTCTTCGTCAAATGGACAATTATTTACTCTTCATTTAATGCTTGCTTCTCATTGATATTTACAAATGTATATAAAATAAACACTGCCATAAACTTCACGGGATCGTGCGCCAAGGCGCACATCAAAATCTAGTTAGTTTAATATCTGCATAGTTAGGCCTTATGTTGGGGTCGGAGGATCCAGTGGTGCGTTAGATGCTGTTTACCGTCCCTGCGAGGGATGTTCCGGGGATCAACGCTATGTTGGTTTTTAGGCCTCTCGTAGGGGTAGTTTGCATCGTCTCTCGTAGCTGCTAGGCCCGATCGCACTTAGGACGTTCCGATTATGCGGTGAAAACCCTAAACTGTCGTGGATCGCTTTAGCTTTGTCCTGATCAAGCAGGATCCCCATGCCATTGTAAATCCAACGTGAAGCATGGGGCGATCGGCTCCTTGAGCCGATCCACAGGGAAACCTGAGAGCCGATAGGGCTCGTATTTAATGTTTACGTGTCTACCATGCAGGAACAATCGAAGCACTAACACCTTCCTGATCGGGTCTAGGTCAGGTGGCACGCCCTAGCAACCGCCAGGACGTTGGCCAGAAGGA

>FLPB002197B07-g0RSP_20011203 (FI167446 )

TCGGTGATATGCAGTGGTCCTGGACATCATTGCAGTCGTCGAGGACTCTGATGCAGCCGATAATTTCGGCTTGAGAGAAGGAGACTGGGGAGAGGAATGGTAGGGGGTGTTCGGTGTTGTTTGGGTGGTTTTCACACAATCGCCCAATGAGTGCGCCAAGAGGTAGGCATGTTTATATAAAAATAGTTATGGCACACCGGTCCCTGGAGTTGAATCAGATGGTTGTTGATGGAGGAATGCGGGGATGCACGAAAGCAGGTGGATTCATAGGATACGACGACTATTTTCATTTGGTAAATGAAAAAAAACATGTTATCATTTACCCACGTCATTTTCATCACTGTTATACAGACCACTAATCAATGCGAATTTCACGGGATCGTGCGCCAAGGCGCACATTTAAATCTAGTTCCATTAAGGACTGCCCTAGCTCCATCGTTATGTAACAGACCGCTAACAATAAGCACATCACATACTTGATCCCCC

>FLPB002430L13-g0RSP_20011203 (FI167447 )

GTATTATAAACACATGTCGTGATACTACTAATACACATAATATTTTACCCAGTGATTGACTATGTGAAAGTTTGGGTGGTGTGTGGATTGATAAGACTTACTTCTCACTATTACACCATCGGTTCATATAAATGTTTAATACATTCCTCTATTTTTGTGGTAGTATATAAGAGTTTGCTACTTCGTGATTTTGTTATCTCAATTTCACCTTGATTGACTTTGTTTTGTAAAAGTTAAAAATATATTATCATTTAGCTACGTGATTTTTATCATTATTATACTATCTGTAATTTGGCCCTAATTTCACGGGATCGTGCGCCAAGGCGCACATTTAAATCTAGTCTTCTTTCAAGCGGGTCCTTGTACTTGAGCCAGGGGTTCTCAATGCCGGCATTTACATACGCCTCGTCCTCCTTCGCCCAATAGGGCTCCTTTCCCTCGTACCCACGGCTCCCAAGGTTGTGGTTGCCGATGTTAAGCTCCCGCATTTCCTTCCCCCACAACTTGCGATTCTTGGTTGCTTGTAGCTCTTCATTGGCCACAAAAGCATCAAAGTCCGCCTGGGTGACATGCGGAAAGGCGGAGTGGACCTCTTCGAAACCTTTGCCCTCAGCAATCAGCTTCCGCACCATGTACTTATAGCTGCTGAGGTGTTGGTGAACTTCAGGAGGG

>FLPB002665J10-b0FSP_20020409 (FI167448 )

AATGAGGATGAGCTACTGTTCCTAAAATTAGAACCAAGGTTGCATCTATAAAATTGATTTGTACTCATGTTGTGTTATTGTGTATGGTTGTTTATAGCCTTTTCAGTTCAGCTATTTTGTTTCTTTTAAACAGCTTTTGACACGCAATGTGTTATCACCCCTTGTCTGATTAGGTTACATTGATATGTTTGTAATAATTATGTTCATGGTGTTGTCCTTTTTTCTTAGCTTAGTATATATGTCATACAAAACATTCATTAATGCCAAAGGCCAGGGCTGCTTCTTCCTGTGTATTTGATTTGGCTCACATTTAGTTGGTAGTTAATTTACCTTTCATTTGCAGTGGTGTTGAGTTTTGGTGTGGCGAGTACATATTAGTTCCAGGTGTGTTGTGACCTCTGAAGACCTTCCTTCGGTGACGACCTTCCTTCGATTGCTCTTGATAGACCAAGAATCTTTGTTAATTTAATATATTTCCCCATTTTTCTGATTGTATATAAGTGTTTGCTATTCTGAGAGTGATTTCTTCTGCTTTATTCCTTTTAAGAAACTTTCACTTCCTTTTGTAGAGCCTAAAAAACATATTATGCACTTCATTTTTTATCATTGTTATATAAAAAAAAACTGACACGTACTTCACGGGATCGTGCGCCAAGGCGCACATTTAAATCTAGTGAAAGCAAAGCAAGGGGGAGTAACTGGTATGGCCGGAGTTGGGTAAAAG

>FLPB002417J02-g0RSP_20011203 (FI167449 )

ATCTAAGAGTTTTGGTAGAAGGACTTGGTTGTTCTCTTAGCCTGCTATATAACATTATCTACAATAATGTTCACCGCCGCTAAACATGTTGTGGTTCAATTCGAATTTCACGGGATCGTGCGCCAAGGCGCACGTTTAAATCTAGTAATGGAACCAGTATGGCCCCTTCACAGCGATTTGAGCTTGCTGGCCGTCCGATCGGCAAATCTAACGGAGCAGATCGTCGTTCCCGATCCGAATGGGCTTTTTCTTTCCCGAACCTGCGTCCAATCCCGTTAAAAAGGCCGTTCTGTCCTCCTATAGGCCCATTCTCGTTCTAGGGCTGCTACTCCCAAAATTAAGTTAGATGGCATCACAAAACTTGGGCCCCGAAATGGTTTATAGGCCTATACAATTGGGCTTCAAACATACCGGTTGTTCTCTTTCCCCTGCGACTGGGACTCGTTGTCTCCTCCGTCCCTAGATCGTCCTAAGCCTGCGCCTCTGTTCTTCCTCCGCCTGCGAGTCTGCGACGCGTGCTACCAGAGCAGGATGAACACCGAAAGTAGGTACTTAATTCACGATCTTTCGCTTTAATTTCAGCCGCAAATTTCACGTATTTCCGCTTCAATTTCAACCGGATTTCTAGATTAGCGTTGACCAAATATGCTTCTCGTCCGGCTTCGCCGCCGGCCAACCTTGCATCCCGTTCAGGCCGTCGAGTGGGCTCCATCACGGAATCGGAAAAGGGAACCAAGCCATCCGGATCGGAAGACCGGTTCGCCTTCTACACG

>FLPB001019J08-g0RSP_20010815 (FI167450 )

CCAAGACACCCGCCACCACCGGCACCGCTTCGGATATGTAACTTTTATGTATGTAAGAGAAATATTCTTTGCTCTTACTATAAGACAGACTACCATCGGTATGTATTTCCCTGTGTTTCCTAGAGATGCGTGTTTTTGACATGTGAAATTAATCTCAAAGATTCGCATCTGCTTAAATAGTTGGTTAATCCACCGTTGATTTTCTGATTACATAGCAGGGATTTTGTATTACATGTCTTATGATGTCTGTTAGTTTTGTGTGGTTTACCTCAATTTCATTCTAACGACACTGTTTTGTAAAACCTAAGAAATATTTTGTCATATTGGTAGTTAAATTTTAATATTGTTACATAAAATGAACATAGACATGGACTACACGGGATCGTGCGCCAAGGCGCACATCGAAATCTAGTATACTTGAATGATTGGGTCGTCCATGCATATTCTGCGAAAGCTGCGAAAGCTACATACTCAAGTACGTTTCAGAGGATGGATGTTATTTTTCATCATCCAGACAAATAAATATAGATGGTCCTCTTTTACGGTCACGGATGGTTCCTAAAAATGACAACCACTATAAAACTTGTAATATCGATGATAATT

>FLPB002439I09-g0RSP_20011203 (FI167451 )

GGTAGCTTTGCAGGACAATCCTAGGGGAAGAGATCCTGAAGATCCATGTCAATCCGGGATGGAAGAAGGGCATGAAGATGAACGAGCATCGACACACAATCACTGCGGACCTGGTGTTCAGTGTTCACACAGGATGGCAATGGAATGGTGGTGATATAAGAGGTGCTCCTCACATGCCCTATTAGTACACACTATAGACATTGGACATGTGCAACCTGACGGTACCTGATGCCTATCGGATAATTATTCACTCTTTACTTAATGCTAATTATCATCGATGTTTCCAATTGTAGTATATAAAATAAACATTGACACAAACTTCACGGGATCGTGCGCCAAGGCGCACAATAAAATCTAGTTGGGATGGAGTGGGCTGCACCATGCACCTTCCTCAGCGGAACCTACCTAAAATCGACTTGAGAGCTACACAAGCTAAACACAAACCTGACTTGAAGCTCGGCGGACTTTTTGAATCAAGCTCGAGTCGCTTGACAAAGCTTTGCTTGGGAAAACTGCAAGTGTGATTCACATTTATCCGTGGACATCTCTGTTGGTTTGGCATAGTAGTGCGCTAGCTTGCGTAGTGCGTAGCTATGCCAAACCCGCGCGAGACCACGCTCCAGATAGATAGAAGCAAAGCCATGCGCCACGTGGGCAGGAAGCAGCAATTCGGAGGTCGTCCCTCGAGTAGCTAGCCAGCAGTAGAGCCCGAGACCACTCCAATAGACCCATTCA

>FLPB002507C14-b0FSP_20020220 (FI167452 )

GTCCTTTTTAGAATCTGGCTTCAGCAAGGGTGACTGATTCCTCAAATATTTGGGAAGTGTGCTCGATGATGAATTAATATATGTTATAAATGGTTGTGCATGCTCGATGATGAATTAAACTATGTTATAGATGGTTGTGCCTTATCTATGATCTTGGGTTGAAAATGAGAACAATCATGCTGAGTATTCCCCCCATAGCCCACATTTGCAATTTCCTCTGATTCACTACATTTTGTAAGTCCTAATAAAAGTATAATCGCTTTTCCACCTATATACGAATTTCACGGGATCGTGCGCCAAGGCGCACATCAAAATCTAGTAAAAATAAAAGATGGGTTCCATCACCTAGGAAAACTCACAAAGCCTGAAAGAGCAAATCGATCGATAGATTGAATTCGTGCAACGTCCATCTCGGACCGGAGTTTCTCACTTTCAAAAGTTGAAAGAGTAGCCACCTTAATTCGCGCCCTTTCCAGAAAGAATGGAGAAAACTGAGAAGGCCGTGCTTTATTAGCCTGAAAAAACAATCGGAGCAGACACTTTGAGACTTTTGGAGCAAGCTCGCAATAGCGATAGGTAAAGAAGTGAAAGAAACACACATGGTTGGGTCGTTACGTGTACTCCATCAAATCAAATGGGGGCCATCGTGCCATACCTACTGTTGCTGTTCAACGGGTTCGATCGGGGTCGTTTTCTTGTTATTTGGACGGTATCGATAAGCTTGATATCGAATTCCTGCAGCCCGGGG

>FLPB002676D20-g0RSP_20020409 (FI167453 )

AATAATATTTTTTCCACTCCATGGTTGCGGCTTGATGGGAACCATCGGGATCCCAGCCATTGTCACAATGGAACCTATGCTGAAGAAGCATCACCATCCAAGCTCAAAAGCCATGCTATTTTTGCCACTTATATGTACTGCATGTATCAGGCATAGATCGCTTTTTACTATATTTTGGCTGCACACTGCCACTGCGCTCCTTATACTTTTGCATCTATTTATATGTTTGTTTTTTTGCTACAACTTTCAAACTGCGAAACCTAATAAGTGTATAAATTTATCACTGTTATATAAAATGTATAATGGCATGAGCTTCACGGGGTCGTGCGCCAAGGCGCACATGTAAATCTAGTGAAGTTCAGGCGTAAGAAGGTACGCATGTCACGGAAGAGAACTCCTTCAGGTGCACGGTCGTATATTCTCTACTTTGTAGTGCCTTGGCGAGGATGGGCGAGTCAGTCTCGAGCTGGACCTGTCCCATGCCCCATGGAGCTGCCTTCAGAACACAGCAACAGATTCTGCATGCACTGCACTGGCCGCATAGTTTAATCTCCCTGCACGGCTGCACCCAACTCTCAAATGCGATTGCTTATTTGATGCTGTAATCCAGTAATTATTAGGGAAAGCTATAATGATAAAAACGTTCGCATTTTAGTGTACGACTTGACAGCCGTCCGACTTTTTACCCGTGGATGGAACACTACTGGA

>FLPB002107J17-b0FSP_20011109 (FI167454 )

GGAGGACTACCCAAGAAAGCAACACACTGATGCAGAACACATAATATGTTGTGTTCTTAATTTTCTCCTCTTTGTCAAACTAATAATTATTCACCCTTCATTTAAATGATTGCTTACATCGATATTTACAAAATAAAGACTCGCATAAACTTCACGGGGTCGTGCGCCAAGGCGCACATCAAAATCTAGTGTGTTCAATCGGCACACCGGCGACCATGCTTCTTGCGCGAATTTTTTTTAAAATAATACTCATTTTTTATTATTAATTTTAGAAATAATAGTCATCATTTTTAATATTTCACAGGAATAATACACCGTCGGGTTACAGGAACCCGAAATTGAAATGTCGGGGCAGAGAAGCCCGATAATTGAAAATCGGGTTTGACGAACCCGAATTCAGGTTGTAC

>FLPB003157G22-g1RSP_20030415 (FI167455 )

GTTATCCTCAAATCATGTTGTTCCATTCAATACACCCATTAAGAATGTACTTTTTAGAATCTTACTGATTCCTCAAATATTTGGGAAATGTGCTCTATGATGAATTAATGTATGTTATAAATGGTTGTGTCTGCTCTATGATCAATTAATGTATGGTATAGATGGTTGTGCCTTATCTATGATCTTGGGCTGAAAATGAGAACAATCAGTTGTATTTGCAATTTTCTTTGATTCACTACATTTTGTAAGTCCTAATAAAAGTATAACCACTTTCTCACCTATACACGAATCTCACGGGGTCGTGCGCCAAGGCGCACATCAAAATCTAGTATATGTGAGAATGTATCCGTCATCGCTGACATGGATAGCACACATGTAACACACTAACACGCGTACGTACGGAGTAACACCTTGTGCGCGCGGGTGGATCTTCCGATCCGACCTCCAACGGCGAGGATTAACCTGAACCGCTCATTGTAGTGCGAGGAATATTCAGATTGCACTTGTACACCC

>FLPB002249D02-g0RSP_20011109 (FI167456 )

TATACATTTAATATATGTGGCTTCTTTTCAAACAGATATGAGGGATCTGCTTAAACACCCAAGAATTGTTAGTTTCTATAATATATTTTCCTGGTTGTATATAAATGTTTTTTCTATAGTTCGCTACACGTTTTGTAAACCCTAAGCAGATTATTATCATTTTTCTATCTACACATGAGTCTCACGGGATCGTGCGCCAAGGCGCACATAAAAATCTAGTAAAAAATAATTGGTAACTGCACAATAAGCAAAGCATGACTACAGTGCAAATAATAAAGGGTTGATAAGTGCACATGAAATACATTGGTAAGTAAATATCTAGATAGTTGATCAAAATACGTTTTCGCTCTAACATTCAACTTCTTTTTAGGAGGACATGACGGTACATTATGTTCAGGATTAACAAAATGATCCTCACCAACAACACAACTTCGTTTCGCTTCAGAAGCATCAAAAGCAGAGCTACTCCTTTTCAA

>FLPB002468E02-b0FSP_20020220 (FI167457 )

TCGATCATGCTGATTTAAGTCTGCTCACAAAGCATAAACTCATCATTGTTTTCTCACATGTGTTTTAGTAGGTCATACAAAATATATAAAAAGTACGAGAGCCACGGGATTGTGCGCCAAGGCGCACATTTAAATCTAGTTGTGTATTAGAGTGAACCTTCACGGTTACACAGGCGTACGTCGTCTAATGCAACTTAATTAAATAGAACGGACTGGTGCAATATTGAGCGGCACATCCCATATTTTTGCTTATTTGTTTTCAAAGCCCCTGATAAGTATCGGTGATAAGTCAGTGATAGGCAAAAACTCCGTTATAGATGATGTTTTTTCTCTCATCACCGTGGACTTCTTGTCTGGTAGTATGTGGACTTGGAGTGCCAGTTTATTACTAGACGTTTTATTATGTGAATCTACATTGGTGTTTTGATGACACCAGCAGTTTATCCTTTGTGCATGCATGCATATATAGACAACAGTTTTTTCATTCATCTATGCCGAGCTGACATCTCGCCATCTCGGCGTCGAACCCCAAAGTCTTTCGGATTAGTTTCTTCTTTGAACGTAGTATATACTCCAT

>FLPB002472F04-b0FSP_20020409 (FI167458 )

TATAAATGTTTCTAAAAAAAATGTTTGTTACTTCATTTATAATTTCTTATGATTTACTACGTTTTGTAAGACCTAACAAAAGTATAATCGCTTTCCCATCTATAGACGAGACCCACGGGGCCGTGCGCCAAGGCGCACTTCAAAATCTAGTTACAGGAATTTACACTAGCCCAACGTCTATGATCTGAACTAGGCTGAAACTAGGAACAGAAACAAAGAAAAAAGAAAAGACAAGGGAATACGTGGATATGCTCACGATGTGTGTCCGGCAAGGGATTTTGCAACTCCGGTCGTGGACGGAAACGGAGCTGCAGACGTCGGATGGTTTGGGCGAGGCTTGGAGGCGATGCTTGTAGACGTGACGGAGCTCCACCGGTGGTCAGCTGGTTCCGGCGAGGTTGGAAAGCGACGAGAGCAACGTCCGCGGCGCCGGCGTGTTCGTCTCCCGTTGCCGCGGATGCTTCCGAGCTCCTATGGAAGAAAGAGGGCATTAGGGAGGCGTGGAAGGACGCGAAAACGACC

>FLPB003181N18-b1FSP_20030415 (FI167459 )

TTAATGGCTGTTTCTAAATCGTTTATTAGTTGAGAAGCCAGGAAAATGCACACCAAGGTGCAGGTTTTTATTAGGTTTACGTTTCCCATGTTGTGGCTTCTAACCGACAATGCCGATGGGCAGCCTATGTAGATGTTGTCGATATTCCTTTTCTTACTTTTGTTTCTTTTGCCAGAACAATAATATTATTTAGTGTAATATATTTTTCTATTTTCATGATTGTATATAAATTGTTTGCCACCCCATTTGTAATTTTGTGTGTATTATCCTAATTTCATCATATTGACTTTGTTTTAAACCTAAAAAAATATTATCATATTTTACATGATTTTTATTGTCGTTATACAAAATATAACTTGAGGTGAAATTTACGGGATCGTGCGCCAAGGCGCACATCTAAATCTAGTACTTTTGGTTTGCTAGTTCTCAATCTGATTGCATCATGATTTGGATATAAGTTGTAAGTTAAATTACAACTAAGTTAGTTGTAAATGGGGTTAGAACTGAGATTTTCTTAGTTTGATGCAACTAAGATAAATACTCAAAATTGTTAAATCTGATTCGTGATTCGTGCTAATTACGAGTTCTAAGATGGGTCATAACTGAGATTTGATGAATCTAAGTTAGTCCATGGGGCCTTCTATAGCA

>FLPB002034L24-b0FSP_20010827 (FI167460 )

TTTCCACCTTAATTTTATAGTTGCATACAAAATATAAATCATAGCGAATTTTACGGGATCGTGCGCCAAGGCGCACATCTAAATCTAGTCTCTATATATTACCACAGCTTCAAGAAGCCAGGTCCCAATTGTAGATGCATGCCCTTGAAAGTTGAAACTCTGGAACGAGTAGGTAAGATGGGGTGGGCTCGGTGGAGCACTGGCGCATGGCCATGGGGAGTTGGGGACAGCCTGTTCGGCGCCGCCCCGGACCACGCGCGGCCAAGGAGGCCACCTCGCGTCGCCCATCTTCTTTGGTGCAGCACAGAACTGCCCCTCTTCCTCCTATGCACCCATGT

>FLPB002418C18-g0RSP_20011203 (FI167461 )

TAACGCACAGTACTCAATCCAGTCTGGAAAGGTTCTATTGATGCTATTTGGTGTCATGGATCTCTTCATCTCATATGTTATACTTACGAGAAAATCATGAGTTTGTCAGATTTTGTGCCACCATGTAGATATCTTGGAAAGGAGTTCTTAAAGAAAATAATATTTTGTCCACTTGATTTTGATCTTCATACAAACCAAAAAATTGTACGAATTTAACGGGATCGTGCGCCAAGGCGCACATCTAAATCTAGTTACATTAAACTTTGAGGGGAAAAAACCATGCTGGAAACCGCCAGTTTTCCCACGGCAGCAACGGCGTCAACTCAGGCAGCAGCGGTGTGAAATCCTCGTCTGTAATGTTGTACACGCCGGAGTCGCCGAGGGGGGCCTGATGCAGTCCTCTCGAACTCCATGACCGGCAGGCAAAGACTTGGAGCACGGCTTGCTGAGAAAGAGCGCCCGGCCGTCCAGGCCGCCGTCGAACTCCTTCCACCGGCCCTCCGTCAGGTCTGCCTCCCAGACCTCAAAGTCGCAGGTTGTCCAAGCGTCGCGTTCGACCCATTGCCTCACCATCAGCAGCTTGCCGCTGGACTCGACTAGATATCGCTCGGATACCACAAAATCAGGGCCGAAATCCTCGCGATCTTGTTGGTCTTGTTGCGGCGAGTCCTGTATCAC

>FLPB002304M11-g0RSP_20020424 (FI167462 )

CTTATCGATACCGTCCATTTTGTAAAATCTAATTTTTTTTGTATCATTTTGGTAGTTAAATTTTATCGCTGTTACACAAAATGAACATCAACATGAACTATACGGGATCGTGCGCCAAGGCGCACATCTAAATCTAGTTTTTTTAATGAAGCACTTGCTTACCTAATTTTTTTTCAAAAAAAAAAAAGACTCGCACGTATCCCAACCAAACAAGGTAAGGCCGCCAAACGAATCCCCATACTCCCATAGTGTGTTCAACTTGACCACAAGCCCTAATTTGTCAATTAACTACATCATCGATCAACTCGGCGCCGCATCAACGATCGGTCACACATGGAGGACCGCATCAGCGGACTCCCCGACGACCTTCTGCACGGCATCCTCCTCCACCTGGACTGCCTCCCCGCCGCCGTGCGCACCAGCCTCTTCTCCCGCCGCTGGCGCCGCGTCTGGGCCGGTCTCCCCGAGCTCTTTCTCGCCGGCCTCCACGTTCCGGGCCAGCCGCCGAGCACCTTCCTGCGCACCATCGACGGCGCCCTCGCCGCCTACTCTGCCCTGAACGATCACGTACCCGCGCTCACCATCACCGTGCCCGACGTCGGAGGCTTCCACGTCCCCGCCTGCCACGTCGCCACGTGGCTGCGCTTCGCCTCGCGTTACGTCGCCGCCACCTTCTCCCTCACCGTGCCCCCACTCCCTCGTGAACGTCAACCATACAATGAGCTCCATCTGCTCCTC

>FLPB002423O05-g0RSP_20011203 (FI167463 )

TATCCTAATTTCACTTTATCTACTTTGTTTTATAAAACTTAAAAAGCGTATTATTATCGTACCTGTTTTTTATAACGTTATACAAAATGTAAATTATGATAAATTTTACGGGGTCGTGCGCCAAGGCGCACATCTAAATCTAGTCATAAATAAAGAGAATGGTAAGAATTCAACAAGCTGATGCTTGCCTTGATGCTTGCCTTGCTGATCAACGTCGCGGTGTCACCCTCGTAGTACTCCTTGTTGCCGGACTCTACCTGTAATAACTGAAACAATGCCCGAAACTACATTTGAGAGCAACAATCAATAAAGGAAGTAGAACCAAATGAAACAAATATGAAATGCATGGAATGACATGGCATATTGAAATATTACTAAGTTCCACAGAGCAATTTTAGAGTCCTGGAATTAAAGTTATGCAATTACTAAGTTCAACAGAGCAATTTTAGAGATTCTAATAACTGAACAAAAATTATTTATTTGGTAAAAGCAGTGATGTAAAAAGTATTTCTATTAAATAATGCATGTCAAAACACGAATTCCATATAAAGCTCATCTCATTTGATTATTTGTCTACAATTTTTACTAGTTTCAGTAATTTCAGCACTAGTGCCGGTAGTGCCTAATGGCAGCTAGTGATGTTGGCTTGCCTGGATGGATGAACATGGAGCTGTTCGTTCGTGCAGCTCTGTTCTCTG

>FLPB002217F08-g0RSP_20011109 (FI167464 )

GATTTCATTAACTCAATTTTACCAACATCAAAATTATTCCCTTTATAGATGGTCCGACTTACTTTTGTTGTTCCTTCCTTGCTTATGTTGTTCTTGACATAGTCATTGAGTGTTTGCTACTATAATATATTTCTTTTGTTTCCCTGGTTGTATATAAATGTTTCTACTTCATTTGTAATTTTGTTTGCTTTATCCTAAGATTATCCTATTGATTTTTGTTTTCTAAAACCGAAGAAATTGATTATCAGTTTTCACCTATTTTTTTAGCAATGGTATATAAAGTGTACATCGGCACGAATTTTACGGGATCGTGCGCCAAGGCGCACATCTAAATCTAGTAGGAGAAGGAGAGCCTACTATTATTGTAGCAGCAGGGAGGGGGATTGGGGGCGAAGGAATGGGAATG

>FLPB002504K14-g0RSP_20020220 (FI167465 )

CCATTGATATTTGTTTGCTTCATGGTATCTACGTATCTGAGGATTGTGTTCCTCACTTTTGTTGTGGTATTGTACATATAAATCAAGCGTATGATGCAATGGTTGTATATGTGAGTGCTTTTGCTTCATGCTACTATCAATTGCGTAGTTATGTAGTTTATTTTGTCCTGCGGTGTGCTTTCTCAGGAAAAACGACATGTGTTTATTCCTATTTCACTACCGTTTTATCCGCTCTTATTATTAGTGCTTTGTGCACTAAATCCCAGATACTTACTATGGAGTGGAGTGAGTATTAGTTATTGTATCATATTCGATTGTATATAATCATATTTTCTCCGTTATGATTAGATGTGTTCAGTTATACCTTAGACCTATAGCTTTTGCTTTTCAAATTCAATAAAAATACCCGTAGATTGGTGCGAGCTCTACGGGGTCGTGCGCCAAGGCGCACATCTAAATCTAGTTTTGAGTAAAACCTGCTTGCCCACCCGATCCACTTGGGTACATGAAGTCGTAAATAGCTTCATGTATTCCGGTCACTGAAGTGGAGATTTTTTTCCCGTCAAACACCATGTCA

>FLPB002591E11-g0RSP_20020220 (FI167466 )

GTTTGTCTCAGTCGATGCTTATCGCAAAATGAAAAAGTTTGTTTCTCTGGTTATAATTTATGGTGATTTGCTATGTTTCGTGAATCCTAAAAAGATTTATTATCACTTTCCTACCTATGAATGTGTTTTACGGGGTCGTGCGCCAAGGCGCACATTTAAATCTAGTAAGCATAAAACCCCATCCCAAAACCCTCAAAACTTCTACCCCATTGGCATCCATTGCCAAAATGGGCAAAAAATCTAGAGAGGTAAGTATAGTGAGAATTCGTCCATAAGGTGTGCATTTCTCATATTGTGTGTGGAATCAAATGCACATATCCAATGATGAATACTCGGAGAGATTCAAACTATATATAGGATAAAAGATTGAAAGAGGATAACAAAGTCAAGAAGCTTCAACACATGAAGCAAGCAAAGAAATGAACCAAAAATAAGAAACCAAATAAAGAGTAGATATTATGATAAAATCAAGTAGAGTGCTCTAAATAATATAAGGAAGCTCCCCAAGGTTTGTGCACAAAGATAGACAATTTTATTGAAATATAGGTGCACAAACATGGTATCATCACTCCCATAATATCA

>FLPB002096P21-g1RSP_20030415 (FI167467 )

CGTTTGTAGTTTTGTATGATTTATTCTAATTTCATCCTACTAACATTGTTTTGTGAAAACTAAAAAAAAAATTATTATCATTCTACATAAAATATACATTGGTGTGAACATTACAGGATCGTGCGCCAAGGCGCACATCTAAATCTAGTAGATGAAAGAAGAGCTGTAAAGACTTGACCGGAAAACAAGTTGTACTACAATGGCGCGGTGCGCTGCCGACCAGGCAAAACCGCGTGGAAAAGTAAATTCTATACTCGCCCGTCGTCAGCCAGCCAGGCAGCTACTACTACGTCGTGAAGGACGTTTTCCCGTCGCGTCCGGTTGTTCCTACTTGCAATCCACCACGCCACGCCCACGCTCACTTTTTCTCTCGTCTTTCCGCTCTCCTCTCCCTCGCCGAGTCCGTGGGCTGCAGCCACGAAGCTTCACCACCGTCCCTCTCATACGCTACGCAACCTCCGCCCCCTTTAAATATCCTCCCCCACGCTGCCAACTCCCTTCCACCGAGACTCAGATTCGCCCAGCTCTTCTTGGTTTCGATTGTCAAGAAAGAGGATCGGCCTGCCATGGCTTCCAAGCGGATCTTGAAGGAGCTCAAGGATCTGCAGAGGGATCCCCCGACCTCCTGCAGCGCAGGTAGTTAATTGGTTGCATCCCCCTTGCCTCTCTTTAGCCTTGCTT

>FLPB002670E21-b0FSP_20020409 (FI167468 )

CAATTATCACTTTTAACAATTTGCTGCTCTGTTTGTCCTAATTTAGTTCTAGCAACAACTTTTTGTAAAATCTAAGAAATGTATTATCATATTGGTGGTAAATTGTATCGTTATATCGGCATGAGCTCCACGAGATCGTGCGCCAAGGCGCACATCTAAATCTAGTATAAGTAAGTTCTAATCTGCATAGGACAATTTCGTTGGTCTGCTTAAGAAACTAGAGAAGACATGTTCTTGTGGATGAGAACCGCTTTTGACATGGCACTTTAGATGATAATTAGGTGAAGACAATTTCGTTGCATTGTTGTAATCCTAATCTGTATGGGACAATTTCCTTGCTTTTTAAATTTGACTTTACTCTATATTCCAGTTGGAGAAATTAATCTTGTGAAGGAATATAAGGATGATCATTGGCTTCATCAAACTAGTTGGAATGATCCATGATTGACTTTCTAAACGCGTTGAATATTCCGCTCGCCGCAAGATTGACCAATCAAAGTACGCCTGAAATAATCAAACATATACGCCGGACCAGCAGCCTCGTCCAGATTCGTTGTCACTATGACGATTCCATGCATGTGGTTAGCGGTATCCAACGGCTGCCGAAGGTCCCTCACTAGCTAGGTACTAAACCGAACGCTCCATAATTTAAACCTTGTGACGTCCTGAATAATCCGCGCGCCAGCCG

>FLPB002285L13-g0RSP_20020409 (FI167469 )

TGATCTAATTGTTGCTTAATTTACCTTTACAGTTGTAGTAATATTAATTTTTGGTGGTGCAAGTACCTATTAGGTTTACACGTTTGTGTGTGTCGTGGCCTCTAAAGACCATGCTGCGACAATGGCCATAGGCAGCTCCTATTTTACTGATTGTATATGTGTTTGCTACACCGTTCATGATTTCTCATGCTTTAGCATAATTTTATCCAATAACCTTCATTTTCCACCAGCTTTTTTTATTGTTGTTATAAGAAATATAAATCACCCCTAATTCCACGGGAACATTGGCACTAACTTCACGGGACCGTGCGCCAAGGCGCACATCAAAATCTAGTTTTATCTGTTGTTGAATGCTTATGCATTAAAGAGGAGTCCATTATCTGTTGTCTATGTTGTCCCGGTATGGATGTCTAAGTTGAGAATAATCAAAAGCGAGAAATCCAATGCGAGCTTTCTCCTTAGACCTTTGTACATACGGCATAGAGGTACCCCTTTGTGATACTTGGTTAAAACATATGTATTGCGATGATAATCCATGTAAATCCGAGCTAATTAGGACAAGGTGCGGGCACTATTGGTAATCTATGCATGAGGCTTGCAACTTGTAGGATATAATTTACATAATACATATGCTTTATTACTACCGTTGACAAAATTGTTTCTTGTTTTTAAAATAAAAGCTCTAGCACAAATATAGCAATCCATGCTTCCCTCTGCGAAGGGCCTTTCTTCTACTTTTATGTTGAGTCAGTTCACCTACTTCCTTCCATCT

>FLPB002198O24-g0RSP_20011203 (FI167470 )

ATACGTCTTCTATCACTGCAAAACCAAAGATTACTACGATTAAGATGAGTTAGTCCCCTACTTATTGTCTGTTGTCTTTTGTACCACCGCTAAACAGTGTTCTGATTCAATTCGAATTCCATGGGGTCGTGCGCCAAGGCGCACATCTAAATCTAGTATGTATTGTAGACTTATAGTTGCAAAACCTGTTCAAATATAACTTCACAGCTATTTATGTAAGCATTGTTGACGATCACGTACGTGATCCATTTTTAAAGATACTAGATGAATGATGATACCCCGCACGTTGCTGCGGTATATTTGATTGCCATATTTGAAGACGCTAAATGAAAACAGGGTATATGTAAGCAATTTGTAAGCCTAAATCATGCATAAACATCCAATAACATATTCGTGAA

>FLPB001063I04-b0FSP_20011203 (FI167471 )

GTCTCATGAACATTGGTGATTAGAGCGTGATCATATTAAGAGATTTAAATCCACACACATAGTGTAAACCCTTTATCGCTTTTCCACATGATTGTAGTAGCGTAATATGAAATATAAAAATGTACGACAGCCCCGGGGTCGTGCGCCAAGGCGCACATCTAAATCTAGTATCACATAAAAGGACGTACCCAGTGCTGAGAGCGGGAAGGTGTTAGTGGCAAGCTTTACCCTCACGAAGTGCAATGTGAGGATACCGCGACTTGAAGATGGCATATCACATAAGGTAAAAAGAAAATATAGGATCATGCCAGCATTTTACTGAACAAGCCATGGAAATACAACCCAGTTCTTCTGAACCCTAATTATTCTACTTCGTCAACATGAGTCTTCTGGACCTGCACATTAATTAACAATACTTAAACTGATTTCCCCAATTCTCAAGATACGTCCTAGTTGCTGATGTACACCAACACTTGACAACATACTCTTGTTGATGCACAGTATAAGCCACC

>FLPB002437P16-g0RSP_20011203 (FI167472 )

CATTTTCCTGATTATATGTAAATGTTTGTGATTTTGTTGTTTCATTTTTATCGTAGTTATGTAAACTGTACATAGGTGAGAATTCAGCAGGGTCGTGCGCCAAGGCGCACATCTAAATCTAGTGTTAACAATTCCTGATGCCTCACATACATACGTTGATCTATCTCAGACGAAAAGGCTTGAATGTCAAGTATTTGCCGTCTCTCAAGAGAAATTTTAGTTACGGGTTAGTGATGCGTGCGTGCTTCACTCTAAATTGTACAACTTTAAGTAGGTGAGCAAGTGCAGAGTATGCGGTTCTTTTTATAGTCACACCTTCCGCATGAAAACTTATCGAGGACCAAAGAACGCTAGGTAGATAGGCACCAAATGCAACCTGCATGTCGGATCTAACCCTACTAAAAAACGCCTGAGTGGGTTATTTCTTGAACCTACCGCTTCTTGTAAACAAGGCTAGCTATCAATTGATCTTACTTACCACAAACTAGTGGAATGCCCGTGCTTCGCCACGGCTTCTTATCGTATGCGTAGTATTACAATATACCTGGCTCCCTACTTTATGCCTAGTGTCACAAATAGTATACGAGTAGATAATGCGCGTAAATATTTAAGTACATAAAATAATAACCGAC

Type 2

>FLPB002007M17-g0RSP_20011203 (FI167473 )

GAAAATAGGTTCCTTATGGAAATATAAGCATACTACCTATGACAGGAAAAATATGAGAAAACCAGATGATTCTACATTTGAATGGTTGAAATTTAAAACACAACTTTCCATAGCAATAATGTTATCACATAAGTTTATCACATATGCATAAACTACTTGTTCACCACAATCGATCCAATGCATCCTAGAGACTTCTAATTAAAAGCTTTGGTGAATTGTCGCCTTAAGGTAGCACCACACCTCGATATTCTAGAACTATGTTCTAGGTGATTATTTTGGTAAGTCAAGATAGCGGCGTCGACCGGTTAAATCTAGAAGACTGCAACACTAAAATATAAGATTTTTCATGACATGTACTGTTAAAATGGCTTAAAGTGTCACAGAAGTGACGTAGCTAAGGGAAAAAATAAAAGATTATATGTCCAAAATAAATTAATATATTTCCTATGACACAATAAATATAATGCTCAACATTCTAAAAAATAATAATGCCCTCGCATTTGCGAGGGCCACCTTGCTAGTTTAAGTTAAAAGTACATAGAATGAATATTGAAGTAGTTCAATATAAAATTAAGAAGGTGTTCTTTTCATGTGAAGGTTTAACAAGACTTGAGTGTATTTGACACTTCATGAGTAAAAACACATGAGTGATTTTAGATCACGATTAATATGTACAAAATTAGATGTCCTGTGCTCTAAAGTGTTATGAGCAT

>FLPB002121H18-b0FSP_20011203 (FI167474 )

GTCTTTAATTTAAATAATATGAAGGTTACATATATAACACGTTCGATGCAATAAGATGATGCCCTCGCATTTGCGAGGGCCACCTTGCTAGTTTACCTAATGACACATGGGTTGAATCTTAATTGTGGTTTAGGTTTTATTTGTGATCACCCAAGTGATACACAAACTAGGGTTGAGATTTAATTCTAGGTTGTAGAGATGAGATGACACTATATTGCATGTGCTAGGGTTTAATCTCCCCTAACCATGATAATGTGGTTGCTTACTTAACATTTTATGTTCTCCTTTAGTTACTAAGATATTGAGAATTGGTTTTATCTTCTACCAATAGATTTCTATTATTATCCTTAATCTATTGTTAAAGTAATTAAAGTTGTTATAGTTCTTTTTATAGTTAACTTTGGTCATCTGGATGAATGGTTTCTCACCACATAAAGTATAGAGTTTGACTCTAAAGGTTTTCTTGTGGTTTTTTAATTGAGGTATAGGTGGAATATAAATGTGGTANGATTCTACTTATGATCC

>FLPB001039O02-b1FSP_20010821 (FI167475 )

GAATTTTTTTATTGAGCATAACATTTCACACTAACCGTTTTACTTCCATCTCATATTGTGAATTAGTTTTGCCACTTTTATAATGGTAAAGAAGATGCCCTCGCATTTGCGAGGGCCACCTTGCTAGTTTGAACAAAAAACAGAAGAGAGATGGCAAGGAGGGAGGAGAGGGTTAGGAGCCTCCCACGCCAAGCGTCAACATGAGGCCGTTGCTCGGCAGGGAAACGGCCGGGCTGGAGAGCCGCGTCGTCCTTGCAGAGCTTGAGAAGGAGTGGGAGGGAGGGTGCAGTATCCCTGAAGACGGCAGCATTCCGTTGCTTCCATAGCTGCCAGCAGCACAGAAAGGC

>FLPB002443O08-d0W1_20030528 (FI167476 )

TTTAAATTACCAGCATTACCACATACAAAAACAAAAGGTCAAAGTTACCTTGTGGCTTTTAAAATATTTGAATAATAGTACGAAAACATATACATACATAAGAATTAACTTTTATCATTTCTGACAATATAAAAATGATGCCCTCGCATTTGCGAGGGCCACCTTGCTAGTTTAAGCTATAAAAACATGTGTGAGCTTTTTATTATCTTTGAAAAAAGTGTTCATATAACATAGAAAGACATATTTTCTACTCTCCGTTTTAAAATAATTGATTCAGCTTTGTCTCGATATGGATATATCTTCACATAAAATTGCTTCTAGAATTCTTACCTAAACAAAATTGAATCACTTATTTTAGGATGCAGAGAATAGAAGAAAAAAGAACCGCTCTAACAGAAATACCTACATGAGTATTCGAATACCGTTTTTTTCAGAATTCAGATAACAGTGCTATAATTCTTGCCTTACTGACAGCGTGACCCTATTCGTTTAGAGAGCAACAAAAAATGCTAAAGTGACCGCAGCAACAGCCTCCAAGAAAAGAGTACCCGCACCAATCGCTCTACAGCCAGCGTTCTTGACATCAGTGTCATCACCAGCGCCGGCGCCGTCCAACCCTTGCGATAAGACCGACCCAGCAGGAGGCGGCGCAAGGACACGTTCCATGGGCGGCGGCGGCG

>FLPB002689M08-g0RSP_20020409 (FI167478 )

ATCAAATTCAATGATACATAAGTAATCCGTTAGCATAGTTGGATTAAACAGATAATAGAGTAAAAACAATATATATATTTAGGTTGTTCGTCGGTCTATCATTAGAGAGAACAATGATGCAAGTGTAAGAGGAGCAATAACAGTATGTTGGATTCCCATGATAAAAACCCACAAATGCAAGGTTACACCTTCAATATCAACTCTAAAAATGGTGGACTTTTAGTTAACAAAAAATAGAAATTTTAGAATATGTGAGATGATATTAATGGAGATGTCATGGTAACTATTATTCACAAAAAAAATAAAAAATATATCACAAATGTGATGTTTTACCTCATATAATTTTGTGGGAGAATTTCAAATTTGTGTTATCTATGACATCATAAATTTATATCATCATGAGCTTTAACCTGCTAAAGATTATGCCCTCGCATTTGCGAGGGCCACCTTGCTAGTTATCATAATATCTATATGTGACTCTAGCTCGGTTTGTCATCGTATACCAAAATGGGGAGATTGTGAGGGTATTTTACCCTTATCCATTATTTTGGTAAGAATGACACCGTGCTAGAGTATTTGGTCTAATACATGTCTATGAGGTTATTCCCAGGTATTAGCCAATGAGGTAAAATGGTGTATCAATGGAACAAGAAGGCAATAGGAGACCCCTCACTTCAATGAAAATCAACAAGGGTTTCTTCAGCACCTGGCCGGCCAGAGGCCGACGGTATCGA

>FLPB002052F15-b0FSP_20010827 (FI167479 )

GATAGTTGTTTGAATGTAACACACGAAATATGTTTGGATCNTACGCTATTGAGTCTAAAAGAAGTGTTAGGGTGGTATATTGAATTCGTATAGAATTGAGCCATACAAATGCAATCCTTAAGAATTTTGGCATCACAAAGGAAATTCATAGGTAAAAATACCCATGTATTAAAAAGTACAAAAGTCATATAAAATTCCTACAATCTAAAAATGGCCTTAGAGGTGTCATAATAACTACCTGCCACAAACAAAAGAAATTTCAAATAATTCTCAGCCATGTGATATTTAACCCCACATGTTGTTTGCACAAGCTATATAAACCTATTGTTTATGTTTCATGTATATTTTACATGAACTTTATTTTACAAATAAATTAATAATCTAGAGACGATGCCCTCGCATTTGCGAGGGCCACCTTGCTAGTTTGCTCTAAAATAAAACCCCCATCGTGCATGGATAAAGACTTTTGGTGCATTAGAAGCAGGCTGACGTAATTATTATACTTTTTGCGAATAGGGTGAAATAATACATAGCTATCTACCGT

>FLPB001075O22-g0RSP_20011203 (FI167480 )

CAATATTTTTTATTTATACATAATAGATATATCATATTGTACATGCGAGCCAATACTCAGCTCATATATACCAAGTTTTTATTTGCAACAATGATACCAATAAGAAGAATGGATTGCATATACCTATATAGAGATTGCAGCTTACTTTGTCGGTGTCGCACTCCAGCTTTTTTGTGTTATAAAAATATTATTAAGAAAACATGAACCACTACCATCCGATTTCCGTTAGAGATATTTCAATTTTTTTTTGTTATTTCCTTGCAACGTACTAATATATTAATTTACAAAATAGAGATAAAGTTAGCAGGTACCAATTTATCTATTTTCGCAGTGAAGGAGACGAATAGAAAGAAAAAACTTTAAAATAATTTAAAATATTTTTTTACAATTTTTACTATAGATGATGCCCTCGCATTTGCGAGGGCCACCATGCTAGTTTAAAATAAAGTCATGATAAACACCAACAAGAATTATGAAATAAAGGGAGTATCTGCTTTTCCTTTCCGTGTATACAATACAGAAATGCAACTGCCTAAATTATGGTCCCA

>FLPB002405D15-g0RSP_20011203 (FI167481 )

TGTTCAAAAAATGATGTACTTTTCAATAAAAGTTGTACATCAAAGAAACATGATACATCGATAGAACAAAAATAAATAGCTAACACTTCTGCACATTGAAGATGATGCCCTCGCATTTGCGAGGGCCACCATGCTAGTTATATGAATAATGCATATATTATTTGATGATAATAATAATAATAAATGAATAAAAATATAATTATTTTATATTCAAATCACACACATTTTTCTAATAATAAATAATATATTATTTTTCCAATTTCGGTTTATAGATTGTTTTGGTTTCAAAAAATACAGAAATGTGACACAATGCTATAAGAGTTAATATGATTGATATGGTAGTATTTACAACAAGGTAGAATCACATTCGCCGAAGCGTAGCAGGAAAGAACCAAGGAGTTAAGCGTGTTGAGGGTGGAGTAGTGTGCGGATGGGTGACCGACCGAGAAGTGATGACCAAGTCTACAAGTTGACTACATATTAAGTGTAATTAGTTTTAAAAATGATGCAAGTGAGAAAGTAACAAGTCAAACAAAAAGAAACAACCGGAACAAAAACGAATACAGAAAAAAGAAAAAAATGAAATTAGGAAAATAAACATTAAAAAATGAAAAACATAATTTATGGAAAATAATATTTAGGCGCGGCGCCGTCACGATGCCGGGCGCGACGCTCACGCCGTGGCCTCGACGGTTCTCGGCTTTCTGCCTTTCTCGCATATCTAGGTTGATCAATTACCATAAAATTATATAC

>FLPB002273M10-b0FSP_20011203 (FI167482 )

ACCATATGTTGCTTGAGACTTGCATACACCTTAATCAAAGAAAATTTTCCCTGGAATTCATACAAATATAGGTGTACACATAGAACCAAAAATGTCCTTATACGTGATCCTTATTTCATTATACACACTAAAGGGCAAATTCACGCCTACGAAAATGTATCCATATCTAAATGAATAAAACGGGTAAACTTAAGGAATAAAAACATGCTTTAATGTTTTTTTTAGAATTTGTAACACATTAAAGATGATGCCCTCGCATTTGCGAGGGCCACTGTGCTAGTTGTAATAGTACTACATAGTTTCCCATGGATCCTTCTTAGTCATTCCAAAATGAAGCTTTTGGGAGGTTTTTGAAATTTCCATGTCTGAAACCAAAAACGACTTCTCTTCATGCATGCTTGACTTCATAAGACAAAGTTTAGCTAGGGGTATAGGGTAGATACATGATTTTCCTGGTTTGAATATGTCTAGAACTTGTTTATCAACTTAATTGAATGCTTACTATATGACATAAGAAGACTATGTGCCTTGGTTTTTCATTTTTTTGATTTTTTTCTAAAATTTGTATGCCCATTTGAAT

>FLPB002055N01-b0FSP_20011109 (FI167483 )

TCAAATGTGACCTACCTATATATTAAGGAGGATAAACCGTTTTATCCTAATTCAAAGATTTTCAAGTCAAAGATAACTATCCTCGCATACTGATCTAACATCATGTAGAACTATAATTCAGTTTTTCAAATCGAATATCTTTCGTAGGTGTTTTAACATATGACATGATAATCATTTTCCCAAAAAAATTATATTGCCAAGATTTTACTCGCATACGTGATATTTTCTTACCTAGATATTTATGATAATTGAAGAACAATTTTGTTTATGGTTACATAATAGGAAATAATATGATATCAGCGAAACCATATTTTATTTTCTTGAACACTTCCAACCAACTACACATGATGCCCTCGCATTTGCGAGGGCCACTGTGCTAGTGTTATATAAAGACGTATATTGATGTGCTAGAGATGTATAACCAAACCAAACGTCTTCCCAAAGACGGACTGTTGACCCATCTCCCACTTCAAAGAAATATCTACTAAAAAAATCATCCTTAAACCCGCATAAGTCTCTTCCAAAAAAAGGGAATCGGTTAGTTTTGCTTTATCATCTTCCAATGTTTTATTATTAGATACTTGTTATGCAGTAATTGTTGCCGAC

>FLPB002296I02-b0FSP_20020513 (FI167484 )

GCATTGCAACAGCGGCGGGTGGTTCTCCCTACTCCTTGCATGTACAAGACTATGTTTTATTCTTTTATTAATAGCACACTATATTTAAAAAAACGTCAACTTGCGGAGAACTGAGAGGGAGGCAGGTGCTTTGATCGACCCTCCTCTCAAACCATCTACTCATCCCATATGGTCGTAAGTATACATAGCTGCTCCTTGCATGTACAAGCCTTTACTTTATTCTTTATTAGTTACCAGCAAACATGAAAAAGGTATACGCGTTTAGACAAAGTTCACCTTGGCATTGGAAATAATGTAATTAAGTTTATGATTCATGTAAAGTTACCAAAAGTGTAGGTTTTGTTTTGATATTTTTCTGTATTTCTAGATGATCAATACTATACGTGGTTAATGCACAATACTTGACTATTGATCTAACATCACATAATCGGCAAGATAAAGAGCGACATAAAATTTAATTTGACGTCTAATAAACACACAAAGTATACAAATATTATCATTCTCATGTGAAATTATTAGATAGCATAATCATAATTTTCCACAAAATAAAATGATTAAGAAAATCTTACAAACAAGTAATATTTTATTTAGGATAACATTTGCCATAGCTATAGAAACATATTATTTTCTAATAATATAACAAAACAATCAAAAATAATTTTTTTTCTCAAAGAGGCATTGCACAATAACCATGATGCCCTCGCATTTGCGAGGGCCACTATGCTAGTTCAAAAACATAGCAACGCATTCATAAG

>FLPB002544J16-g0RSP_20020220 (FI167485 )

CCGCATGCACATAAAATACATGTGACCGTGTGTAGAACCATTAACCTCGAGTACTGATTTAACATCATGTAATAACTATAGTTCATTTTACAGACCAATATCTCACTTACATGTATGTTCCAACATATATATAGCATAATAATCATTTGCCCAGAATTTTTAAATTGCCAAAGTTTCTACTCAGATATGTGGTATTTTGTTTACCAAAGCATTTGTGATAGTTTAGTAGAAGAATTTTATTTAAGGTCACATAATGAGCAATTATTTTTTTATCTCTTGAACAATTTCAACCAACTACAGATGATGCCCTCGCATTTGCGAGGGCCACTATGCTAGTTATTAATGAAAGTTGATTAATTGGGGCTGGGAACCCCGTTGCCAGCTCTTTTTTGCAAAATTATTGGATAAGCGGATGTGCCACTAGTCCATTATGAAAGTCTGTCAGGAGTAAATGACAAAATTGAAAGATAAACACCACATATTTCCTCATGAGCTATAAAACATTGACACAAATTGAGAAGCATTTTGAAGGTTTTAAAGGTAGCACATGAGAATTTACTTGGAATGGTTTGAAATGCCATGCATAGGTATTTATGGTGGACACTTTGGAATAACTTGGTTTTCAAGGGTTTGGAAGCACGAG

>FLPB002305F22-b0FSP_20020513 (FI167486 )

GATACTCTTTAATTGATATATTTGTGGTGTAGACATTCACAAATGAAATAGCTTTTGTGTTCCATACAATTTTATTGTAGTTAGTTGTAGTGATGGTCAATATTTTATTGGACTTACGAAACTATCCTCCTTTGATTTAATACATTGGTAATTAATGCATAATGTACATTATGTGGAAATTAATTAAAATAGCTAGATTTTCTTCAAAAGATGTTACATAATTCATTCTTATTTCTTAATTACATAATCAAAATATGCACACATAAGTAAATAAAACTTAGGTATATAAACATATAACGTTCTATATTTCTTAATAAAGGTTATACATTAGAGGAAAAATAATATTTCCACATGATTAAATAAATAGTTCATGATTTCGGAAAATTGAAGATGATGCCCTCGCATTTGCGAGGGCCACTATGCTAGTTATCAATAATAACATTACCAGGAAGCTCATGAATGGGGCATTTGAGCATTAAAGACTTCAATCTCCCCCAAGCTTGGGCAATACTCTCTCCATCATGAGGCCAAAAATTATATATGCGATTCCGATCCTTGTGAATTTCACTTGGAGGATAGAACTTAGAATAAAACCGGGGCACAATATCATTCCATTCAAGAGAATCCCCATTATCCAGTAATTTATACCAATGCGCCGCCTTACCGGGAC

>FLPB002270E05-g0RSP_20011203 (FI167487 )

AGGACTTACTCAATAACATACCTTAGTCAACCTCTCTTTATCATTACTTTATGTATTTTTTAATGATTTGAATATCATCTGAAGCTATTACGATTACTTCTTATACACCATACCCCATTTTTCCTCTGGGCAAAATAGAACATGACATTAGAAAAGGTAGAGATGCTGGCTGCACCTTTGTGTCGATACAACCACCACTGTTATTATGGTCCATGGGATGGTGTTCTTAAGGCCGTTTCACACATGATTTTTTTAGCATGACATATATAATAATATATTATTGTATTAGTAGAATAGCATGGTGCATAAAAATCTACCTAAAGTGGACACCTTTATTACTAAAAAAAACTAACAACTACTTAAAGAGGATGCCCTCGCATTTGCGAGGGCCACTTTGCTAGTTCATACAATGATATAACCTTGTTATTAATAACATCCAATGTTCATGATTATGAAACTAATCATCCATTAATCAACAAGCTAGTTAAGAGGCATACTAGGGACTCTTTGTTGTTTACATATCACACATGTATAATGTTCGGTTAATACAATTATAGCATGACATATGACGT

>FLPB002256B19-b0FSP_20011203 (FI167488 )

ATTGTGAAATATATGACACTACGTGAATATCTACCAAACTATGAATTTATTTTAGAGTCGCCTTTGAGATATTGTAGATGTTGCCCTCGCATTTGCGAGGGCCACCTTGCTAGTTATTCTTAATAGAACAAGGAATAGTCGGTATACCTGGGTCGCCAAGCTTCTTTGGAACCTTGCCATTGAAAGAGTAATTAGCAAGCATAGTGGAAATCTCCTCATTAGGAATTTTCCTTTTGTTAGTGACAATATCTTTCATATACTTNTGAATAAGTGAGACAGTNTTAATAGCATCAGTCAAAGGGATTTGCAAGAATAATGGGTTTCATCCAATCATAAAATTTATTATAGTGTTCTTCTTCCTTTGATTTTAGTTTCTTAGCAGGAAAAGGCATTTGCTTTTGAACCCAAGGTTCTCTTTCATTACCATGTTTCTTAGCAATAAAATCTTCTCTAGTATACTTTTTATTCTTAACATGCTTTTCAGGTTCATTTTCAACC

>FLPB002300D02-g0RSP_20020409 (FI167489 )

GTATTGCCAATTCAAAGCTTGAAGTCAAATATGTGATTTACTAGTTCAAATTCTGTTGGTTGTTTGGTTAATATAAATTGATGAAAGCATAAATTCACCTGTCAACGTGTAATAGAAGTTGAAAGAAACCGCAGAGATCGAATCTATTTATATGACTCTATCTTTATTTTTGACACTGTAAGTATGGTAGTAGATTCGAATATTAAGCCCTTAGAAACTGAAATGTTACAAAAAATAAATCATTACCTACCAAAGTTCTATTTTTCTTAGATTAAGTGCCTATTATATATGATCACGTACTCAGCACAATTAACTAAAATTGCACAAAAAATCATCCAGCATTTTTGACACATTTAAGATAGTGCCCTCGCATTTGCGAGGGCCACCTTGCTAGTTGTTATAAAATGACATGTTCAATTGTTTGAGGCTTACAACATCACACAAGCCTACCTGATGAAGTAGTTAACAAAACAAAATAACTTGGATGCATGACAACCTCCAAATGCCTATTTAAGTTCTCGTAAACAATTTGCCTATAGGCTGTAAATAACCGAATTATCCCCCAACCCCATTGCGCATTATTAATAAACACGAAAAATCCAAGCCTCAAACCCTACTGAACAGCATATATATGTCCATCAGAACGGGAGAGTAATAGTTTGATTTGACGGAGTATTTCTGTATACTCAG

>FLPB002354B08-b0FSP_20020416 (FI167490 )

GTCCAGAAGTATAGATACATAGATTTTTTGCAGGTATAAATAAATAATATTGGGTAGTTGTCAGCATAAGTGATCCGTGATATTTACCACCCAGTATCGGCAACTAGATACAACCCCCTTTTACTAGGAAAAACAGTATGATCGTAAAATTTTAATCCTGATAAAATTGTATGGTGATTTTAATATTGAGTATAAGAGATCTTAAAATGTTTTTACTGTATCACACTCGGCTCGAAAAAAAACTAAAAGCTATTATGTGTTTCACACTCAGTTGAAAAAAATAGTTCTATTAGTATATTATTTTCGAAAAATATTATTGTTTGTGAGTTTGTACGGTTCTCCTATGATGTGGTGAAACAAACTTTATGTTATTTATTACTATACAACTATATTACTCTCAACCACAAAATAGAAATATCTAGAATTTTTTTAACGCGTTTCTCAGTCACTTTTGACAGACCAAAGATTATGTCCTGTCTCTCCAAAAACAAAATACCATAGAATGTTGCCCTCGCATTTGCGAGGGCCACCATGCTAGTTCTCTCAATAACAGATGTTTGTTTGACAATAACACAAATACGTGGTGTGAAAGATTAGGAAACTATATCAAGGAACCACAT

>FLPB002560D04-b0FSP_20020220 (FI167491 )

GAAACAATGATTTTCCGTGGCCACCTGCATCCTCGGATGATAGCTCCATGAGCTAGACATTCCACCGATTAGTCACTCGCTCCCAGCAACATATATATTGATATCATTTGTTCCAAGAATTAATCTCTCTAGAGCCATTTTTTTATTCATGAGCAATAATAAAGCACATAATCGACTTCGGTTGTAGACCACAACAAATTCTAGATCACATAGATTTACAACGTGAAAATTATTGTGTAGATTTACAATGATCTGGGATTTGTATTATTCTAGATCCCATGACATGATCCATAAATTTATTTAAAGTCATTTTGACATATTAAAGATGATGCCCTCGCATTTGCGAGGGCCACTAAGCTAGTTTCTCAAAATAACATATGTATTCTATTACAGATGCCCTCATCAAGCTATCGTTAATCTAATTTAGCTAGAATACTTTTATTCAAATTTTTAACCCAAATATGAATATTACTCAGAGAATTAGACAATACACAGTAACTGAGATAATTACACGGCCAGGATCCTATACAGGTCCACAAATCGTACAACATCACACTTAATTCAGTAGGAGTATATAAATTGCTTTGCAGAAACGTTAGGAAATGGATTAAAGAAAATTAAAAACAGAC

>FLPB002154J16-b0FSP_20011203 (FI167492 )

TCAAGTGCGATAGAAGCTGTCAGGCCGGCTGACTTAGTATCGATTAGCAGAGTTATGCACAATGATTAGTTTAGTTCACATTCTCATAAGTCCAGACCCGACTTCCTAAACCCGTTAATGAGAAAAAAATGGGCCCCCATCACAACATTTTGTGAAGGAATGTTTTGTATTTTTTATTACTTAGCAACCTAATGATAACTTCATATACACCATAAACTCAAATTTCTATATGTAGGAATATATACATGCATAAATATCATGTATTGTCTTACTAAAAGATAACGAAGAATATTTGGATAAGATGGAAAAATCTTTGATACATTAAAGTTGGTGCCCTCGCATTTGCGAGGGCCACTGTGCTAGTTCATATAATAACATAACCTTGTTATTAATAACATCCAATGTTCATGATCATGAAACTATGATCATCTATTAATCAACAAGATAGTTATACAAGAGGCTTATTAGGGACTCATTGTTGTTTACATAACACACATG

>FLPB002656L18-g0RSP_20020409 (FI167493 )

CAGGAATATATGTAAAAGAATGATGTAATTTAACATGTACTTTTTTTTTTGTTTCAATCTATGTTTTATAGTCCCTCTCTGTTCACAATTACTTTGCATTCTGGGTTTGGTCAAAATTAAATTTCATAAAATTTGAACAAATATTTATCAAAAATATCAATATTTATCTGATAAAATTTATTGTAATAGAATCATCGTAAATTATATTTTAATTATATATTTTTGGTATCTGGATATTGATATTTTTTTATATTTTTAGTCAATCTTTGCAAAGAGACTCTGACTAGCAAACTAAATAAAAACGGAGAGAGTAATGCCTTAAGGTTGAAGAGAACATGACACTGAATAAAATTTGGTGAAGTATCATGGTAAAACTATGCTACTAATAGATGAGTGTTCATGACCGATAAGGGCACAACCATGCAATAGGATAGCGGGATTGAATACATTTCGTGTTGCTAAGACATGCAACAAAATAGTGAACTTTTCAGTTGCTGCCCTCGCATTTGCGAGGGCCACCGTGCTAGTTATTGTAGAAACATGATAAATAGATTCCCCACATATTTAGACTATTTCAGTTATTCTACAATTAAAATGATGATTTTTTTTTCAAAATATTTTGTGAGATTTCATCATGATTTTACTAAAATTTCAAATGTATATCATATCAAATTGAAATGGGTCAAACATGGCAAAATGACACAGCCC

>FLPB002438N06-g0RSP_20011203 (FI167494 )

CAAAAACTAAACAGAAAAAAAGATGGAAAAGACACTAAGAAAGGAACTTAATCCATCTTAGAAAACTATCTCTATGCTTGGCCTTGGTATAAAATAAGAAAATTATGTACTGTGCCTCGTAGATCTAACTAATTATAAATAAATAATTTGTAAATAAAGAAAAGCTTGATATGGTGTCATGTATTTTCGAACCAAGGTTGTAAGAAAAGTATGAAATCCCAATGCATAAGAGAGTAAATATACCCATATAATTAATATAATCACATGCAATAATATACTTTTATAGAGATTATAGAACCACATTATGTCCTACATTATAGAGTGGATGTGATGTTTTTTAAAGGAGTATAGCCACAATGAAATTTGGATATTCTTGAAGCTGTAAATTTAGGATTACAACTTTTTAATACATTAAAGACGATGCCCTCGCATTTGCGAGGGCCACTGTACTAGTTTAATAAATGACGAAATCCAATCCAACTCTTTAATCTCTGCATCCTGGGTACATATTTTTTTGTCAGGATCACGGCTTGCTGGCTAGTTGATCGATCTGTCATCTAGCTTGAAAGCAACCCGCGTGCTTGGAGGGACGCGCCTGGGCAGCCACCGAGCTACAGCAACTGGGATCGGCTTCGCGGAAATTCCCGCGCGCTAGGACAGTGCTCCCACGAAATCCACCGGAATGAATGGTCTTGTCCGACT

>FLPB002040D13-b2FSP_20011109 (FI167495 )

GTCTGGTTGGTTTTGATCTTGCTTTTAAATTACAAGTGTAAGTATATGGTGATTTGGCGGCCTTTTCGAACAGAATCCCAATTTATTTGTGCTAACATATATTTGAACAATGACTCACACTTGAAGAGTTCTCAGTTGTATTCATACAACACTTTGCTTCGACAAAGAAATGAGCGGTCATGCTGTAATTGTATCGTATACTTTAGTTTGTATACACATATTTCATATTTAATAATGATGTGTAATGGTGTATGCCATTCATTTGTAGGTTCACCATTTTCAAAATTAAGAGTGAGTTTCATTAAGGATACTATAGTTTTTTTTCCATCGCATACCTATGAAAACCCCTTCTAGACACATTCAGATGTTGCCCTCGCATTTGCGAGGGCCATTGTGCTAGTTCATCTAATGACATAACCTTGTTATTAATAACATCCAATGTTCATGATCACGAAACCATAATCGTCTATTAATCAACAAGCTAGTTATACAAGAGGCTTACTANGGACTCCTTGTTGTTTACATAACACACATGTATCAATGTTTCGGTTAATACAATTATAGCATGTTATGCA

>FLPB002257K14-b0FSP_20011109 (FI167496 )

CCACAATTCACTTTATTAATTATTAAAACATAATCATATGAAAAACAAGGTCAAAGTGACACCTTTAAGATGTAGCATTAACTAAAACATTGTGTATTTTGTAATGAAAGTAGTAAACAAAAACGTTTTCTAACACAAAGGGCACACACACTCCCTTTGTTATTGCATCCATCTATGGAAGGGACCATATTTTTATAGTGACATATTGCAAATATCTAAGAAACTATGAATGTATTTCTCGAACTGATGCCCTCGCATTTGCGAGGGCCACTATGCTAGTTCTAGAAAAGATACATGTACTAATTTGTGCAAACAAGACAAATCTGATTATTGCAAACAACAAAACCAAATGCTCAAAACAACACCAAATTTTTTAATTTTTTCATAGACCACCAAACATGAAATTTCTCACCAGAATGTTACTTCCAGTTAGAACACGATCTCATGTTAGTTGTAAAAAAAATCAGATTTTTTTGTTTTCCATTGTTTGCAAAAAATTTATTGGACCAAGAGCATATATATGAGCTTGGGTTGAAAAGTGCATTTTCAAGA

>FLPB002165B21-g0RSP_20011109 (FI167497 )

TAATATTTATTGCAATATATCTTTGGTGGTTACAAAATCATATCATATCTTATTGACATAATATGGATATACATGATTACAGATAATTAAACAAATAAGTTAACAAAATATCTAATGAGTAAAGATGTTGCCCTCGCATTTGCGAGGGCCACCTTGCTAGTTCTTATAAAATGCAGAAAGTACTAACCCTGGCGAAAAATACAAATTATTCAACTACTTTTGCAGCTGGCCAAACATATTTTGGTGGGACTAACATCACAAGGTATTCCTT

>FLPB002159L22-b0FSP_20011109 (FI167498 )

TCTGTAAATATAGTTCTTGATAATTGTCGACACAATAAAGACGAAGTTCCTCTCTATCTAATAAAAATTTCAGATGATACCCTCGCATTTGCGAGGGCCACCATGCTAGTTGACATTAATTTTAAAACCGAGGAAGTATATAATAGTGTTCGATGTTTCAGTGTAGTCCCTCCGTTCCGATCTACTTGACTTCAGTTGGAACCATTGCTTGTCGATTTGTTTGTCTCCCTCGCGGTCAGTTAGAACGAACAATGCTTAGAACTTTCTCTCTGTTTCTCTCAGTTTCTAATACAACAATTCAACACAGTAGCATTTTTCTCATCCATTAATTAATAAGAAAGTATCCAGTAGTCTTTTTCCATTTATCATCCCAGCGGTATATATGACATGTCCGGACCGAAAATACATTTTGTCTACCTATCGAGAATCCACAGTTCATGACCCCTTCGCGCTAGGCCTCTCGTCATATTGTCTTGCTTTGTATCCGTACGGCGTACGCTATGTTGTTGATGTACTTGTGCAAGAACTATAACAAAATCTTGTACTCGCTCGGAGTAGAACTTTTTATGGAAAATTTTGAATTTATGGACAATGTTTCTGAAATAAATATTATTACTATT

>FLPB002243E13-b0FSP_20011109 (FI167499 )

ATATAAAAATATAGGGATGCAAAAAACTAGAATATAGGCTTGCAAAAAATGTGGAGAGGTATCCTACTAGCGAGCACACAACAATTTATCTACCTAGTAGCCAGTGTTAGTTTTGTTGGTGTCCTTGGAACTTCCGTCTAAACAATAAACGTGTTAGGTGTAGAGAACCATGACCACTAGCATTTATTAGTTCATATTATTTTTATTACTTATTAGCAATATAATCACGTATAAAATGAATACTTATACATGTCTAAAATATTTTCTGCCAAGCAACAAATGGGATAAATAAGAGAAAATTAATTTAAAATATAAAATTAGCACTCTTGATATACCAAAGATGGAGCCCTCGCATTTGCGAGGGCCACGCTGCTAGTTGCTAATAAAAACGTAAACAATACTGGACATCATCAAAGGAATTAGCAGGCCGGCCATAACCGTGAGCATGTGAACACTATCATGTGAACATACCAATTAGAAGTAAAACTTGATCATCAGCATGCTATTTATTTAGGTCGAGGCTTGGCATAATCTCCTAATCATAATTAGGACTCTAATATGATCAGATCTGCTCTGATATCATTGTTAGATTTAACAGGTCATTAGGGGAGAACATTAACATAATCTAATCATATCTT

>FLPB002053E12-b0FSP_20010827 (FI167500 )

CATTTACCAGATTGGATCTCACATCAAATGAATGGAAATTAGGCAAAATAGACACTACTAGCGTCTCTCCCTACAAAAACTAAAACTCGCAGTACTAGGTTGTCAAGTGATATCTTCGTAGGTGTATCAACAAAAGGAAAAAATAAACATTTTACACGAAGCTAGCTAGATCGTGGTGGAAATGCAAATATGTGCTAAGATAGTATAAACGGAGCAATAGGTTGAGTATATTGATCCGTAAATATGCATGTTTGAGCAATCAGATCAGAACAACTGGGTTTCCAATTTAGTTATTACACATATAAAACAGTGATGCTAAAGTGTACGGAGAGCTTCTACAGAACATCAATTCTGCTCTAAGATGCAAACCTTACATGGAGGATAGAAAAACCCGACAGATTCAAATTGAAATGAGAATAAACATTTGCAATCTATTAGAGAAAAATCAATATATTACAACACATACTGAAATCCTAATTTTTTCACATCTATCTACTACTTTCGTCGACTATGCAACTATATTTCGTACAATAGAAAGTATCACTTTTGACAAACTAAAGAGGATGCCCTCGCATTTGCGAGGGTCACACTGCTAGTACCTACCTAATAATAAAGGAAATAAGGTTTCTTGCGGTCCAACACTTTTTTAGATCGATTTGCCCTCCACCAAATTACATAATACATACAACTGCCACATTACCGGTTTTGTTCTTTTTTCTT

>FLPB002685K23-b0FSP_20020409 (FI167477 )

ATAAATAGAAGGAAAAAAGGTTAAATAAATTAAAATTATTTTTTAACATTTTTCACTATAGATGATGCCCTCGCATTTGCGAGGCCCACCTTGCTAGTTTTAAAATAAATGTCACAACTATTTTTACCTACAGAGACATTTATACGTGAAATATATACAGATACATTCGAATCTAGATGAAGTTAAGTAACTTATTTATAGACGGCGGGAGTATTATTGATTCTCGCGCTAAAGCACAAACTCCACACCGGTTCAACAGTTTGCACTGCGCGCGACGCAGGTCGCCTCGATGTCTTCTGATCCGTTTCTTGTCTGATCGATCTCAAGATGTGCGTCTGGTCTCAAGATGCTCGCAAAACGTAACATTTATCGTTGCGTGATCGGAAGTTTCGAAGCAAGCAACAGGTTGTGGGTACAGGGAGTGAGGCTACTTGTTCCGATGCGATTTCGTCGGTATTGTGCGAGGCAAGTTGGCTAGCTGCACCTGCAGTCGCCGTGAAATCTCATTAACACCGAGGATGTCAGACCAGCTGCAGTCTGTGATCTTCGTACACTTGGCCTCAATGGCGAGTGATGACTGTCGATGAATGAATAGTTAACGGCCCTGTGAATCCATCACATGGCTGAGATAGCTACGGTCCGTGGATCCCTCTAGCTCTAGCTTCCACTGCTAGCTACGAGTATACT

>FLPB002041M21-b0FSP_20020214 (FI167501 )

TACACTGTTAACACAAGAAGAATTTGTGTGACATACATCGACATTGACATATATCTGAAAACTGAATGCTTGATAGTATTATGATTTTCTTATAGGATTATTTAGATATTAGTATATAGTAATGATTACATATATACTATATAACTTTCGATACATCATACAAAATATGTAACATATCCAAATGTTTCCCTCGCATTTGCGAGGGCCACCATGCTAGTTAGAATTAATATTATATACTAACATGCCAAAGCACCATGGTGATATATGTGTCCTTCCGATACAGATGGAGCAATCAAGTAAAGATTTATCGGTCATTATCAAATTAGTCATGAGCA

>FLPB002535F23-b0FSP_20020220 (FI167502 )

GTCAACTGAAAATAAATATCACTAGAAACATTTTCACCCTTTTAACCCAATATACGTTCATGACTACAATTGTGGTCATAACAATAGATGGTTCAAAAGGAAATCTGTTGGACAAAGAAACTCTCCGGGAACCAATGCCATTGCAGAAGTATATAGTACAAAGGTTTGAAAAAATAATTATACCGTTATTATTGCGTAATAATTTGAATTTTATTTAATCTAAAATATTTTATGCGTAATTTGCTTTGTAATATTATATGAACAATTTTCTCTAGATATATGTAAGGCACTCCTATGTGCCAATTATACGAACACAAAGCGAAAATAACACGGATATTAACAGATATATGGCATTGCGATTGATATTTGAGCCAAGACTATGCATCAAGTAACAAAACTCATTTGGGTAAAATTAATTAAACACTAGGTTTGGTACTTTTAATAATTATAAACATTACTTCTATAAACCTACAGCATATAAAACATTATAGAAAAAATAAATAGCTAGAAGTAAATAACTTTTTAGATGTTGCCCTCGCATTTGCGAGGGACACATTGCTAGTTTGAACGAAAAATAATATAATATAATGGTAAAATTATTATACTCAGGAAGAACTTAATTTTGTTATGATAAGAACCGGCCTAGCTACTGAGTGCTGACCCACGGCTGTAGAGAGCAGCA

>FLPB002320N05-g0RSP_20020513 (FI167503 )

TTTGCTTTCGGAGTTCTGGTCTTACCATACTATATATAGGATTTTAGTTCGTATTGATCCAAATACCATAACTTATTTTAATTTTCCAAAACATGAAACTATCAAAATAACATCAAGATAATTATTTTGGCGTTTTTGCATATTACTTACATTCACAATCGAACCTAATGGCTCAAACTATGATTAAAATACATGTGTATATTTTCGAAAAAGACGGTATTGTAGTTCCAAATCTAATCTTGCACTGATACTAGAGATAAGTTCCATTTTTAACTGATCTGCATGAGCATATTCAAGCATTTGAAATTATTGCATAATGTTAGATCACGCAATATTATAATTTAGATTGGTCCAAAATAAAATATAGGACAGTTAAATTCTAAGATTAATAATTTCATGTTTCCACTATTTATTAGGCTATTACACCATACATTTATTTCATGAAAACATTTAAAAAAGTTAAATTCTATGCCCTCGCATTTGCGAGGGCTAGTTTGCTAGTTCTACATAATTATAATTATTTATGCCTTTTATTTTGGTGTATTATGCTATTTTGGATGTTTATTATAGTGTTAAATCAATTTAAAAAGAAAAGGAAAAAAATTGGGGCCGCCAGGGTTCGAACCTGGGCGGCACGGTTTGGCACAAACCAACCAGGGACAGAGCCAGTGCGGTACGTTTCGGGATATGTCAAATCCACACGAC

>FLPB003182N12-g0RSP_20020509 (FI167504 )

ATGGTGACTACAGAACCATGTAATTCTACAGCATAAGTTTTGGTAGAACAGTAAAAAAATAGGCATACTCGGGGCTTGCCCTCGCATTTGCGAGGGCCATCCTGCTAGTTTACATAAAAATATGTATCTCTATGTTTTTAAGCACCCTATAAGATATTGAAAAATATTGCCTGGTACCATACTATTTAACCATGAAAAGTTTCGCGGAGAAATGAGAACACTTTCGAATTTCTTGTACAAATTATAGGGTCTATGTAACAAAGTAGGTGCTAGGGATCTGAATAATCCTGCATAAAATGGACATTTTGGCATACAAAGTAATGTCTGTTGTAGCTTACTACACTTCTCCCGCATCTGTGCATGGAGGTGGAGGGCAGGGCCCTCAGGTTAGTGACGAGAAAGGGGAAGGGACGAGCTGCTGGAGGTGGCGGCAGTGGTGCTCTTCGACGTGTACATGTCCATCATCACCTGGTCTGCGTGGTTGTTCATATGCTCCCATGCCTACAGTGCGGCCACATTATTCAAGCACCTC

>FLPB002425F13-b0FSP_20011203 (FI167506 )

GTCTATTTTTTTTATCAACGAGGAGAAAAATCTCTATACAAGTTTTTCTTCCAATCATTTCTCATGAAATAAATCATCAAGTATTGTGCGTTGGATATCCTAAACAACCAACACATCAATTTCCCATAATAGACACACCGTTCAAACCTTCCGCAACCAGTGGATACACGTTGAACCATTTCCTTGCCAAAGTGTTCAACTATGTTTTCATATGAATATATATAGTATTGATTGTCAAAAAAACCTTGAAGTCAAATACGTGATTTATTTGTTCAGATTTGTTGGTTGTTTGATTAATATAAATTGATGAAAGCATAAATTCATCTCTCAACGTCTAATAGAAGTCGAAAGAAACCGTGGAGAATAAATCTTTTTAGATGACCCTATCTTCATTTTTGACAGACGAATATTGTAAGTATGGTAGTAGATATGAATATTAAGCCCTTAGAAATTCAAATGTTACAAAAAATAAATCATTACCTACCACAATTATATATGATCACTTACTCACTACAATTAACTAAAATTGCAGAAAATCATCAAGTATTTTTTGCCACACTAAAGATAGGGCCCTCGCATTTGCGAGGGCCACCTTGCTAGTTTAAATATAGAAACAGATTTTATTTTACATAGAAAAGGGCACAGATATAATTTTCGAAAGGGGGAAGGACCCCGGGTTTGATTTGCATAGCTGCAGGGGGTTTGCAGAAAGCGGACAGGCC

>FLPB002360F15-g0RSP_20011109 (FI167507 )

CGTAATATCTATGTTTAACATTAATTGTGCCAGGGAATCAGTGAGACAAAAGCCTTCAACACAAATAAGATGAAAACCAAAAATTGTCTTATATTTCTTAACGGTGATAAATTCTTTTTTATATCATCTCGATAAGCTAATTGGTTTTGTTAATGTGTTTGAAAAATCAAATGTCCTTTGCCAATGATTAAGGGAATCCTAATTATCATCTGGAGCAAACCATAATCGAAAGGAAGAATAAGGAATATATGTTTGATCAACCTTTTGATTTTTCTATTCACTAATTACACATATGACTTTTACAGGGATATGAATACAAATTTTTGATGACATAATCATAATTCACTACCANACTATATAATTTAGCACACAAGTGACAATATATTTAGCTTTACATTACTATATATCACTATAAAACTATATATTTTCCTTCCATCGAATGTGAATAAAAAATATACTAGAAATTTGGTGAACACATTTCCAACAAACTAAAAATGATACCCTCGCATTTGCGAGGACCACCTTGCTAGTATATAGTAACAATACAACATAGCTACATGAGGCAAATAGTTGAAGCGAAGCGGAATTAATGTTCCGCTACTGTGCTCCCTACGCTACTACTAGCTAGAGGCATATACAAGCATGTGTTGCTTTTTTACATCTCATATGGAGTAGAAA

>FLPB002231G15-b0FSP_20011109 (FI167508 )

AACATAACATTTCTTTACGAAAAGCAGCAATGCAAAATCACATAAAAATTGATGGCTTAGTCGCCGTATAAACTTTCGTTCTAGGTAGTATTTTTTGCAATTTAAATATAAAAAATATATGGTCATTGGTTTGCTATTTTAGACACAATTCACCACAAAATTAAAAAAAACTATGTGTGGCAAACATGCGATAGTATATTTATCTTTATATTAAATGTAACTATAAAACTGTATAATTTCCTTGCCACCAAATGTGAATATCAAATAAACTATCAGTTTATTTCACACAAATCCAAAACACTAAAAGTGACACCCTCGCATTTGCGAGGGCCACCTTGCTAGTTCAAACAAAAGCAAAAACAAAAACATACAGACGCTCCAAGTAAAGTACATAAGATGTGGCCGAATAAAAATATAGTTTCAAGAGAAGGAACCTGATAATTTGTCGATGAAGAAGGGGATGCCCTGGGCATCCCCAAGCTTAGAAGTTTGAGTCTTCTTAAAATATGCANGGGTGAACCACCGGGGCATCCCCAAGCTTAGAGCTTTCACTCTTCTTGATCATATTGTATCATCCTCCTCTC

>FLPB001068J09-b0FSP_20011203 (FI167509 )

GATCATAGAGTGGATGTAAATATTTTGTTGAAAGAAGGGTAACACCAAATTTGTACTTTAAAGATGACGCCCTCGCATTTGCGAGGGTCACTGTGCTAGTTAACTTAATATCTAATTTGGTTGATTTTTAATAATTATTTGAATAGGGAAAAGTCTCTGCTTTCTTCTTTTTATCAGATTAATTCTACAACTAATCTTGACATGGGACCAGTGGCATATTGTAGATCTTTTTAGTAGCTTTCCAACAATATAAAATTTGTTGAATTTGGCCAAGCCAAACAGATTCTATTGAATTTCAAAGTTGGAACCAGTATTGAAATTATTTGAATTCATTTAAACGAGATTTAAATAAAAGGGCCGGCGGGAAACTCTAACGGGCCAGGTTTTCAAATTAACGGCCTGCTCAGCCCAACAGCGCATCCAGTGCGAGTGGACTGCCTGACCGCGTGGGCCCCGCATGTCAGTGGCTCTTAAAACCCGAATCGGTACATTTTAAAGTGAGGCGTTGGATTAGAATCGAATCGGACGGCTCACGCGCATCATCGTCTCTGACGAGATGCCGTGGCGAC

>FLPB002096F15-g0RSP_20010827 (FI167510 )

ATACATACAAGAATGCAGTATACGGTATTCACCCAAACTACCCATGATCGTGATTGGTCCTATTAGAATGGTGCAAGTTTAATCTTTTACACATACATCCACGCAATGGATGTATATACGGCAAGGGTGGTCCGTGTTTGATGCAAAAAAAAAAATCGTAGAGTTGTTTCTCAACCTTACGTGTATTATTAGGATCTACTAAATATATATATTCCAATATTCGATATGACTATATGAATAATGATCAATAAAGTTCAGATGTTTCACAGTAAAGTAATAAAATGTTGCCATAAAAATTATAATAATGATTTGTCATTTCTTAGAAATAAAATCATATACAATTTCATTCAGGAAGAAGGAGGTAAATGGGACGGAAAGAGTTTGTAAAAAGTAATACAGGTGATGCCCTCGCATTTGCGAGGGCCCCTGTGCTAGTTTAAAGTTTCTTGAAATTTGATTATATATATTTTTTCTTTTTACGTGAGTTCGGTTTGGACGGTAGGTGTATTTCGGCGACCAATTTTTTTTTTAACGGTTTGAGTACGACAAGCAGGCTCCCGC

>FLPB002092P08-g0RSP_20010827 (FI167511 )

TCATGATGGTTATCGATTATAATGTGATTGTTCACGAGATAATTTGCTTCACAAACACTCAAAACTCAGTAAAGATGTTGCCCTCGCATTTGCGAGGGCCGCTGTGCTAGTTGAATGAAAGTTGCGTACATATCTGAGGATCACTCACGTAAATTGGCAGATTTTTCTGAGTTACCTACAGAGAATACTACTCAAATTCGTGACAGCAAGAAATCTGTTTCTGCGCAGTAATCCAAATCTAGTATGAACCTTACTATCAAAGACTTTACTTGGCACAACAATGCAGTAAAATAAAGATAAGGAGAGGTTGCTACAGTAGTAATAACTTCCAAGACTCAAATATAAAACAAAAGTGCAGAAATAAAATCATGGGTTGTCTCCCATAAGCGCTTTTCTTTAACGCCTTTCAGCTAGGCGCAGAAAGTGTGAATCAAGTATTATCAAGAGATGAAGCATCAACAGAGGGGTTTGGAGTTTTCTCAACTATGCATTGTATCTTATCTTATGTAAGTTTCAGAGGCTCCTTTTTCATTATTCTAGGCTTGTTATTCTCATCAAACAAATTTTAGGAACAATCCAATCATAATTCTTTG

>FLPB002584F15-g0RSP_20020220 (FI167512 )

CAGTCTTTGTTAAATTTATCGTTATAGATCTATGTGAATTCTACCTTTTTCTATTTCGCTTACAAATACTAATACATTCAAGAGTCTGCCCTCGCATTTGCGAGGGCTGCACTGCTAGTTGTTAAGTAAAGTACATTATAAGCTAGAATATTGATTTCCCATTTTTCAACTCAGAAAACACAGGGCTATGAAGTTACTCGGGGTAGCCAGCATAATTCAACCCACAGGAAAATGCTCTTCAGCTCAGGATTGAAAACCAACACATCAAGTGACCGACTTCCTGATGTGGTTCGACAAGCAAAAGATAGGCTTCATCAAAGACTTCGAAGTGTAGACTTGTCTTCCGGAAGAAGGTCCAGATAACCAATTTAGCTCGCACTGTTGGTCTACTTTAAGCACCAAATCTATTTTTACAACAATATATACGCAGTTCTACATTGATTCGCAATTTTCAGTTTCTGCAGGCATAC

>FLPB002683H03-b0FSP_20020409 (FI167513 )

CGATTTTGTAATAGAGAGCGCCTCACCTCACAGATTTGGACAAAACAGTTGGAGAACCAAATAGTCGGAAGTTGGACTGTGAGGATTGGGGACTCACACGTGTAATGTAGGCAAAGGGTGCCACAAAGTATTCTTCTTGATTTTAATGTGTATCAAGAACTTTTTATTTCTACACTGACTCGGTGATGTAGTCCATCCTCCACTATTTGACTATTTGTGTATGTCTATCTAACTGTATATATATATAAATATGTCGAAGATTTATAAAAATTTGGATATATCTATTATTATTTAGTGTCTAGATACACTCAAAATTTGATAAACTTTCGACATGTTTCGTGGGACAGAGAGAGTACATTTTATACGCAAGTGATATAGATTTAGCAAGAAATGTCGCTCTAAAATATATTACAAGCCATGAAACAATGTGGACAAAAAAATCAATAAATGTATTTTCATGAAAAAATTTGACACCCCGCACAATTTGCCATCTCTCTTAGAAAAATAAAGATGATGCCCTCGCATTTGCGAGGGCCGCTGTGCTAGTTAACATACTAAATGTACAATATTTATAAAGTTAATTTAAGAAGGAATGGCTTTCTATGCTTTTTCAGGAACGCGATCAATGCATCAAAGAACTTACTTGACATTGTGACCTTTTGCTTATATAGGCATCAATTGAAA

>FLPB002563K09-g0RSP_20020220 (FI167514 )

TTTATCATGAAACAATGTAGCGATACTGATATATTTACAGGAATATCTTTGCATGCTGGTTTGTATTTTGTCAAACTCCAAGTTAATTATCTGATCCAATAACAAATTAAAGAAAATTAACAGGCAATGGTTGACACAATGTATTGTGACCTTACCTGTCCAATCCACGATTCCTCTTCAGCGAATCAAGGAAAGGCAAGTTGGGTGACTATTATATTGCTAACGGTGTGTTTTGAGTTTTATCAAAATAACATGCCAGACTTTTTAAAGGTGGTTCAAAATTGCAGATGGTGACATTGAAATCAAGGTGAATTCTTCAAAAAAAATTCAAAAGTGAGATCAGGATTGGTGCATTTGTTTCAATAGTGGAGTATTTTTTATTTCCCAGTAGTTATAAATAATACTTCATGAGACACCACTAGAGATGTCCTCGCATTTGCGAGGGATATTGTGCTAGTTAACTTTAAGATGGTCTTCTTGTAATATGCAAAATGTCCTAGATTCAGCTAATATATTGATGAAATGTAACTCAAATCAACACAAATAAAAGATATAA

>FLPB002417H11-g0RSP_20011203 (FI167515 )

AAAATAGTTACATATTAAAGATGATACACATAACCTATAGACTTATTTTCACATAACCTATAGACTTATCTCATTACTTAGTTGTCCTACCTATTAATAGATGACAAGTTATTTACTATTAATATAATCATGAACAAATTACTTTTCTTTTCTATTCAGAAAATAGTTACATATTAAAGATGATACACATAACCTATAGACTTATTTTCGCATAAACACTACAATAATGCCCTCGCATTTGCGAGGGCCATCTTGCTAGTTTCTGTAACATCCCAAAAATTTCAAAAAAACAAAGAAATGAATTTCCCTATTTCCAAATTTTGGAATCAACAAAAACTTTTATTAATTTAAGTTTCATACATAGTGATCATGCTTAGATGTNGTGATATATATTGCCATGATTGTTTGTTTATTATATTGAACATAATTCCAAAACCCAAAACCCTAACCTTCTCAAAACCAAATAGGTCAAATATGAAGAAACTAAAATATAATAAAAAGACATATGTGGGCATATAGCCCTAGTATGCAAATCTTGACTAATACCCTTCTTACTTTTCTAAATAGTGGTGAACCATTGTTAGACCCCACTAAACCCTAAACCTCACCCCTCTTCTCA

>FLPB002296K07-b0FSP_20020513 (FI167516 )

CAGAGCTTTGAAGCATATTCCTTCAAGAAAAAGAAAAGGTCTCAGAGGATATTTTTTGCGAGATGGATTATATATTCAAAGAGGAGGATAGGCTAGATCCTTCCTAGACGATTCATCTAGCCATGCATAACCTCTAAAGTTCCACACTAACATAATAAAATAACACTGTTACAAACTCCTAAAAGTATAATTATCTATATTAAACATTTGGTATGTATGGTCTGATAATTTAAAAGTGATTATACTTATAGAAATAAAAAAATTTACAAAATTAAGAAATCTTGCATTTAGAAATGTAATATTGCTTTGTAAGACTTACATATTTAGACATACATATTTATAAAAATGGTGGTTGTAAAATGGATTATTTCTATAACATAATGTGAATTCCCATGGATCTATAAGTGTAGTTCTCGGTCAATTTCGACATACAATTGACAATGCCCATCTCTCTAGTGATACTAAAGTTGATACCCTCGCATTTGCGAGGGCCCTCATGCTAGTTAGCAAAATAGTACAGGTAATAGAGTTTTTACGTTTCTGGAGGGGGCACCCCCACTCGATCCGCCCCTGCCTGTAACATCTAAGCTTCAAATAATTTATGTTTCAGTCGGTTGGTTCCCTACTGAATCAGCACGACACATTTCCTAGAAGCTGATAATCCTTTATCGCGCTTGTGGCCGGTGGATTCATACGGAC

>FLPB001048O11-g0RSP_20010815 (FI167517 )

CAAACTCTTAACACTCTAATAATCTATATTAAGCATTTGGTGTGAATGGTGTGACAATTTAAAAGTGACTACACATACAAAAATAACAAAAATTATAAAATCTAAAAAATCTCGCATTTAGAAACATAATATCGCTTTATAAAAATCTATTTAGACATAGACATTCATCAAAAAAAAAATGGTGGTTGTAAAATGGATTATTTATGTTACATAATGTGAATGCCCATGAATCTATAAATGTAGTTGTAGGTCAATTTAAAGTTTTCAACACATAATTTACAATGCTCATCCAATGATTGTATGATACTAAAAATGATGCCCTCGCATTTGCGAGGGCCCTCATGCTAGTTGACCCGAAAAGGTTAGATCGAATCCATTTACTGTATCAAGCTGAATTTGTCGCACAGCAATACACCATTTCAGAGCGACATGTCGTGCCGATCGAGAACTCCAATTCGAACTTGGCCTATCCAGCTCGATCCCCCTGCGTACACCTATCGATTGTTTTCGCCATGATATTGGCTGTCTGATGCATGCACGATAGGGACGCGATGGTACTTTGTA

Type 3

>FLPB002031P06-b0FSP_20011203 (FI167518 )

ATACCAGCCGACAGAGGGATGTCCTTGCATCACTTTAAGTAGCAATTTCGATCATTCACTAGAAGCAGCTACAACTATGTATGCCAAAAGGTTACAACACGGGCGCGGCGTGCCGCCGCGCCTATGCTTCCTAGTTATGAATCAAATACAATCAAAAACTTGAGACCTTAATGGGCTGCTGGTACATCTCTCCTGTGGGTAGCATTAGGTTCCTGATCTATCGTTCTGATCTCTCCTCTCCTTCTGCCTCTCCCGGACCACAAAGTTCAATACCTTGTCGTCGTCTGTCGTATCTACTTTTGTCAGAAATCACATTTATATCCAATACTAAACCAAAGTATAAAATGCATAGCCTCATTTATTGAGTAATCCTGCATGGTTCAGAAAAAACCCTCGTTTATTTGGTTTGAGTAGATAAAATCCATAGGA

>FLPB002681C02-b0FSP_20020409 (FI167519 )

TGTAAATATCTTCCTCTTTCTTAAAGTAAGATAAGGCATCTTACTTTAAACAAATATCTAGATTGGTATCAGAAATTAATCCTACTAAATTTCTCAATTGTTACTCTTTCATCAACCCTTCCACAAGCATTCAAAATGACTACTAAGGCTAACCATGCATGCTAAAAAAATACTAGCACGGGCGCGGCGTGCCGCCGCGCCTATGCATCCTAGTACATATCAAATGAATGTGCCCGACGGATGCACTTGTTTCATTGACCAGTGCAACGGAACAGGCTGTATAGCAGACACCTGTATATTACTTCCATCGTAGTCGAATGCATATGCCCGAATTGTTGAAGTATAAAATCGGTATAGATAGATAGCTAAGATACCATATGTGACTTGCACGTTCCTCACCGATCCGATCGTGTTGATAATTGATATCTTACCTACTTGTATCAATCTGATTGAACGGTTGTAATCCTGTTATGCATATATATAATGAGAGGAAGGGCCTAAGGGTTGACCACGGCGAAATCATTCGTTCAAACTCGGATGATGTAATTACTTGTTGATTCATTGATCGATGCAGTGAAACGTCTTGGCACCTAAAACATATGTTTATCATTGTCAATAACCGTCTATGTCTTCCCTCCCGGAGGATGTGTCGGGCCTCTTCTCCGCCACTCAAGGATGCCTCAGCATTTGGACACGTGGTAGACACCTAAAACATATTTTATCATG

>FLPB002531K09-b0FSP_20020220 (FI167520 )

CATAAGTACAATCAAGTCAGCTCAACGTGAAGGGTCCAAGAGAAAGTAGCTGCTCGACTGCTGAAACAAATAGTTTGCATGAAGCAACTACTATGTCGACATTAATTTAAAAAATGTCGACATTAGAAGCGGAAATATCATGCCTAATGTTGAGTATTTGCCCCATACTGTGGTCTTAAATGCCTACCAGATAACTATGTATAATTAGTACTCCCCGTACCGTTTTAATCTACTCAAAGTTTTGGCTACCAATGTATGTTTCATGCCATGGCCTGCGTCGATTAAATAAGAACTGAGCATCTGATGAAGGGAGTAGATTGTATTCACTCAGAAAGGAACTAAAACCAAATATACATACAAGAAATGTACCCACACGGGCGCGGCGTGCCGCCGCGCCTATGCCTCCTAGTATCATTTAATATCACTATATATGATTTAAATCTCAAGTAATTCATATAAGAGAGTTTGGAACCAGGGGTCCAAACATCCCATGAATTCATGTGAGACAAGTTTAAATGAAGTATAAGACTCCACATAATTTACTTTAATAGTTTTGGACAATATCAACTAGTTAAACTAGTCAGA

>FLPB002671O18-b0FSP_20020409 (FI167521 )

CGTAGAACTGCCTGGCTTAGATTTAAATCGGCGACCCGCAAGATAGTTCAGTAACATGTACAACCTCCACCATATGGCGCTGCTACATCTAGATAGTTTATCTGCCAAAGTTAGTTTCAGACTTCCCAGTCTACAGTTAACTTTGCAAAATCGCTTTTCAGATATCACGGGCGCGGCGTGCCGCCGCGCCGATGCCTCCTAGTACCGATGCAAGGATGCAAGGCGAGCCTGCAGCGGCCAACTCTCGCCGTGCATCTCCTGAGGTAATAGCTATTTTTTTATGACGCCGGCATCTCTAGGAGCGTTGGCCTGTAGTTGATTAGCCTCAGGTCTGATCGATCTGATATTACGGGTACAGGTAACCTATGCCATTGCTTATCTCCTCTTTCCGATCATATCCGGCCGGCAGAGAGCTTCTCCTCCACTTCTAATTAATTTTCTCAACCATGGTATGATTTTGATGTTCAAATTCCATTCATG

>FLPA001039C09-g0RSP_20010509 (FI167522 )

TGCTGCTTATTTACCAATTATGTGTGAGTACATGCTTCTAACTTCACCTCACGCCATAAACGGAATCCCAATCATGAGCGCAACTGTATTTACTTGTTCAGTTGCCTGAGTGAGCTTTCTCCACAATATGGATGTGGTTACTGTTCAAAATCTTAGTTATTATTACTGGACTTAATTTTTGTGTGAGTACCAATTATTATTACCAGAGTTAATTTTTGTGTCTTTTGATGCGCTTATTTACACCATTCTTAGGATCAACTTTTTAAGCACGTAATTGTATAAAAAAAAATTAACCACCTTAAAACACATTATTTTAACAACAAAGTATTCATTACTTGTATCAATTCAAGTGTGGTTTTAAAACTATTTACACTATATCATTGTACAATCGGCGCGGCGTGCCGCCGCGCCCATTTATGCTAGTTTCAATAACAGTGGCATGCAATGCCGCTAATCTGGTGGGGCCAAGTGGCTTAGCCCTGGGCTAAAACTGGTATCTCGTATCCATGGGTAGCTCTGTGGGCTATGCCACATGTTTTTGGATGGCCCACGCGCACCCTTAACTGTCGACGCGCCACA

>FLPB002430B21-g0RSP_20011203 (FI167523 )

ACCATATTTTAGAAAGGGTAGAACCTGCTTTTCTCCATGCACTGTCTATGCAGAACAATTGCATCAACAATGTAATCAGCTCTAATTACACATTTGTGCACATATTGCTTATGTCAGACAATCTCAAGGGCTATATATGTAATTAGCTCTTATTACAAATTTGTATTACCTACAACAGTCGCTCTTGTGTGTAAATTGATTCCAATCATTTATGAAAAAAAGAGAAAATTTCTTTAGCTGGATAAGGGCACACGGCCGCGGCCGCCAGGTGCACATCAACCTTATATAGAATGCTCTATGTCACATTCTTTAAAACAATGAAAGAAAAAAGGTAATGGAGGTGAATGGTCAAGCTGTATAGGCTAGGAGATCAACCTAGATTATGGTCATTGAAGATGCAATAGAAATACAATCTTTTTATTCTTGGGCTCATGTTTGGCTCATGTCTAAAAGAAAGGCGCGGCGTGCCGCCGCGCCAGTTCTGCTAGTGTGTCTAATGGAGTGTTCATAGAAGAATTATCTAACACTCACAAATTAAGAATATTTAACAAGACGTATGTACCTTGGTGCATCTCACTATCTTAAAT

>FLPB002716D15-g0RSP_20020409 (FI167524 )

AGATCAAGATGCTGCCATTGCGACTGATGGACTTGACCAACCTCTATGTTGTATATCAGTGTAGTATTTTATTTTTGTTAGAGACCCTTTCCATGAACTATATTGTAAGTTGCTACATTATGTTGAATTGTAGTATGCCAGACTATTGCTCTCTATAACCTTATGCCAGTTCTGAAGAAACAGTCTCTGTTCTGCTAATTTTGCTCATATCTAACTGGCTATTTCCGGTCCACTCAGTTCTATTGCACATCTATTAAATTTTCTGTCTTTTAGTTCATTCCCTCATCTTGATGTAACAAAAGATTGGATCGAAAATTAGATCACTAATAGTACTAATTATAAAGCCTGATGGCATTTAAGTTATCAAAATTGTTATACTGCCATTAGACGAATCTTGGAACAAGGTTATTTGCCATCTGCCTTTATTCTACAAATTTGTATGCATTAGTTCTATTAATTTTTACCATGCCAATGCTTCCATACCAAAAAGATAACACCACGGGCGCGGCGTGCCGCCGCGCCAATGCTTCCTAGTATGCTTTACCGCTAGTTAACTGCACCAGCACATTCGTTATTGAGGCTCACGCGGCATCTCCAGACTTCAGGGCTATGTCCAACATAGCGTGAACCATTAGTCAGAGGCGGTGATGCTCAGCATAGCGATCGGACGGTATCGATAAG

>FLPB002234D08-g0RSP_20011109 (FI167525 )

ACATATTACCAATATATAACATTATAGCACATGGGAGCATATGCTCCTGCCACCGGATTTTTTTTTTGAAATATCAAAAAAATTCGAACAAAAATTTCACGGGTTACTTATAGCACTGCTACTACACACTACCATCTCACGGGCGCGGCGTGCCGCCGCGCCTTTGCTTCCTAGTAATACAATACAGAAGAACCACTTGCGTTCGTCACAAACTATGGACAGATTTAAAAAGAAGAGAGCACGGACATCTGATGATGAAACAACATGCTTTCTTGCAAA

>FLPB002412K08-b0FSP_20011203 (FI167526 )

GTGCCTTCACAAATTTAGATTATCCACTATATTAATACTGAAAACAAATTGAGTACATCATATCCTCAACAATTACAAAACTATGGTTTCAAAAAAAAAACAATTACAAAACTAAGATCATTACGGGCGCGGCGTGCCGCCGCGCCAATGCTTACTAGTATACATGAATGAGAAATGATATTATTTCATCGGATTCATTTGTCCTTTCTAGGCCTCCATCGATCTAAGTACGGTTCTCAGAGGAAGCACTGGTGATAACATAAGTTTCAACATATGAAACTTCACTATCTCACATTTAGTTCGAGCAGTGCCAGCAGCTTCGAAGCTATGCATTTGGGTGGTCGGTGGTTCGGTCCATTTTGTGGTTGGTGCAGCATCGTTTTTATAAGCTTTGCATGCTCATAAATCTCTTGCCACATACGGTTACTTGTCTATTGTTAGGATTAGATCCACGGTATCAATTTATGGATACCTCTATATAAAGAGTGCGTTTTATATTTCTCTGGCCTCCGTTACTTATCGTCTCCACTATCTCTCTGGCCTCCGTCTTATCTCTCAAACTAGCTTCACCTACCCGGCCTACGATATAATG

>FLPB002008E21-g0RSP_20011203 (FI167527 )

TGAAATGCTACAACAAATTTGCATGTTGCCAGATGATAGTAAACTGAAGACTATCTCCTTTGGAATTGGTCTTGTGAACAAAACAGAGGGAATAGTGGGCAGCGCCATGAAACAATTGATGATTTCAGCTCACTATTAATAGGCATGATGATCAGTCCCTGTGATGTTAGTGAACCTTTACATCCAGAAACAATGACACTTCTTAATTCTTTCCAGGTAACTAAATTTAAATAAATTGAGGCCCACATAATTCTAAATGGACCACCGTATTTACTAGCCCACGGGTATTTACTAGCACACCAATTGTGTTAATCCAGTTTTATTATACATATTATAATTTAAGTAGTGTATCTATGTCTTTTCACCTAATTTTCCGTCAGACAGCCACTATACTGGCGCGGCGTGCCGCCGCGCCTATCTTTGCTAGTTCCCTCAAAGGCTGGATAAATTACTTGTTTAGACACGTTACAAAAACAAATTGCGCCCCAAAGAGCATGTAGGCCCTGCAAAGTATGTATATTTATGAGAGTTTATGTGATGCTAGATAGGGAAAGGGTGAAATATCAAAATTTTATTTATATTAAACTATTGTACTCTTTCTAATTCATACCATCACGAAATGTATTTCTATTTTAATGGATATATCATTCAATACATTGGTCTTCACTAAGAAAAATATCCATGACTACATGATTCAATAGGTG

>FLPB002303N16-g0RSP_20020424 (FI167528 )

ATCTTGCAGTGATTATAATTAGCACACAATACTAGCAGTATCGACAATAATGGACCTCCGTCAGCAATTTTAATCCATAAGCAATTGTACCAAAATCTAAGTGACATATTCATGCCATCTCACGCATTTTCCAACAGTTAGCCACGGCACAGGCGCGGCGTGCCGCCGCGCCAGCCATTGCTAGTTGATTAATCAAGCGGCCAGCCGGGAACGCGTATGTGAACAGAAGAAACCAATTGGTAAATACGTTGCTACAGCTTCACTACTGGTTGCCCACACCACACACATCCAGCAGCTGCACAGCAAGTCAATGGTGGTGGCAGAGGAGAAGCCAAATTGTAAACGCGGTTTTACTGGGTGGGCGGCGAGACGACAACACTCGGAATGGTTATGGGCGGAGAGACACACAAAGTATCGAGATCTCCTCCTCGC

>FLPA001042H10-b0FSP_20010509 (FI167529 )

ACTATAGATTTTGACTCATAGTTGTGTGCATCAGATATATATTCATGAAATTTTTATCCATAATCACTGCCACATGCATTGTAACCGCTATCGTCTCATCGGCGCGGCGTGCCGCCGCGCCACACCTTGCTAGTAGACTTAAAATTGTGAGGATTGGAAGAGACAGGAAGTAAGAGATGATCAGTGAAATAGGAGGAGTGGGACATGAACCGGGAGAGACTAGGAAGAGTTGCGCATGAGTAGAATAAATGGCAAGGTAAGTGGGAAGGGAATATGTGAAGGCGCCCACGCATGGTAGGAAGACGGGTTATTTCCTTATATTTGCTGATCATCAAATAAAAATCTAAACAGAAGATAGAATAATTTGCTGATGTGGCAGTTTGCTGAGGTGAATAGGCTGCATAGAAATAGTTAGTGGGGATGAACTATTTAGTTATTATAGATAAGTTATTCTTTCATTAGTTTGAGTGGTGTTTTGGAACATGGGTGCGTATGCTCCCTATATTTTGAAATGCATCTTATGTACA

>FLPB002039G22-g0RSP_20010827 (FI167530 )

TAGGCTCGGTACACACCTTTGAAATTGAATCACAAGATCGACCATTGATTCAAGTATAGTCTTAAAAATATTACACCAATCACATTGTAAGGTCGGCGCGGCGTGCCGCCGCGCCCATCCATTGCTAGTACTATATATACACATACAAAGGAAGCGTTTGGTAGGCTGCATGTCCCTTAGCTCGCATCGGCCCAGCTTTTTCGGT

>FLPB002719F03-b0FSP_20020409 (FI167531 )

GATGCAATCTGGCAAGTACGTCTTATAACAATCTATATGACACTACTGACACTACGAGCGTATTAGACAGTCGGCGCGGCGTGCCGCCGCGCCCACTCCTGCTAGTGAACTATTACATCGAGACAAAGAAAGACTCCTAGAGACGAAGCAGTTGCTGCGGCATGGTCTCTCAGACGCATGCAACTCCAGCGGGCACCGCCGTGGCCTTCCCGCCGTCCTCCAGCTTCCTCATCTTGGCGCGGTGATCGTCGGCCGCCGCAGAGGCGCTCTCCCGCCTCTTCCTCTTTGCCTCCTCTGCGGCAGCGTCGGCGGCCGCCATGTCGAGCAGGCGCTGCACCATCTCCATGGCGATGTCCATGCCAGGGACGTGCTCCTTGACAACCTTGTTGCCCTCGAACGCGGCCGCGGCTTCCCG

>FLPB002058G04-b0FSP_20010827 (FI167532 )

TTGAGTACCTTCAAGGGCACCACCTCTTGGACAGCTTACGCCCTAACCTTAAAAATACCAACTCGCTGCGACTTACAAAATAACGGATTCTTACTAGACAATAAAATTAACTAGATAATTACCATGATCTGGCGCGGCGTGCCGCCGCGCCCTTCTTTGCTAGTATTAAAAAGAGAATAATCGAGAAATCAGAAGACACGAAAGTAACTGCAGTAGTGTAAATCAGAAGCATAGTCTCTAGTAATTTAGAAATATAACATAGTTTTTTTTTATGTGTACATGATGCGGGTAATCACAACAATTTACGGAAAAATTAGTTTTATTATCTAGGGCTGATTTAAATTAGATTTTAATTTTGTGGCATTCTAGACTGTAGTTATGTCGTGAGATCATTTACTGTAATTTGGACTTATATTAGTTAGATGATTGCACTTAAAAGGCTATTAGATTGCATTGTAGTTGCTCCGACAGACAGATTGAGTTTATATGAATATTCCCGAGTATATGAATGGAACTGAACGTATGTCTAGGATATAAATGTGCAGAGGACTGAAGATAAAATTCATAGTGCCACAACAAAGTAAAGTGCAGATTCTTAAGGCTCCTCTATGTGTCTCTACGTGTGATATTTCTCCTCCGGTGGAGTGCCTCACTGTATCAGCAAAACAGATTTATCGTAACCAGTGTAACAATAGTGCATTTTCTTTTGGATTACA

>FLPB002360E09-b0FSP_20011109 (FI167533 )

GTCTATGCACAGTGAGATTTTGTTACGGAGTACCAACTATCTTAAAGCACACTGTCCGGACAACAAAATACACAATACTTCTATCAATTCAAATGTGTTTCTAAATCGTATCATACAGTCGGCGCGGCGTGCCGCCGCGCCTATCCATTGCTAGTTGCTTTTAATCGAATACAATATGACTGGAATCTCAAGATCCACCCAAGCCTCTCTTTTTTTCAGTTATTCACATTTCTGGCTCCAACAGCTTATCCATATCGAATTTCTAGACTAAGAAAAAACATAGCGATTTTACACCGTTTAGACATTGAAAAGAGAGTAGTATTTCTGTACTTACAAAGGAGTCCAAATAGAGAAATTGAGATACAGTAGAAATCATGTAACCAGTTACCAGTATCTTCAGGAACAAATAACATAACGGTTTACATAACACAGTTACATCAAAGAAGGTCCTGCAATCAACATCAACATCTGCATTGTACTTGTACAGGTAGTAAACAGTACTACTAGTAAACAATCCATTACCGTTCACAGATGGCAAACCAAGAGGGAAACCATTACTGGTGGCCATGGCCGGACGTAGAGGCTTCATCTATTGAGACTGTTGCAGCCTGCTTGCCGTGGCCTGCCGTGGAGGCTTCGTCTCTTGAGATTGTTGCATTCTCCTGAATTCCTTGAGCTTGTGCAGGGGGGGCAGGCCCGGAGGCTGACTGGGCAGTAAACGGCGACAGGTGCGCGTAGGTGCCCCCGATGTCTGTCCTGTTAATGCTTTCCCATGC

>FLPB002456M18-b0FSP_20020220 (FI167534 )

GTCCATTTCTTGGATCAAGCAGGCTCCTAAAAAAAGTCCTCATTCAAGTTTGTGCTTCTTGAATCTTCTTTGGTTGGCAATGCAGTAGGCTGGATCTGTATATTTGCATTCAGTATATTTCAGTGTTATGGAAATCGATATCTAGGATGGTATGCATTGTTAGAGATGAACATATTATCATGGCTGAAGCACGAACATTATCTATTGTCAACTATATAATTATTCATGAGAAATTACCATCAATTAGCCACCATATGGCGCGGCGTGCCGCCGCGCCGATCATTGCTAGTATAGATAATAATGCAATGACTATAATTATACTTCAGAATTTGCCCTGTGGTTCAGCGGTTACTTTTATTTGAAACTCTTATGCTTGCAATGCACCGGGTGGAAAATTCTGCGAGCACACATGTCTAGTAAGTTTGCTCGCAGTAACCACTCAGGTAGAAAAACAAAACCTAATATGTGGCGCTAAAGTAAATTACAAGTGCCCATGGTACGTTAGAGCACTACCTCGGGAGGAAAAACCCATGTCGACCTTGTATCGTACTCATGCAGCGGCTCAGAGCTATGGTCGTCAATGTCCCACTGGCCGACAAAGTAGCAGGTTGTGAGTTCCTTTGTAGGTGCAGGAAAACCATTGCAAAAGCGCTTGCTGCATGAACAAGCTTTTTGACTCTCTTCACTGCGAGCCGGAACCCAGGAG

>FLPB002438D06-g0RSP_20011203 (FI167535 )

GATCAATGCTAAAAACAACTTCAGGTCCATTTACTATCAGTCTGTTGATTTAAAGCATCCTTTATTCTTTAACTCCAGGCTGGGCGCGGCGTGCCGCCGCGCCTGTCCTTGCTAGTCCATGTAAGCCCAAATCGGCATTCAACATACTGCATCGACTCTTTTTCTCACGAGGCAAAGACAAAGAACATGCATGGACTATTAGTTTTCTGAGCCGTGTACGCCGTAGCAAGCCAAACCCTGCATTCAACACGCGTAGACCTCTCGGCAAGCAGCCAAACTAAGATCGAGCTGCTGGAGCACTCCATACCAAGAGTCAAGCGGTCGTCTAGTAGTAGAGAAAAGAAATGCTGCTCGAGAAAGCGGCTGGCGGCCGGCGGGATTGGTGCACCGTGACCTACCGCGTGGAGAGCACGCACGTAGCAGCCTAGGTGTCTCGCTCGTCGGAGCGATAGGAGTGGAGCAATGCCGATCAGGCATGGGCGCGCTCGAATAAACGATTCGATTCGCTTCGCTCGTGTCGCGGCCACAGCCACAGCCACAGCCACGCCCTAGTACTCTGCATGCATGGCGGACGATCGAGCAGCAGCGGCATGCATGGCTATGCTTGCCTGGCCAGGCCGTGACAAACGCCGCGCGTCAGCGCGCACCAGGCTCTGACTCCGGCGTCGGCGCCGGGGCGTGAAG

>FLPB002438G17-g0RSP_20011203 (FI167536 )

TGCTCAAGCAAACACGAAACGGTGCTCAACAATGCATGTCCATCACTGCGAGCATGAAGGCTATTTCAAAATCACACACACGTTCGGTTACATCAACTGATTTAGAGAAAAATTGAAACACGTCTAAATAGGTAAAAACAATCCATAAACATTCTGCACCGCTAAAAATTACTATCAATCGGGCGCGGCGTGCCGCCGCGCCTATTATTGCTAGTTAGTGTAATTGACATGCGATTTGTCTTGACAACTTTTGTGTGCGTGGATTGATTCTATTCTCTGTGCAGGAATTTTAAGTGCAGCAAGGAAGGAAGGACATGTTCTTGGTACAGAAAGTGCACCCTTTTACATAGTAGAGAAAGATCTAGTTGCAAAGCAGCTCCTGCGTACTTGTGGAGGCCTTATAGTCACCGGCGCTGTATTCTATGGCCTTCATGGTTTGAACAATGTGGGAGTTCTTCAGTCTGAAGATCTGAAAGAAGCTCCGGAGGAATGGAAAGAAGTGCCTACGGATTGGAGCACAAAATTCAGTGATGTAAAGGGGGTTGATGAAGCCAAAGCTGAGCTCGAGGACGTCATATGCTACCTACGAGATCCCGATGTGAGTGCCACATTGCCATCAACCTGCGTCCAGGTGTTTTAGTCCAGTAAATAATTCTTTCTGGATGCAGAGTTGTTACCCTAGTCTTACATGCTTTCCATGCAGAGTTTCACAACGCCTTGGTGGC

>FLPB003165B09-g0RSP_20020508 (FI167537 )

AGAGGAATAAGTACTATTATTTTGCTAGCTTATTCTTACCCATGAGCATTTACTTTTGAAGATTTAGTTTATTGTTGATAGATGAAAAGAACATTGCACATATTCCTTGATTATTTTGGAATCATATATTGTATTGTTCAGCTCTTCATATGGAATGTTCAAATAAAGATGATCAGTTTCTCCTTGGATTTTCCCACCATACAGAACAATGCCCACTCACTTCTAACTATTTTAACACATTTCTTATCGAGATTTAACATGTAAGCATTAACTTTGGGCTGGGCGCGGCGTGCCGCCGCGCCTGTCCTTGCTAGTAGAATAAAGAAAGAGTTTCAGTTAGAACAATACAAGGGGTCCATAGTAGGGGACGAAAATCTAAAAGTGAATATCACAGAATTCTACAAGAAGTTATTAAGGGCACCGGCTTGGATAATTTCTCTCTAAGAGAAGATATCAGTTCGGATATTTCACAAATTTATACCTTGGAAAACATTATGCTGACAACAGACTTCACCAAGGAGAAGGTTTCAGAGGCAATATCTCAAAAGCTAAAGGACAACGCTTTATGGGTAAACGCAAAGAAGAAAAATAAAAAGCAAAAGGGAGATGTATAGATGTGCATATGAACCTGTTGCGTGAAATTATATTTAAAATATCAAAAAATTCTGAAATACAATTTGCATGTATATCTACACATTCTATGCTCGTACATAAGTTTTTTTTTT

>FLPB002264I01-g0RSP_20011109 (FI167538 )

GGCTCAGACTTTCAACGTTCAATATATATAGCACCTGGGTCGTGTTGCTATATTATGACCCAAGATAGATCAGCAGAAGCCTCCATGTTTGTGTCACAAGCGAATGAGTAGCTCGGCAAATGTGTTGATCCTTTGGACAATCTGACGTCTGAGACGGAAAGCAAGCCACGAGGTCATAGCCCTCTGTCAACATATGAATTTGTGCAGGAGTTCATGGGCGAACCTGAGGTCGCAAGTAAGTAATTATTAGAAGAGTCATGTGTTATAATGAGCTGCAGGTGCACTTCATAGATGGATTCAGAAAATATTAACGCAAATGTGGGGAAGAACCGCGATCAGCACTTCGATTTAGAAGTAACTAAGACTTGATATGTAATTTATTTATATATATTTCATCAAAATATGCTTTGCTATGTAAACAGATTATTTACGCTGTATACATTCAGTAGCATCATGAGAACATTATATCGATTAAAATCATTCCACAGGCGCGGCGTGGCGCCGCGCCAATGCTTCCTAGTAGTTTCTAATCTTCAGTCAGACTAGAACTTCTAGTCGTTTTCTTGTTTACATTATTTGTGGGAAAATGTAGTGTAGGAC

>FLPB002672F24-b0FSP_20020409 (FI167539 )

GAGAGTGCTGTAAAAATATTGATAAATTCGCTAGCAAAGCCCATCTACCCAGACTACCAATATTATCAGCAAATCGATCCAGGACCCACAGAAGGCCAGACAAAATTGTCATTAGGCCGGCCTACCAAGAAACAATATTATAGATTTAAATTATTAGAGAAGCACATCTAACTGTGCTGCACTCCTTTTATTTACGATAAATGTTGATTCTATAAACAAAATAACTCGCTTTCTTACGTTTTTCGACAGTTACTCTTCAAACCAGCCACTCTGCAACCTATCCAACAAATATTCACAAGCGACCGATAATGCATACCAATCATACCATTGCATGAAATTTCCACAATAAACTCAAACGAAACTACAGAACAAGTACACATAAGAACCGAAGCTCAAGATTCCAAGACTCCAAAACTAGGTAACACTAATAACTCACACAAAAAGGTTATGGTCACGGGCGCGGCGTGTCGCCGCGCCTATGCTTCCTAGTCATATGTAAGTACATGTACACTTTCGAAAAAACAATACATTAGAACAATGTCTCTTTATGTTCCTGAATTCTCATGACAAGTTGAAAACATAGGCAGATAGGCTCATAACTGAGTACCGGTGATGACTTTCTATGATGGTCCGTATACCCAATACTTTTCCAGAATGTGTTTGTCAATGTATAGGTGACAAAGCTTTTGGCTGCTAACTTATTCGACTATTCTGCATTTACCAAGAACCAAACAACTATGTTGTCAAAACTTAACATCGAGAAAATAACTGTGTTTTCTATTTTAG

>FLPB002306C17-g0RSP_20020513 (FI167540 )

AATATAAGTCCGTTTTAACATCAACGCTAACTGTATGCATCAAAAAGGAACCACCACGGGCGCGGCGTGTCGCCGCGCCGCAAGCTTCCTAGTACTATATATAAAAGTTGAACCCATCTGCTGAAATCTCAGCGTTTTGGAGTTTTAGACACTGTCCACAGTTCCTTTTTATACAATTAAACATTGACGCATGGATCCTCGAAGCTGGGTTCAG

>FLPB002034C09-g0RSP_20020220 (FI167541 )

CTGTCCCAGACTTAGTGTACTTTGCTTAGACACTACTGTGATTAGTCTATACGTGTGTTGTATGATGGCTGTTAGATTTAATTTGTAACAGAGCCTGTTGTATTATTGCTTATGGTTATAATCTCTTCTGTCGAAAGAAGTTCATGTTTTCTCTGTTTTAACATAGCATGTTGTATCGGTCAGAGAAAATTTAGGTGTTACAACTGTTTACTTTGTATGATTTCTATTGGCTCACAATTTCTCTAATTATGTCTACTTATTTCATGCATTTTCTCCATATTGTATCAAGTTCTATTTTTTGCCCACAGTCTATCTACTGTGTAATTCCATGAATATCGTCCCACGGGCGCGGCGTGTCGCCGCGCCTAACCTTCCTAGTATGTATGCAAAGGTTGATGTAGTTTATCTTGTTCTGCTGAAGTGTGGCCAAGAGTAGTGGGACATATTTTTCGCACAATGTAAGTTAATACTATCTCTAGCTTGCTAAGTTGTCATCAGAGTACCTGAAT

>FLPB002466I21-b0FSP_20020220 (FI167542 )

CTGGTAGCTGGGCGCGCACTAACGAGCGTCCAGCTGTGACGGACGGCGCGTGACAGAGGAGAAGAACCCGTATGCATTCTGGTGGTCGACGAGGCGCTACCGTGGGAGATGGAAGGGATCGAGGAGACGCTGGCTAGCTTCCTGGGGTGGAGCATCATGTGTGGGGATGCGGGGCGGCGCCGGTGTGGCGATGAGGGTGTGAATTCCGATTTGTGGATGGAGGACAGTGTCTTCGGTCCTCAGGACAAGAAGGGAACTGCACGTGGCCTGCTGCCTCTAGATTCCGTAATATGTTTTGCAGTAATGAGTGGCTCCCATGCTTAGATGTCTTCTACATTTTTCTCACTCATTTTTGCCCCCATATTACTGTGTACTTAGCATACACTCGGGCAATTATACGAATCGCACAATTCATATATATTGTCACACCCCGCATATCTCATAGGCCTTGCAATCACAGCCACGGGCGCGGCGTGTCGCCGCGCCATGGCTTTCTAGTATTAGTTACATTAGGAGGTGGAATATTATATGTAAAGAAAACCATTCTTCAAACAATTTTTGCAACAGGACGAC

>FLPB001009O04-g0RSP_20010815 (FI167543 )

GGGTGTGTGGTGTCGGTCTGTAAGCTGGATTCAGCACTTTGTATCAATCTCGCCGTTGTTGGCTTTGTTAATTCAAAGTCGGGCACGTCCAGTGCCTTTCATCTAAAAAAAAACTTAAAGCACACAAATTCATACATGATGCCATCACACCCGCGCATATCTCATGTGCCTTGCAATCACGGGCGCGGCGGCGCCGCGCCATGGCTTCCTAGTTTCACATAAGTCACACGGTGCTATCTTTCTGTGCGCTCATTTCACGGTGCATATGCTGTATCCCCTTGGCATAGTCCCTGGTGGTGTCGAGGAAGTAGAGCAGCGCACATGAGAACGCTGCAGGCGAGCATGGGGATGGGCCCGAAGGTCCACATGAAGAGCGCGAGCGAGACGTATAAGGCGCGGAGCCCCAGCGACCAGGCGTGGCTGCCGCGGTTCACCGTGCGCGCCACGTACGCCGCGAAATCCTCCGCCGCGCCTTCCTCCGGCGGCAGGCCCAGCAGGAAGCTGGCGTGCGCGTACAGCCGGATGGCCTGCACGTTGCAGACGAAGGCGAGCATGAAGCAAAGCGAGATGGCCAGGTACTTGACCGCGAACACCTGCCCCGTCTTGCTCCCGTACACCATCATCGGCGACGATGAAGACGA

>FLPB002146L13-b0FSP_20011109 (FI167544 )

CCACTTGAACTTTCAAGTATGTTAATCGGACCGTCTTGGTGAACCATGTTCATTATATGAACCATCTACTATCTGCTTCCATGTCATCCTTCAGTTTCCATCTCTGCATTGTTCACAGTAATAATGTGTTTGCAACTTTTTTAAGCTATATGCAAATTATACCACGGTCTTGCTATAAAAAGTATGCCCATAGCATCTCACAGAACTTATAGCATCTTTCCCAAGTCTATCGGCCAATGAAACATGTTCTACCAGTTCCAAAATAGCCAATCACCTTACAAAAGAATTTTTATTCATTAGCCACACCAATGGCGCGGCGTGCCGCCGCGCTAGCCTTTGCTAGTTAACCTAAACACGATGAGAGGGAGATAAAGATGGACCTGCAACAACAGTAGGACTTCTGTGCAACACAATCACTAGGAGGAGACACATAACGGCTGTTAGACATATGCTTTGTA

>FLPB002213E03-b0FSP_20011109 (FI167545 )

ATAGTATTTCTACTTTATATAAACAATATTTCCACAACATCACTTATGCGGTACTTTCGTACCCTGAATTCCTGAATATTGCGGTACTTCTACAATAGGTTATCCAACAATATAATAGCCAAGGCCGCGGCGTGCCGCCGCGCATACCTTCCTAGTTGATAGTTATGCTAGGAGCGAGTAAGACTGTTTGCATTATTGCTATTGAAAATTACACAATGAGTTATGTCCCTGTCATTTCAAGCAGTCTCATAAGATTACTTCATCAAGGTAGGGCAGAGACTGCCACACTAATCCAACCACCACAGGGGTCTTTCTATGAGAATATTGACAGAGACTGCCCCACTAATCCAACCAGCACAGCCTTTTTGGTTGAAGGATCAGTTCCTCTTGGCTTTCTGGGTCACTCATATTCTTCTTTGGGAATTAAACTCCCCCTTGAGTTAACCGGGCAAAAGTTTCTTAATTCATACATCTCATTGACAACTTTACGCACAGAGAATAAAAGATCTGGGCGGAGCATTGTATGGTCTGGGGATGTTTGTATTGCTTTTCATGGAGCCTATTCGCCAACTCGTG

>FLPB002269P03-g0RSP_20011203 (FI167546 )

TCTGCTCCTCTGCATACATACAGACACGTGAGCTAACTTTCAGACACAGCGACTTACTTTCGAGTGCATGCATGTACATCCAAAACAGTAATTCCAAACTACACCTCCTTTTACCATCCTGAGTGGCGCCAAATCACTCACCGAATGCCACTGCCAGCTGCAACCTGCTCCTGTTGTCAGCATAGAGCCAGCAGCTACCTATCCTCAGCCGAGAGCCACAGCAATATTTCGTATAGATTGTAAAAGGGAAGGAGAAAATATGGTATATAACATCGGTTCTCTCTACTGGTCAGCTCATTATCGCAAACCTAGATTCAACAAAAAAAATATTTATGCATCGGTTATCTGAAACAGAAACCAACAATAAATATCGACTTCAGATAACATCCAGTACTAGCTCCGTTTCATCTCATTTGAGCTTCACAAGGATGGAAAACAGCAATAAGAAATACCACTTACCTCACTATTACCAGACTCCAGCGCGGCGTGCCGCCGCGCCTCCATTTGCTAGTTGTTATCAAATTATATTGATATTCAAATAATTTAGAATCTGCAAAACAAAACAGAAATCAATTTTAAGAC

>FLPB002361L24-b0FSP_20011203 (FI167547 )

AAAGGGGAGCTGCATCGGTTCAGTATTTGATGTATCCGTACAAGTTGATACACTATCGTACGAATCACATTCTGTTTAAGATTGGGTTGTAATAAGTTTTATGTGTTCTGTACCAGTTGTCAGGATCAAATGGTAACGTGATTTATAACATTCGTGTTCTTTTTTTTTCTCTGTGTTTCTCTTTATAGTCGGTATCAACTTGGTCAGTACCATGTCACTTTCATCAATTTAGACGGCATGAATTCATTGTTCTGAATTTTGCACAGATGAGCAATTCTACATGTTGAAGAATAAGCTTTGAGGGTTGCCTCTGTCCTTTTTTTGCCTACATTTTATTTACAACTTTTGTTAAGTGAAACAATCTTCTTCTCTCTGGAGGCAAACACTATCACACATTTCCCTGCAAGCATTTGTATCGCCACGGGCGCGGCGTGTCGCCGCGTAAAACCTTCCTAGTATCACTAACAGCACTACCGGTTTACTGGAGTCACATGAAACGTTTCCTAGATTGGGCTCAAGATCAGATAACGTGTTCTTGGCGGCCTAACCTGACCATGCAACCAGATGGCATTCGGTTCACCGGTGACTCGACAAACTGTAAAACTAAGGCTGGAAACCATGTCTAGGAGGTAGGCATATAGATGTGTAGCGCGTGTGTGTAAGGATTTGCTGGTTCTCACCGAGCTCGTCGCAGTCCGTGGTTGAAGTCGGGAGCCAGAGGAC

>FLPB002125I18-g0RSP_20011203 (FI167548 )

AGGTAATAAGGTATCATGGGTGAAGCACAGAATTGTTCCTAACCAAGCCACCACTGCCTTCTTCGATAGCTCTATCCATATTTTCTTTGTCCACGGGCGCGGCGTGGCGCCGCGGTTAACTTCCTAGTAAACTATATCAAAGCTAACCCTTTCTACTTAAACCAGAGCGTTTTGGGTACTGTTGCCTTAACGATTTTGTCCAATTAAACATCACCACGTCGTTCTTTTATTACTGTAGATGGATGCATGATTCCTATAGACACGCACCGATGATTTTCCTTTCGCATTTTTTCCCGTGTGTTAAATTCGTGGTGACCGGTAAGTTTCCGGTAATCTCTTTTATTTCTTCCGGTTAATGCCCCTCTTTCTATTATCGTTTCCTATTCTCTACCCGTGATTTCCGGTCTTTCCTTTTTTTATTCGTTTGTTCACTCTGCGTTTATCGTGGGTGGACCCCGCTTGCACCGCTTCCACCCAACAAACGAGGAGCCCGCCGGCCTCTCTACTCTACCGCTTTTCCTCGATCTCTGCCCATTTTCTCGCCTCGGGCTGCTCGCTCTCCCTTCCCCTCCGTTGGGAAGTCGGGTGTCCCCGCTCGCCATGGGTCTGCTGCCC

>FLPB002308I10-g0RSP_20020409 (FI167549 )

TTTTGAAAAGAAAAAAAGGCAAAACAATGCCGCAACGGGCGCGGCGGTTTTTCCGCTTCTCTCGCGATAATTTGTCGATGCAATTTCCCACACACAAAATAGTTGGATGATTCGGGCGCGACGGTTTTTCCGCCTCTCTCGCGATAATTTGTCGACGCAATTTCCCACATAAAAAATAGTTGGATGATTCAATATATTTACCGAACATTTGCGCCATTTAGAGCAAACGGCCAAATGTGGATACTTAGAGGATTAAAAAAACGCAAAAGAATGCCGCCACGGACGCGGCGTGCCGCCGCGCCAAAGCCTACTAGTATATATTGATTGTATGTGGAAAAGTAGCTCTCTCTCTCTCTCATTTTATGTGAATTTCAGAGCAAAATGGACGGTGACGAAAACACCCACATCCAAAAGTGTTTCCTTATATTGATTACCTTTGCTGCAAGGCTGTACAAATAACTTTTCAATCAGTCTAGTTGCAGTACCTAACTAATAATTGGTGTGCTCACAAACTCGTGTATGTACCTGGACTGGACAGGGCTACTAAGAAAGTCGTATCTGCATGTATGCATGACTATGATCAGCTACGTAATTGCAGAAAAGATAATCCAGTCGATCGCACGTATGGAGTACTTGCTAATAAAATACTTTACTGGATGCATAAAACCCATATGCTATTTAATTAGATCGATCAGGTAGTTGCTAATCACATAGTTCACTGGATCCATAAACCTAGATGCTACTAATTAGATATAGATTGATCGAACTATATTACTTACATGGACGGTATCGA

>FLPB002702B09-g0RSP_20020409 (FI167550 )

TTATCGATACCGTCTATCTCAAATAACACGTCCACAGAAAAAAAAAAAGTAACGCCAGACTTTGATAAACAGTAAAATCAACAAAACAATAACCACAATCCAGCGCGGCGTGCCGCCGCGCCTTCATTTGCTAGTGTTTAATTAAGCATCAGCACCACTTCGGATCGGAGTACTAATACTTTTGTTTGTCCTATGCTTCTAAGCACTGTAATTTTTCCAATGTCTCGAACGGTTTTTGACTAGTCAGCGGCTCAGCGCAATCCACATGCATCGACCCGTGAAACCCACATGCTCTGCCAGTCTGCCCCAAACGGCGGGCAACCGTCCGTCAAACTGTGGCCTACCACAGTGGTGTAGCACGTCAGGTACCATCTATTTTCTGCATTCAGTACCGCACAGAATAAGTTGTCAGGACGCAAATCTCAATACAAGATGAATGGTCAAGAAAACGAGCCCATCTGAGCTCGGGACCAGAAACGCCTCTCCCAAGTCCCCTCCTTCACTTTCCGTTCAATCCTCCCCCTTTTCCGTTTCCGTCTCGTCCTCTACCGCCGGCTGCTGCTGACCCTGCCGCTGTTGCTGCTTTCCGTCCGGGCCTCCCTTTCCTGTACGTACGTGCTCCACTCGCCTCTAATTGTCCATGCTTTGTCGCTTGATTTACGGACCAACGCTATGCCTCTTCGCGTGCGTGCGGTTCTCGTTTGGTTTCGAGCACTGCGGCAGCTCGGTGCTGGGAGCCGTCG

>FLPB001082C09-g0RSP_20011109 (FI167551 )

CGCCCAAAACTGCAACATACTGCTACTAGGAAAAGGAGAAGCCAAAGAATGCATCTCCAACTCTGCAGATACTAACATCTACAAACACAATCATCTGCCCTTTGTGAGACACATCAATCTATGTAAAGATGTCACACACACAAAAAAACAAATCTGCTGTATAAAGCCAGAACAAACACAATTCTAAAAAGAAAGAAAAAATACTAAAATCTGCAGGTATTTAACACCGCCAAGAATTACCATCGTCCAGCCACCACATGGCGCGGCGTGCCGCCGCGACCTTCTTTGCTAGTGTTTTTAATAATTAATCAAGTCCATAATTATTATTGGATTTAACTATTCTTGGAAACACAACAAGAAAGTATTTTTACTTTAAGGAGGAAAAAATTAA

>FLPB002357O07-g0RSP_20020416 (FI167552 )

TGCCTTTTTTTTTCTATACAATTTCATCAGTAATATTTGGTGTGATGATCCAATAAGAATATGAGCTGAATATGAGTCATTGCTATTTGGAGTCCTTGGTGTCGGGTCTCCTATGGTGCAGCTCCTAAGCGACATATACACTCATCCAAGACGTTCTCCGGTTACCAAGTGTGTGTCAAGCTCTTGAATCCAGGATTTTTATCATTTCGTTTATTTTCAAGGGTGTAATGAAATTTATGACAGATCACCTATACATGTACCACATGCATATACCCTTGCTTGGTAGATGGTTTCCCTGACAATCATGTTTGAATAATACTGAAAACGATGAACTGACATTTAGGAAACTATGAAATGCACTCCAGGAAAGTACCATCTTTCATTTCTCAACTTACAACCACGTTATCTGCGCGGCGTGCCGCCGCGCCATATTTTGCTAGTTCCCTTTTATCCTCCACCAGACGCACAACAGCACAAGAGCCTGGCCATGGTCGCATGCCGA

>FLPB002031K12-b0FSP_20011203 (FI167553 )

AAAGTACCAGATTCTCATTTTGAATTCCCCTGAAAAATGGTACTCGGTGCAAATTTCAAGATTCAAAGTCGGTTGTTAATACTTACTGTTAGAAAATGTTCTTCAAATGTTTACACAATGTGTATATCTCTTTCTGGGTGATGAATTTAGATTAAGGTTATTCAGATGTATGTTCTATGTTACAAATTCTAACTCTTCTAGGTGCCTACGAGAATGGGCAAATGTATTACTATTGAGAAAATATGTGTATGCCAGTTCGTAGCTTGATTTGAGCTAAAATCATTGGTACTATATTGTTTAAATCTATTTATTTCATTACTGTGGTAATTAACATTTAAATAAGTGGTATATATCCAATTGACTACGGTAAAAACAACTTCAGATCCATTTACTATCAGACCTCTATTGATTTAAAGCATCCTTTATTCTTTAAGTACTCGAGGCTGGCCGCGGCGGCACGCCGCGCTTGTCCTTGCTAGTGTGGCAATGGATTTGTGATGTTGAGCCGGCTCATATCCCTCCAGTTGAAGATCTTCCACCTGATAATGTCCACCATCCACCCGTATAATAGGCCAACTCATACCGTAAAC

Type 4

>FLPB002708I13-b0FSP_20020409 (FI167554 )

CAGCAACTGCAAGAAGCATAACTCAGTTGCTTAGCTTGAAGGTGATGGCTATCTAGGTGAAATTACTTTGTGCTTCATGTGTAGATTGGTTCAAAGAATCAAATCTCATTGTAGGCATGCTACCAAGCTGGAAAAGCAATTGGCTGGAAACTAGGTTTCTCTTACAGATAGCTCAGTGGACAAGAGCTATATCGAAGTTAGCAAAGGCATCCATATTTTGCCAGTTTTGATTTTGTTCTGCACTTCCATGTGTATCAGAGGAAATACCCAGCTGGAAAAGCAATTGGCTGGAAACCAGGTTTCTCTTACAGATAGCTCAGTGGAGAAGAGCTATATCAAAGTTAGCAAAGGCATCCATATTTTGCCAGTTTTGCTTTTGCTCTGCACTGCCATATGTGTATCGGAGGAAATAAGGAGGTGCTTTCTATGTGCTTGTTGTAGATGACCATATTATAGATTTGCTTTATAGTGGAGTTAACGAGTGATGTAAGGGCACTGACTTTGATTTTCTCCAAGCAATAATATAAGGGCACTCGAGTTTGGTTTGTTTCTAAAAAGTGGATATAGATGGAAATAAGATTAGACTGATTGGCCGCCACTGTACGGCGAGGAGGCGCCCCAAGGGGCGCGCCAACCACTAGTATGTACAATTTTGGTTAGAAATGTCTTCATTTACGGGCACTAAAAAGTGTGGGTATATATATAACAGCTAAAGATACAAACATTAATTATAGATTCACATATCTCACCTTTTTTTGTA

>FLPB002429I03-g0RSP_20011203 (FI167555 )

CGCATAGTGCTTGTCATGAGTTTGTTTTATCTTTACAATTTAGATATGTAATGAGCTGGAGAGTGCGGACTTTGTTAATAAAATGAAGATTCATGACTTCTCAGTCCTATCTGCTTGAGGGGTTGGTCAGGGTGAAAAAGGCATGTAAGCTTCTGTCAATTCAAGGCATATCTGGCATAGTGCTTGTCATCAGTTTGTTTTCTAGCTGTGCGAAACAAGCAATACATTCCTGATTGATTAGTAGGGGAAACAATGCAATCTTGCTCTGACGATTCCTCCAGTTTGTAAGGTGCTATGTATACGGTTTGCATCACATAATAGTTGTTCACTGAGATGGTTGATTGCTGTTGTAAGCTATGCTGTGTTTATTTTGCTTCATGGTCTGGGTCGGATAATGGACGTTTGCAGTTTACTTTCAAGTTTAAATCAATGGAGGATTAGGCCGCAGCAGACCGGTGAGGAGGCGCCCCATGGGGCGCCCCAACCACTAGTATATATGGAAGTCGTCTCCCTCTTCAACCCCCACTAGCAATGCCTCAATGACCATCCAGGCATCCACCACGTACATCTCTATGGAGCTCAGCCCCAAGTAGCAGTCTGCCATGTATACAGTACTCTTGGATTTTCAAAAATATTGCAGCCCCCGAAGAGATCATCATGAACCAAAATAGAGTGATGGCCGCCCTGTCGACCATGCCGTTCAAGACAAGTCGCTGGCCGTCAGCATACTCCAG

>FLPB002506F09-b0FSP_20020220 (FI167556 )

GTCCTTCCTTCATAGAATTATTCTATTCCTGCCTAGCATACACTGACTATGGCTCATGCTGGAACACAAGATTAGTCACTCGCCTAAGCGGCTTGGGGGAGATTCACCGGTGCAGGGTGCGCTTCCAGATATTGAGGCGCATCTGATTTTTGTTTACTGCCCTTTTTCACACTATAAGTAATTAAATTTAGTAGTAGTAGTAGTTAATTGGTAGAGCTGTGCATAGCTTTAGACTTTTTCTCACACTGCAAATCCGTTGCTGACAGAACGATTGTATTGATTTGTTCTTATTCTTTGTGTTGTCACTATTATGTAAGAAAAACATGACTCAAACCATATAAAATCATCAGGTGAACAATCCTCGAATGTGTCATGTCATCCTTGTTTCACTGAACAAGAGCTGACATCAAGTCTTCAAATTGGGGCGCACATACCCGGCCAGGAGGCGCCCCAAGGGGCGCCCCAACCACTAGTGTAGTATCATTTTGGAAGATGAGATCGACGGTGCTGAGTGATGATGCGCCCTGTGTACTTGCGTACCTTGTTCCTTTTGTCTGCTGCTAGTGTTACTGTAACCCATACTATTCTTCCTGGGCGGTATCATTGCTGCCT

>FLPB002590H23-g0RSP_20020220 (FI167557 )

TTATACCTTGTATGAAATTGAGGAGCATCGAGTAGAGTTGTTTGTGAAAAAAACACTGGTACAGGGTATGATAAAACTGTTGCTATCATGCGTTGTGATTGGGGCGCACAGCTCCAGTGAGGAGGCGCCCCATGGGGCGCCCCAACTACTAGTATATTATAAAAAGGGAGAAGATTGCTGTTCATTGAACATGCCTGCTATCCCTTTCTGATGATCCTGACTGACGGGGGACTGACGAAAATGTTAAAGTGCTGCTTGATTAGCGTACTACTACTTAAAACAGGAACAGGATGGAGTACGTGGGGGAGTTTGCTACTATTTCATGGCTGTGAGCGGGTGTTTCCAGCTGGGAGATGGAGCAGTCGTTGGAGTAGTGCGATCTTGATGGGGGGCCAAGGGAGAGGGGTAGTGTAGTGTAGTGTAGGCCGGTTCTAGGCTCACAAAATGTCTGCCTTTGTGTTTGGGCAATTCCGGACGTATTTGATTTTCGTCAAATAATGGAAACACTTGTAGGACACAGAAAAGCTGTAGCTGTAGGCCCAATTCTGTGCAAAAAAGTGTTGTAGCCCATTGGGTTTTCTGGACTTGATGGTAGCTGGGGCCT

>FLPB003140F01-b0FSP_20020424 (FI167558 )

GTCTAAATTGCTCATGGATCTCTTTAATTGGGTAGTTATTGAGCCGCAAAGGACCAGCGAGGAGGCGCCCCAAGGGGCGCCCCAACTACTAGTGTAGCAGAAAATCTGTGGGTGATTAGTATTTGTGTGTAGTTTGTGGAGGAAGCAGGCAAGTCGACAAGGAAAAGTACTACGTAGTAGTGTTTGTGAATTGGCAGTCGATGAGCAGGGGTCGCGTATGGGTAAATGTTGGAGACGAGTTATGAGGGTGCTGAATTACCAGGTAGCTGTAGCTGGCGTATTTGTTGCTGCCAGTTGGATGTGTGCGTCGAAAGTACAATAGACTCGTGAATTAATAAACTGGTGGCAAAATGGTGCGCGCGGGTCAACTGAAGTGGAGCGGATCATTTTATTTTACCAAACAGACGACGAGGTAGAGGCGAATCCGTTTCATTTCTTTATTCGTTCCGTACGCGCGCGAGGTGACGTGGCAGCAGATCGAGGGATGCGACGCGTAAAGCTCCCGGCCCCCTGCACATCGCGAATTCACTGGGCGTGTACGGGTTCGGATCCCCTGGAGTTGGCCTCAACTCTACCGCATGGAGTTGGGGCAATCCTGACCCTTGTTTCCCCTCTATGGCTTAGATTTCTCCACATCTGTAAATTCTGTTAAAGGCATGGTTTTCTCTTCTGGATA

>FLPB002574L04-b0FSP_20020220 (FI167559 )

TCTTACTGGTTTCTTCTGTTTTGAAGGGGGTTCCTGATTAGTTAGGTGATGTGTAAAGTGGATGAGGAGTCCATCGAGGTTGTATTGACAGTGACACTGCCAGTTGCAGATGTGTAAAGTACTTTTGTGTAACCGATATATTCAATGAAAAAATCGTTTGCTACAAGTGAAGAGTTCTCCTTGAAATTATTAACCCCTCAAGACTTGATTTGCTACCAGTGCATGCATGCATATTGCATTCACATAATGTATTAGTGTTCAGGAGAGAAAGCAACTATTCATGTACAACTAACTAACTGTCAAAACATAGAATATTACGACCAGAGTGGATATGTCATCATGTGTTGCGTTTGGGACGCAGAAGAACGAGGAGGAGGCGCCCCAGGGGGCGCCCCAACCACTAGTGAGTAGTAATTCGAGGGGCAATCACGCATCTTAATCGTGCAACGCACATATTCCTTGGTACGTTTCCCATAAGTACGTCTCCCGGGCCGTCCACAATTTCCAAACCCTTGTGTATCCAGGCTCTCGTGATCATGGGCTTCATGGCGACCACCGCGTTGCTGTCCGCCGCCCGAACCGGCGC

>FLPB002697D12-g0RSP_20020409 (FI167560 )

TTATCGATACCGTCAGTTCTGATGTGGATGCAAGACCACGTAGGAGGCAAGCCAAGGAAACCCGTATTGGGCATTGAATTATTTGTCATTGGACTAGGATTTGAGCGTTGAATTATTTGTCATTGTACTGGGATTAGATCGCACAGCTCCAGTGAGGAGGCGCCCCATGGGGCGCCCCAACCACTAGTTCGTTTCATGTTCATGTGTCATTGTAACATATCCACGAAATACACGTATCTATACTTGGTATACTACGATCGCAGCGCACTGAGAAACAATACACGGCGGAAGGCGCACGCTAGAGCACCCCGAAGAACCGGCCCCAGCACGTGCCGCTCCTCCTCGCCTCCGCTTCATCCTGCTCCCCAGCCGCCGCCGCGGGCAGCGGCGCCTCTCCCCGTACGCCCCAGATCCCTTCGAGGACCTGGTCGAGGCTGGCCGGTACCCCGTACGTGTCCGCCTCGGGCGCGGCGA

>FLPB003139N03-b0FSP_20020430 (FI167561 )

GAATTTTGGAAGTGGCTGAGATTTGTTATTGTTGGACTTCCAAGGTTTTCCATGAGATGGTGCCTTGATAAACAGTTGTCAGCTTGTATTATAATTTAATAGAAGCTGTAAAGTGAAAGTTATTAGTGATTAAAACGGCCAGAATGAGTTTTGATTTGGATGCAAGACCGTTGAGGAGGCATTCCAAGGAAACCTGTATTGAGTTGAATTATTTGTGATTGGACTAGGATTAGATCGCACAGCTCTGGCGAGGAGGCGCCCCATGGGGCGCCCCAACCACTAGTTTGAGTAAAGTATGACATGCCCTAGTTTAGATTTTTACCTTGCTTTAATAGGGTGGAGATACTCTACCTCATATCACATGTGTTGAACTCTTGTTATACATGAGGCTCCGCGAACCAAATCCCAATACATTCCT

>FLPB002448F21-b0FSP_20011203 (FI167562 )

GTCTGGATGTGCTTTACCAGTGTCAGCTTTGTAAATAATTAGTATATCAGAAAGAGGCACTGCCAGGTTGGTGATTGGATGGGATTGGAATGCAGAAGACCAGCGAGGAGGCGCCCCATGGGGCGCCCCAACCACTAGTACAATTTAAACACGTCGGGACCTCCTAGCTAAGCTCTCTGCTCAAGGAACAACAACGCAGTGATAAACTGCTAGCTACTCGAACGTACTGGTACGACGATCAATTGGATTTTCGTAGGATTGTGGACGTACCACGGCAGAGGAAACCTTCGTAGGATATGTGTCAACGCGTGACGATCCCATGGATCTCTTGGCACGTACTAGGATCACGTGCGGTCGTGTGAGCAACGGTGTGCATCAATCAATCTCTGCACTCTGCAGCAATGGGGTGGCTCTGATTCTCTGGGCCAGAGATAACGCACGCCAACCAACCGGGCGGGCCATTTGGCCCACTATCAGTATCAGAGGATGACACGACTGCGCGAGAGGCTACTTGTGAGCTGGGAGAAACTGCTTCAGTCCCAAGTGCCAACTATTCCAAGGAATCTCTGCATCTCATGCAGACAAAAAAAAAAAAA

>FLPB002425K12-g0RSP_20011203 (FI167563 )

GCTTCTTGTGCTCCATAACTGTACGTATATTGTTCAAGAGATTATTTTCGAGTTTTGTGTTGGAAGCCATCAAATCAAGGGTTTCAAATTCAGCACCCAAGTGGTACTATTAATTAATGCTTTTCCACATGCCAAGTTAAACATAAATATAAGCTGAGAAGCTGATTGCAAGAGAATGCTTGTGACCGGTCACCATTTATCATTAGGTGTTGGATTGGATTGCAAAGCACCAGCGAGGAGGCGCCCCATGGGGCGCCCCAACCACTAGTATCCATCATAGTAGTCATAGTATGTGCAACTATCCTCACCATAAGGCGACACCATCTATAGTCGTTCAGACTACTCGACGGTTCGGCAGTACTCTGAGCGAAAGGCTCTTCATTATGCCATAGAGCACCTGCTCCATAACTAGTGACTACTTCCGGCTGCTTTTCCCAGGCATGAGTCTTGTAAACATAAGATTTTATTTCGGACAAATTCGCATAGTAGATGTTGCCGGCTTGGATGGTATGGACCTTGCTGTTGTCGACGGAGACACACCGGTTCATGCCGACAAAGAAGGTGTGGCTGGCGAATGTTTCTGACCGGCTCGAG

Type 5

>FLPB002007H19-b0FSP_20011203 (FI167564 )

GTCAATGAGGACGAAAGTCCAATAAGATACAAACATTGAAGACCATTTTCACGAAAAATCAAATAATTTTAATAATTCTGAAACCAACGGAAATATATACTACATTATACTAAGCTGAATTTAAAATGTTTGAAATTAAATCAAACATGATAGAAAGAATTGTCTACAGAATCTAGCCGCGCAAATGCGCGGGCCACTCCGCTAGTGTTGTACTAATGTAGTAGTATAAGAAATCAGCCGAGTTTTTACACCAACAGGTATATCACTTACGTGTGGTGCTCATGCGTGAGGTCCCACACTTCAGTGAGCACAAGTCAAGTGCGGTAGGTACGTAAACTCCAAGCCATCTTCTTGTGTCAAGAGAAGATGCATCAGGATACGTATTGGAAGTCTCTGGGTCCTTCAGGATCCTTCGGTTGTCCCTCTCACAAAAAATACCTCTCTCATTCTCGGCCT

>FLPB002666D08-b0FSP_20020409 (FI167565 )

CTGGGAAANGTATTTGGCTAAGAAATAAATAAGTAAATCCTCACATGGTGTGTTTAGGAAACATGTTTTTTAAGAAGTTGAAAGCATTTGAAAAATAGACAATTAATACAATACATTTACAAAGATGTGAACAAAACAATACACACATCTAGCCGCGCAAATGCGCGGGCCACCTTGCTAGTTATATAATTAAAACCGAAGACAAATAACGGCTCAGATTTAGCACTTTAAAGAGAACGGTTCAGATTTAAAGAAAAAAGAAATTAAATTACCCACCGCTGGCATTAAAATAAAAACAACTTATCCTCTCACACCGTTACGTTTTTCCTATTTTCTTCGCGTTGTGGCCGCCGTATCCTGGAGTCCTACCTAGATCGTTCCAGAGCTTACCTAGGTCAATGGAGGCTTGCAGGATAGATAACAAATCAAAAGATATCATCCAACGTGCAGTGAAGCAATTTGCCGCCGATGATTTATTCTAGCGTTTCGCATGCTGCCGCGGCCGTCTATTCCGATTCCCCGCGATGCAGTGTACTCAGGCATGCTACTGGTATTCAGTGTGCCTAGGGTATCCTCAACTCGACATCCAGTCTGCCATGTGCATGCACGTCAAAGTTAGTGTGGTTTTCAATCTCACGCATACAACATAAACCATGCAGGCAGCCATACTGGATGGATCCATCAATCCAGCCAAATAGCTCAAAGCCAGGTAAATAAATTTGAGATTCAAATTTCCATCGGAGTACTGCATCTAC

>FLPB002683H18-g0RSP_20020409 (FI167566 )

ATCAAGAATGGGCATAATCAATAGGATATGCCACTGATGCATCAAGCTGGTACATCACCGGTCTTTTTTTTTCTTTTACTAGGCATGACAAAATAACTACACCAGTGATTCGGCACACTTCATAGCTAAAGTTGTGGAGTTAATACAGGTTCAATTAAGTTCTCCAGGTATGCATTTATTCATGTGAAGATGTTTCAGCACCTGGATAAAAACGTACGTGCGAGCAGTAGGGAAGCTCTAAAAACTACATACCATAATATTTAAACATAATTCGATTACTATTTCTCTTAATATTTTCTGATAAAGTTAGCAAAAGGAGAAAAAAAAGCACATTAGATGTGAAGATTCATAATATATATATCAGTACTAGCGGGTCACCCATCCAAGAGTACTTATTTAATTTCATTAATTCTCCAATAAAGTATTAGAGGATGATTTAATGTGTATAGAATGCAATGAATTAGATACAGATCTATAAAGAATAGGCAAAAATAAGGAAATACAATAAATGAAATAGGAAAGGAATTAAATATTAGTTGTGCAGTCAAATCATCATAAATCTAGCCGCGCAAATGCGCGGGCCACTAAGCTAGTTCTTTAAAAGAAGTTCATCATGTTTTTCTGCCTGGAGGAGAACATGTATAAGTTCAGAATAAACTTGGTAATTTCTGGCACGAAAATTGTTGCTGTAGGATCCTATCAGCTGGGAGCATAGTTGATAGAGTCTTTTGTAATTCTCCTCTTCTGAAGGTTCCG

>FLPB002138M04-g0RSP_20011109 (FI167567 )

CGACACGCATCAAGGAAGAGAACATATGGTTTATGAAAACATATGGGTTAGATAGAAGCTATCTTATTTCTCTAACTCATCATGGAGAACTTTTCAGCACATATTGATGCAAGTATCAATATATTAAGATTTACAAGAAAATCTATACATGGTTTTGCATCCATCATCCGCTTTCACATCGAAGGTATCATCATCAATATAATGCACCTACTTATGTTATCAAGGTTCAATAGAATTTAAGACTACAAATTTTATATAACGTCTAATTCGTAATGAATTAAATTTAATAACTGATGAGTGAATATTGAAATAAAGTTAAAAGAGAGTAAGTTTAAAAATAATTCACAGAAGCTAGCCGCGCAAATGCGCGGGCCATGCAGCTAGTTTTACGTATTTTATATTTATGGTCATATGTGGGCCAATTACGGATTTTATTAAGTTGAGTCAGTTTTGGTTTGGGCCTGT

>FLPB002256F15-g0RSP_20011109 (FI167568 )

TGATTTTCTTATTGGAAATATCTTCTTTTTGATTTAACACCAAGAATTGAGGAATCATTGAAGATCAAGGTTCATATATTAGTCTAAAAGCCTATGATTTTTAGGGCATGCCAAAACAAAATGGTTCAATCCTCCATTGTCTCACATCGAAGTTCTAACTAAAAACCTTAATACATCTCACGAATTTTCTTTGCAAACTCGAAACTTTTAGAGGATAATTTAGTAACATGTTATTACCCTGAAGCAAATAACCGCCAAAGAAAACCTTCGCAAATCTAGCCGCGCAAATGCGCGGGTCATCCTGCTAGTTTCTTTTATGAGATATGCTAGTAGGATGGCAAAATACATTACTTATCAGAAGTGTGTATTAAAGGTGTATATAAAATACAACCAAAAGTTTTGCTAGTTCG

>FLPB002538O15-b0FSP_20020220 (FI167569 )

TGGATGATACGACATCTGATGGATTCCATAATGTGATGACTATTACAGGAAGATATGGACAACGCCATCAACAAAGTCAAAAGGAATGATACACGCTCGATCCCCTTAAAAGCTAAGGAAAAAAGGCATGTGCATTGTGTAACTGACAATCATAAGTACAAGATGTAAAATTATTTCAGATTTTTGTCTATGGTGGTAATACTTGGCTACCCAAAAAGCTTTCACTCAAACTTCTCTAGGTTTTGACAACTAATAAGTAAAACTTATATACTTCTGAAATACATACATGATGTAAACAATACTATCTTCACAACGCTAGCCGCGCAAATGCGCGGGTCAGCCTGCTAGTTGCTTTAGATTATAATGGTTTGAATTTTGATGATTGCCACATCTCTGAAGTTATAAAGTTCTTGCAAAAACTTGCTAAAAGTCCTAATGCTAGTGCTATAAATTTGGCTTTCACGCATCATATTACAAATGCTCTCATAAAAGCTAGAGAAGAGAAATTAGAGCGCGAAGCCTCTATTCCTAAAAAGCTAGAGGATGGTTGGGAGCCCATCATTAAGATGAAGGTTAAAGATTTTGATTGTAATGCTTTATGTGATCTTGGTGCAAGTATTTCTGTTATGCCTAAGAAAATTTATAATATGCTTGACTTGCCACCGCTGAAAAATTGTTATTTGGATGTTAATCTTGCTGATCATTCTACAAAGAAACCTTTGGGTAAAGTTGATAATGTTCGCATTACCGGAC

>FLPB002258O21-b0FSP_20011203 (FI167570 )

TCTTCTTTATAGAAAAAAGCATGGTGCTAAATAGTGACCAAAGTTCGTATAGTGAACACCTTAAAAATACCCAAAACATCATTCTCACTAATATCTATAAAATCTAGCCGCGCAAATGCGCGGGTCAACCTGCTAGTTATAGATACGTTTGAGACGTATCAATCATCACTGGAGGGCTCTACATCACCATGCCCGCCTCCGGACTGATGCGTGAGTAGTTCATCCTTGGACTATGGGTCCATAGCAGTAGCTTGATGGCTGTCTTCTCCTATTATTCCATAACGTTTAGATCTTGTGAGCTGCCTATCATGATCAAGATCATCTATTTGTAATGCTACATGTTGTGTTTGTTGGGATCCGATGAATATGGAATACTATGTGATAACGCGCGAAGCACATGTCCGTTGGGAACCCCCAAGAGGAAGGTGTGATGCGTACAGCAGCAAGTTTTCCCTCATTAAGAAACCAAGGTTATCGAACCAGTATGAGATGAACGCCACGTGAAGGTTGTTGGTGAAGGAGTGTAGTG

>FLPB002143A19-b0FSP_20011109 (FI167571 )

TCAAATGAATAGTATTGTTTGTCACAATATTTATACTTTCGGAAAATAGAGGCATGTAATTTTTTGTGTCAATAGTATTTTCTGTAGTTCCCCATACACGACATGTAAATTATAATGTATTGTCATATTTAATTTTAATCAAAAATATTTACCTAAAATAGTGATCAAAAGTACACACTACAAATATTTGTGTCAACGACTAGCCGCGCAAATGCGCGGGTCACCCTGCTAGTTTATAATAAAGAACTTAATAATTGTTATTTTGCTCTGGTCAAATGAAAACAATGAGTTGTTCAAATTATGACATTACTCCATGTACGATGGATAAGTTATTATAAATCTTAATGGTGAAACACACATACATAACACTGACGCTAAAATGCCATAAGGCAAATGATTTGAATTCCACTTATTTGTGGAACCGCCATTTAGGTCATGTTAGAAAGGATCGCATGAAGGAACTCCATGCAAATGGATTTTTGGAGTCATTTGATTTGTTGAATCGTCTGACACTTGCAAAATCTTTTCTAAAAGGTAATGACTGAAATACCG

>FLPB002285I11-g0RSP_20020409 (FI167572 )

CGTCGTCATATATCTCTAAGCTTTGACAAGTAATAAGAAAAAATAGCATGGTAAGTAGTATTAGCTTCACAACGATAACAATGTACATACAAATTAAGTTGTGACACATTATTAGGAGATGACATAACAAAAAAATATTTTAGAATCTTATTTTCACCCCTCTTGAAGATATGTAATATAAAAAAACAAATATAACACCAAAAAAGAAATTCATCATAAAGATCTGAGTTGCTACTCACCCTAGAGTCAATCTTAAGGGCCTTAAAAATAATAATCAATGTCATGTATTGGTGAAAGAGAGAGAATTATATTAGGTAATTGGCAAATAGCTGTCATAGTAACGGCATAGACAACAAACTCCACGGTGCATCTTGGTTACCAGATTTTCTACAATTCTAAACTTCGACCTAATCACATCTCAAAAATATTACATAAATAATTAAAAATCACAGGCTAGCCGCGCAAATGCGCGGGTCGCCCTGCTAGTTTAAGCATAATGCCACAAGCATAGGAAAACTAGACAAGCTCAACTTCAAGATTTTTAGCATATAGAGAGGTGTTTTAGTAACATGAAAATTTCTACAACCATATTTTCCTCTCTCATAAAGATTTTCAGTAGCATCATGAACAAACTCAACAATATAACTATCAAATGAAACATTCTTATCATGAGCTATATGCATAAAATTATTACTACTCCCAACATAAGCATAGTCATTCTTATTAATTGTAGTGGGAGCAAATTCAACAAAGTAGCTATCATTATTATTCTCATCACCAT

>FLPB003140A17-b0FSP_20020424 (FI167573 )

GTTGGTTAAATAATATAATTATATTAATGTATAATTGTTCAGTGATTCTGTTTTCCTATTACATACAGATTGAATAATGTAGGATTTAAATTCTATGTGAAGTTAAAATTGTAAGATACATAAAGAACATTATGAGGAAATGTTCAGGTTTTAATGTCTAAATATGTTTTTTACAAGAGTTGTAAGACAAATTGAGAAAAAGGAGAAAATAAAAATATCCTAATATTTGTTATTAAGTATCTTAAGTATTATATGAGTCCTCTAAATATAATACAATAATCTAGCCGCGCAAATGCGCGGGTGACCTTCCTAGTTCATTTAAATTGATAAACAAGGTCTAGACATATTCAAACTAGCAGGAAAATCATGTATCTACCCCCTAACCCTAAACTCTGTCTTATGAAGTCAAGCATGCATGAAGAGAAGTGGTTTTTGGTTTCAAGCATGGAAATTTCAAAAACCCTCCCAAGAGCTACATATTGGAGTGACTAAGAAGGATAGATGTTTAATTTTTCATATTCATGTTTTAAACTTGATTAAATCATGGTCAGACCTAGGATTTGACCAAGATTCAAATGGGCATAAAAATTCAAAAAATAAATGAAAAACCAATGCACATAGTCATCTTATGTCATCTAGTAAGCATTCAATTAAGTTGATAAACAATGTCT

>FLPB001016F12-b0FSP_20020220 (FI167574 )

GTCTATTACTTTCCCTCATTATTGCAACAAAGACACTAGCCATGTTCTGCTTGACAAAGACGGTTCTTGATGTATTTATCTCTGCTCTATTTTTGATGTTCTACTTAGAGCTTTCTTCTTCTTTGTTGCCTGATTTGTTCTTCTTGAATGCATCAAACTTTAATGATCAATTAAGTAGTAATGACATTTATTGAATATAAGTGAATTCAAATTTATTAAAAAGTAAAAATGGCTTCCTACAATTTAATTGTCAGAGGAATATTTACTAGGCTATCTAGATTCATATAAATTTAATGGATAATAAAACAATTAAAAAGAGAGTCAAGATGTCGCCCGCGCATGTGCGCGGGCCACTTTGCTAGTTGAATTAGGAAAAAGATAACCTGAGTTATTTTGAATGATGGGACGTCACAATCACTAATGAAAGATAATTGTGGGAAACTGAAATCTATTCAATAAAATGGGAGAAAAAATGTATCAAAAACAAAATTAATTATACCATTCTGCAGATTGTTCACCGATATAAACAAAGAACTGTTTGATCTGCATGTAGGTACATCCAATCAAGGCTTTTTCCCCGATCTCT

>FLPB002168I09-g0RSP_20011109 (FI167575 )

CAACCATAATAGTTGAGCAGTAATTTACGCATATACATTGGGATAAATATATTTTAAAAAACTATAACTAATTAAAAAAATATAGGGTCAGTACTATTCTTGATACCAAAATCTATTAAAATGAAACAAAACAAAAAAGTTAGTAATTTGAACACGCCATCCTAAGAATTTAGATAAATGCCTTATTGTTTTATCAAGTCTCTTCCGTGGATAGTACAAGAAATTTAAATATACCATCAAGGTGTCGCCCGCGCATGTGCGCGGGCCACTGTGCTAGTTTGAGAAAAAAAGTAATATTAAAAAAAAATCGGCGTTCTGATGAGGAGCACTAGCAGTCCAATGGGCTGTAGACAACATGGAAAAATTACTACATATATGAAGGACAGAAGAAATGCGGCGCTGGGAGAGCTCCTCGCACTGACCAATCGTATCGCGCCATGTCGCGCACAATAAACAGTACCTTATGAACAGTACAACCAGTGCCGGATGAACAGTACCCGACAAACAGTACACACGTGAAAAGTACGTTCTAATTCAACCTTTAACTCCCAATGCACAGTTTTTTTTTGAAAATTTTCCGATGCAAACCTTTTACTTCAAACTTTTCGACCGTATTTTCTAAT

>FLPB001044J10-g0RSP_20010815 (FI167576 )

TACACATCATAGAAATTCATGTATGTTCCGTAATAACTTTTTATTGTAGGCATATTAAGTACTAAATATTTTAGCATACGACATTTGACTAATATACATTATATAATTTACAAAACCTGCTTTTAGCTTAGAACAATGTTTTTTAAGTCGAGTCAATCCTTATTTTCGAGGTTACTTATAACATTGCATTTTTTTACTCTACTAAACCAGGTTGGTCCGCAAAACAAATCTTAAAATAAATTATTGCATGTTACTTAAAATTTATAAAAACTTAATTAAAAACAATTAAAGTTGATGCCCGCGCATGTGCGCGGGCCACTGTGCTAGTTCAAACAAAAACAAAAACAAAAACAAACCGACGCTCCAAGTAAAGAACACAAGATGTGATTGAATAAAAATATAGTTTCAGGGGAGGAACCTGATGATGTTGTCGATGAAGCAGGGGATGCCTTGGGCATCCCCAAGCTTAGACGCTTGAGTCTTCTTAAAATATGCAGNGGGTGAACCACGGGGTCATCCCCAAGCTTAGAGCTTTCACTCCTCTTGATCATAGTATATCATTCTCCTCTCTTGACCCTTGAAAACTTCCTTCACACCAAACTTCAAGCAAACTCATTAGAGGGTTAGTGCACAATTAATAATTCACACATTCAGAGGTGACACAAACA

>FLPB002450F10-g0RSP_20020220 (FI167577 )

TGAATATTCAGCATCCCTTAAATTGTATAACTCTATATATAACACATTTAGAAATAAAACAGTACAAGATCAATTGAGTAGTAATTTTAGCACATGTATTGGGTTAGATAAACTAAAAAAGGAACTCATCAAAATAAAAATAAAAGTATTAAAATGTTAGAATGCCCTCCTACGGTTTACTTATGAGCCGAATTTTAATTGTTATATCGGGAATGGTTTCTCTTCAATGGATAAAAGACCATGAAGATGTTGCCCGCGCATGTGCGCGGGCCAATTTGCTAGTTAAATTAATGATTACTGCGCAGGAAAATGATTTCTAAAATCTTAAATGACTTAAGCATGTTGCAAAACAAAAAGTGCTACAGCAAGTAAAATAAGTGGAGGGAGAAAGAAAGAAAGAAATGTTGTTTAGCTCTCTTATGCATTAAGATATACTTGTTAACAAGGTTGATCAAGTCTTGCTACCAAATAAATTTGATATGACATAAGAAGAAATAAACCTCAAATCAATCATGTTACCTTATATTGAATTGATATGGATCCAATCACAAGAGTTTGATATTCTTCTTTAGGCTCATATATAGGACAATCAATGGATCCCACTTTGATAATTTTCACATTAAAAATTGTATTAACTCCACATACTTTATCAATCCTCTTGGGAAAATAGACAGTGTGTTCCTTATCATCAACA

Type 6

>FLPB002110F05-g0RSP_20011203 (FI167578 )

AACACACAATAGGTCGCAAAACATGGTCGGAACTGCCTTTAATGGCATTAATTGAGAGGTTAACAACTTGGAAGTGTCGGATATTTTCAGCTGTTTATCATTTGCTGTATGACAAAATGTATTTGAACTATGTAATTAAGGATATATTATTAATGATTATAATGTATACTTTCGGTACTTGACAAAAGAGCTAAGTTGTTTCATAAGATGCAACAATATGAAATTTAATGTTCCCAATATGTTTGACTATTCATTAGTTCAGACGTGCATTGCACGTGCAATCTTACTAGTTACTACTAAAACGACAACTTAGACATCCATGCCAATTTTGTTTCCTCATTTTTCCCTCGTCAACATAGCTCTTGATAAAGCCATATGTTTTAAGTTTTCAAAGGATGCCTAGCTTTCCAGATACTTTGTGTGTAGCTCTAGATACATTTCTTTCCAGGAATTGGTACACATACTTGGTCGAGAAAAGCTTAAGCCGTCAGAGACCTAATGACACCATCCGACACTGGAGAAATATGCATTTGAATTG

>FLPB002685H14-b0FSP_20020409 (FI167579 )

AGTTAGCAGAAAAGGTCCACATGACAACATCGAATGTCGCACACTATGTTTTTGATCGGAGGTCTTAAATCTCCATTTGAGCCACTATCTAACCCTGCACTAATTTATCTGCTGTAGGGAACATTCTCTTCTCCATATTAATTAAGTAGGTATATATTGTATGCTCGCATATTTTTTCTGGAAAATTAAAAAAATATCTTCACAATGTCGTGCTTAATTTGGTTAACACGTGCATTGCACGTGCACAATTACTAGTATATACGAAACGTACGTAAATAGGGAAATAGTGAAGGATGGACTACACCACCGAGTCAGTCCGGAAAAACGAAGTGTTTGATACACATTAAAATTAAGAAGACTACCAAGCCAAAAAAAATTTAAGAAAACTATTTTCTCTCACCCGTTTCCTACTTTACACGTATGAGTCCCCAATCCTCACAGTCCAACTTCTGACTATTTGGTTCTCGAACTGTGCTGCCCAAATCTCTGAGGTGAGGCGCTCTCCATAGCCAAATCGCTGCCGCTGTCGACGTTGCCCGTCGCCTAGGACCGGTGCTTGAGGTGCTGCTGTTGGAGGTGGATATCCTTGTGAGCTAGCCCCCGCTAGCCGAGGGAACTTTCACCTCCATCTAGGTTCCTTTCTTTCTTGCGTAGGATCCCCAATCTTGGTAGCTCCGGCAGCCGGGGGCTTTA

>FLPB001008N17-g0RSP_20010815 (FI167580 )

GGTTTTTTGTACATTAAAATAGAGAGCTCATTTGACGGCAAATGATTTTGTATACTATGTTATTGAATTAATATATTGTCCATGAAATACTGAAATTTGTGGTTCTCATTTTTTAGAGAAAATTCATGTTCGTCCTACATATTTTTGCTAACGTGCAGTGCACGTGCATATGTACTAGTATATTAAAGATAACCATACAAAAGCAAAATACAACTCAGACGTACGCACTCCAGAAACATCCAGAGATAGCTAGCTTGGACAAATTGCACAAAGCACCCTGGAAAAACAAAAAAACTGAGAAAAGTCCTTCCAGGTCCTTGAGCATTCCGCCGTCGTTGACCTCTGCTCGCACTCCCGTACTGGAGATGGAGAAGACGGCCAGCCCACTTAAAGACCTTGGAGGAGACGCCATCGAAGACAAGCTTCTTTGCAGACCGCAAGATCCATCCGCCGCAACCGACGGAATATATTCCGCTATCGATCATCGGTCGGGGTAAATCCCCAGCGGCCTCCGAGCTCAGCTCCGGCCAACTCCATCGGAGATCGGCGAAGCTTCTCCTTCCCTTCTTCCTCATCACCCGCTCCAAACCAGCCAGCACCTGACATCTCCAGAACGTACCTTCGACAAACTTATTC

>FLPB002404A02-g0RSP_20011203 (FI167581 )

TTTGTGTCCCTCTGTTGTCATTTTGATAAGTTGTGTTATGTGTTACAAGCTATTGAAATAATTATAGTTTCAATGCCATGTTCTAAGCATGTTGGGTACATATGAATGCTCTCCGACGAACAAGTTTATTGATTTCAGGACGAGCGATTTTAAAAAATATTATACGTGCATTGCACGTGCACTTTTACTAGTGTGTGTTAAAAGAAAAGAACCCACTAAAACACAGCCCAAAACACCATCTCCCCTCCGTCTAAATTTTTCTCCGGAGCCACCCTTCTATTTCTGTCCGTACAAGTTCCACACAGCCACCATGAAGCAAGCTCAATGTCGTGATGTTCTGCAATTCTGTTGCTGCTTCTTGTCGATGTAATCTGACGCACACGCACCACGTTCTCCACATCGCGCGACACGGAGCACCCCACGAACACGCAGTTCCGCCGCTCAAGTTCCACGTTGTCTATCACGTTGGCTTCAAAACTGAAGCGCGTTCTTTTCTATCACAACAGATTCATAGCCGAGCCGACTGAATCCTTTCGGTGCTATCAAGAAACCCAAGATCAAATCCGGCGAAGTTCTTCCCCTTGTTTGTCTCAATCCAGAACCTGTCTATCGGTTTTGTCAGTTAAAAGTTCAGTTTCGGTAGCACCTGTAGGGCTGTGACTCTATCTCGTTCACTAGCCCAGACCAGCCCAGTGGCTTTGATCCTTTTCTCTATGCAGGTGATCCGGAGATCAAAATCCCCAATGT

>FLPB002065N07-b0FSP_20010827 (FI167582 )

ACATAATTATTTGAATATTGTGAATTTACTTGGATTTTGAGCAATGTTTACTGAAATATCTGTATAAGTATGTTATTGTACCTGAGTGATAATTTGTTATATTGGCATAGTATTAGACCACTTGGTTAATTTGTTTAATTCATTTGACTGCAAAGGTTGAGAAAACAAATTCATCTTACACGTGCATTGCACGTGCATGTTTACTAGTATATATAAGCAGCCGACCATATNGGTGCTGTGCAATCTCTAATATCTCTTCTACAAGAACGTCTGTTTTTTCGTTTCAGTATCTAAAGGTATAATAGATGCCANCCCATGGGGGTCTCAGGTAAATCCAATTTGACTAGCCCTTGATTTTACCCTCGACAAATCTGAACCGTCTCTATTTTTACCGTGGTGGTATAAAAACGCATCCAACAGACGAAACCCAACCCTAGCCGCCTCTGCCCACTCGCCGCGTGCGTCCTATCTCTCTCCCCAATCCCCTTCCTTCCACTCCTCCCCAATGGCTTTCCTCCTCCTCCCCGCGAGCGGCCGGTCGCGCTGATGCCTCCCTGCTCGT

>FLPB002090B14-g0RSP_20011109 (FI167583 )

TGAATCTTAATTTTTATCCGTCGTAGTTTCTTGGCTGGTTCACAAAGTTTTTTCTGGATCCTGGAAATGTACTCCTTATGATTAGTCTAAATTATTTCTTGTAAAGGGGTGGTTTTATACTGTATAAAGAACCGTCCATTCAAATGAACAATTTTTTGGTTCTCAAGTTTTTTGGTTCATGTTTGCTCTACTATGTGGCGATTGTAATGGAAGCACCGTCCATTCAAAAGAACAACTTATTCAAATCATGAAGAGCATGCATGGTCGACAGTTGTAGTTCGTTTATTTTTCTTGGTAGGTATACATAACTTCAATTATTTTAGGATATCTATAGTGTTATGTATGTTAAAATCAATGGAATATGCCATATTAGAAATACGTGCATTGCACGTGCAATATTACTAGTGAATGTCAAAAGTGCCTTTCGCTCCTACGTACAGTGCTTCTGCTGTTTAAAAAAT

>FLPB002342L09-g0RSP_20020513 (FI167584 )

GTCCTATATGCTTTGTGGAATAAGTACTATAAAACGGTAACTTGCAGCAAATGAGTGGTGACTTAAGTATGTCCTATATGCTTTGTGGAATAAGTACTATTAAACGGAAATTTAATAATCTAATTCTTCACAACTTATTTTAGCAAGAAAAATAGTTTACTGGAGGAATATACATATATGGAAATTGATTAGGTAATTGAGGCTTGGTTACATCTTACCAGCATGACATGAAAGATCAAAAAAGAGTGTCGACCCTTTATCTAAACTACTTATAAAAGAGCGATTTTGTATTTTATAAATATGTATTATTACAATTAGTATTAATAGATATTGAGACGTGCATTGCACGTGCATATTTACTAGTATTAATTGGGATGTGTGATATTATGGTAACATAGCTAGTTAGTACCACATCACTCTCTTTCTTCTTTTATTGTCATACCATGTCACCAAAATGCCTTGAGATGTGTTATGTTACCATCTATGTTACCCTCACTATGAGCAATCTTACCTGTTGATGGAGTATGTGGGATAGAGGAGATGTTCCGCCGCCTGGCTAGTTACCACGGGACTGTCATGATAATCAGTGATTAATAAAATAAATATCTAAACAAAAATTATAGCACTTGCTCGCTGACATGGCAAACCGTGGTGAGTTGGATATGTTGTATGTGAAAAGAAATAGATAGTAGGGATGCCCTATTTAGTACTGTAATTGTATTGGATAGGTTGTATGTGAAAAAAAAAACATATAGTAATTAC

>FLPB002479F06-g1RSP_20020409 (FI167585 )

AGATCGCCTCACTATAGCTTTGGCTAAAATTATGAAATTGACCCTGACTTGCGAGTAATATGAATCTAGGGCACAACTCAATTCATATCTGATTTGATGAAAATTTGATGTTGTTCCACCCTAGAAGAAAAATGAATAGACATTGATCGTTTGTGTTTTTTAAGGGACAATAACACATATAATTATACATAACGTGCATGTGCACGTGCTTGATTACTAGTATCTTAGATACTCGTTTTGCAAGTTGTGGTACTAAACTGCACATCAGGTGCAACTTGTAAGGTAGCATGAAGGTGACATGTGGCAGGGAAAAATATATATGGGTGCTTTTGGAAATCCTACAATGCCACATGTCAGCTCTCAAATGATTGTGGACCTATTTGCTCCATCGATACAAGTTGTAGTATCTTAAATACTCAATTTGCAAGTTGTTGCAAGTTGTGGTACTAAACTGCACA

Type 7

>FLPB001049N23-b4FSP_20010827 (FI167586 )

CATATATATGTCATAGAAAAGCATATGTTTATAGACTAGATACATGTGTGCAGACACAAATTAAGAAGTCGGTAGCAGATAAACTCATGTTTAACTCAGTTACATCATTAAATTGTTATAGAATAAACATAGAATAGTAGATTCAACTAGAGTCATACAAATCCTACAATTTCCAGGTTCCTAGATTGTAAAAATAATCAAGAAGCACATATAAACATAAATTGTCTAAGAAAACATAATTGTATAAAACAATATTCATGAGTTGTTGTATATGCGATTCAACAAAACATATATCTCTTGCCCGTGCAACGCACGGGTTGATGACTAGTTCTTATTAATTGTAGTGGGAGCAAATTCAACAAAGTAGCTATCATTATTATTCTCATCACCATAATCATAAAATGTAGGAGGCATAGTATAATCATAATAAACTTTATCCTCAATAGTAGGTGGCACCAAAATACCACTATCATCATAAATGGGAGGCAAAATATCATCAAAGTAAATTTTTTCCTCCATGCTTGGGGGACTAAAAATATCATGATCATCAAAACAAGCTTCCCCAAGATTAAATTCTTCTCATAGCATTAGCAG

>FLPB002409P10-g0RSP_20011203 (FI167587 )

CTAGTATTCCCTTCGAGGTCAACCCTGTGCTGCCATTTTATTTGCTATGTTTGAACTCACAAGGTTATCTGTTGCCAGCCATTGGAAAACAAATAGGTACCCGCCTAGGAAGCTATCCCAACGTAGGCGGAAGCTCAAGAAATTAGTGATTTGGATTTACCAATCTTTCTGAGATTCATCAGGTTTATCCTCTCTAACAAATATTTTGTGCAACAATCACCGACATCGCTAAATTGTACTGGCGTAGGCACGTGGCAGACCACAAGATTTGGGGCAAATCTCCTATTACAAGTGTTCTGTGCATTTTTTTTTTGAAATACACTCTTAAGTTCGCTCATTTAGCCACAAAGAAAAATTTTACAAAATTTAGAGCTCGAATGTTTACTTGTGTTCACCAAAAAGGGAATAAACATGTTAACCTATTTGAAGTGAAAGTAAAATCAATTTTAATTTCTCTCATTTAGCCACAAAGAAATGTTTAACAAAATTCTCAACATTATAGCCCGTGCGGTGCACGGGTTGATGACTAGTTTAATGAATGGCAATGATATTGGAGATCAGCTGTACATGTTGGCCAAGACACCATCTTCGACTATAACGATGAGTTATGCATGACTATGCATGTATCGTGTCCGCAGGTTCCACCGGATTATGCTAGTAATGAATTTCACACCTCCAGAGTTCTCATCATGGACTCTCTGAATATGGATCCAAAGCTTTGGGGTCACATGAGAAATATGCTGCAAAAGTAATTA

>FLPB002048J10-g1RSP_20011109 (FI167588 )

GTTTCTAAATATAAAACGATTTGACGGTACAATTTGTTCACGTGACTAATGTGAAGGATGCAATTCAAAGAGTATAACTCCTTTAATTTCCCTTCAATGTGTAAGTTGGTAATCATGGATACACTATAAAACATACCACCATGCAAGATCAATAATAAAACTCTAAGGCTCACCCGTGCGGAGCACGGGTTGATGACTAGTATATACAAAGGCGGAGACAGGCCCCAGGCAACACGGGCCATGGCCTGGGGCGTGGCCTTCCTAGCAGGCTCATAGTCGTAATTTTGGCCCGGGGCGTAGCCCAGCTCAGCCCAGTTTTGGGTGAATTTTGTCACCGTTTAGCCCAGCTGAGACTCGTTCAGGCTCCAGGTCCAGCCGCCAGGCGAACCGAAAAGAAGCACGGAACGCGCTGCGCGCTGAGCGCCGTTGCCGCCGCCGCCGCCGCTGGCTCGTCGCCTCGTCGGCTCGTCCTGTCGTCCGGC

>FLPB002040A21-b0FSP_20011109 (FI167589 )

TTATATTGCCTCAATAAATACAAATTTGTTCCATCTCAAAAATATTTTTGTCCGGTGCTTTTCTAAGAATTACAAATTGCACATTTCGTCAATTGCTGGAGTGTGCATATGCATGGGTTGGATACCTCGTAGAAATATGAAGCTGAATTATAAGAGCACGCATGCACCAATTAAATATTGCATTTCAAATTAAATTGAATAAAACTATAGTTTGTAAATTCGAGCCCGTGCAGCGCACGGGTTAATGACTAGTATATATAGTAAGCATTAAAGTTGATAAACAAGGTCTAGACATATTCAAACCAGATAACTCATGTATATATCTACCCCTAACCCTAAACTCTGTCGTATGAAGTCAATCATGAAGAGAAGTGGTTTTG

>FLPB002520M17-g0RSP_20020220 (FI167590 )

CATAATAGGTTATGACCCAATAGATGATACACTGATCTTTTCACACAATGGTACCTAACCATGTCATCAACCTATGTTGCATGCTGGTGTGCATTAAATAATAATAATAATAACTGTCATCTAAGGACATAATCTAATGACAAATCTTCTAAAAAAATAATTAATCACAAATTTATGGTAGCAATATGACACTTTTTAGTTATATCCTTTATGTCTAACAAAAATAAAAAGTTACATCCTTGCTTAGTCATTAATCACAGAAAATAACTTATTTTTATTCGATATATTTGATTTAGATAAAATTTCATATATAACAATGTATAAATATAAAATAAGCTAGCCCGTGCAGGTGCACGGGATGATGACTAGTATGGCTAGTTTAACTAGTTGATATTGTCCAAAACTATTAAGTAAATTATATGAAGTCTTATACTTCATTTAAAC

>FLPB002116L04-b0FSP_20010827 (FI167591 )

AAGTAAAAAATAATTATTCCATGCACTGATTCTTATCAACATTGTCTGTATTTGCTCCAAACTGCAACACAAATAAACAATAAGATAAATATAAAAATTCATTGCATTTACTATTATTTCCTAAATACATGCACTACGGTGAAGAACACTGTTATTTTGCTTCACTAATTGTGAATATTACACACATCTAGATGCTATAACGTATCATAAGAATTATATATTTCCTAAATATTTAGCCCGTGCGGCTGCACGGGTTGATGACTAGTATGACTCAATTGTTTACTCATTATCTTTACCATTGCTTCGAATTGCTGCATTCATCTCATGTGCTTTACAATAGTATTGATCAAGATTATGATAGCATGTCTGATACGTCTCCAACGTATCTATAATTTCTGATGTTCCATGCTTGTTTTATGACAATACCTACATGTTTTGTTCACACTTTATGATGATTTTATGCGTTTTCCGGAACTAACCTATTGACGAGATGCCGAAGTGACAGTTCCTGTTTTCTGCTGTTTCTTGGTTTCAGAAATCATAGTAAGGAAATATTCTCGGAATTGGACGAAATCAACGCC

>FLPB002095F24-b0FSP_20011109 (FI167592 )

CAATAGTAAATCGGTTTTCTCCTCATGGGTACATAAACTTTTTAGTTTTATATTGTTAGATTAATAATTTCTTACCATATATCTTTGAATGCTAGCCCGTGCTGGAGCACGGGTTGAGGACTAGTTAGATTAATTGTGTGCTTAGAATAGTTTGTGTGAATATATGTGATGTATGGAATAGCCAATGAATTAGTTGAGTGATTATACTCGTATTTCAAATTTAGACGCTAGCACCGGAGAATACCCGGAGGAAGAAGGTTGCTACCAAGAAGAGGAGGAGGAGAACTTTGAGAACTACCAAGGCAAGCTAATATTCTTGCAAAGTGCAAAGCTCTTTGGGGCAAGGCACCATTAGTCTTACCTTTTCTTACCATAAGCCTATCCCAAGTTTCTACATTACAAGTTTTTACTTGTTTTCTCAAGAAGTTAATTTTATAGTTAACTTTGGTCTAAGTTAAAGATGGTTACTAGAGTAGTAAAATTAGTATCAACTAGCAAAGCAAGGTAGCACCCCTCATGATTAGAGCTAGTGCTAATTATTTAAAACTTGACTACTCTAGATGGGAACAATGTGACTTGAACTGAATTTTTAAACCTTGGAATGAT

>FLPA001041E06-b0FSP_20010509 (FI167593 )

GAAAAATGAAATAAAATGATAGTACTGTTAAATTGATATGATTAGCAGCTTCCCTACGAATGCAAAAAAAATGCANCAAGGTAGAAACTTACCGATATTTTCCACAAAGAATCGGACAAAACTTCAAACAAACTATAACCTATTGAATGATTGCTCATTTCCTTTGTACTATACCATCACAAAGCGTTTACTTAATCCCAAGATTGACAAATACGTGGAAAAGATATACTGTAGTTCCTTTTTCAAGAATAACAAAATAATTATATGTTTTAGTAGTCAAAAAATATTTTATTATGTTTTTCCTAATTGCCACATATTAATATTAAGAACATCTAGCCCGTGCTGGTGCACGGGTTGCCGACTAGTAACTGTATAATTATAACGGTAGTTTTTGTGTTTTCGTGAAGCATGTTCTGGGGCATGGTCGATCAAAACATGATTTTCTCCTCCAGACTGTAGAGATATTCTTAGGCCTTGTCATTGATAAGATTTATAACAGCAGA

>FLPB001079O08-b0FSP_20020220 (FI167594 )

ACATATGACATACACGCATACTAATGTAGATACAAAGTACTAATTTTATATCACCGCAATAGTTATTCATCTAAGAATGTGAAAATAATGTTAGCCCGTGCGGAGGCACGGGTTGATGACTAGTTCTTATTATTTTGGTTGATGACCACCATTCATATCCGAACTTAATTAATTGGAGTACAAATAATTTAGTTTATGATTTCGTGGTAAGCTAGTTTGACAAATTAACCTTCAAAATGGCGATTCGCCTACCATGCATGCATGTTTGGCCTACCTGGGAAATTTTAATTTGTGGTATTGTACACACACTGCACATGATAGCGAATAAAAAAAACACCTATTTGGACCCCTTTGGTCAGCAA
